# Synthesis of Carboranyl-Containing β-Arylaliphatic Acids for Potential Application in BNCT

**DOI:** 10.3390/molecules30153250

**Published:** 2025-08-02

**Authors:** Lana I. Lissovskaya, Ilya V. Korolkov

**Affiliations:** 1 The Institute of Nuclear Physics, Ibragimov Str. 1, Almaty 050032, Kazakhstan; 2 L.N. Gumilyov Eurasian National University, Satbaev Str. 5, Astana 010008, Kazakhstan

**Keywords:** carboranes, β-aryl aliphatic acids, boron neutron capture therapy of cancer, BNCT

## Abstract

One of the promising research areas involving carborane derivatives is boron neutron capture therapy (BNCT). Due to the high boron atom content in carborane molecules, these compounds are considered potential candidates for BNCT-based cancer treatment. Despite ongoing studies on various biologically active carboranyl-containing compounds, the search continues for substances that meet the stringent requirements of effective BNCT agents. In this study, the synthesis of carboranyl-containing derivatives of β-arylaliphatic acids is described, along with the investigation of their reactivity with primary and secondary amines, as well as with metals and their hydroxides. The molecular structures of the synthesized compounds were confirmed using Fourier-transform infrared (FTIR) spectroscopy, nuclear magnetic resonance (NMR) spectroscopy, elemental analysis, and mass spectrometry (LC-MS). Cytotoxicity of the water-soluble compound potassium 3-(2-isopropyl-1,2-dicarba-closo-dodecaboran-1-yl)-3-phenylpropanoate was evaluated using several cell lines, including HdFn and MCF-7.

## 1. Introduction

Cancer remains one of the leading causes of death worldwide, representing a significant global public health challenge [1,2,3]. Based on global demographic projections, it is estimated that the annual number of new cancer cases will reach approximately 420 million by 2025 [4]. This represents a steady and concerning increase in the incidence of cancer over time. In 2018 alone, it was estimated that 18 million new cases of cancer were recorded globally, with approximately 9.5 million cases among men and 8.5 million among women. The number of cancer-related deaths in that year was estimated at 9.6 million, highlighting the urgent need for more effective and targeted treatment approaches [5].

Conventional cancer treatments, such as chemotherapy and radiation therapy, are widely used, but they often lack selectivity and can cause significant damage to healthy tissue. This has led to the search for alternative therapies that can be more precise and have fewer side effects. One promising approach is boron neutron capture therapy (BNCT) [6,7].

BNCT represents a groundbreaking and targeted strategy in cancer treatment, capitalizing on the nuclear interaction between non-radioactive boron-10 isotope (^10^B) and low-energy thermal neutrons. This interaction gives rise to high-energy alpha particles and lithium-7 recoil nuclei. These particles, characterized by their high linear energy transfer (LET) capability, have a range of approximately 5 to 10 microns, corresponding to the dimensions of a single cellular unit. This remarkable precision enables the precise eradication of malignant cells while sparing adjacent healthy tissues from harm [8]. The success of BNCT critically hinges on the development of boron delivery agents that possess several essential characteristics: a high concentration of boron, selective accumulation in tumor cells compared to healthy cells, reduced systemic toxicity, and adequate stability in biological environments [9]. While clinically approved agents such as boronophenylalanine (BPA) and sodium borocaptate (BSH) show promise, their limitations regarding selectivity and uptake in tumors have led to ongoing research into more efficient compounds [10,11,12,13,14,15].

Carboranes, a group of compounds rich in boron, have become a promising foundation for the development of BNCT drugs due to their high boron content and diverse structural possibilities [16]. By attaching biologically active components to carboranes, researchers can create innovative agents that meet the requirements for successful BNCT application. Recent research has focused on a variety of carborane derivatives, including those that have been combined with peptides [17,18], amino acids [17,19], porphyrins [20], nucleosides [21], polymers [22,23], carbohydrates [24], and small organic molecules [25,26,27] to improve their ability to target tumors and enhance their therapeutic effects.

Amino acid and short peptide-based drug delivery systems have gained significant interest as potential platforms for the development of next-generation boron carriers. A growing body of research is focused on the design and development of new delivery systems that incorporate amino acids and peptides modified with boron-containing groups, such as boronate or carborane. Kahl and colleagues developed an efficient methodology for synthesizing 3-amino-1-carboxy-ortho-carborane, along with protected derivatives of all three C-amino-C-carboxycarboranes [28]. Their approach involved the initial formation of carboranyl acids through deprotonation and subsequent carboxylation. These acids were then converted into the corresponding Boc-protected amines using a Curtius rearrangement in the presence of *tert*-butanol. Ujváry and Nachman reported the synthesis of 3-(12-hydroxy-para-carboranyl)propionic acid as a hydrophobic N-terminal tyrosine mimic [29]. In a related study [30], the same group also synthesized 3-[12-(mercaptomethyl)-1,12-dicarba-closo-dodecaboran(12)-1-yl]propionic acid, which, upon oxidation, served as a substitute for Tyr(SO3H) residues commonly present in various bioactive peptides.

Cinnamic acid (CA) is a naturally occurring phenylpropanoid compound characterized by the presence of an α,β-unsaturated carboxylic acid moiety. It has attracted considerable attention due to its broad spectrum of pharmacological properties, including anti-inflammatory [31], antimicrobial [32,33], anticancer [34,35], and antifungal [36] activities. The structural framework of CA comprises three key reactive centers—an aromatic benzene ring, a conjugated double bond, and a carboxylic acid group—which collectively contribute to its high chemical reactivity and biological potential [37]. These functional groups serve as versatile sites for chemical modification, enabling the synthesis of a wide array of bioactive derivatives. Such derivatives have demonstrated significant efficacy in modulating oxidative stress and inflammatory responses, primarily by reducing reactive oxygen species (ROS) and suppressing pro-inflammatory cytokines.

Hydrocinnamic acid, also known as 3-phenylpropanoic acid, is a saturated derivative of cinnamic acid resulting from the hydrogenation of the α, β-unsaturated double bond present in the parent compound [38]. It exhibits characteristics typical of both aromatic compounds and aliphatic carboxylic acids. A key feature of its chemistry is the cooperative involvement of all three structural components, the aromatic ring, aliphatic chain, and carboxyl group, in molecular transformations leading to the synthesis of new compounds [39,40,41]. In some cases, the two-carbon aliphatic chain acts as a spacer between the aromatic ring and carboxylic group, separating and facilitating spatial interactions between these moieties. These through-space effects may account for the biological activity observed in certain hydrocinnamic acid derivatives. These acids have attracted significant attention due to their wide range of applications in the synthesis of complex molecules for medical [42,43], agricultural [44], biological [45,46], and industrial purposes [47]. Moreover, these compounds are not only used in fundamental research but also in routine laboratory practices across various scientific fields.

Recent studies suggest that compounds based on hydrocinnamic acid may also exhibit antiproliferative and anticancer potential, especially when incorporated into hybrid molecular structures with other bioactive components [48,49,50,51]. Furthermore, its adjustable structure allows for the development of new conjugates for drug delivery and therapeutic use. Despite its relative simplicity, hydrocinnamic acid serves as a valuable component in medicinal chemistry, particularly for designing molecules with improved pharmacokinetic properties and reduced toxicity.

Kavaliauskas et al. described the synthesis of 3-((4-hydroxyphenyl)amino)propanoic acid derivatives functionalized with various aromatic and heterocyclic substituents. These compounds demonstrated significant antibacterial and antifungal activity, including effectiveness against multidrug-resistant bacterial and fungal strains scaffolds [52]. Tianyu He and Rabi A. Musah conducted a study evaluating the cytotoxic effects of 2-amino-3-(1,7-dicarba-closododecaboranyl-1-thio)propanoic acid across different concentrations and levels of neutron beam irradiation. The compound’s performance was compared to that of 4-borono-L-phenylalanine (BPA), a widely used boron delivery agent in BNCT, to determine its viability as a potential alternative therapeutic candidate [53]. Blanca Colín-Lozano et al. have synthesized a small series of five 3-[(4-arylmethoxy)phenyl]propanoic acids using an easy and short synthetic route. The compounds were tested in vitro against a set of four proteins identified as key elements in diabetes [54]. Sergey Kuranov and colleagues synthesized a series of bornyl derivatives of *p*-(benzyloxy)phenylpropionic acid, and their hypoglycemic activity was assessed through oral glucose tolerance tests conducted in mice [55].

This paper presents the synthesis and characterization of novel carboranyl-containing derivatives of 3-phenylpropanoic acid: 3-(2-isopropyl-1,2-dicarba-closo-dodecaboran-1-yl)-3-phenylpropanoic acid, 3-(2-isopropyl-1,2-dicarba-closo-dodecaboran-1-yl)-3-(naphthalene-1-yl)propanoic acid, and 3-(2-isopropyl-1,2-dicarba-closo-dodecaboran-1-yl)-3-(4-fluorophenyl)propanoic acid. The reactions of these compounds with various primary and secondary amines, such as methylamine, butylamine, tertiary butylamine, allylamine, cyclohexylamine, ethylenediamine, morpholine, piperidine, and diethylamine, have been investigated. Additionally, the synthesis of the obtained carboranyl-containing derivatives of hydrocinnamic acid with metal and metal hydroxides has been carried out. Some of the salts obtained in this way are highly soluble in water, which is one of the key properties in the case of medical applications. Cytotoxicity studies of the water-soluble compound potassium 2-isopropyl-ortho-carboranyl-3-phenylpropanoate were conducted using MCF-7, HdFn cell lines. This compound exhibits toxicity at concentrations up to 0.069 mM toward human MCF-7 and HDFn cells. However, it does not induce a significant toxic effect on the viability of MCF-7 and HDFn cells at concentrations of 0.06776 mM and 0.05427 mM, respectively. In this context, the current research aims to synthesize and characterize novel carboranyl-containing β-aryl aliphatic acid derivatives, and to assess their chemical reactivity and primary biological activity as potential candidates for BNCT.

## 2. Results and Discussion

### 2.1. Synthesis of Arylidenemalonates

**Scheme 1:**
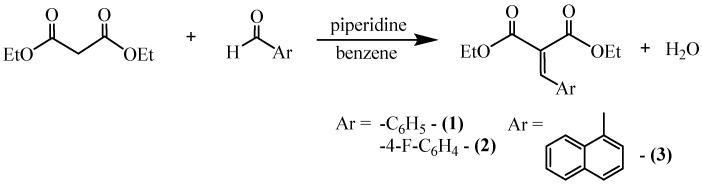
Synthesis of diethyl arylidenemalonates.

The first step involved the synthesis of arylidenemalonates, specifically diethyl 2-benzylidenemalonate (**1**), Diethyl 2-(4-fluorobenzylidene)malonate (**2**), and Diethyl 2-(naphthalen-1-yl)malonate (**3**)—according to the procedure specified in the 2008 study by Kaumanns et al. [56] using piperidine as a catalyst and subsequent treatment with hydrochloric acid with a good yield of more than 60% (Scheme 1). The structures of these malon ether derivatives were confirmed using FTIR and NMR spectroscopy to ensure accuracy. The obtained data are consistent with those reported in previous studies [57,58,59].

### 2.2. Synthesis of Carboranyl-Containing Derivatives of Arylidene Malonates

**Scheme 2:**
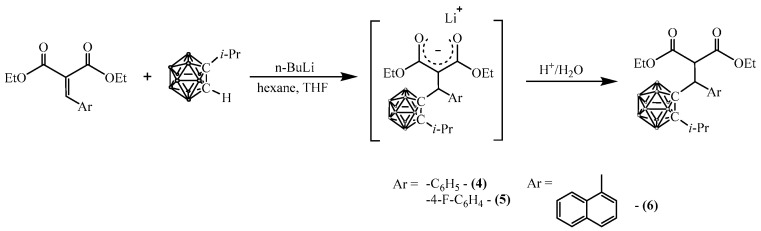
Synthesis of diethyl arylidenemalonates with isopropyl-*o*-carborane.

The interaction of isopropyl-*o*-carborane with an aromatic derivative of malonic ether takes place with the formation of a complex with a lithium ion, while the negative charge is redistributed between the oxygen atoms of the ester group. Furthermore, after treating the reaction mixture with hydrochloric acid, we obtain an addition product at the outlet, resulting in the breaking of the double carbon-carbon bond (Scheme 2). In 2004, A.V.Kazantsev et al. investigated some features of conjugate addition reactions with o-carborane metal derivatives [60].

### 2.3. Synthesis of Carboranyl-Containing Derivatives of 3-Arylpropanoic Acid

In particular, the malonic synthesis—involving successive C-2 functionalization of malonate esters, followed by hydrolysis of one or both ester groups and final decarboxylation—represents a highly effective strategy for producing functionalized esters or related carboxylic acids. Each step of this process is continually being refined to improve stereocontrol, employ more environmentally friendly reaction conditions, and achieve higher yields [61].

The critical step in this multistep sequence is the decarboxylation process, which is typically carried out under harsh thermal conditions (up to 200 °C), using either conventional [62,63] or microwave heating [64,65]. The mechanism begins with an intramolecular electron redistribution, leading to the formation of a six-membered cyclic transition state. Subsequent C–C bond cleavage results in the release of carbon dioxide and the formation of an enol intermediate, which rapidly tautomerizes under acidic conditions to afford the more stable monocarboxylic acid.

**Scheme 3:**
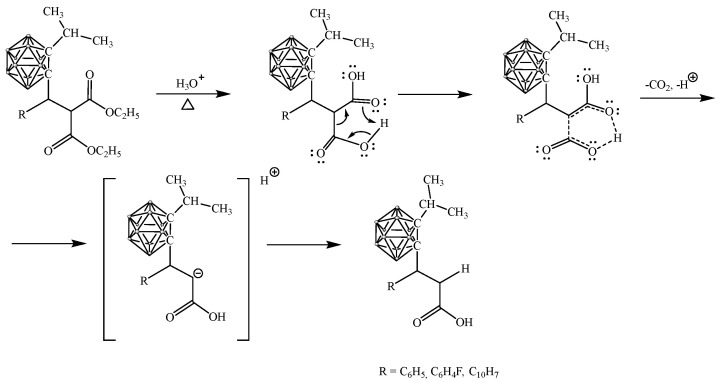
The reaction mechanism of the synthesis of carboranyl-containing derivatives of hydrocinnamic acid.

If carboranyl-containing arylidene malonates are boiled in acetic and hydrobromic acids, the resulting alkylated product undergoes acid-catalyzed hydrolysis of both esters, which leads to the formation of malonic acid. When this malonic acid is heated, it undergoes decarboxylation giving a substituted carboxylic acid (Scheme 3).

**Figure 1:**
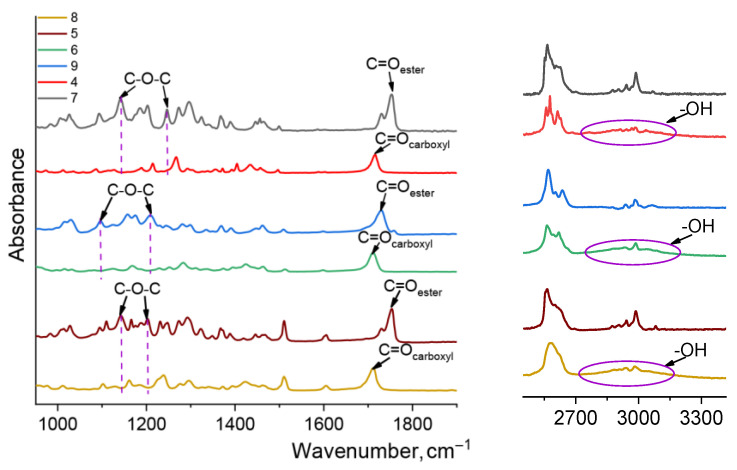
Comparison of FTIR spectra of diethyl α-(isopropyl-*o*-carboranyl)-arylidenemalonates and obtained carboranyl-containing derivatives of 3-arylpropanoic acid.

**Scheme 4:**
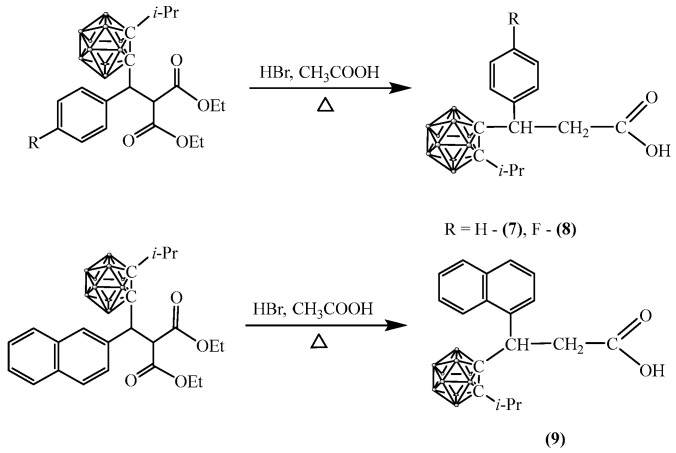
Synthesis of carboranyl-containing derivatives of 3-arylpropanoic acid.

Thus, carboranyl-containing derivatives of 3-phenylpropanoic acid were obtained: 3-(2-isopropyl-1,2-dicarba-closo-dodecaboran-1-yl)-3-phenylpropanoic acid (**7**), 3-(2-isopropyl-1,2-dicarba-closo-dodecaboran-1-yl)-3-(naphthalene-1-yl)propanoic acid (**8**), and 3-(2-isopropyl-1,2-dicarba-closo-dodecaboran-1-yl)-3-(4-fluorophenyl)propanoic acid (**9**) (Scheme 4). The comparative analysis of FTIR spectra demonstrates structural changes in the compounds after synthesis and supports the presence of functional groups consistent with the proposed structures (Figure 1). Based on the analysis of the ^1^H and ^13^C NMR spectra of the obtained products of β-arylaliphatic acids, it is possible to notice the absence of two -CH_3_ bonds, which were observed in the ^1^H NMR spectra~0.55–1.22 ppm and ^13^C NMR spectra~13–14 ppm of diethyl α-(isopropyl-o-carboranyl)- arylidenemalonates. Additionally, referring to the NMR spectra of DEPT-135 starting compounds, it can be noted that the presence of two -CH_2_- bonds in the range of ~61–62 ppm. While in acid products, only one -CH_2_- bond is observed in the range of ~41–42 ppm, which is assumed to belong to the aliphatic chain of 3-arylpropanoic acid. In the ^13^C NMR spectra and APT-WALTZ for carboranyl-containing derivatives of hydrocinnamic acid, one C=O bond is observed in the range of ~171–176 ppm, which indicates the presence of one carboxyl group, whereas in the ^13^C NMR spectra for their starting compounds there are two quaternary carbon atoms double-bonded to an oxygen atom in the range of 165–168 ppm, which belong to the ether group. The presence of a carborane core in the acid products is confirmed by the ^1^H and ^11^B NMR spectra, which show 10 hydrogen atoms in the range of 1.0–4.0 ppm and 10 boron atoms in the range of ~(−5)–(−9) ppm, respectively.

### 2.4. Synthesis of Carboranyl-Containing Ammonium Carboxylate Salts

Ammonium carboxylate salts are an important class of compounds that can be easily prepared through a simple acid-base reaction. This reaction involves the neutralization of carboxylic acids with amines, where the carboxylic acid acts as a donor of protons and the amine acts as an acceptor of protons. The formation of ammonium carboxylate salts occurs when a proton is transferred from the carboxyl group of the carboxylic acid to the nitrogen atom in the amine. This results in the formation of a carboxylate ion (R-COO-) and an ammonium ion (R′-NH_4_^+^), where R and R’ represent different groups. This reaction is typically spontaneous at room temperature and does not require any additional reagents or catalysts. It is a highly convenient and environmentally friendly synthetic route, making it an important tool for the synthesis of various compounds.

The mechanism of ammonium carboxylate salt formation is governed by proton transfer equilibrium. Non-covalent interactions such as hydrogen bonding and electrostatic attraction bring the carboxylic acid and amine molecules into close proximity, stabilizing the transition state and facilitating the proton transfer. The extent of salt formation depends on the relative pKa values of the acid and amine, as well as on the polarity and dielectric constant of the solvent. In polar solvents, complete ionization occurs, while in non-polar media, the salt can exist partially as a hydrogen-bonded complex or fully as separated ions.

The reaction conditions can be carefully adjusted to control the formation and stability of ammonium carboxylate salts. Factors such as solvent choice, temperature, concentration, and water content can all influence the reaction equilibrium. In solid-state or mechanochemical reactions, the two reactants are ground together, often producing crystalline salts with high purity and low waste.

Beyond their straightforward synthesis, ammonium carboxylate salts play a valuable role as intermediates in various synthetic transformations. One notable application is in the formation of amide bonds, where the thermal or coupling agent-mediated dehydration of these salts leads to the creation of amides, which is a crucial step in peptide synthesis and drug development [66,67,68,69]. Additionally, these salts have found use in supramolecular chemistry, ionic liquids, catalysis, and the production of advanced materials due to their adjustable ionic interactions, stability, and solubility.

Odendal et al. conducted a structural analysis of primary ammonium carboxylate salts based on their crystallographic data [70]. Experiments on the formation of salts were carried out using 5 primary amines (propylamine, cyclohexylamine, aniline, benzylamine, and phenylethylamine) in combination with 7 carboxylic acids (formic, acetic, propionic, hexahydrobenzoic, benzoic, phenylacetic, and diphenylacetic acids). Haines group synthesized and structurally analyzed a series of ammonium carboxylate salts to investigate the factors that influence the ability of organic cations to act as effective crystallizing agents for specific functional groups [71]. The study employed phenylethylamine, benzylamine, and aniline as representative amines, which were reacted with either fumaric acid or succinic acid. In all resulting salts, hydrogen bonding was observed between the cationic ammonium donors (NH_3_^+^) and the carboxylate anionic acceptors (COO^−^). Remarkably, the crystal structures revealed consistent packing arrangements and recurring hydrogen-bonding motifs across the entire series. *A. Lemer* also investigated the structural characteristics of ammonium carboxylate salts, demonstrating that three-component salt structures with a ladder-type hydrogen-bonding motif can be achieved by adjusting the amine-to-acid ratio during synthesis [72]. In his study, six distinct salts were synthesized and characterized: 2-propylammonium benzoate, benzylammonium (R)-2-phenylpropionate, (RS)-1-phenylethylammonium naphthalene-1-carboxylate, benzylammonium benzoate–benzoic acid (1/1/1), cyclopropylammonium–benzoate–benzoic acid (1/1/1), and cyclopropylammonium-*ea*-*cis*-cyclohexane-1,4-dicarboxylate-*ee*-*trans*-cyclohexane-1,4-dicarboxylic acid (2/1/1).To summarize, the synthesis of ammonium carboxylate salts from carboxylic acids and amines offers a simple, effective, and flexible method for accessing a diverse range of ionic compounds with wide-ranging applications across various branches of chemistry.

In this study, the reactions of carboranyl-containing 3-arylpropanoic acids with primary or secondary amines and metal hydroxides, which are both strong nucleophiles, can lead to the formation of minor amounts of nido-carborane by-products, as can be seen in ^11^B NMR spectra. Although the closo-carborane framework (e.g., 1,2-C_2_B_10_H_12_) is generally stable compounds, it is well-established that exposure to strong nucleophiles or bases can induce partial deboronation, leading to the formation of nido-carborane species [73,74,75,76]. Nevertheless, through multiple recrystallization and thorough washing steps, these by-products can be minimized or removed.

**Scheme 5:**
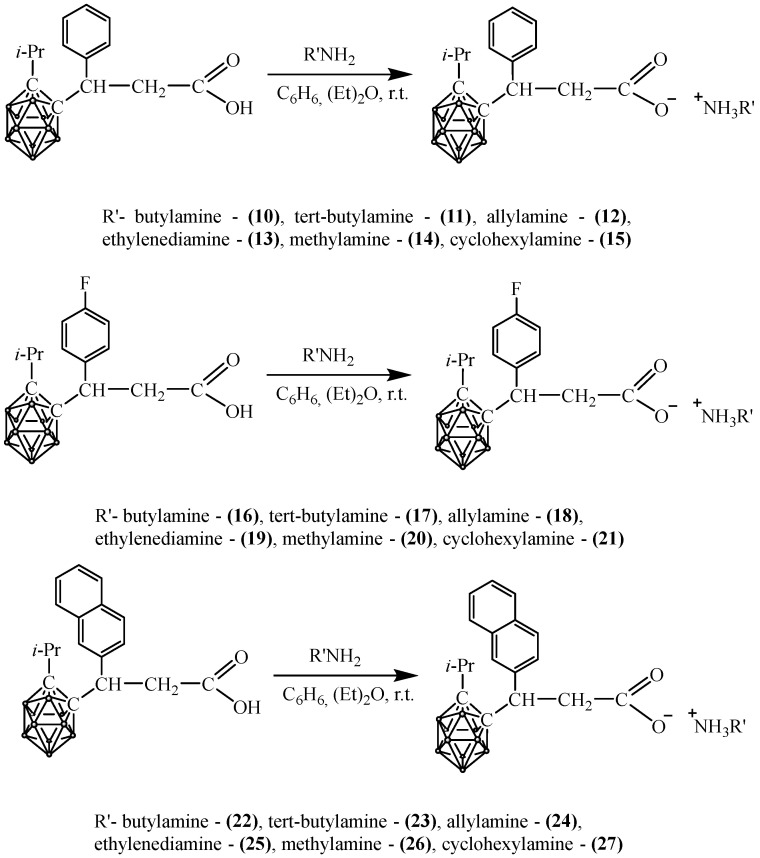
Synthesis of carboranyl-containing derivatives of 3-arylpropanoic acid with primary amines.

The following primary and secondary amines were used to study the reaction of carboranyl-containing derivatives of 3-arylpropanoic acid with amines (Scheme 5): linear aliphatic (methylamine, butylamine, diethylamine), branched (tert-butylamine), unsaturated (allylamine), multifunctional (ethylenediamine), cyclic (cyclohexylamine), and heterocyclic (morpholine, piperidine).

The synthesis of ammonium carboxylate salts from carboranyl-containing derivatives of 3-arylpropanoic acid and primary amines was systematically investigated to evaluate the influence of electronic and steric factors on reaction efficiency.

The formation of the ammonium carboxylates proceeded via direct acid-base neutralization, driven by the proton transfer from the carboranyl-substituted 3-arylpropanoic acid to the primary amine. The significant pK_a_ gap between the carboxylic acid functionality (pK_a_~4–5) and the primary amine (conjugate acid pK_a_~9–11) ensured favorable thermodynamics for salt formation under mild conditions (Table 1).

The introduction of the bulky carborane cluster into the 3-arylpropanoic acid framework introduced steric hindrance around the carboxyl group. This steric effect, while generally not preventing salt formation, could reduce the rate of initial pre-association between the acid and the amine, particularly when solid-state or low-solvent systems were employed. Additionally, the electron-withdrawing nature of the carborane moiety slightly stabilized the carboxyl group, enhancing its acidity and favoring proton transfer.

The presence of a fluorine atom in the phenyl ring has an additional effect on the course of the reaction. The electronegative fluorine atom exerted an inductive electron-withdrawing effect, which lowered the pK_a_ of the carboxylic acid, slightly increasing its acidity. As a result, proton transfer to the primary amine occurred more readily, thereby enhancing the efficiency of salt formation. The small size of the fluorine atom minimized steric interference, allowing unhindered access of the amine to the reactive site.

Replacement of the phenyl ring with a naphthalene moiety altered both electronic and steric profiles of the molecule. The naphthalene ring generally maintains or slightly increases the acidity of the carboxyl group due to its extended conjugation and electron delocalization. However, its larger size introduced notable steric hindrance, especially near the carboxyl group, which affected the approach and binding of the primary amine. Consequently, salt formation with naphthalene derivatives was somewhat slower compared to their phenyl analogs, particularly when bulky primary amines were employed or under conditions of limited solubility.

Thus, the formation of ammonium carboxylate salts in 3-arylpropanoic acid derivatives functionalized by a carborane cluster is largely determined by both electronic and spatial effects. The presence of isopropyl-*o*-carborane and a fluorine atom increases acidity and promotes reactivity, while replacing the phenyl radical with naphthalene introduces spatial constraints that can moderately reduce the reaction rate without completely suppressing salt formation (Table 1).

**Figure 2:**
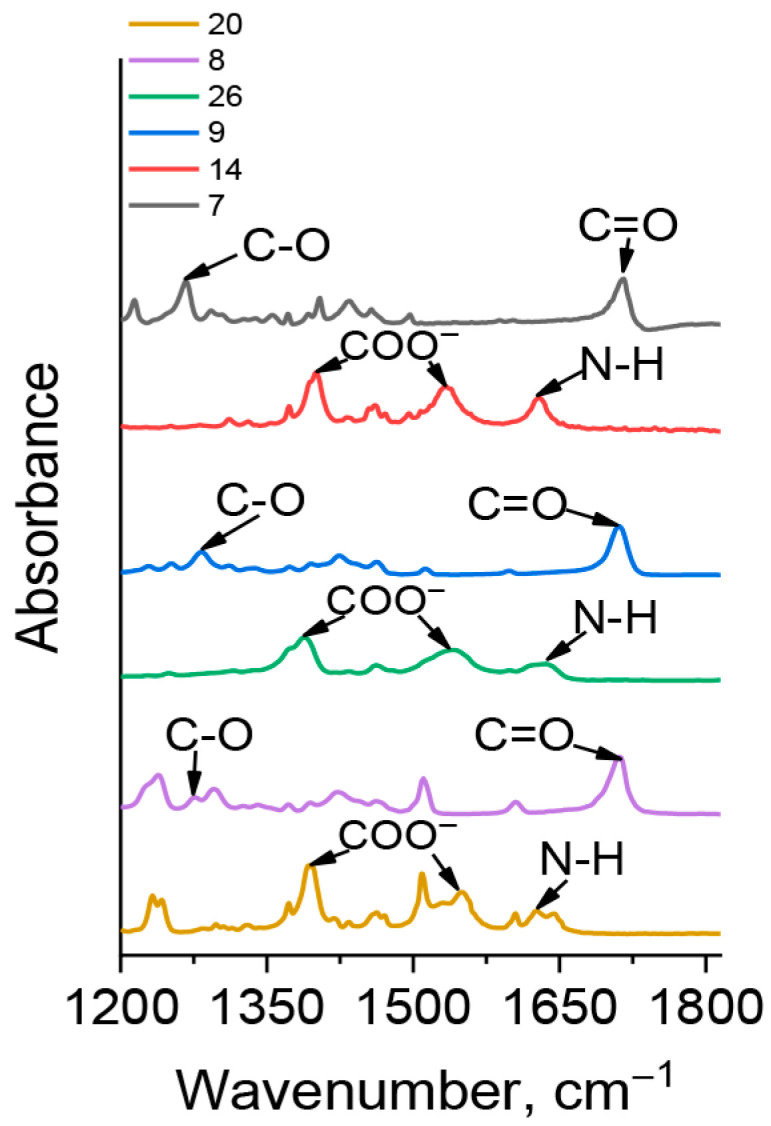
Comparison of FTIR spectra of carboranyl-containing 3-arylpropanoic acid (**7**, **8**, **9**) and obtained Methylammonium 3-(2-isopropyl-1,2-dicarba-closo-dodecaboran-1-yl)-3-phenylpropanoate (**14**), Methylammonium 3-(2-isopropyl-1,2-dicarba-closo-dodecaboran-1-yl)-3-(4-fluorophenyl)propanoate (**20**), and Methylammonium 3-(2-isopropyl-1,2-dicarba-closo-dodecaboran-1-yl)-3-(naphthalene-1-yl)propanoate (**26**).

Ammonium carboxylate salts were obtained by synthesizing carboranyl-containing 3-arylpropanoic acids with primary amines (Scheme 5). The comparative analysis of FTIR spectra demonstrates structural changes in the compounds after synthesis and supports the presence of functional groups consistent with the proposed structures (Figure 2).Analyzing the ^1^H and ^13^C NMR spectra of the obtained salts, it can be noted that the -CH_2_- and C-H bonds in the aliphatic chain of 3-arylpropanoic acid are preserved; thus, the addition of the primary amine does not affect the alpha and beta carbon atoms of the carboranyl containing 3-arylpropanoic acid. For the ^1^H NMR spectra of the starting acids, the peak of the -CH_2_- bond of the aliphatic chain is in the range of ~2.9–3.2 ppm and is represented as a doublet; however, for some ammonium carboxylate salts this peak may shift to the left due to a redistribution of the electron density. The presence of the C-H bond in the ^1^H NMR spectra is confirmed by a peak in the range of ~4–5 ppm. Also, in the ^1^H NMR spectra of salts, peaks in the region of ~6–7 ppm are present, which belong to the protonated amino group, indicating the formation of an ionic bond during the reaction of the initial carboranyl-containing 3-arylpropanoic acids with primary amines. The ^1^H NMR spectra of the ammonium carboxylate salts exhibit no signals in the acidic proton region (~10.35 or 12.30 ppm), indicating that the carboxylic acid has been fully deprotonated. In the NMR spectra of carbon ^13^C, DEPT-135, APT WALTZ, the peak of the -CH_2_- bond of the aliphatic chain, observed in the range of ~41–42 ppm, remains in the same region for the obtained salts, the peak of the C-H bond appears in the range of ~30–40 ppm, which is also visible in the DEPT-90 spectra. Carbon ^13^C NMR spectra and APT-WALTZ of ammonium carboxylate salts show only one peak of the quaternary carbon atom of the carboxylate group in the region of ~175 ppm and two peaks of the quaternary carbon atoms in the region~88–90 ppm, which belong to the carborane core. The presence of a carborane core in the acid products is confirmed by the ^1^H and ^11^B NMR spectra, which show 10 hydrogen atoms in the range of 1.0–4.0 ppm and 10 boron atoms in the range of ~(−5)–(−12) ppm, respectively.

Both methylamine and butylamine exhibited high reactivity with the carboranyl carboxylic acid. Methylamine, being the smallest and least sterically hindered, readily formed the ammonium carboxylate salt at room temperature. Butylamine, with a slightly larger hydrophobic chain, also reacted efficiently, although precipitation occurred more slowly in polar solvents. Both amines demonstrated rapid proton transfer due to favorable acid–base pK_a_ differences and minimal steric hindrance. However, in both cases, the yield of the product during synthesis with 3-(2-isopropyl-1,2-dicarba-closo-dodecaboran-1-yl)-3-(4-fluorophenyl)propanoic acid (**16**, **20**) is lower due to the inductive electron acceptor action of fluorine.

In contrast, tert-butylamine exhibited reduced reactivity due to significant steric bulk near the nucleophilic nitrogen atom. Although the acid–base reaction was thermodynamically favorable, the approach to the carboxylic acid site was sterically impeded, leading to slower salt formation. Nevertheless, in the case of 3-(2-isopropyl-1,2-dicarba-closo-dodecaboran-1-yl)-3-(4-fluorophenyl)propanoic acid (**17**), the presence of a fluorine atom had a positive impact on the reaction.

Despite the favorable basicity and nucleophilicity of allylamine, several factors make it challenging to obtain ammonium carboxylate salts. Firstly, the terminal alkene group present in allylamine can lead to side reactions such as polymerization in air or oxidative degradation. These reactions can consume free amine, reducing the efficiency of acid–base neutralization and leading to a decrease in apparent yield. Additionally, the resulting salt may be highly soluble in the reaction medium, leading to low crystallinity and a decrease in yield if only solid material is desired. The minimum salt yield is observed during the synthesis of allylamine with 3-(2-isopropyl-1,2-dicarba-closo-dodecaboran-1-yl)-3-phenylpropanoic acid (**12**).

The reaction of ethylenediamine with carboranyl-containing 3-arylpropanoic acid derivatives resulted in the formation of pronounced ammonium carboxylate salts. Ethylenediamine, as a bifunctional primary diamine, is capable of interacting with two acidic protons simultaneously, which often leads to the formation of bis(ammonium) dicarboxylate salts or crosslinked supramolecular assemblies depending on the acid structure and stoichiometry. In this case, the ratio of reagents (1/1) leads to the production of a mono(ammonium) carboxylate salt product. When the acid contained bulky substituents, such as an isopropyl-*o*-carborane cage, reduced salt yield or less crystalline material was observed. The steric hindrance around the carboxyl group can prevent optimal proton transfer. The presence of a naphthalene ring slightly increased the acidity of the carboxyl group, and therefore, the reaction with 3-(2-isopropyl-1,2-dicarba-closo-dodecaboran-1-yl)-3-(naphthalene-1-yll)propanoic acid (**25**) gave higher yields.

Cyclohexylamine exhibited intermediate reactivity. Its cyclic structure introduced moderate steric bulk, which somewhat slowed the reaction rate compared to linear aliphatic amines. Nevertheless, salt formation proceeded successfully under ambient conditions, yielding crystalline compounds in good yields.

**Scheme 6:**
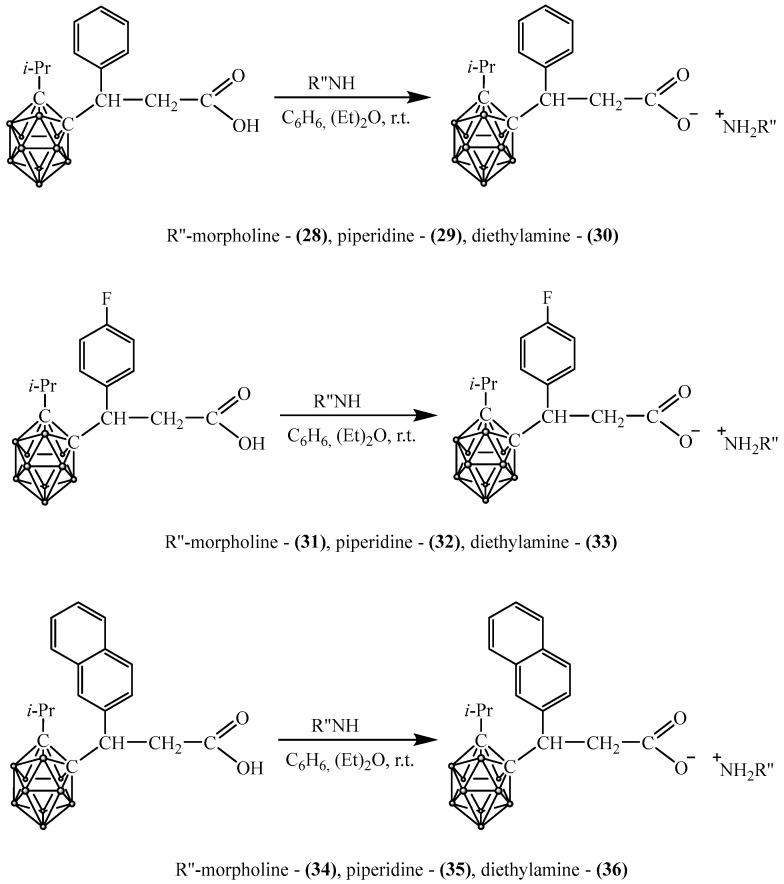
Synthesis of carboranyl-containing derivatives of 3-arylpropanoic acid with secondary amines.

The reactivity of secondary amines, namely morpholine, piperidine, and diethylamine, with carboranyl-containing 3-arylpropanoic acid was investigated to evaluate the influence of ring structure, steric bulk, and electronic character on ammonium carboxylate salt formation (Scheme 6). All three amines reacted under mild conditions (room temperature, equimolar ratios) with the carboranyl acid to afford the corresponding salts; however, yields and crystallization behavior varied significantly based on amine structure (Table 2).

Analyzing the ^1^H NMR spectra of salts obtained by synthesizing secondary amines with carboranyl containing 3-arylpropanoic acids, the presence of two hydrogen atoms in the range of ~2.9–3.2 ppm is observed with a possible shift to the left due to the redistribution of electron density. The -CH_2_- bond of the aliphatic acid chain is observed in this region; therefore, the alpha carbon atom does not participate in the reaction. The preservation of the C-H bond of the aliphatic chain, which is confirmed by the presence of a peak in the range of ~4–5 ppm, indicates that the beta carbon atom of the acid is also not involved in the reaction. There is also a peak in the range of ~6–7 ppm, in some cases ~10 ppm, that shows two hydrogen atoms, indicating the formation of an ammonium ion. The absence of proton signals in the ~10.35 or 12.30 ppm region in the ^1^H NMR spectra of the synthesized ammonium carboxylate salts confirms that the carboxylic acid moiety has undergone complete deprotonation.

The carbon NMR spectra of ^13^C, DEPT-135, DEPT-90, and APT-WALTZ of the obtained salts are characterized by the presence of a peak in the range of ~41–42 ppm, which refers to the -CH_2_- bond of the aliphatic acid chain and which is observed in the initial carboranyl-containing 3-arylpropanoic acid, as well as the preservation of the C-H bond peak in the range ~44–45 ppm. Similarly, to the salts that were obtained during the reaction with primary amines, the salts obtained during the synthesis of initial acids with secondary amines are characterized by the presence of three quaternary carbon atoms, one of which belongs to a carboxylate in the region of ~175 ppm, and two quaternary carbons in the region of ~88–90 ppm, which belong to the carborane core. The presence of the carborane core in the acid derivatives was confirmed by ^1^H and ^11^B NMR spectroscopy. The ^1^H NMR spectra exhibited signals corresponding to ten cage hydrogen atoms in the ~1.0–4.0 ppm range, while the ^11^B NMR spectra displayed resonances for ten boron atoms between approximately ~(−5) and (−12) ppm, consistent with the characteristic chemical environment of the carborane cluster.

**Figure 3:**
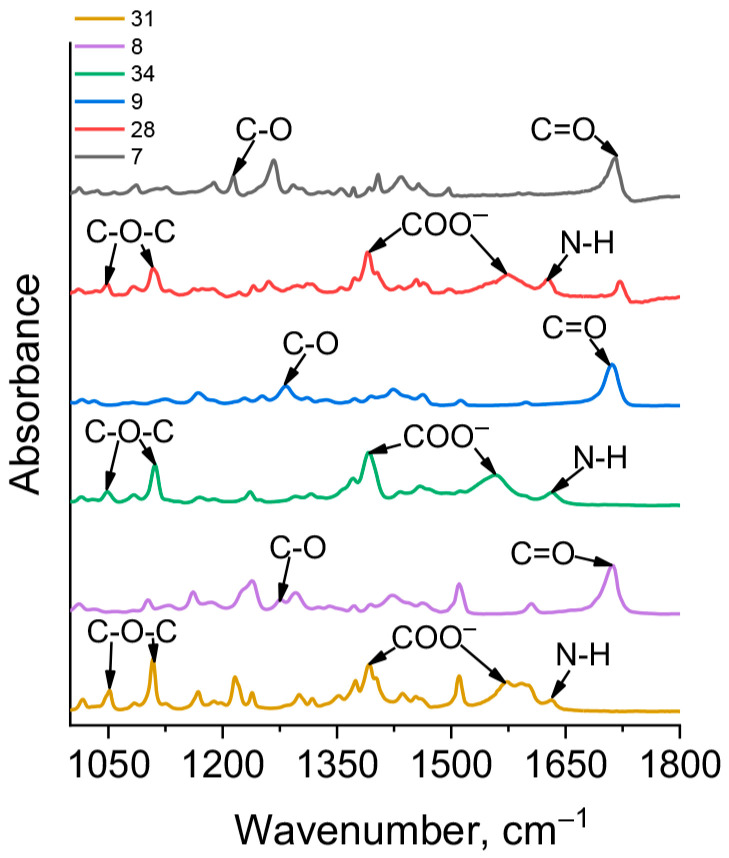
Comparison of FTIR spectra of carboranyl-containing 3-arylpropanoic acid (**7**, **8**, **9**) and obtained Morpholine 3-(2-isopropyl-1,2-dicarba-closo-dodecaboran-1-yl)-3-phenylpropanoate salt (**28**), Morpholine 3-(2-isopropyl-1,2-dicarba-closo-dodecaboran-1-yl)-3-(4-fluorophenyl)propanoate salt (**31**), Morpholine 3-(2-isopropyl-1,2-dicarba-closo-dodecaboran-1-yl)-3-(naphthalene-1-yl)propanoate salt (**34**).

The comparative analysis of FTIR spectra demonstrates structural changes in the compounds after synthesis and supports the presence of functional groups consistent with the proposed structures (Figure 3).

Morpholine contains an oxygen atom adjacent to the nitrogen within a six-membered ring, which exerts an electron-withdrawing inductive effect. This reduces the basicity of the nitrogen lone pair compared to other secondary amines such as piperidine. As a result, the proton transfer from the carboxylic acid to the morpholine nitrogen is less favorable kinetically, particularly at room temperature, leading to moderately slower salt formation under otherwise identical conditions. The polar ether functionality may enhance solubility in polar solvents, affecting crystallization yield but not reaction completeness.

Piperidine demonstrated exceptional reactivity among the three amines examined. Its saturated, non-polar ring structure provides a strong basicity and minimal spatial limitations, facilitating the rapid and extensive formation of ammonium carboxylate salts. The resulting product was easily isolated as a solid crystal with a high yield. The favorable spatial characteristics of piperidine and its affinity for protons contributed to the efficient formation of hydrogen bonds and compact salt packing, even in the presence of a bulky isopropyl-*o*-carborane substituent.

In contrast, diethylamine showed lower yields and less favorable crystallization. As a cyclic secondary amine, diethylamine has a more complex spatial structure and is more volatile than its cyclic counterparts. This is due to the presence of two bulky ethyl groups next to the nitrogen atom, which creates significant spatial obstacles around the single proton responsible for proton capture. These spatial constraints prevent proximity between the carboxylic acid and diethylamine, especially in the presence of an isopropyl-*o*-carborane group in the β-position of the acid. As a result, the proton transfer process is less efficient, and the reaction occurs more slowly compared to using smaller or cyclic secondary amines.

### 2.5. Synthesis of Carboranyl-Containing Carboxylate Salts Using Metals and Their Hydroxides

Carboxylate salts represent a diverse and widely studied class of compounds with applications across organic, inorganic, and materials chemistry. These salts are formed when carboxylic acids react with bases or metal sources to yield the corresponding carboxylate anion (R-COO^−^) paired with a cation (metal or organic). Due to their structural versatility, carboxylate salts play important roles in various fields of activity. One of the most straightforward and widely employed methods for preparing carboxylate salts is the neutralization reaction, in which a carboxylic acid reacts with a metal hydroxide, metal oxide, or basic salt. This acid–base reaction results in the formation of a metal carboxylate and water as the only by-product. The simplicity and efficiency of the neutralization approach make it particularly attractive for both laboratory and industrial synthesis. It is compatible with a wide variety of carboxylic acids—including aromatic, aliphatic, unsaturated, and functionalized derivatives—as well as with alkali, alkaline earth, and transition metal hydroxides.

The resulting carboxylate salts often display distinct coordination geometries depending on the metal center and the nature of the acid, influencing their solubility, crystallinity, and thermal stability. Spectroscopic techniques such as FTIR and NMR, as well as crystallographic and thermal analyses, are commonly used to confirm salt formation and characterize structural features. Several salts of 3-phenylpropanoic acid have been previously investigated, with studies focusing on their structural characteristics, crystal packing, chemical and antioxidant properties, as well as their solubility profiles [77,78].

**Scheme 7:**
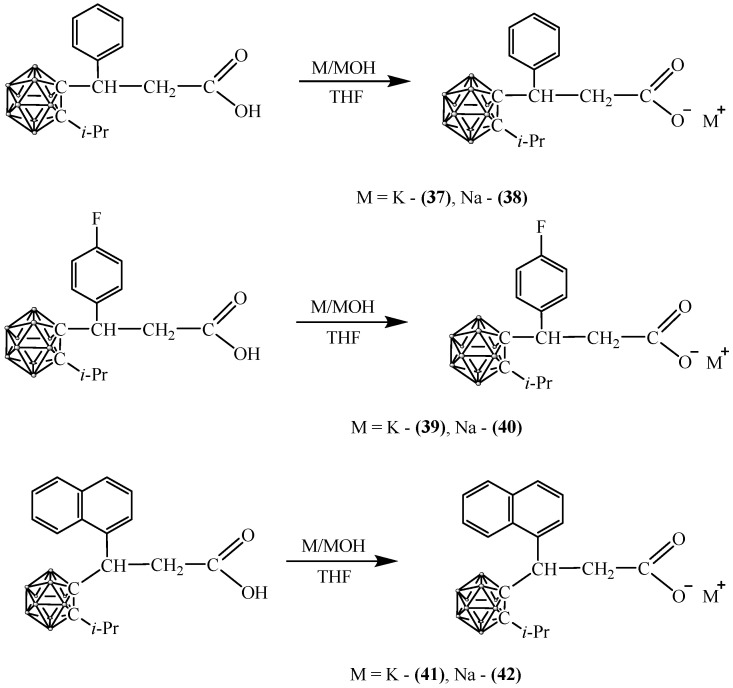
Synthesis of carboranyl-containing derivatives of 3-arylpropanoic acid with metals and their hydroxides.

**Figure 4:**
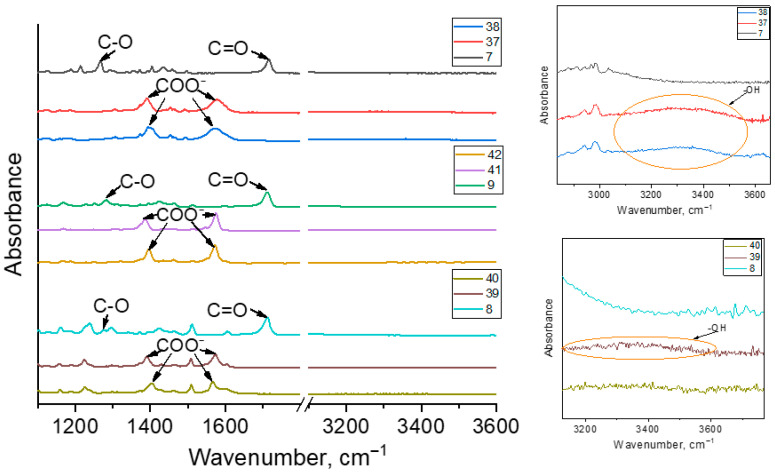
Comparison of FTIR spectra of carboranyl-containing 3-arylpropanoic acid (**7**, **8**, **9**) and obtained potassium and sodium salts (**37**, **38**, **39**, **40**, **41**, **42**).

This study focuses on the synthesis of sodium and potassium carboxylate salts via neutralization reactions, exploring the influence of the type of metal ion, acid structure, and reaction conditions on product yield (Scheme 7). It also investigates the physico-chemical properties, as well as the structure of the resulting salts using FTIR and NMR spectroscopies, and elemental analysis. The comparative analysis of FTIR spectra demonstrates structural changes in the compounds after synthesis and supports the presence of functional groups consistent with the proposed structures (Figure 4).

Solubility tests demonstrated that the potassium salt is more soluble in water than its sodium counterpart, consistent with the general trend of higher hydration energy and larger lattice spacing for K^+^ salts. The potassium salts of 3-(2-isopropyl-1,2-dicarba-closo-dodecaboran-1-yl)-3-phenylpropanoic acid (**37**) and 3-(2-isopropyl-1,2-dicarba-closo-dodecaboran-1-yl)-3-(4-fluorophenyl)propanoic acid (**39**) exhibit good water solubility, making them promising candidates for boron-based therapeutic agents for BNCT. Their high aqueous solubility contributes to improved bioavailability, facilitates administration, supports more predictable pharmacokinetic behavior, and may help reduce systemic toxicity. Both salts are readily soluble in polar solvents, such as methanol and ethanol, but are only sparingly soluble in less polar solvents, like ethyl acetate or acetone.

The ^1^H NMR spectra of the obtained carboxylate salts indicate the absence of acid proton signals in the range of ~10.35 or 12.30 ppm, which confirms the complete deprotonation of carboxylic acid. The peaks of the bonds of -CH_2_- and C-H of the aliphatic chain of 3-arylpropanoic acid remain in the regions of ~2.5 ppm for -CH_2_- and ~4.0–5.0 ppm for C-H, respectively. There is also an extensive peak in the range of ~1.0–4.0 ppm, which belongs to the protons of the carborane core. The NMR spectra of carbon ^13^C also indicate the presence of only 3 quaternary carbon atoms in the regions of ~88 ppm and ~90 ppm, which belong to the quaternary carbon atoms of the carborane core, and a peak in the region of ~175 ppm, related to the quaternary carbon carboxylate ion. The NMR spectra of ^11^B show peaks in the region of ~(−6)–(−12) ppm, which confirms the presence of 10 boron atoms of the isopropyl-o-carborane substituent.

To summarize, sodium and potassium salts of arylaliphatic acids containing a carborane moiety were successfully synthesized under mild neutralization conditions, yielding stable and water-soluble products. Spectroscopic data confirmed the complete deprotonation of the carboxylic acid and the preservation of the carborane structure. The excellent water solubility, particularly of the potassium salts, highlights their potential for biomedical applications where solubility and biocompatibility are of critical importance.

### 2.6. In Vitro Cytotoxicity Testing

**Figure 5:**
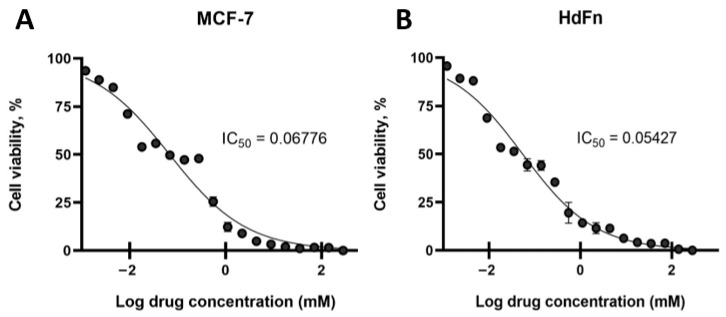
Viability of human cells MCF-7 (**A**) and HDFn (**B**) after treatment with various concentrations of compound 37.

Cytotoxicity studies represent a crucial first step in evaluating the biomedical applicability of newly synthesized compounds, including their potential use in boron neutron capture therapy (BNCT). Primary cytotoxicity studies were performed for the water-soluble compound potassium 3-(2-isopropyl-1,2-dicarba-closo-dodecaboran-1-yl)-3-phenylpropanoate (compound 37). Prior to cytotoxicity studies, the purity of the compound 37 was quantified by HPLC peak area, and the purity was 95.6%. The results of the cytotoxicity assays showed that compound 37 exhibited a significant toxic effect on MCF-7 cancer cells at concentrations ranging from 281.69 mM to 2.201 mM (Figure 5A). At a concentration of 0.06776 mM and below, the cytotoxicity of this compound did not exceed the IC_50_ value.

Regarding human dermal fibroblasts (HDFn), compound 37 caused considerable cell death at concentrations from 281.69 mM to 0.069 mM. At a concentration of 0.05427 mM and lower, the cytotoxicity of compound 37 did not exceed the IC_50_ threshold (Figure 5B).

Thus, compound 37 is toxic at concentrations up to 0.069 mM toward human MCF-7 and HDFn cells. It does not induce a significant toxic effect on the viability of MCF-7 and HDFn cells at concentrations of 0.06776 mM and 0.05427 mM, respectively.

These data show that compound 37 exhibits comparable cytotoxicity toward non-malignant HDFn fibroblasts (IC_50_ = 0.054 mM) and malignant MCF-7 breast cancer cells (IC_50_ = 0.067 mM). Therefore, it possesses no inherent cytotoxic selectivity. For BNCT, however, intrinsic anticancer activity is not mandatory, because the therapeutic effect arises from the high-LET α-particles and ^7^Li nuclei generated when thermal neutrons are captured by ^10^B. The key requirements for potential BNCT agents are selective delivery to tumor tissue and efficient accumulation of boron within cancer cells (~10^9 10^B atoms per cell). To enable effective delivery of the carborane compound, we are investigating nanoscale delivery systems such as iron oxide-based nanocarriers, as described in [79] that facilitate intracellular boron accumulation at concentrations up to 1–6 × 10^10^ atoms per cell, thereby meeting the established threshold for BNCT efficacy. Currently, studies are underway to conjugate the water-soluble compound 37 to a nanocarrier in order to achieve selective accumulation in cancer cells. Primary cytotoxicity studies indicate that the synthesized compounds do not exhibit high intrinsic toxicity, which supports the feasibility of conducting further investigations focused on tumor selectivity, intracellular accumulation, and nanocarrier-based delivery systems.

## 3. Materials and Methods

### 3.1. Materials and Chemicals

Benzaldehyde, 1-naphthaldehyde, 4-fluorobenzaldehyde, diethyl malonate, butylamine, tert-butylamine, allylamine, morpholine, methylamine (40% in H_2_O), ethylenediamine, diethylamine, cyclohexylamine, and butyllithium (n-BuLi) in hexane, tetrahydrofuran (THF), and isopropyl alcohol (IPA) were supplied by Sigma Aldrich (St. Louis, MO, USA). All other chemicals and solvents, such as piperidine, isopropyl-o-carborane, hydrochloric acid, acetic acid, hydrobromic acid, ethyl acetate, diethyl ether, metallic potassium, metallic sodium, potassium hydroxide, sodium hydroxide, sodium sulfate had purity of analytical grade. The hexane was purified by drying over calcium chloride overnight, followed by distillation. Purification of benzene was achieved by overnight treatment with sodium metal and subsequent distillation.

Thin layer chromatography (TLC) was performed using 20 × 20 cm aluminum oxide plates. The spots were developed with iodine. Various ratios of ethyl acetate and hexane were used as eluents.

### 3.2. Methods of Characterization

FTIR spectra were recorded on an InfraLum FT-08 FTIR Spectrometer (Lumex, St. Petersburg, Russia) with a Single Reflection Diamond ATR accessory (GladiATR, PIKE, Memphis, TN, USA) to study chemical group shifts before and after synthesis. Measurements were taken in the range of 400 to 4000 cm^−1^. The spectra obtained were processed in the SpectraLUM^®^ (version 2.0.1.295). All spectra (25 scans at 2 cm^−1^ resolution) were recorded at 21–25 °C.

The melting point of the obtained substances was measured using the Buchi Melting Point M-560 device (BÜCHI Labortechnik AG, Flawil, Switzerland).

NMR spectra were obtained with JEOL ECA-500 MHz (JEOL, Tokyo, Japan) spectrometer (^1^H: 500 MHz, ^13^C: 126 MHz, ^11^B: 160 MHz) and with NMR spectrometer Spinsolve 80^ULTRA^ (Magritek, Aachen, Germany and Wellington, New Zealand) (^1^H: 80 MHz, ^13^C: 20 MHz, ^11^B: 26 MHz, ^19^F: 76 MHz) benchtop NMR. All measurements were taken at a temperature of 20–25 °C. All boron, carbon, and fluorine NMR spectra were recorded using proton-decoupled acquisition. The spectra obtained were processed in the MestReNova (version 14.2.1-27684) and Spinsolve (version 2.6.0).

Elemental analysis was conducted using an Elementar Unicube analyzer (Elementar Analysensysteme GmbH, Langenselbold, Germany). Each sample was measured in triplicate, and the average values for carbon (C), hydrogen (H), and nitrogen (N) content were calculated. Sulfonamide was employed as the calibration standard.

HPLC was performed on ECS01 Gradient Analytical System (Prague, Czech Republic) using Astra C18-HE HPLC column (Prague, Czech Republic) with ECD2800 Analytical UV-VIS Diode Array Detector (Prague, Czech Republic).

The obtained substances were also investigated using the LC-MS Q-TOF Liquid Chromatography system (UHPLC ELUTE (Bruker, Bremen, Germany) with a high-resolution mass spectrometric detector impact II VIP (Bruker, Bremen, Germany)).

### 3.3. Synthesis of Diethyl Arylidenemalonates: General Procedure

In a 100 mL round-bottom flask equipped with a Dean-Stark trap, diethyl malonate and corresponding arylaldehyde (1 equiv.), 1 mL (10 mmol) of piperidine as catalyst, and 40 mL of benzene were added. The mixture was vigorously boiled for 11–18 h until the calculated amount of water was released. After the synthesis was complete as confirmed by TLC, the reaction mixture was treated with hydrochloric acid and extracted with ethyl acetate. The ethyl acetate extract was dried over sodium sulfate and then filtered to remove the desiccant. The ethyl acetate was then distilled off using a rotary evaporator to obtain final product. The product was either distilled (when liquid) or recrystallized from IPA (when solid) to obtain the purified diethyl arylidenemalonates.

Diethyl 2-benzylidenemalonate (**1**). From diethyl malonate 6.8 mL (44.8 mmol) and benzaldehyde. Yield—7.83 mL (78%); colorless oil; bp 215–217 °C/30 mmHg.

FTIR spectrum, ν, cm^−1^: 1721 (C=O), 1629 (C=C), 2850–3000 (C-H), 3030–3050 (C-H, ar.), 1254, 1058 (C-O-C), 1448 (C=C, ring modes), 691 (out-of-plane, C-H), 1209, 1080 (in-plane, C-H).

^1^H NMR (80 MHz, without solvent): δ = 0.75 (m, 6H, -CH_3_, -CH_3_), 3.80 (m, *J* = 6.9 Hz, 4H, -CH_2_-, -CH_2_-), 6.91 (td, *J* = 6.9 Hz, 5H, C_sp2_H), 7.30 (s, 1H, -CH=).

^13^C NMR (20 MHz, without solvent): δ = 13.21 (-CH_3_), 13.42 (-CH_3_), 60.89 (-CH_2_-, -CH_2_-), 126.40 (C_sp2_H), 128.39 (C_sp2_H), 129.03 (C_sp2_H), 130.09 (C_sp2_H), 132.62 (C_sp2_H), 138.60 (C_4_), 140.90 (-CH=), 163.27 (C4), 165.68 (C_4_), 166.35 (C_4_).

Diethyl 2-(4-fluorobenzylidene)malonate (**2**). From diethyl malonate 6.8 mL (44.8 mmol) and 4-fluorobenzaldehyde. Yield—6.50 mL (60%); oily yellow liquid; bp 210 °C (75 mbar).

FTIR spectrum, ν, cm^−1^: 1721 (C=O), 1632 (C=C), 2902, 2940, 2982 (C-H), 3078 (C-H, ar.), 1257, 1062 (C-O-C), 1509 (C=C, ring modes), 834 (out-of-plane, C-H), 1020 (in-plane, C-H).

^1^H NMR (80 MHz, CDCl_3_): δ = 1.08 (m, *J* = 7.1, 2.2 Hz, 6H, -CH_3_, -CH_3_), 4.10 (m, *J* = 7.1, 4.1 Hz, 4H, -CH_2_-, -CH_2_-), 6.84 (m, *J* = 8.7 Hz, 2H, C_sp2_H), 7.24 (m, 2H, C_sp2_H), 7.30 (s, 1H, -CH=).

^13^C NMR (20 MHz, CDCl_3_): δ = 13.67 (-CH_3_), 13.88 (-CH_3_), 61.42 (-CH_2_-), 61.50 (-CH_2_-), 115.27 (C_sp2_H), 116.35 (C_sp2_H), 126.04 (C_4_), 129.00 (C_4_), 131.30 (C_sp2_H), 131.72 (C_sp2_H), 140.41 (-CH=), 163.76 (C_4_), 166.28 (C_4_), 169.94 (C_4_).

^19^F NMR (76 MHz, CDCl_3_): δ = −106.97 (s, 1F).

Diethyl 2-(naphthalen-1-yl)malonate (**3**). From diethyl malonate 6.8 mL (44.8 mmol) and 1-naphthaldehyde. Yield—11.88 mL (78%); oily yellow liquid; bp 228 °C (10 mbar).

FTIR spectrum, ν, cm^−1^: 1722 (C=O), 1628 (C=C), 2937, 2982 (C-H), 3030–3060 (C-H, ar.), 1206, 1274 (C-O-C), 1508 (C=C, ring modes), 735, 775, 798 (out-of-plane, C-H), 1019 (in-plane, C-H).

^1^H NMR (500 MHz, CDCl_3_): δ = 1.05 (t, *J* = 7.1 Hz, 3H, -CH_3_), 1.37 (t, *J* = 7.1 Hz, 3H, -CH_3_), 4.16 (q, *J* = 7.1 Hz, 2H, -CH_2_-), 4.37 (q, *J* = 7.2 Hz, 2H, -CH_2_-), 7.42 (t, *J* = 7.7 Hz, 1H, C_sp2_H), 7.54 (m, 3H, C_sp2_H), 7.86 (m, 2H, C_sp2_H), 8.00 (d, *J* = 8.1 Hz, 1H, C_sp2_H), 8.48 (s, 1H, -CH=).

^13^C NMR (126 MHz, CDCl_3_): δ = 13.87 (-CH_3_), 14.30 (-CH_3_), 61.56 (-CH_2_-), 61.86 (-CH_2_-), 124.13 (C_sp2_H), 125.27 (C_sp2_H), 126.51 (C_sp2_H), 126.49(C_sp2_H), 127.02(C_sp2_H), 128.77 (C_sp2_H), 129.35 (C_sp2_H), 130.57 (C_sp2_H), 130.91 (C_sp2_H), 131.42 (C_sp2_H), 133.45 (-CH=), 141.32 (C_4_), 164.03 (C_4_), 166.22 (C_4_).

### 3.4. Synthesis of Diethyl Arylidenemalonates with Isopropyl-o-carborane: General Procedure

In a 250 mL three-necked flask equipped with a stir bar and located under an argon atmosphere, 7.66 mL (44 mmol) isopropyl-*o*-carborane, 20 mL hexane, and 19.36 mL (2.5 N, 44 mmol +10%) n-BuLi solution in hexane were added. The mixture was allowed to stir for approximately 30 min, until a white precipitate formed. The precipitate was then dissolved by adding 30 mL of THF. The flask with the reaction mixture was cooled to −50 °C using a cryostat. Then 44 mmol (1 equiv.) of corresponding diethyl arylidenemalonate was added, and the flask was allowed to continue stirring for approximately 5–6 h, while increasing the temperature by 10 °C every hour. When the temperature reached 0 °C, the mixture was stirred for an additional hour. After that the cryostat was turned off, and the reaction was allowed to complete at room temperature. The reaction was monitored using TLC to determine when it was completed. After the reaction has finished, the mixture is treated with hydrochloric acid until it reaches a neutral pH. It is then extracted with ethyl acetate and allowed to dry for a day under sodium sulfate. Next, the substance is filtered through a funnel to remove any remaining sodium sulfate, and the solvent is evaporated using a rotary unit to obtain a resin-like product. This product is then crystallized over time with hexane at −10 °C and recrystallized in IPA to obtain white crystals.

Diethyl 2-[4-(1-isopropyl-1,2-dicarba-*closo*-dodecaboran-1-yl)benzyl]propanedioate (**4**). From diethyl 2-benzylidenemalonate 9.86 mL (44 mmol) and isopropyl-*o*-carborane. Yield—8.56 g (45%); white crystals; mp 73.8–74.2 °C.

FTIR spectrum, ν, cm^−1^: 1752 (C=O, ester), 2564, 2625 (B-H), 1456 (C=C, ring modes), 702 (out-of-plane, C-H), 1202, 1026 (in-plane, C-H), 2875–2986 (C-H), 3065 (C-H, ar.), 1246, 1141 (C-O-C).

^1^H NMR (80 MHz, DMSO-*d*_6_): δ = 0.5–4.0 (m, 10H, B-H), 0.73 (m, *J* = 7.1 Hz, 6H, -CH_3_, -CH_3_), 1.18 (m, 6H, CH(CH_3_)_2_), 2.88 (m, *J* = 6.6 Hz, 1H, CH(CH_3_)_2_), 3.55 (m, J = 7.1 Hz, 2H, (C-H)_2_), 4.18 (m, 4H, (-CH_2_-)_2_), 7.30 (m, 5H, C_sp2_H).

^13^C NMR (20 MHz, DMSO-*d*_6_): δ = 13.56 (-CH_3_), 14.06 (-CH_3_), 23.92 (-CH_3_), 25.06 (-CH_3_), 30.14 (CH(CH_3_)_2_), 46.89 (C-H), 59.88 (C-H), 61.91 (-CH_2_-), 62.67 (-CH_2_-), 87.65 (C_4_), 91.13 (C_4_), 128.46 (C_sp2_H, 2 atoms), 129.00 (C_sp2_H, 3 atoms), 137.49 (C_4_), 165.69 (C=O), 167.14 (C=O).

^11^B NMR (26 MHz, DMSO-*d*_6_): δ = −7.5 (10B).

Elemental analysis: calculated: for C_19_H_34_B_10_O_4_ (%): C 52.52, H 7.89; found (%): C 52.17, H 7. 821.

Diethyl 2-[4-fluoro-4-(1-isopropyl-1,2-dicarba-*closo*-dodecaboran-1-yl)benzyl]propanedioate (**5**). From diethyl 2-(4-fluorobenzylidene)malonate 10.6 mL (44 mmol) and isopropyl-*o*-carborane. Yield—12. 73 g (66%); white crystals; mp 101.2–104.2 °C.

FTIR spectrum, ν, cm^−1^: 1752 (C=O, ester), 1510 (C=C, ring modes), 1142, 1203 (C-O-C), 850 (out-of-plane, C-H), 1012 (in-plane, C-H), 2903, 2941, 2986 (C-H), 3083 (C-H, ar.), 2563, 2600 (B-H).

^1^H NMR (80 MHz, CDCl_3_): δ = 1.0–4.5 (m, 10H, B-H), 0.98 (m, 6H, -CH_3_, -CH_3_), 1.36 (m, 6H, CH(CH_3_)_2_), 2.83 (m, 1H, CH(CH_3_)_2_), 3.74 (q, *J* = 7.1 Hz, 2H, (C-H)_2_), 4.32 (m, 4H, (-CH_2_-)_2_), 7.17 (m, 4H, C_sp2_H).

^13^C NMR (20 MHz, CDCl_3_): δ = 13.60 (-CH_3_), 13.95 (-CH_3_), 24.07 (-CH_3_), 24.92 (-CH_3_), 30.43 (CH(CH_3_)_2_), 45.57 (C-H), 60.00 (C-H), 62.05 (-CH_2_-), 62.78 (-CH_2_-), 86.26 (C_4_), 90.11 (C_4_), 114.67 (C_sp2_H), 115.75 (C_sp2_H), 132.02 (C_sp2_H), 132.58 (C_sp2_H), 133.34 (C_4_), 133.52 (C_4_), 166.09 (C=O), 167.36 (C=O).

^19^F NMR (76 MHz, CDCl_3_): δ = −110.11 (s, 1F).

^11^B NMR (26 MHz, CDCl_3_): δ = −3.8 (5B), −9.92 (5B).

Elemental analysis: calculated: for C_19_H_33_B_10_O_4_F (%): C 50.43, H 7.35; F = 4.20; found (%): C 50.21, H = 7.050.

Diethyl 2-[(1-isopropyl-1,2-dicarba-*closo*-dodecaboran-1-yl)naphthalen-1-yl]propanedioate (**6**). From diethyl 2-(naphthalen-1-yl)malonate 14.96 mL (44 mmol) and isopropyl-*o*-carborane. Yield—13.8 g (65%); white crystals; mp 126.2–129.3 °C.

FTIR spectrum, ν, cm^−1^: 1730 (C=O, ester), 2568, 2602, 2636 (B-H), 2938, 2982 (C-H), 3065 (C-H, ar.), 1208, 1298 (C-O-C), 1508 (C=C, ring modes), 736, 782, 795 (out-of-plane, C-H), 1016 (in-plane, C-H).

^1^H NMR (500 MHz, CDCl_3_): δ = 1.6–2.7 (m, 10H, B-H), 0.34 (t, *J* = 7.1 Hz, 2H, -CH_2_-), 0.55 (d, *J* = 6.8 Hz, 3H, -CH_3_), 1.22 (d, *J* = 6.8 Hz, 3H, -CH_3_), 1.37 (t, *J* = 7.1 Hz, 2H, -CH_2_-), 1.55 (s, 6H, CH(CH_3_)_2_), 2.76 (m, *J* = 6.8 Hz, 1H, CH(CH_3_)_2_), 3.21 (m, *J* = 10.7, 7.2 Hz, 1H, C-H), 3.36 (m, *J* = 10.8, 7.1 Hz, 1H, C-H), 7.47 (t, *J* = 7.7 Hz, 2H, C_sp2_H), 7.55 (t, *J* = 8.6, 6.6, 1.4 Hz, 1H, C_sp2_H), 7.67 (d, *J* = 7.3 Hz, 1H, C_sp2_H), 7.82 (t, 2H, C_sp2_H), 8.14 (d, *J* = 8.7 Hz, 1H, C_sp2_H).

^13^C NMR (126 MHz, CDCl_3_): δ = 12.93 (-CH_3_), 14.00 (-CH_3_), 23.31 (-CH_3_), 24.95 (-CH_3_), 30.53 (CH(CH_3_)_2_), 40.09 (C-H), 61.20 (C-H), 61.69 (-CH_2_-), 62.82 (-CH_2_-), 122.70 (C_sp2_H), 124.56 (C_sp2_H), 125.83 (C_sp2_H), 126.71 (C_sp2_H), 129.31 (C_sp2_H), 129.51 (C_sp2_H), 130.12 (C_sp2_H), 131.71 (C_sp2_H), 133.24 (C_sp2_H), 133.66 (C_sp2_H), 141.33 (C_4_), 142.27 (C_4_), 165.73 (C=O), 168.08 (C=O).

^11^B NMR (160 MHz, CDCl_3_): δ = −4.70 (7B), −11.26 (3B).

Elemental analysis: calculated: for C_23_H_36_B_10_O_4_ (%): C 56.99, H 7.49; found (%): C 56.50, H 7.679.

### 3.5. Synthesis of Isopropyl-o-carboranyl Containing Derivatives of 3-Arylpropanoic Acid: General Procedure

In a 250 mL round-bottom flask equipped with a stirrer and a reflux condenser (10 mmol) of the corresponding isopropyl-*o*-carboranyl containing diethyl arylidenemalonate, 50 mL (874 mmol) of acetic acid, and 20 mL (368 mmol) of hydrobromic acid were added. The mixture was vigorously stirred and heated for 24 h, during which time the color of the solution changed from light yellow to light brown. The process of the reaction was controlled by TLC. At the end of the reaction, white, dirty crystals precipitated out. These crystals were filtered using a Schott glass filter and thoroughly washed with deionized water. The resulting solid was dried on a feather cup and recrystallized to obtain a pure product. If the product is soluble in acids, the mixture is extracted using a mixture of diethyl ether and deionized water. The ethyl acetate extract was dried over sodium sulfate and then filtered to remove the desiccant. The ethyl acetate was then distilled off using a rotary evaporator. The resulting product is recrystallized to purify.

3-(2-isopropyl-1,2-dicarba-*closo*-dodecaboran-1-yl)-3-phenylpropanoic acid (**7**). From diethyl 2-[4-(1-isopropyl-1,2-dicarba-closo-dodecaboran-1-yl)benzyl]propanedioate 4.32 g (10 mmol). Yield—1.89 g (60%); white crystals; mp 159–160.6 °C; recrystallized using benzene/hexane.

FTIR spectrum, ν, cm^−1^: 2700–3200 (O-H), 3030, 3063 (C-H, ar.), 2873–2981 (C-H), 2558, 2612 (B-H), 1715 (C=O, acid), 1457 (C=C, ring modes), 1214, 1267 (C-O), 1086, 1213 (in-plane, C-H), 698 (out-of-plane, C-H).

^1^H NMR (80 MHz, DMSO-*d*_6_): δ = 1.4–2.6 (m, 10H, B-H), 1.19 (d, *J* = 6.3 Hz, 6H, CH(CH_3_)_2_), 2.46 (q, *J* = 1.9 Hz, 1H, CH(CH_3_)_2_), 2.97 (d, *J* = 7.7 Hz, 2H, -CH_2_-), 3.86 (t, *J* = 7.7 Hz, 1H, C-H), 7.29 (s, 5H, C_sp2_H), 12.30 (s, 1H, -COOH).

^13^C NMR (20 MHz, DMSO-*d*_6_): δ = 24.58 (-CH_3_), 24.87 (-CH_3_), 30.78 (C-H), 41.50 (-CH_2_), 43.66 (C-H), 87.73 (C_4_, carborane core), 90.70 (C_4_, carborane core), 128.51 (C_sp2_H), 128.73 (C_sp2_H), 129.66 (C_sp2_H, 3 atoms), 139. 49 (C_4_, phenyl), 171.52 (C_4_, -COOH).

^11^B NMR (26 MHz, DMSO-*d*_6_): −4.6 (5B), −9.3 (5B).

LC-MS for C_14_H_26_B_10_O_2_ [M + H]^+^: 335.2898.

Elemental analysis: calculated: for C_14_H_26_B_10_O_2_ (%): C 50.27, H 7.83; found (%): C 50.13, H 7.468.

3-(2-isopropyl-1,2-dicarba-*closo*-dodecaboran-1-yl)-3-(4-fluorophenyl)propanoic acid (**8**). From diethyl 2-[4-fluoro-4-(1-isopropyl-1,2-dicarba-closo-dodecaboran-1-yl)benzyl]propanedioate 4.38 g (10 mmol). Yield—2.7 g (92%); white crystals; mp 150–153 °C; recrystallized using benzene.

FTIR spectrum, ν, cm^−1^: 2700–3200 (O-H), 3054 (C-H, ar.), 2938, 2982 (C-H), 2575, 2622 (B-H), 1711 (C=O, acid), 1510 (C=C, ring modes), 1275 (C-O), 1010 (in-plane, C-H), 837 (out-of-plane, C-H).

^1^H NMR (80 MHz, CDCl_3_): δ = 0.5–4.0 (m, 10H, B-H), 1.24 (dd, J = 6.8, 2.1 Hz, 6H, CH(CH_3_)_2_), 2.52 (m, *J* = 6.8 Hz, 1H, CH(CH_3_)_2_), 2.98 (d, *J* = 7.7 Hz, 2H, -CH_2_-), 3.80 (t, *J* = 7.8 Hz, 1H, C-H), 7.07 (m, J = 7.3, 5.4 Hz, 4H, C_sp2_H), 10.35 (s, 1H, -COOH).

^13^C NMR (20 MHz, CDCl_3_): δ = 24.55 (-CH_3_), 24.80 (-CH_3_), 31.22 (CH(CH_3_)_2_), 41.73 (-CH_2_-), 42.91 (C-H), 85.78 (C_4_, carborane core), 89.81 (C_4_, carborane core), 115.26 (C_sp2_H), 116.33 (C_sp2_H), 128.49 (benzene), 130.70 (C_sp2_H), 131.11 (C_sp2_H), 134.10 (C_4_, benzylidene), 134.28 (C_4_, benzylidene), 176.30 (C_4_, -COOH).

^11^B NMR (26 MHz, CDCl_3_): δ = −4.9 (5B), −9.09 (5B).

^19^F NMR (76 MHz, CDCl_3_): δ = −110.44 (s, 1F).

LC-MS for C_14_H_25_B_10_O_2_F [M + H]^+^: 353.2802.

Elemental analysis: calculated: for C_14_H_25_B_10_O_2_F (%): C 47.72, H 7.15; found (%): C 47.35, H 7. 082.

3-(2-isopropyl-1,2-dicarba-*closo*-dodecaboran-1-yl)-3-(naphthalene-1-yl)propanoic acid (**9**). From diethyl 2-[(1-isopropyl-1,2-dicarba-closo-dodecaboran-1-yl)naphthalen-1-yl]propanedioate 4.82 g (10 mmol). Yield—2 g (55%); white crystals; mp 190.1–192.8 °C; recrystallized using cyclohexane.

FTIR spectrum, ν, cm^−1^: 2700–3200 (O-H), 3037 (C-H, ar.), 2936, 2986 (C-H), 2561, 2594, 2618 (B-H), 1710 (C=O, acid), 1463 (C=C, ring modes), 1282 (C-O), 1014 (in-plane, C-H), 737, 771, 791 (out-of-plane, C-H).

^1^H NMR (500 MHz, CDCl_3_): δ = 1.0–4.0 (m, 10H, B-H), 1.32 (d, *J* = 6.6 Hz, 6H, CH(CH_3_)_2_), 2.44 (p, *J =* 1.9 Hz, 1H, CH(CH_3_)_2_), 3.22 (d, *J* = 7.6 Hz, 2H, -CH_2_-), 4.98 (t, *J* = 7.8 Hz, 1H, C-H), 8.33–7.31 (m, 7H, C_sp2_H), 12.33 (s, 1H, -COOH).

^13^C NMR (20 MHz, CDCl_3_): δ = 24.74 (-CH_3_), 25.03 (CH_3_), 30.74 (CH(CH_3_)_2_), 37.21 (C-H), 42.84 (-CH_2_-), 88.84 (C4, carborane core), 90.05 (C4, carborane core), 123.41 (C_sp2_H), 125.75 (C_sp2_H), 126.30 (C_sp2_H), 127.01 (C_sp2_H), 127.28 (C_sp2_H), 129.23 (C_sp2_H), 129.41 (C_sp2_H), 131.76 (C_4_, naphthyl), 133.93 (C_4_, naphthyl), 136.28 (C_4_, naphthyl), 171.47 (C_4_, -COOH).

^11^B NMR (26 MHz, CDCl_3_): δ = −9.13 (10B).

LC-MS for C_18_H_28_B_10_O_2_ [M + H]^+^: 385.3044.

Elemental analysis: calculated: for C_18_H_28_B_10_O_2_ (%): C 56.22, H 7.34; found (%): C 57.48, H 7.660.

### 3.6. Synthesis of Isopropyl-o-carboranyl Containing 3-Arylpropanoic Acid with Amines: General Procedure

In a 50 mL round-bottomed flask equipped with a stir bar, an equimolar/mmol + 10% (amine) amount of isopropyl-*o*-carboranyl containing 3-arylpropanoic acid and amine was added to a 1:2 mixture of diethyl ether and benzene. The mixture was stirred for approximately 10 min. The precipitate was filtered using a Schott glass filter, washed with diethyl ether, and then recrystallized using benzene.

#### 3.6.1. Synthesis of Isopropyl-o-carboranyl Containing 3-Arylpropanoic Acid with Primary Amines

Butylammonium 3-(2-isopropyl-1,2-dicarba-*closo*-dodecaboran-1-yl)-3-phenylpropanoate (**10**). From 3-(2-isopropyl-1,2-dicarba-closo-dodecaboran-1-yl)-3-phenylpropanoic acid 0.09 g (0.27 mmol) and butylamine 0.028 mL (0.27 mmol). Yield—0.09 g (90%); white crystals; mp 185–189 °C.

FTIR spectrum, ν, cm^−1^: 3000–3200 (NH_3_^+^), 3033 (C-H, ar.), 2870, 2958, 2978 (C-H), 2556, 2581, 2612 (B-H), 1621 (N-H), 1540 (COO^−^, ν_asym), 1496 (C=C, ring modes), 1382 (COO^−^, ν_sym), 1012 (in-plane, C-H), 702 (out-of-plane, C-H).

^1^H NMR (80 MHz, DMSO-*d*_6_): δ = 1.0–4.0 (m, 10H, B-H), 0.89 (d, *J* = 5.3 Hz, 3H, -CH_3_), 1.26 (d, *J* = 6.8 Hz, 8 H, CH(CH_3_)_2_, -CH_2_-), 2.56–2.48 (m, 4H, (-CH_2_-)_2_), 2.67 (d, *J* = 7.1 Hz, 2H, -CH_2_-), 2.91 (t, *J* = 6.8 Hz, 1H, CH(CH_3_)_2_), 4.05 (t, *J* = 7.2 Hz, 1H, C-H), 6.87 (s, 3H, NH_3_^+^), 7.30 (s, 4H, C_sp2_H), 7.40 (s, 1H, C_sp2_H).

^13^C NMR (20 MHz, DMSO-*d*_6_): δ = 14.01 (-CH_3_), 19.69 (-CH_2_-), 24.62 (-CH_3_), 24.86 (-CH_3_), 30.14 (-CH_2_-), 30.64 (C-H), 38.81 (-CH_2_-), 45.19 (C-H), 45.58 (-CH_2_-), 89.79 (C_4_, carborane core), 90.81 (C_4_, carborane core), 127.99 (C_sp2_H), 128.45 (C_sp2_H), 128.81 (C_sp2_H), 129.03 (C_sp2_H, 2 atoms), 141. 47 (C_4_, phenyl), 173. 00 (C_4_, -COO^−^).

^11^B NMR (26 MHz, DMSO-*d*_6_): δ = −8.27 (m, 10B).

Elemental analysis: calculated: for C_18_H_37_B_10_O_2_N (%): C 53.03, H 9.15, N 3.44; found (%): C 52.59, H 8.929, N 3.19.

Tert-butylammonium 3-(2-isopropyl-1,2-dicarba-*closo*-dodecaboran-1-yl)-3-phenylpropanoate (**11**). From 3-(2-isopropyl-1,2-dicarba-closo-dodecaboran-1-yl)-3-phenylpropanoic acid 0.09 g (0.27 mmol) and tert-butylamine 0.028 mL (0.27 mmol). Yield—0.06 g (66%); white crystals; mp 223 °C.

FTIR spectrum, ν, cm^−1^: 3000–3200 (NH_3_^+^), 3033 (C-H, ar.), 2820, 2876, 2977 (C-H), 2569, 2593, 2615 (B-H), 1630 (N-H), 1532 (COO^−^, ν_asym),1455 (C=C, ring mode), 1395 (COO^−^, ν_sym), 1010 (in-plane, C-H), 700 (out-of-plane, C-H).

^1^H NMR (80 MHz, DMSO-*d*_6_): δ = 1.5–4.0 (m, 10H, B-H), 1.08 (d, *J* = 3.2 Hz, 6H, CH(CH_3_)_2_), 1.18 (s, 3H, NH_3_^+^), 2.63 (d, *J* = 7.4 Hz, 2H, -CH_2_-), 2.84 (d, J = 6.7 Hz, 1H, CH(CH_3_)_2_), 3.41 (s, 9H, (-CH_3_)_3_), 3.91 (t, *J* = 7.3 Hz, 1H. C-H), 7.20 (s, 5H, C_sp2_H).

^11^B NMR (26 MHz, DMSO-*d*_6_): δ = −7.21 (m, 10B).

Elemental analysis: calculated: for C_18_H_37_B_10_O_2_N (%): C 53.03, H 9.15, N 3.44; found (%): C 52.30, H 8.927, N 3.54.

Allylammonium 3-(2-isopropyl-1,2-dicarba-*closo*-dodecaboran-1-yl)-3-phenylpropanoate (**12**). From 3-(2-isopropyl-1,2-dicarba-closo-dodecaboran-1-yl)-3-phenylpropanoic acid 0.09 g (0.27 mmol) and allylamine 0.020 mL (0.27 mmol). Yield—0.013 g (14%); white crystals; mp 139 °C.

FTIR spectrum, ν, cm^−1^: 3000–3200 (NH_3_^+^), 3033 (C-H, ar.), 2880, 2933, 2984 (C-H), 2557, 2577, 2613 (B-H), 1622 (N-H), 1541 (COO^−^, ν_asym), 1454 (C=C, ring modes), 1400 (COO^−^, ν_sym), 1010 (in-plane, C-H), 702 (out-of-plane, C-H.

^1^H NMR (80 MHz, DMSO-*d*_6_): δ = 1.0–4.0 (m, 10H, B-H), 1.16 (d, *J* = 6.6 Hz, 6H, CH(CH_3_)_2_), 2.44 (q, *J* = 1.8 Hz, 1H, CH(CH_3_)_2_), 2.96–2.59 (m, 4H, -CH_2_-, -CH_2_-), 3.16 (dt, *J* = 5.7, 1.3 Hz, 2H, =CH_2_), 3.92 (t, *J* = 7.3 Hz, 1H, -CH=), 5.30–4.96 (m, 1H, C-H), 7.21 (s, 5H, C_sp2_H), 8.83 (s, 3H, NH_3_^+^)

^13^C NMR (20 MHz, DMSO-*d*_6_): δ = 24.59 (-CH_3_), 24.82 (-CH_3_), 30.68 (C-H), 41.19 (-CH_2_-), 44.45 (-CH_2_-), 44.73 (C-H), 89.18 (C_4_, carborane core), 90.71 (C_4_, carborane core), 119. 17 (=CH_2_), 128.00 (C_sp2_H), 128.49 (C_sp2_H), 128.75 (C_sp2_H), 129.85 (C_sp2_H, 2 atoms), 132.44 (-CH=), 140.90 (C_4_, phenyl), 172. 88 (C_4_, -COO^−^).

^11^B NMR (26 MHz, DMSO-*d*_6_): δ = −6.77 (m, 10B).

Elemental analysis: calculated: for C_17_H_33_B_10_O_2_N (%): C 52.16, H 8.49, N 3.58; found (%): C 52.46, H 8.781, N 3.78.

Ethan-1-amino-2-ammonium 3-(2-isopropyl-1,2-dicarba-*closo*-dodecaboran-1-yl)-3-phenylpropanoate (**13**). From 3-(2-isopropyl-1,2-dicarba-closo-dodecaboran-1-yl)-3-phenylpropanoic acid 0.09 g (0.27 mmol) and ethylenediamine 0.018 mL (0.27 mmol). Yield—0.05 g (54%); white crystals; mp 108 °C.

FTIR spectrum, ν, cm^−1^: 3200–3600 (-NH_2_), 3000–3200 (NH_3_^+^), 3032 (C-H, ar.), 2874, 2940, 2978 (C-H), 2562, 2589, 2615 (B-H), 1621 (N-H), 1558 (COO^−^, ν_asym), 1456 (C=C, ring modes), 1391 (COO^−^, ν_sym), 1011 (in-plane, C-H), 700 (out-of-plane, C-H).

^1^H NMR (80 MHz, DMSO-*d*_6_): δ = 1.0–4.0 (m, 10H, B-H), 1.32 (d, *J* = 6.6 Hz, 6H, CH(CH_3_)_2_), 2.66 (s, 4H, (-CH_2_-)_2_), 3.00 (m, *J* = 6.5 Hz, 2H, -CH_2_-), 3.48 (q, *J* = 7.0 Hz, 1H, CH(CH_3_)_2_), 4.10 (t, *J* = 7.0 Hz, 1H, C-H), 5.99 (s, 5H, -NH_2_, NH_3_^+^), 7.41 (d, *J* = 7.8 Hz, 5H, C_sp2_H).

^13^C NMR (20 MHz, DMSO-*d*_6_): δ = 24.69 (-CH_3_), 24.95 (-CH_3_), 30.75 (C-H), 45.34 (C-H), 45.85 (-CH_2_-), 40.10 (-CH_2_-), 89.81 (C_4_, carborane core), 90.87 (C_4_, carborane core), 127.96 (C_sp2_H), 128.55 (C_sp2_H, 2 atoms), 128.88 (C_sp2_H), 130.00 (C_sp2_H), 141.53 (C_4_, phenyl), 173.82 (C_4_, -COO^−^).

^11^B NMR (26 MHz, DMSO-*d*_6_): δ = −5.97 (m, 10B).

Elemental analysis: calculated: for C_16_H_34_B_10_O_2_N_2_ (%): C 48.72, H 8.69, N 7.10; found (%): C 47.57, H 8.580, N 5.97.

Methylammonium 3-(2-isopropyl-1,2-dicarba-*closo*-dodecaboran-1-yl)-3-phenylpropanoate (**14**). From 3-(2-isopropyl-1,2-dicarba-closo-dodecaboran-1-yl)-3-phenylpropanoic acid 0.09 g (0.27 mmol) and methylamine 0.011 mL (0.27 mmol). Yield—0.07 g (82%); white crystals; mp 160–172 °C.

FTIR spectrum, ν, cm^−1^: 3000–3200 (NH_3_^+^), 3030, 3063 (C-H, ar.), 2789, 2938, 2979 (C-H), 2566, 2587, 2628 (B-H), 1628 (N-H), 1533 (COO^−^, ν_asym), 1455 (C=C, ring modes), 1400 (COO^−^, ν_sym), 1011 (in-plane, C-H), 702 (out-of-plane, C-H).

^1^H NMR (80 MHz, DMSO-*d*_6_): δ = 1.0–4.0 (m, 10H, B-H), 1.16 (d, *J* = 6.7 Hz, 6H, CH(CH_3_)_2_), 1.90–1.55 (THF), 2.08 (s, 3H, -CH_3_), 2.43 (THF), 2.59 (d, J = 7.1 Hz, 2H, -CH_2_-), 2.84 (q, *J* = 6.4 Hz, 1H, CH(CH_3_)_2_), 3.65–3.33 (H_2_O), 3.94 (t, *J* = 7.2 Hz, 1H, C-H), 7.20 (s, 5H, C_sp2_H), 7.68 (s, 3H, NH_3_^+^).

^13^C NMR (20 MHz, DMSO-*d*_6_): δ = 24.33 (-CH_3_), 24.51 (-CH_3_), 24.77 (-CH_3_), 25.54 (C-H), 30.55 (C-H), 45.10 (-CH_2_-), 67.42 (THF), 89.67 (C_4_, carborane core), 90.71 (C_4_, carborane core), 127.79 (C_sp2_H, 2 atoms), 128.36 (C_sp2_H, 2 atoms), 129.85 (C_sp2_H), 141. 36 (C_4_, phenyl), 173. 06 (C_4_, -COO^−^).

^11^B NMR (26 MHz, DMSO-*d*_6_): δ = −8.46 (m, 10B).

Elemental analysis: calculated: for C_15_H_29_B_10_O_2_N (%): C 49.56, H 8.04, N 3.85; found (%): C 48.26, H 8.323, N 3.57.

Cyclohexylammonium 3-(2-isopropyl-1,2-dicarba-*closo*-dodecaboran-1-yl)-3-phenylpropanoate (**15**). From 3-(2-isopropyl-1,2-dicarba-closo-dodecaboran-1-yl)-3-phenylpropanoic acid 0.09 g (0.27 mmol) and cyclohexylamine 0.030 mL (0.27 mmol). Yield—0.08 g (75%); white crystals; mp 215–236 °C.

FTIR spectrum, ν, cm^−1^: 3000–3200 (NH_3_^+^), 3035 (C-H, ar.), 2854, 2924, 2940 (C-H), 2558, 2576, 2596 (B-H), 1627 (N-H), 1538 (COO^−^, ν_asym), 1454 (C=C, ring modes), 1393 (COO^−^, ν_sym), 1011 (in-plane, C-H), 701 (out-of-plane, C-H).

^1^H NMR (500 MHz, DMSO-*d*_6_): δ = 1.5–2.5 (m, 10H, B-H), 1.18–0.99 (m, 7H, (-CH_2_-)_2_, NH_3_^+^), 1.22 (t, *J* = 8.3 Hz, 6H, CH(CH_3_)_2_), 1.54 (d, *J* = 12.5 Hz, 1H, CH(CH_3_)_2_), 1.64 (d, *J* = 13.0 Hz, 2H, -CH_2_-), 1.75 (d, *J* = 11.8 Hz, 2H, -CH_2_-), 2.62 (dd, *J* = 11.3, 6.3 Hz, 2H, -CH_2_-), 2.76–2.68 (m, 1H, C-H), 2.89 (dt, *J* = 13.4, 6.8 Hz, 2H, -CH_2_-), 3.38 (s, H_2_O), 4.00 (dd, *J* = 9.6, 4.8 Hz, 1H, C-H), 7.25 (dd, *J* = 10.1, 4.4 Hz, 2H, C_sp2_H), 7.33–7.28 (m, 2H, C_sp2_H), 7.37 (s, 1H, C_sp2_H).

^11^B NMR (26 MHz, DMSO-*d*_6_): δ = −9.05 (m, 10B).

Elemental analysis: calculated: for C_20_H_39_B_10_O_2_N (%): C 55.40, H 9.07, N 3.23; found (%): C 54.97, H 8.862, N 3.06.

Butylammonium 3-(2-isopropyl-1,2-dicarba-*closo*-dodecaboran-1-yl)-3-(4-fluorophenyl)propanoate (**16**). From 3-(2-isopropyl-1,2-dicarba-closo-dodecaboran-1-yl)-3-(4-fluorophenyl)propanoic acid 0.52 g (1.49 mmol) and butylamine 0.158 mL (1.6 mmol). Yield—0.44 g (75%); white crystals; mp 171.8–182.4 °C.

FTIR spectrum, ν, cm^−1^: 3000–3200 (NH_3_^+^), 3044 (C-H, ar.), 2876, 2941, 2960, 2980 (C-H), 2559, 2591, 2635 (B-H), 1622 (N-H), 1549 (COO^−^, ν_asym), 1509 (C=C, ring modes), 1400 (COO^−^, ν_sym), 1011 (in-plane, C-H), 841 (out-of-plane, C-H).

^1^H NMR (80 MHz, DMSO-*d*_6_): δ = 1.0–4.0 (m, 10H, B-H), 1.49 (m, *J* = 6.2 Hz, 4H, (-CH_2_-)_2_), 1.87 (d, *J* = 6.6 Hz, 9H, (-CH_3_)_3_), 3.20 (d, *J* = 6.5 Hz, 4H, (-CH_2_-)_2_), 3.32 (m. 1H, CH(CH_3_)_2_), 4.62 (t, *J* = 7.4 Hz, 1H, C-H), 8.10–7.55 (m, 4H, C_sp2_H), 8.71 (s, 3H, NH_3_^+^).

^13^C NMR (20 MHz, DMSO-*d*_6_): δ = 16.45 (-CH_3_), 22.21 (-CH_2_-), 27.14 (-CH_3_), 27.33 (-CH_3_), 32.40 (-CH_2_-), 33.18 (C-H), 41.19 (-CH_2_-), 47.03 (C-H). 48.20 (-CH_2_-), 91.87 (C_4_, carborane core), 93.21 (C_4_, carborane core), 117.16 (C_sp2_H), 118.19 (C_sp2_H), 134.14 (C_sp2_H), 134.54 (C_sp2_H), 140.05 (C_4_, benzylidene), 140.21 (C_4_, benzylidene), 175.66 (C_4_, -COO^−^).

^11^B NMR (26 MHz, DMSO-*d*_6_): δ = −5.00 (m, 10B).

^19^F NMR (76 MHz, DMSO-*d*_6_): δ = −112.89 (s, 1F).

Elemental analysis: calculated: for C_18_H_36_B_10_O_2_NF (%): C 50.81, H 8.53, N 3.29; found (%): C 49.70, H 8.597, N 3.04.

Tert-butylammonium 3-(2-isopropyl-1,2-dicarba-*closo*-dodecaboran-1-yl)-3-(4-fluorophenyl)propanoate (**17**). From 3-(2-isopropyl-1,2-dicarba-closo-dodecaboran-1-yl)-3-(4-fluorophenyl)propanoic acid 0.52 g (1.49 mmol) and tert-butylamine 0.168 mL (1.6 mmol). Yield—0.51 g (88%); white crystals; mp 207.5–220.2 °C.

FTIR spectrum, ν, cm^−1^: 3000–3200 (NH_3_^+^), 3033, 3052 (C-H, ar.), 2825, 2884, 2980 (C-H), 2563, 2597, 2630 (B-H), 1629 (N-H), 1530 (COO^−^, ν_asym), 1511 (C=C, ring modes), 1399 (COO^−^, ν_sym), 1010 (in-plane, C-H), 840 (out-of-plane, C-H).

^1^H NMR (80 MHz, DMSO-*d*_6_): δ = 1.0–4.0 (m, 10H, B-H), 1.03 (d, *J* = 6.1 Hz, 6H, CH(CH_3_)_2_), 1.15 (s, 3H, NH_3_^+^), 2.39 (p, *J* = 1.9 Hz, 9H, (-CH_3_)_3_), 2.54 (s, 2H, -CH_2_-), 2.90–2.63 (m, 1H, CH(CH_3_)_2_), 3.91 (t, *J* = 7.3 Hz, 1H, C-H), 7.42–6.75 (m, 4H, C_sp2_H).

^11^B NMR (26 MHz, DMSO-*d*_6_): δ = −7.87 (m, 10B).

^19^F NMR (76 MHz, DMSO-*d*_6_): δ = −113.33 (s, 1F).

Elemental analysis: calculated: for C_18_H_36_B_10_O_2_NF (%): C 50.81, H 8.53, N 3.29; found (%): C 50.42, H 8.421, N 3.15.

Allylammonium 3-(2-isopropyl-1,2-dicarba-*closo*-dodecaboran-1-yl)-3-(4-fluorophenyl)propanoate (**18**). From 3-(2-isopropyl-1,2-dicarba-closo-dodecaboran-1-yl)-3-(4-fluorophenyl)propanoic acid 0.52 g (1.49 mmol) and allylamine 0.12 mL (1.6 mmol). Yield—0.43 g (78%); white crystals; mp 144.3–158.8 °C.

FTIR spectrum, ν, cm^−1^: 3000–3200 (NH_3_^+^), 3010–3100 (C-H, ar.), 2886, 2932, 2980 (C-H), 2562, 2588, 2611 (B-H), 1620 (N-H), 1541 (COO^−^, ν_asym), 1510 (C=C, ring modes), 1403 (COO^−^, ν_sym), 1012 (in-plane, C-H), 841 (out-of-plane, C-H).

^1^H NMR (80 MHz, DMSO-*d*_6_): δ = 1.0–4.0 (m, 10H, B-H), 1.10 (d, *J* = 6.6 Hz, 6H, CH(CH_3_)_2_), 2.38 (t, *J* = 1.9 Hz, 1H, CH(CH_3_)_2_), 2.54 (m, *J* = 7.4 Hz, 2H, -CH_2_-), 3.06 (d, *J* = 5.5 Hz, 2H, -CH_2_-), 3.88 (t, *J* = 7.2 Hz, 1H, C-H), 5.23–4.83 (m, 2H, -CH=CH_2_), 5.86–5.37 (m, 1H, -CH=CH_2_), 7.31–6.83 (m, 4H, C_sp2_H), 7.66 (s, 3H, NH_3_^+^).

^13^C NMR (20 MHz, DMSO-*d*_6_): δ = 24.49 (-CH_3_), 24.68 (-CH_3_), 30.55 (C-H), 41.33 (CH_2_), 44.21 (C-H), 45.20 (CH_2_), 89.12 (C_4_, carborane core), 90.56 (C_4_, carborane core), 114.54 (C_sp2_H), 115.59 (C_sp2_H), 118.50 (=CH_2_), 131.69 (C_sp2_H), 133.05 (C_sp2_H), 137.21 (C_4_, benzylidene), 137.37 (C_4_, benzylidene), 172.97 (C_4_, -COO^−^).

^11^B NMR (26 MHz, DMSO-*d*_6_): δ = −7.32 (m, 10B).

^19^F NMR (76 MHz, DMSO-*d*_6_): δ = 113.04 (s, 1F).

Elemental analysis: calculated: for C_17_H_32_B_10_O_2_NF (%): C 49.87, H 7.87, N 3.42; found (%): C 49.25, H 8.141, N 3.20.

Ethan-1-amino-2-ammonium 3-(2-isopropyl-1,2-dicarba-*closo*-dodecaboran-1-yl)-3-(4-fluorophenyl)propanoate (**19**). From 3-(2-isopropyl-1,2-dicarba-closo-dodecaboran-1-yl)-3-(4-fluorophenyl)propanoic acid 0.52 g (1.49 mmol) and ethylenediamine 0.1 mL (1.6 mmol). Yield—0.25 g (44%); white crystals; mp 65.7–81.5 °C.

FTIR spectrum, ν, cm^−1^: 3200–3400 (-NH_2_), 3000–3200 (NH_3_^+^), 3060–3100 (C-H, ar.), 2857, 2943, 2973 (C-H), 2562, 2581, 2619 (B-H), 1625 (N-H), 1560 (COO^−^, ν_asym), 1507 (C=C, ring modes), 1390 (COO^−^, ν_sym), 1010 (in-plane, C-H), 843 (out-of-plane, C-H).

^1^H NMR (80 MHz, DMSO-*d*_6_): δ = 1.0–3.0 (m, 10H, B-H), 1.14 (d, *J* = 6.6 Hz, 6H, CH(CH_3_)_2_), 2.48 (m, 3H, NH_3_^+^), 2.62 (d, *J* = 7.8 Hz, 2H, -CH_2_-), 2.75 (m, 2H, -NH_2_), 3.30 (q, J = 6.9 Hz, 1H, CH(CH_3_)_2_), 3.92 (t, J = 7.1 Hz, 1H, C-H), 4.99 (s, 4H, (-CH_2_-)_2_), 7.43–6.78 (m, 4H, C_sp2_H).

^13^C NMR (20 MHz, DMSO-*d*_6_): δ = 24.51 (-CH_3_), 24.73 (-CH_3_), 30.58 (C-H), 40.92 (-CH_2_-)2, 44.45 (C-H), 45.82 (-CH_2_-), 89.37 (C_4_, carborane core), 90.65 (C_4_, carborane core), 114.56 (C_sp2_H), 115.62 (C_sp2_H), 131.51 (C_sp2_H), 131.89 (C_sp2_H), 137.45 (C_4_, benzylidene), 137.61 (C_4_, benzylidene), 173.51 (C_4_, -COO^−^).

^11^B NMR (26 MHz, DMSO-*d*_6_): δ = −7.68 (m, 5B), −34.50 (m, 5B).

^19^F NMR (76 MHz, DMSO-*d*_6_): δ = 112.90 (s, 1F).

Elemental analysis: calculated: for C_16_H_33_B_10_O_2_N_2_F (%): C 46.60, H 8.06, N 6.79; found (%): C 46.75, H 8.281, N 7.01.

Methylammonium 3-(2-isopropyl-1,2-dicarba-*closo*-dodecaboran-1-yl)-3-(4-fluorophenyl)propanoate (**20**). From 3-(2-isopropyl-1,2-dicarba-closo-dodecaboran-1-yl)-3-(4-fluorophenyl)propanoic acid 0.52 g (1.49 mmol) and methylamine 0.15 mL (1.6 mmol). Yield—0.34 g (66%); white crystals; mp 171.5–180.8 °C.

FTIR spectrum, ν, cm^−1^: 3000–3200 (NH_3_^+^), 3068, 3100 (C-H, ar.), 2804, 2900, 3000 (C-H), 2563, 2573, 2590 (B-H), 1626 (N-H), 1550 (COO^−^, ν_asym), 1508 (C=C, ring modes), 1396 (COO^−^, ν_sym), 1012 (in-plane, C-H), 845 (out-of-plane, C-H).

^1^H NMR (80 MHz, DMSO-*d*_6_): δ = 1.0–3.5 (m, 10H, B-H), 1.24 (d, *J* = 6.6 Hz, 6H, CH(CH_3_)_2_), 2.17 (s, 3H, -CH_3_), 2.52 (d, 2H, -CH_2_-), 2.77 (m. *J* = 10.2 Hz, 1H, CH(CH_3_)_2_), 4.02 (t, *J* = 7.3 Hz, 1H, C-H), 7.44–6.92 (m, 4H, C_sp2_H), 8.45 (s, 3H, NH_3_^+^).

^13^C NMR (20 MHz, DMSO-*d*_6_): δ = 24.78 (-CH_3_), 24.29 (-CH_3_), 24.58 (-CH_3_), 30.63 (C-H), 44.50 (C-H), 45.74 (-CH_2_-), 89.44 (C_4_, carborane core), 90.68 (C_4_, carborane core), 114.60 (C_sp2_H), 115.66 (C_sp2_H), 131.60 (C_sp2_H), 132.00 (C_sp2_H), 137.52 (C_4_, benzylidene), 137.68 (C_4_, benzylidene), 173.20 (C_4_, -COO^−^).

^11^B NMR (26 MHz, DMSO-*d*_6_): δ = −7.5 (m, 10B).

^19^F NMR (76 MHz, DMSO-*d*_6_): δ = −112.99 (s, 1F).

Elemental analysis: calculated: for C_15_H_28_B_10_O_2_NF (%): C 47.23, H 7.40, N 3.67; found (%): C 46.01, H 7.706, N 3.39.

Cyclohexylammonium 3-(2-isopropyl-1,2-dicarba-*closo*-dodecaboran-1-yl)-3-(4-fluorophenyl)propanoate (**21**). From 3-(2-isopropyl-1,2-dicarba-closo-dodecaboran-1-yl)-3-(4-fluorophenyl)propanoic acid 0.52 g (1.49 mmol) and cyclohexylamine 0.18 mL (1.6 mmol). Yield—0.5 g (80%); white crystals; mp 216.4–225.7 °C.

FTIR spectrum, ν, cm^−1^: 3000–3200 (NH_3_^+^), 3046 (C-H, ar.), 2857, 2941, 2974 (C-H), 2560, 2590, 2637 (B-H), 1625 (N-H), 1545 (COO^−^, ν_asym), 1509 (C=C, ring modes), 1402 (COO^−^, ν_sym), 1011 (in-plane, C-H), 841 (out-of-plane, C-H).

^1^H NMR (80 MHz, DMSO-*d*_6_): δ = 1.0–4.0 (m, 10H, B-H), 0.94 (s, 3H, NH_3_^+^), 1.08 (d, *J* = 6.7 Hz, 6H, CH(CH_3_)_2_), 1.49 (m, 4H, (-CH_2_-)_2_), 2.36 (p, *J* = 2.0 Hz, 7H, C-H, (-CH_2_-)_3_), 2.52 (m, 2H, -CH_2_-), 2.52 (s, 2H, -CH_2_-), 2.66 (m, *J* = 6.9 Hz, 1H, CH(CH_3_)_2_), 3.87 (t, *J* = 7.1 Hz, 1H, C-H), 7.06 (p, *J* = 8.7 Hz, 4H, C_sp2_H).

^11^B NMR (26 MHz, DMSO-*d*_6_): δ = −7. 68 (m, 10B).

^19^F NMR (76 MHz, DMSO-*d*_6_): δ = −113.27 (s, 1F).

Elemental analysis: calculated: for C_20_H_38_B_10_O_2_NF (%): C 53.18, H 8.48, N 3.10; found (%): C 52.73, H 8.423, N 2.86.

Butylammonium 3-(2-isopropyl-1,2-dicarba-*closo*-dodecaboran-1-yl)-3-(naphthalene-1-yl)propanoate (**22**). From 3-(2-isopropyl-1,2-dicarba-closo-dodecaboran-1-yl)-3-(naphthalene-1-yl)propanoic acid 0.1 g (0.27 mmol) and butylamine 0.028 mL (0.27 mmol). Yield—0.1 g (88%); white crystals; mp 198–205 °C.

FTIR spectrum, ν, cm^−1^: 3000–3200 (NH_3_^+^), 3066 (C-H, ar.), 2875, 2992 (C-H), 2556, 2603, 2641 (B-H), 1631 (N-H), 1544 (COO^−^, ν_asym), 1460 (C=C, ring modes), 1385 (COO^−^, ν_sym), 1373 (-CH_3_), 741, 780, 795 (out-of-plane, C-H).

^1^H NMR (500 MHz, DMSO-*d*_6_): δ = 1.5–2.65 (m, 10H, B-H), 0.76 (t, *J* = 7.3 Hz, 4H, (-CH_2_-)_2_), 1.16 (m, *J* = 7.3 Hz, 2H, -CH_2_-), 1.27 (m, 9H, CH(CH_3_)_3_), 2.77 (m, 2H, -CH_2_-), 2.95 (m, *J* = 6.5 Hz, 1H, CH(CH_3_)_2_), 3.32 (m, 3H, NH_3_^+^, H_2_O), 5.05 (dd, *J* = 10.2, 4.3 Hz, 1H, C-H), 7.48 (m, *J* = 7.7, 2.8 Hz, 2H, C_sp2_H), 7.54 (m, 2H, C_sp2_H), 7.82 (d, *J* = 8.0 Hz, 1H, C_sp2_H), 7.89 (d, J = 8.1, 1.5 Hz, 1H, C_sp2_H), 8.16 (d, J = 8.6 Hz, 1H, C_sp2_H).

^13^C NMR (126 MHz, DMSO-*d*_6_): δ = 14.00 (-CH_3_), 19.62 (-CH_2_-), 24.71 (-CH_3_), 24.95 (-CH_3_), 29.88 (-CH_2_-), 30.50 (C-H), 38.64 (C-H), 38.73 (-CH_2_-), 46.68 (-CH_2_-), 89.94 (C_4_, carborane core), 90.70 (C_4_, carborane core), 124.07 (C_sp2_H), 125.77 (C_sp2_H), 126.04 (C_sp2_H), 126.51 (C_sp2_H), 127.36 (C_sp2_H), 128.54 (C_sp2_H), 129.22 (C_sp2_H), 132.03 (C_4_, naphthyl), 133.90 (C_4_, naphthyl), 138.18 (C_4_, naphthyl), 172.89 (C_4_, -COO^−^).

^11^B NMR (160 MHz, DMSO-*d*_6_): δ = −6.43 (m, 4B), −13.11 (m, 6B).

Elemental analysis: calculated: for C_22_H_39_B_10_O_2_N (%): C 57.75, H 8.59, N 3.06; found (%): C 57.02, H 8.129, N 2.94.

Tert-butylammonium 3-(2-isopropyl-1,2-dicarba-*closo*-dodecaboran-1-yl)-3-(naphthalene-1-yl)propanoate (**23**). From 3-(2-isopropyl-1,2-dicarba-closo-dodecaboran-1-yl)-3-(naphthalene-1-yl)propanoic acid 0.1 g (0.27 mmol) and tert-butylamine 0.028 mL (0.27 mmol). Yield—0.07 g (68%); white crystals; mp 190–222 °C.

FTIR spectrum, ν, cm^−1^: 3000–3200 (NH_3_^+^), 3065 (C-H, ar.), 2875–3000 (C-H), 2555–2600 (B-H), 1631 (N-H), 1535 (COO^−^, ν_asym), 1464 (C=C, ring modes), 1381 (COO^−^, ν_sym), 1373 (-CH_3_), 739, 783, 795 (out-of-plane, C-H).

^1^H NMR (500 MHz, DMSO-*d*_6_): δ = 1.5–2.5 (m, 10H, B-H), 1.00 (s, 9H, (-CH_3_)_3_), 1.27 (dd, *J* = 10.5, 6.7 Hz, 6H, CH(CH_3_)_2_), 2.76 (m, 2H, -CH_2_-), 2.96 (m, *J* = 6.8 Hz, 1H, CH(CH_3_)_2_), 3.32 (m, *J* = 31.7, 16.7 Hz, 3H, NH_3_^+^, H_2_O), 5.07 (dd, *J* = 10.1, 4.2 Hz, 1H, C-H), 7.47 (m, 2H, C_sp2_H), 7.53 (m, *J* = 8.3, 5.0, 1.5 Hz, 2H, C_sp2_H), 7.81 (d, *J* = 8.0 Hz, 1H, C_sp2_H), 7.88 (d, *J* = 8.1, 1.4 Hz, 1H, C_sp2_H), 8.16 (d, *J* = 8.7 Hz, 1H, C_sp2_H).

^13^C NMR (126 MHz, DMSO-*d*_6_): δ = 24.69 (-CH_3_), 24.95 (-CH_3_), 24.77 (-CH_3_), 30.47 (C-H), 38.74 (C-H), 46.96 (-CH_2_-), 50.45 (-CH_3_), 79.73 (C_4_, tert-butylammonium), 89.89 (C_4_, carborane core), 90.79 (C_4_, carborane core), 124.15 (C_sp2_H), 125.78 (C_sp2_H), 126.01 (C_sp2_H), 126.45 (C_sp2_H), 127.33 (C_sp2_H), 128.47 (C_sp2_H), 129.19 (C_sp2_H), 132.08 (C_4_, naphthyl), 133.93 (C_4_, naphthyl), 138.34 (C_4_, naphthyl), 172.74 (C_4_, -COO^−^).

^11^B NMR (160 MHz, DMSO-*d*_6_): δ = −5.58 (m, 5B), −12.36 (m, 5B).

Elemental analysis: calculated: for C_22_H_39_B_10_O_2_N (%): C 57.75, H 8.59, N 3.06; found (%): C 56.97, H 8.403, N 2.75.

Allylammonium 3-(2-isopropyl-1,2-dicarba-*closo*-dodecaboran-1-yl)-3-(naphthalene-1-yl)propanoate (**24**). From 3-(2-isopropyl-1,2-dicarba-closo-dodecaboran-1-yl)-3-(naphthalene-1-yl)propanoic acid 0.1 g (0.27 mmol) and allylamine 0.020 mL (0.27 mmol). Yield—0.08 g (75%); white crystals; mp 169–176 °C.

FTIR spectrum, ν, cm^−1^: 3000–3200 (NH_3_^+^), 3000–3100 (C-H, ar.), 2700–2995 (C-H), 2555–2650 (B-H), 1625 (N-H), 1541 (COO^−^, ν_asym), 1462 (C=C, ring modes), 1389 (COO^−^, ν_sym), 1374 (-CH_3_), 743, 781, 795 (out-of-plane, C-H).

^1^H NMR (500 MHz, DMSO-*d*_6_): δ = 1.5–2.5 (m, 10H, B-H), 1.29 (m, *J* = 6.9 Hz, 6H, CH(CH_3_)_2_), 2.88 (m, *J* = 35.2, 14.4, 5.5 Hz, 2H, -CH_2_-), 3.00 (m, *J* = 15.1, 10.9 Hz, 1H, CH(CH_3_)_2_), 3.08 (dd, *J* = 5.8, 1.7 Hz, 2H, -CH_2_-), 3.5–4.5 (m, 3H, NH_3_^+^), 5.00 (m, 2H, =CH_2_), 5.08 (m, *J* = 17.1, 1.6 Hz, 1H, -CH=), 5.67 (m, 1H, C-H), 7.29 (Benzene), 7.44 (t, *J* = 7.3 Hz, 1H, C_sp2_H), 7.49 (m, *J* = 8.5, 6.8, 1.6 Hz, 1H, C_sp2_H), 7.57 (d, *J* = 7.4 Hz, 1H, C_sp2_H), 7.75 (d, *J* = 8.1 Hz, 1H, C_sp2_H), 7.81 (d, 1H, C_sp2_H), 7.86 (d, *J* = 1.4 Hz, 1H, C_sp2_H), 8.16 (d, *J* = 8.6 Hz, 1H, C_sp2_H).

^13^C NMR (126 MHz, DMSO-*d*_6_): δ = 24.74 (-CH_3_), 24.98 (-CH_3_), 30.55 (C-H), 38.33 (C-H), 40.20 (C-H), 41.65 (-CH_2_-), 45.88 (-CH_2_-), 89.95 (C_4_, carborane core), 90.32 (C_4_, carborane core), 118.91 (=CH_2_), 123.98 (C_sp2_H), 125.82 (C_sp2_H), 126.14 (C_sp2_H), 126.65 (C_sp2_H), 127.36 (C_sp2_H), 128.88 (C_sp2_H), 129.27 (C_sp2_H), 132.00 (C_4_, naphthyl), 133.14 (C_4_, naphthyl), 133.93 (C_4_, naphthyl), 172. 82 (C_4_, -COO^−^).

^11^B NMR (160 MHz, DMSO-*d*_6_): δ = −5.25 (m, 5B), −11.26 (m, 5B).

Elemental analysis: calculated: for C_21_H_35_B_10_O_2_N (%): C 57.11, H 7.99, N 3.17; found (%): C 56.21, H 7.845, N 2.79.

Ethan-1-amino-2-ammonium 3-(2-isopropyl-1,2-dicarba-*closo*-dodecaboran-1-yl)-3-(naphthalene-1-yl)propanoate (**25**). From 3-(2-isopropyl-1,2-dicarba-closo-dodecaboran-1-yl)-3-(naphthalene-1-yl)propanoic acid 0.1 g (0.27 mmol) and ethylenediamine 0.018 mL (0.27 mmol). Yield—0.1 g (93%); white crystals; mp 173–179 °C.

FTIR spectrum, ν, cm^−1^: 3000–3200 (NH_3_^+^), 3063 (C-H, ar.), 2850–2987 (C-H), 2566–2650 (B-H), 1620 (N-H), 1540 (COO^−^, ν_asym), 1462 (C=C, ring modes), 1385 (COO^−^, ν_sym), 1372 (-CH_3_), 738, 779, 794 (out-of-plane, C-H).

^1^H NMR (500 MHz, CDCl_3_): δ = 1.5–2.6 (m, 10H, B-H), 1.30 (dd, *J* = 21.3, 6.7 Hz, 6H, CH(CH_3_)_2_), 1.79 (s, 2H, -NH_2_), 2.71 (m, *J* = 6.9 Hz, 1H, CH(CH_3_)_2_), 2.89 (m, 2H, -CH_2_-), 3.32 (s, 7H, NH_3_^+^, (-CH_2_-)_2_), 4.88 (dd, *J* = 11.1, 5.0 Hz, 1H, C-H), 7.37 (m, *J* = 17.9 Hz, 1H, C_sp2_H), 7.46 (m, 2H, C_sp2_H), 7.56 (d, *J* = 7.4, 1.2 Hz, 1H, C_sp2_H), 7.70 (d, *J* = 8.2 Hz, 1H, C_sp2_H), 7.79 (d, *J* = 7.8, 1.9 Hz, 1H, C_sp2_H), 8.11 (d, *J* = 8,2 Hz, 1H, C_sp2_H).

^11^B NMR (26 MHz, DMSO-*d*_6_): δ = −9.28 (m, 10B).

Elemental analysis: calculated: for C_20_H_36_B_10_O_2_N_2_ (%): C 54.04, H 8.16, N 6.30; found (%): C 54.56, H 8.183, N 5.58.

Methylammonium 3-(2-isopropyl-1,2-dicarba-*closo*-dodecaboran-1-yl)-3-(naphthalene-1-yl)propanoate (**26**). From 3-(2-isopropyl-1,2-dicarba-closo-dodecaboran-1-yl)-3-(naphthalene-1-yl)propanoic acid 0.1 g (0.27 mmol) and methylamine 0.011 mL (0.27 mmol). Yield—0.04 g (46%); white crystals; mp 165–193 °C.

FTIR spectrum, ν, cm^−1^: 3000–3200 (NH_3_^+^), 3069 (C-H, ar.), 2875–3000 (C-H), 2557–2640 (B-H), 1635 (N-H), 1540 (COO^−^, ν_asym), 1462 (C=C, ring modes), 1388 (COO^−^, ν_sym), 1371 (-CH_3_), 741, 780, 795 (out-of-plane, C-H).

^1^H NMR (500 MHz, DMSO-*d*_6_): δ = 1.4–2.4 (m, 10H, B-H), 1.27 (dd, *J* = 10.2, 6.8 Hz, 6H, CH(CH_3_)_2_), 2.11 (s, 3H, -CH_3_), 2.77 (m, 2H, -CH_2_-), 2.95 (m, *J* = 6.6 Hz, 1H, CH(CH_3_)_2_), 3.32 (s, 3H, NH_3_^+^, H_2_O), 5.05 (dd, *J* = 10.3, 4.3 Hz, 1H, C-H), 7.33 (s, benzene), 7.48 (m, *J* = 7.6, 3.3 Hz, 2H, C_sp2_H), 7.55 (m, 1H, C_sp2_H), 7.82 (d, *J* = 8.0 Hz, 1H, C_sp2_H), 7.90 (d, *J* = 8.3, 1.4 Hz, 1H, C_sp2_H), 8.16 (d, *J* = 8.7 Hz, 1H, C_sp2_H), 8.29 (s, 1H, C_sp2_H).

^13^C NMR (126 MHz, DMSO-*d*_6_): δ = 24.54 (-CH_3_), 24.73 (-CH_3_), 24.97 (-CH_3_), 30.52 (C-H), 38.51 (C-H), 46.36 (-CH_2_-), 89.98 (C_4_, carborane core), 90.58 (C_4_, carborane core), 124.06 (C_sp2_H), 125.82 (C_sp2_H), 126.11 (C_sp2_H), 126.59 (C_sp2_H), 127.37 (C_sp2_H), 128.61 (C_sp2_H), 128.88 (C_sp2_H), 129.25 (C_4_, naphthyl), 132.03 (C_4_, naphthyl), 133.91 (C_4_, naphthyl), 172.99 (C_4_, -COO^−^).

^11^B NMR (160 MHz, DMSO-*d*_6_): δ = −5.38 (m, 4B), −11.19 (m, 6B).

Elemental analysis: calculated: for C_19_H_31_B_10_O_2_N (%): C 55.20, H 7.56, N 3.39; found (%): C 54.19, H 8.128, N 2.78.

Cyclohexylammonium 3-(2-isopropyl-1,2-dicarba-*closo*-dodecaboran-1-yl)-3-(naphthalene-1-yl)propanoate (**27**). From 3-(2-isopropyl-1,2-dicarba-closo-dodecaboran-1-yl)-3-(naphthalene-1-yl)propanoic acid 0.1 g (0.27 mmol) and cyclohexylamine 0.030 mL (0.27 mmol). Yield—0.1 g (83%); white crystals; mp 221–239 °C.

FTIR spectrum, ν, cm^−1^: 3000–3200 (NH_3_^+^), 3066 (C-H, ar.), 2858, 2933, 2980, 2995 (C-H), 2555–2642 (B-H), 1630 (N-H), 1535 (COO^−^, ν_asym), 1454 (C=C, ring modes), 1391 (COO^−^, ν_sym), 1373 (-CH_3_), 742, 781, 795 (out-of-plane, C-H).

^1^H NMR (500 MHz, DMSO-*d*_6_): δ = 1.5–2.5 (m, 10H, B-H), 0.99 (m, 4H, (-CH_2_-)_2_), 1.27 (m, 6H, CH(CH_3_)_2_), 1.53 (m, 4H, (-CH_2_-)_2_), 2.57 (m, *J* = 12.1, 7.7, 4.3 Hz, 1H, C-H), 2.85–2.68 (m, 2H, -CH_2_-), 2.95 (m, *J* = 7.3 Hz, 1H, CH(CH_3_)_2_), 3.32 (m, 5H, -CH_2_-, NH_3_^+^), 5.06 (dd, *J* = 10.3, 4.3 Hz, 1H, C-H), 7.51 (m, *J* = 31.2, 7.7, 4.2 Hz, 4H, C_sp2_H), 7.81 (d, *J* = 8.1 Hz, 1H, C_sp2_H), 7.88 (d, *J* = 8.2 Hz, 1H, C_sp2_H), 8.16 (d, *J* = 8.6 Hz, 1H, C_sp2_H).

^13^C NMR (126 MHz, DMSO-*d*_6_): δ = 24.41 (-CH_2_-), 24.71 (-CH_3_), 24.98 (-CH_3_), 25.22 (-CH_2_-), 30.48 (C-H), 31.68 (C-H), 38.74 (C-H), 49.50 (-CH_2_-), 79.25 (-CH_2_-), 79.52 (-CH_2_-), 79.78 (-CH_2_-), 89.99 (C_4_, carborane core), 90.78 (C_4_, carborane core), 124.15 (C_sp2_H), 125.77 (C_sp2_H), 126.03 (C_sp2_H), 126.46 (C_sp2_H), 127.38 (C_sp2_H), 128.50 (C_sp2_H), 129.20 (C_sp2_H), 132.06 (C_4_, naphthyl), 133.91 (C_4_, naphthyl), 138.28 (C_4_, naphthyl), 172.33 (C_4_, -COO^−^).

^11^B NMR (160 MHz, DMSO-*d*_6_): δ = −7.54 (m, 5B), −12.86 (m, 5B).

Elemental analysis: calculated: for C_24_H_41_B_10_O_2_N (%): C 59.58, H 8.54, N 2.90; found (%): C 59.82, H 8.580, N 3.35.

#### 3.6.2. Synthesis of Isopropyl-o-carboranyl Containing 3-Arylpropanoic Acid with Secondary Amines

Morpholine 3-(2-isopropyl-1,2-dicarba-*closo*-dodecaboran-1-yl)-3-phenylpropanoate salt (**28**). From 3-(2-isopropyl-1,2-dicarba-closo-dodecaboran-1-yl)-3-phenylpropanoic acid 0.09 g (0.27 mmol) and morpholine 0.023 mL (0.27 mmol). Yield—0.09 g (90%); white crystals; mp 139–145 °C.

FTIR spectrum, ν, cm^−1^: 3000–3200 (NH_2_^+^), 3036 (C-H, ar.), 2866, 2921, 2976 (C-H), 2569, 2602, 2621 (B-H), 1628 (N-H), 1574 (COO^−^, ν_asym), 1456 (C=C, ring modes), 1390 (COO^−^, ν), 1239 (C-N), 1107 (C-O-C, ν_asym), 1048 (C-O-C, ν_sym), 1009 (in-plane, C-H), 704 (out-of-plane, C-H).

^1^H NMR (80 MHz, CDCl_3_): δ = 1.5–4.5 (m, 10H, B-H), 1.44 (dd, *J* = 6.9, 1.9 Hz, 6H, CH(CH_3_)_2_), 2.73–2.58 (m, 4H, (-CH_2_-)_2_), 3.03 (m, *J* = 4.2 Hz, 3H, CH(CH_3_)_2_, -CH_2_-), 3.58 (m, *J* = 6.3, 3.4 Hz, 4H, (-CH_2_-)_2_), 4.02 (m, *J* = 9.5, 6.3 Hz, 1H, C-H), 7.40 (s, 5H, C_sp2_H), 9.99 (s, 2H, NH_2_^+^).

^13^C NMR (20 MHz, CDCl_3_): δ = 24. 62 (-CH_3_), 24.83 (-CH_3_), 30.92 (C-H), 42.87 (-CH_2_-), 44.86 (-CH_2_-), 45.04 (C-H), 64.15 (-CH_2_-), 87.42 (C_4_, carborane core), 89.50 (C_4_, carborane core), 128.06 (C_sp2_H, 2 atoms), 128.50 (C_sp2_H, 2 atoms), 129. 55 (C_sp2_H), 140.42 (C_4_, phenyl), 176.97 (C_4_, -COO^−^).

^11^B NMR (26 MHz, CDCl_3_): δ = −7.14 (m, 10 B).

Elemental analysis: calculated: for C_18_H_35_B_10_O_3_N (%): C 51.28, H 8.37, N 3.32; found (%): C 50.99, H 8.122, N 3.05.

Piperidine 3-(2-isopropyl-1,2-dicarba-*closo*-dodecaboran-1-yl)-3-phenylpropanoate salt (**29**). From 3-(2-isopropyl-1,2-dicarba-closo-dodecaboran-1-yl)-3-phenylpropanoic acid 0.09 g (0.27 mmol) and piperidine 0.026 mL (0.27 mmol). Yield—0.08 g (78%); white crystals; mp 190 °C.

FTIR spectrum, ν, cm^−1^: 3000–3200 (NH_2_^+^), 3030 (C-H, ar.), 2869, 2930, 2958 (C-H), 2555, 2582, 2623 (B-H), 1623 (N-H), 1551 (COO^−^, ν_asym), 1454 (C=C, ring modes), 1391 (COO^−^, ν_sym), 1074 (C-N), 1010 (in-plane, C-H), 701 (out-of-plane, C-H).

^1^H NMR (80 MHz, CDCl_3_): δ = 1.0–4.0 (m, 10H, B-H), 1.41 (d, *J* = 7.5 Hz, 10H, CH(CH_3_)_2_, (-CH_2_-)_2_), 2.52 (s, 4H, (-CH_2_-)_2_), 2.95 (m, *J* = 6.6 Hz, 5H, (-CH_2_-), CH(CH_3_)_2_, -CH_2_-), 4.05 (t, *J* = 7.9 Hz, 1H, C-H), 7.39 (s, 5H, C_sp2_H), 9.83 (s, 2H, NH_2_^+^).

^13^C NMR (20 MHz, CDCl_3_): δ = 22.24 (-CH_2_-), 24.52 (-CH_3_), 24.70 (-CH_3_), 30.72 (C-H), 43.49 (-CH_2_-), 44.74 (-CH_2_-), 44.96 (C-H), 87. 67 (C_4_, carborane core), 89.45 (C_4_, carborane core), 127.70 (C_sp2_H, 2 atoms), 128.26 (C_sp2_H, 2 atoms), 129.52 (C_sp2_H), 140.53 (C_4_, phenyl), 176.34 (C_4_, -COO^−^).

^11^B NMR (26 MHz, CDCl_3_): δ = −8.82 (m, 10B).

Elemental analysis: calculated: for C_19_H_37_B_10_O_2_N (%): C 54.39, H 8.89, N 3.34; found (%): C 53.58, H 8.740, N 2.89.

Diethylammonium 3-(2-isopropyl-1,2-dicarba-*closo*-dodecaboran-1-yl)-3-phenylpropanoate salt (**30**). From 3-(2-isopropyl-1,2-dicarba-closo-dodecaboran-1-yl)-3-phenylpropanoic acid 0.09 g (0.27 mmol) and diethylamine 0.028 mL (0.27 mmol). Yield—0.05 g (54%); white crystals; mp 194.6 °C.

FTIR spectrum, ν, cm^−1^: 3000–3200 (NH_2_^+^), 3024 (C-H, ar.), 2876, 2942, 2985 (C-H), 2557, 2598, 2630 (B-H), 1635 (N-H), 1559 (COO^−^, ν_asym), 1468 (C=C, ring modes), 1384 (COO^−^, ν_sym), 1071 (C-N), 1010 (in-plane, C-H), 700 (out-of-plane, C-H).

^1^H NMR (80 MHz, CDCl_3_): δ = 1.0–4.0 (m, 10H, B-H), 0.94 (t, *J* = 7.2 Hz, 6H, (-CH_3_)_2_), 1.36 (dd, *J* = 6.8, 2.6 Hz, 6H, CH(CH_3_)_2_), 2.47 (m, *J* = 7.3 Hz, 4H, (-CH_2_-)_2_), 2.93 (d, *J* = 7.9 Hz, 3H, CH(CH_3_)_2_, -CH_2_-), 4.00 (t, *J* = 7.7 Hz, 1H, C-H), 7.33 (s, 5H, C_sp2_H), 10.03 (s, 2H, NH_2_^+^).

^13^C NMR (20 MHz, CDCl_3_): δ = 10.94 (-CH_3_), 24.52 (-CH_3_), 24.80 (-CH_3_), 30.79 (C-H), 41.67 (-CH_2_-), 44.72 (-CH_2_-), 44.97 (C-H), 87.64 (C_4_, carborane core), 89.55 (C_4_,), 127.80 (C_sp2_H, 2 atoms), 128.25 (C_sp2_H, 2 atoms), 129.46 (C_sp2_H), 140.54 (C_4_, phenyl), 175.41 (C_4_, -COO^−^).

^11^B NMR (26 MHz, CDCl_3_): δ = −5.97 (m, 10B).

Elemental analysis: calculated: for C_18_H_37_B_10_O_2_N (%): C 53.03, H 9.15, N 3.44; found (%): C 53.32, H 9.304, N 3.84.

Morpholine 3-(2-isopropyl-1,2-dicarba-*closo*-dodecaboran-1-yl)-3-(4-fluorophenyl)propanoate salt (**31**). From 3-(2-isopropyl-1,2-dicarba-closo-dodecaboran-1-yl)-3-(4-fluorophenyl)propanoic acid 0.52 g (1.49 mmol) and morpholine 0.138 mL (1.6 mmol). Yield—0.51 g (85%); white crystals; mp 166.5–176.5 °C.

FTIR spectrum, ν, cm^−1^: 3000–3200 (NH_2_^+^), 3042, 3053 (C-H, ar.), 2870, 2969, 2985 (C-H), 2550, 2565, 2611 (B-H), 1631 (N-H), 1572 (COO^−^, ν_asym), 1510 (C=C, ring modes), 1392 (COO^−^, ν_asym), 1216 (C-N), 1108 (C-O-C, ν_asym), 1051 (C-O-C, ν_sym), 1014 (in-plane, C-H), 817 (out-of-plane, C-H).

^1^H NMR (80 MHz, DMSO-*d*_6_): δ = 1.0–4.0 (m, 10H, B-H), 1.16 (d, *J* = 6.7 Hz, 6H, CH(CH_3_)_2_), 2.45 (d, *J* = 2.2 Hz, 1H, CH(CH_3_)_2_), 2.66 (d, *J* = 3.4 Hz, 2H, -CH_2_-), 2.86–2.72 (m, 4H, (-CH_2_-)_2_), 3.46 (dd, *J* = 6.1, 3.4 Hz, 4H, (-CH_2_-)_2_), 3.91 (t, *J* = 7.6 Hz, 1H, C-H), 7.47–6.86 (m, 4H, C_sp2_H), 8.11 (s, 2H, NH_2_^+^).

^13^C NMR (20 MHz, DMSO-*d*_6_): δ = 24.56 (-CH_3_), 24.77 (-CH_3_), 30.64 (C-H), 43.83 (C-H), 44. 03 (-CH_2_-), 65. 24 (-CH_2_-), 88.68 (C_4_, carborane core), 90.66 (C_4_, carborane core), 114.71 (C_sp2_H), 115.76 (C_sp2_H), 131.53 (C_sp2_H), 131.95 (C_sp2_H), 136.79 (C_4_, benzylidene), 136.95 (C_4_, benzylidene), 172.76 (C_4_, -COO^−^).

^11^B NMR (26 MHz, DMSO-*d*_6_): δ = −8.05 (m, 10B).

^19^F NMR (76 MHz, DMSO-*d*_6_): δ = −112.69 (s, 1F).

Elemental analysis: calculated: for C_18_H_34_B_10_O_3_NF (%): C 49.20, H 7.80, N 3.19; found (%): C 48.80, H 7.942, N 3.10.

Piperidine 3-(2-isopropyl-1,2-dicarba-*closo*-dodecaboran-1-yl)-3-(4-fluorophenyl)propanoate salt (**32**). From 3-(2-isopropyl-1,2-dicarba-closo-dodecaboran-1-yl)-3-(4-fluorophenyl)propanoic acid 0.52 g (1.49 mmol) and piperidine 0.158 mL (1.6 mmol). Yield—0.51 g (85%); white crystals; mp 142.2–151.6 °C.

FTIR spectrum, ν, cm^−1^: 3000–3200 (NH_2_^+^), 3045 (C-H, ar.), 2865, 2946, 2989 (C-H), 2562, 2600, 2620 (B-H), 1626 (N-H), 1575 (COO^−^, ν_asym), 1507 (C=C, ring modes), 1389 (COO^−^, ν_sym), 1033 (C-N), 1010 (in-plane, C-H), 852 (out-of-plane, C-H).

^1^H NMR (80 MHz, DMSO-*d*_6_): δ = 11.5–4.5 (m, 10H, B-H), 1.16 (d, *J* = 6.7 Hz, 6H, CH(CH_3_)_2_), 1.36 (s, 4H, (-CH_2_-)_2_), 2.45 (d, *J* = 1.8 Hz, 3H, -CH_2_-, CH(CH_3_)_2_), 2.63 (s, 6H, (-CH_2_-)_3_), 3.94 (t, *J* = 7.3 Hz, 1H, C-H), 6.10 (m, 2H, NH_2_^+^), 7.47–6.88 (m, 4H, C_sp2_H).

^13^C NMR (20 MHz, DMSO-*d*_6_): δ = 22.58 (-CH_2_-)_2_, 22.88 (-CH_2_-)_2_, 24.57 (-CH_3_), 24.76 (-CH_3_), 30.58 (C-H), 43.74 (C-H), 44.37 (-CH_2_-), 45.45 (C-H), 89.35 (C_4_, carborane core), 90.63 (C_4_, carborane core), 114.56 (C_sp2_H), 115.61 (C_sp2_H),131.59 (C_sp2_H), 131.97 (C_sp2_H), 137.36 (C_4_, benzylidene), 137. 52 (C_4_, benzylidene), 173.12 (C_4_, -COO^−^).

^11^B NMR (26 MHz, DMSO-*d*_6_): δ = −7.5 (m, 5B), -34.53 (m, 5B).

^19^F NMR (76 MHz, DMSO-*d*_6_): δ = −1130.18 (s, 1F).

Elemental analysis: calculated: for C_19_H_36_B_10_O_2_NF (%): C 52.17, H 8.29, N 3.20; found (%): C 52.84, H 8.456, N 3.56.

Diethylammonium 3-(2-isopropyl-1,2-dicarba-*closo*-dodecaboran-1-yl)-3-(4-fluorophenyl)propanoate salt (**33**). From 3-(2-isopropyl-1,2-dicarba-closo-dodecaboran-1-yl)-3-(4-fluorophenyl)propanoic acid 0.52 g (1.49 mmol) and diethylamine 0.16 mL (1.6 mmol). Yield—0.36 g (62%); white crystals; mp 172.2 °C.

FTIR spectrum, ν, cm^−1^: 3000–3200 (NH_2_^+^), 3042 (C-H, ar.), 2874, 2943, 2990 (C-H), 2565, 2595, 2619 (B-H), 1635 (N-H), 1542 (COO^−^, ν_asym), 1507 (C=C, ring modes), 1389 (COO^−^, ν_sym), 1070 (C-N), 1012 (in-plane, C-H), 815 (out-of-plane, C-H).

^1^H NMR (80 MHz, DMSO-*d*_6_): δ = 1.0–4.0 (m, 10H, B-H), 0.85 (t, *J* = 7.2 Hz, 4H, (-CH_2_-)_2_), 1.13 (d, *J* = 6.7 Hz, 6H, CH(CH_3_)_2_), 2.61–2.38 (m, 6H, (-CH_3_)_2_), 2.68 (m, 2H, -CH_2_-), 3.29 (d, *J* = 6.9 Hz, 1H, CH(CH_3_)_2_), 3.90 (t, *J* = 7.4 Hz, 1H, C-H), 7.30–6.86 (m, 4H, C_sp2_H), 7.68 (s, 2H, NH_2_^+^).

^13^C NMR (20 MHz, DMSO-*d*_6_): δ = 11.67 (-CH_3_)_2_, 24.53 (-CH_3_), 24.73 (-CH_3_), 30.56 (C-H), 39.05 (C-H), 41.42 (-CH_2_-), 44.36 (C-H), 45.40 (-CH_2_-), 89.18 (C_4_, carborane core), 90.60 (C_4_, carborane core), 114.51 (C_sp2_H). 115.57 (C_sp2_H), 131.47 (C_sp2_H), 131.95 (C_sp2_H), 137.33 (C_4_, benzylidene), 137.49 (C_4_, benzylidene), 172.92 (C_4_, -COO^−^).

^11^B NMR (26 MHz, DMSO-*d*_6_): δ = −8.30 (m, 10 B).

^19^F NMR (76 MHz, DMSO-*d*_6_): δ = −113.24 (s, 1F).

Elemental analysis: calculated: for C_18_H_36_B_10_O_2_NF (%): C 50.81, H 8.53, N 3.29; found (%): C 50.40, H 8.408, N 3.04.

Morpholine 3-(2-isopropyl-1,2-dicarba-*closo*-dodecaboran-1-yl)-3-(naphthalene-1-yl)propanoate salt (**34**). From 3-(2-isopropyl-1,2-dicarba-closo-dodecaboran-1-yl)-3-(naphthalene-1-yl)propanoic acid 0.1 g (0.27 mmol) and morpholine 0.023 mL (0.27 mmol). Yield—0.07 g (63%); white crystals; mp 197–207 °C.

FTIR spectrum, ν, cm^−1^: 3000–3200 (NH_2_^+^), 3057 (C-H, ar.), 2700–2984 (C-H), 2547–2656 (B-H), 1633 (N-H), 1557 (COO^−^, ν_asym), 1459 (C=C, ring modes), 1391 (COO^−^, ν_sym), 1370 (-CH_3_), 1235 (C-N), 1111 (C-O-C, ν_asym), 1048 (C-O-C, ν_sym), 742, 781, 793 (out-of-plane, C-H).

^1^H NMR (500 MHz, DMSO-*d*_6_): δ = 1.5–2.6 (m, 10H, B-H), 1.23 (t, *J* = 6.8 Hz, 6H, CH(CH_3_)_2_), 2.64–2.57 (m, 4H, (-CH_2_-)_2_), 2.82 (p, *J* = 6.8 Hz, 1H, CH(CH_3_)_2_), 2.96 (m, 2H, -CH_2_-), 3.39 (m, 6H, (-CH_2_-)_2_, NH_2_^+^), 4.89 (dd, *J* = 11.1, 4.5 Hz, 1H, C-H), 7.28 (benzene), 7.45 (m, *J* = 7.7, 4.1 Hz, 2H, C_sp2_H), 7.53 (m 2H, C_sp2_H), 7.81 (d, *J* = 8.1 Hz, 1H, C_sp2_H), 7.87 (d, 1H, C_sp2_H), 8.07 (d, *J* = 8.6 Hz, 1H, C_sp2_H).

^13^C NMR (126 MHz, DMSO-*d*_6_): δ = 24.79 (-CH_3_), 25.03 (-CH_3_), 30.67 (C-H), 37.87 (C-H), 44.22 (-CH_2_-)_2_, 44.58 (C-H), 65.19 (-CH_2_-)_2_, 89.62 (C_4_, carborane core), 89.97 (C_4_, carborane core), 123.74 (C_sp2_H), 125.83 (C_sp2_H), 126.25 (C_sp2_H), 126.87 (C_sp2_H), 127.34 (C_sp2_H), 128.96 (C_sp2_H), 129.38 (C_sp2_H), 131.89 (C_4_, naphthyl), 133.94 (C_4_, naphthyl), 137.12 (C_4_, naphthyl), 172.53 (C_4_, -COO^−^).

^11^B NMR (160 MHz, DMSO-*d*_6_): δ = −5.37 (m, 5B), −11.46 (m, 5B).

Elemental analysis: calculated: for C_22_H_37_B_10_O_3_N (%): C 56.03, H 7.91, N 2.97; found (%): C 55.77, H 8.069, N 2.94.

Piperidine 3-(2-isopropyl-1,2-dicarba-*closo*-dodecaboran-1-yl)-3-(naphthalene-1-yl)propanoate salt (**35**). From 3-(2-isopropyl-1,2-dicarba-closo-dodecaboran-1-yl)-3-(naphthalene-1-yl)propanoic acid 0.1 g (0.27 mmol) and piperidine 0.026 mL (0.27 mmol). Yield—0.09 g (85%); white crystals; mp 220–235 °C.

FTIR spectrum, ν, cm^−1^: 3000–3200 (NH_2_^+^), 3050 (C-H, ar.), 2745–3000 (C-H), 2570–2642 (B-H), 1627 (N-H), 1544 (COO^−^, ν_asym), 1465 (C=C, ring modes), 1395 (COO^−^, ν_sym), 1379 (-CH_3_), 1070 (C-N), 738, 778, 793 (out-of-plane, C-H).

^1^H NMR (500 MHz, CDCl_3_): δ = 1.4–2.6 (m, 10H, B-H), 0.96 (s, *J* = 3.6 Hz, 6H, (-CH_2_-)_3_), 1.30 (t, *J* = 6.8 Hz, 6H, CH(CH_3_)_2_), 1.75 (s, *J* = 6.5 Hz, 6H, (-CH_2_-)_2_, NH_2_^+^), 2.74 (m, *J* = 6.9 Hz, 2H, -CH_2_-), 2.83 (m, *J* = 15.3, 4.3 Hz, 1H, CH(CH_3_)_2_), 4.90 (dd, *J* = 11.7, 4.3 Hz, 1H, C-H), 7. 25 (s, 1H, C_sp2_H), 7.36 (benzene), 7.44 (m, 2H, C_sp2_H), 7.59 (d, *J* = 7.6 Hz, 1H, C_sp2_H), 7.71 (d, *J* = 8.1 Hz, 1H, C_sp2_H), 7.80 (dd, *J* = 8.0, 1.6 Hz, 1H, C_sp2_H), 8.15 (d, *J* = 8.4 Hz, 1H, C_sp2_H).

^11^B NMR (160 MHz, CDCl_3_): δ = −4.90 (m, 4B), −11.14 (m, 6B).

Elemental analysis: calculated: for C_23_H_39_B_10_O_2_N (%): C 58.83, H 8.37, N 2.98; found (%): C 58.46, H 8.129, N 2.78.

Diethylammonium 3-(2-isopropyl-1,2-dicarba-*closo*-dodecaboran-1-yl)-3-(naphthalene-1-yl)propanoate salt (**36**). From 3-(2-isopropyl-1,2-dicarba-closo-dodecaboran-1-yl)-3-(naphthalene-1-yl)propanoic acid 0.1 g (0.27 mmol) and diethylamine 0.028 mL (0.27 mmol). Yield—0.07 g (68%); white crystals; mp 208–209 °C.

FTIR spectrum, ν, cm^−1^: 3000–3200 (NH_2_^+^), 3041 (C-H, ar.), 2941, 2982 (C-H), 2565–2647 (B-H), 1623 (N-H), 1555 (COO^−^, ν_asym), 1453 (C=C, ring modes), 1387 (COO^−^, ν_sym), 1373 (-CH_3_), 1070 (C-N), 732, 782, 796 (out-of-plane, C-H).

^1^H NMR (500 MHz, DMSO-*d*_6_): δ = 1.5–2.5 (m, 10H, B-H), 0.82 (t, *J* = 7.3 Hz, 6H, (-CH_3_)_2_), 1.27 (dd, *J* = 8.3, 6.6 Hz, 6H, CH(CH_3_)_2_), 2.80 (m, *J* = 15.0, 4.3 Hz, 1H, CH(CH_3_)_2_), 2.90 (m, 2H, -CH_2_-), 3.36 (m, 6H, (-CH_2_-)_2_, NH_2_^+^), 5.02 (dd, *J* = 10.5, 4.4 Hz, 1H, C-H), 7.33 (d, 1H, C_sp2_H), 7.48 (td, *J* = 7.6, 4.3 Hz, 2H, C_sp2_H), 7.55 (m, 1H, C_sp2_H), 7.83 (d, *J* = 8.0 Hz, 1H, C_sp2_H), 7.90 (dd, *J* = 8.4, 1.4 Hz, 1H, C_sp2_H), 8.15 (d, *J* = 8.7 Hz, 1H, C_sp2_H).

^11^B NMR (26 MHz, DMSO-*d*_6_): δ = −6.56 (m, 10B).

Elemental analysis: calculated: for C_22_H_39_B_10_O_2_N (%): C 57.75, H 8.59, N 3.06; found (%): C 56.08, H 8.567, N 2.62.

### 3.7. Synthesis of Carboranyl-Containing 3-Arylpropanoic Acid with Potassium and Sodium Metallic/Hydroxides

In a 50 mL round-bottom flask equipped with a stir bar, (0.4 mmol) of the corresponding isopropyl-*o*-carboranyl containing 3-arylpropanoic acid, (0.4 + 10% mmol) of metal or metal hydroxide were added, along with 10 mL of THF. The reaction mixture was stirred for three days until all the metal/metal hydroxide reacted and precipitated as a white powder. The precipitate was then filtered using a Schott glass filter and washed with THF. Thus, salts of carboranyl-containing 3-arylpropanoate are obtained with yields of at least 60% for all reactions.

Potassium 3-(2-isopropyl-1,2-dicarba-*closo*-dodecaboran-1-yl)-3-phenylpropanoate (**37**). From 3-(2-isopropyl-1,2-dicarba-closo-dodecaboran-1-yl)-3-phenylpropanoic acid 0.13 g (0.4 mmol) and metallic potassium 0.017 g (0.4 + 10% mmol). Yield—0.1 g (70%); white crystals; decomposes when heated. A similar product is obtained by using potassium hydroxide.

FTIR spectrum, ν, cm^−1^: 3200–3600 (O-H, H_2_O), 3050 (C-H, ar.), 2985–2985 (C-H), 2570, 2618 (B-H), 1575 (COO^−^, ν_asym), 1453 (C=C, ring modes), 1390 (COO^−^, ν_sym), 1082 (in-plane, C-H), 705 (out-of-plane, C-H).

^1^H NMR (500 MHz, DMSO-*d*_6_): δ = 1.32–2.41 (m, 10H, B-H), 1.23 (dd, *J* = 12.0, 5.5 Hz, 6H, CH(CH_3_)_2_), 2.48–2.44 (m, 2H, -CH_2_-), 2.97 (td, *J* = 13.5, 6.7 Hz, 1H, CH(CH_3_)_2_), 3.37 (s, H_2_O), 4.05 (m, 1H, C-H), 7.23–7.18 (m, 2H, C_sp2_H), 7.35–7.26 (m, 3H, C_sp2_H).

^13^C NMR (20 MHz, D_2_O): δ = 24.23 (-CH_3_), 24.51 (-CH_3_), 25.29 (C-H), 44.67 (C-H), 67.98 (-CH_2_-), 87.45 (C_4_, carborane core), 90.25 (C_4_, carborane core), 128.49 (C_sp2_H, 5 atoms), 140.19 (C_4_, phenyl), 178.35 (C_4_, -COO^−^).

^11^B NMR (26 MHz, D_2_O): δ = −5.24 (m, 10B).

Elemental analysis: calculated: for C_14_H_25_B_10_O_2_K (%): C 45.15, H 5.64; found (%): C 45.31, H 5.754.

Sodium 3-(2-isopropyl-1,2-dicarba-*closo*-dodecaboran-1-yl)-3-phenylpropanoate (**38**). From 3-(2-isopropyl-1,2-dicarba-closo-dodecaboran-1-yl)-3-phenylpropanoic acid 0.13 g (0.4 mmol) and metallic sodium 0.010 g (0.4 + 10% mmol). Yield—0.12 g (89%); white crystals; decomposes when heated. A similar product is obtained by using sodium hydroxide.

FTIR spectrum, ν, cm^−1^: 3200–3600 (O-H, H_2_O), 3050 (C-H, ar.), 2850–2983 (C-H), 2573, 2616 (B-H), 1572 (COO^−^, ν_asym), 1453 (C=C, ring modes), 1394 (COO^−^, ν_sym), 1084 (in-plane, C-H), 704 (out-of-plane, C-H).

^1^H NMR (500 MHz, DMSO-*d*_6_): δ = 1.32–2.41 (m, 10H, B-H), 1.26–1.17 (m, 6H, CH(CH_3_)_2_), 2.62–2.53 (m, 2H, -CH_2_-), 2.91 (dt, *J* = 13.5, 6.8 Hz, 1H, CH(CH_3_)_2_), 3.40 (s, H_2_O), 4.06 (dd, *J* = 9.8, 4.2 Hz, 1H, C-H), 7.26–7.21 (m, 2H, C_sp2_H), 7.34–7.27 (m, 3H, C_sp2_H).

^11^B NMR (26 MHz, CDCl_3_): δ = −6.34 (m, 10B).

Elemental analysis: calculated: for C_14_H_25_B_10_O_2_Na (%): C 47.17, H 7.07; found (%): C 47.51, H 7.477.

Potassium 3-(2-isopropyl-1,2-dicarba-*closo*-dodecaboran-1-yl)-3-(4-fluorophenyl)propanoate (**39**). From 3-(2-isopropyl-1,2-dicarba-closo-dodecaboran-1-yl)-3-(4-fluorophenyl)propanoic acid 0.14 g (0.4 mmol) and metallic potassium 0.017 g (0.4 + 10% mmol). Yield—0.09 g (63%); white crystals; decomposes when heated. A similar product is obtained by using potassium hydroxide.

FTIR spectrum, ν, cm^−1^: 3200–3600 (O-H, H_2_O), 3079 (C-H, ar.), 2880, 2943, 2985 (C-H), 2571, 2588 (B-H), 1573 (COO^−^, ν_asym), 1508 (C=C, ring modes), 1391 (COO^−^, ν_sym), 1057 (C-O), 1013 (in-plane, C-H), 746, 849 (out-of-plane, C-H).

^1^H NMR (80 MHz, DMSO-*d*_6_): δ = 1.0–4.0 (m, 10H, B-H), 1.17 (d, *J* = 6.5 Hz, 6H, CH(CH_3_)_2_), 1.71 (s, 1H, CH(CH_3_)_2_), 2.79 (d, *J* = 6.7 Hz, 2H, -CH_2_-), 4.00 (m, 1H, C-H). 7.13 (m, 3H, C_sp2_H), 3.50 (s, H_2_O), 7.39–6.82 (m, 4H, C_sp2_H).

^13^C NMR (20 MHz, DMSO-*d*_6_): δ = 24.57 (-CH_3_), 24.80 (-CH_3_), 30.49 (CH(CH_3_)_2_), 44.78 (C-H), 46.61 (-CH_2_-), 90.06 (C_4_, carborane core), 90.75 (C_4_, carborane core), 114.51 (C_sp2_H), 115.53 (C_sp2_H), 131.59 (C_sp2_H), 131.99 (C_sp2_H), 137.99 (C_4_, benzylidene), 138.14 (C_4_, benzylidene), 172.31 (C_4_, -COO^−^).

^11^B NMR (26 MHz, CDCl_3_): δ = −7.32 (m, 10B).

^19^F NMR (76 MHz, CDCl_3_): δ = −113.43 (s, 1F).

Elemental analysis: calculated: for C_14_H_24_B_10_O_2_KF (%): C 43.05, H 6.20; found (%): C 42.57, H 5.425.

Sodium 3-(2-isopropyl-1,2-dicarba-*closo*-dodecaboran-1-yl)-3-(4-fluorophenyl)propanoate (**40**). From 3-(2-isopropyl-1,2-dicarba-closo-dodecaboran-1-yl)-3-(4-fluorophenyl)propanoic acid 0.14 g (0.4 mmol) and metallic sodium 0.010 g (0.4 + 10% mmol). Yield—0.1 g (70%); white crystals; decomposes when heated. A similar product is obtained by using sodium hydroxide.

FTIR spectrum, ν, cm^−1^: 2976 (C-H, ar.), 2869, 2929 (C-H), 2563, 2587, 2607 (B-H), 1564 (COO^−^, ν_asym), 1507 (C=C, ring modes), 1402 (COO^−^, ν_sym), 1054 (C-O), 1012 (in-plane, C-H), 734, 841 (out-of-plane, C-H).

^1^H NMR (80 MHz, DMSO-*d*_6_): δ = 1.0–4.0 (m, 10H, B-H), 1.18 (d, J = 6.7 Hz, 6H, CH(CH_3_)_2_), 2.00–1.43 (m, 2H, -CH_2_-), 3.03–2.70 (m, 1H, CH(CH_3_)_2_), 3.78–3.26 (m, H2O), 4.07 (t, J = 6.8 Hz, 1H, C-H), 7.46–6.82 (m, 4H, C_sp2_H).

^11^B NMR (26 MHz, DMSO-*d*_6_): δ = −8.62 (m, 10B)

Elemental analysis: calculated: for C_14_H_24_B_10_O_2_NaF (%): C 44.91, H 6.46; found (%): C 44.96, H 6.591.

Potassium 3-(2-isopropyl-1,2-dicarba-*closo*-dodecaboran-1-yl)-3-(naphthalene-1-yl)propanoate (**41**). From 3-(2-isopropyl-1,2-dicarba-closo-dodecaboran-1-yl)-3-(naphthalene-1-yl)propanoic acid 0.15 g (0.4 mmol) and metallic potassium 0.017 g (0.4 + 10% mmol). Yield—0.13 g (81%); white crystals; decomposes when heated. A similar product is obtained by using potassium hydroxide.

FTIR spectrum, ν, cm^−1^: 3070 (C-H, ar.), 2868, 2932, 2978, 2998 (C-H), 2556, 2594, 2619 (B-H), 1574 (COO^−^, ν_asym), 1459 (C=C, ring modes), 1385 (COO^−^, ν_sym), 1053 (C-O), 1015 (in-plane, C-H), 737, 787, 797 (out-of-plane, C-H).

^1^H NMR (80 MHz, DMSO-*d*_6_): δ = 1.0–4.0 (m, 10H, B-H), 1.25 (d, *J* = 6.6 Hz, 6H, CH(CH_3_)_2_), 1.82–1.57 (THF), 2.63 (d, *J* = 6.8 Hz, 2H, -CH_2_-), 2.99 (q, *J* = 6.7 Hz, 1H, CH(CH_3_)_2_), 3.46 (m, H_2_O), 5.13 (t, *J* = 6.8 Hz, 1H, C-H), 7.61–7.21 (m, 4H, C_sp2_H), 7.99–7.62 (m, 2H, C_sp2_H), 8.17 (d, *J* = 7.3 Hz, 1H, C_sp2_H).

^11^B NMR (26 MHz, DMSO-*d*_6_): δ = −3.05 (5B), −8.21 (5B).

Elemental analysis: calculated: for C_18_H_27_B_10_O_2_K (%): C 51.15, H 6.44; found (%): C 52.70, H 7.298.

Sodium 3-(2-isopropyl-1,2-dicarba-*closo*-dodecaboran-1-yl)-3-(naphthalene-1-yl)propanoate (**42**). From 3-(2-isopropyl-1,2-dicarba-closo-dodecaboran-1-yl)-3-(naphthalene-1-yl)propanoic acid 0.15 g (0.4 mmol) and metallic sodium 0.010 g (0.4 + 10% mmol). Yield—0.09 g (60%); white crystals; decomposes when heated. A similar product is obtained by using sodium hydroxide.

FTIR spectrum, ν, cm^−1^: 3065 (C-H, ar.), 2874, 2940, 2978 (C-H), 2567, 2610 (B-H), 1571 (COO^−^, ν_asym), 1460 (C=C, ring modes), 1395 (COO^−^, ν_sym), 1057 (C-O), 1014 (in-plane, C-H), 731, 781, 794 (out-of-plane, C-H).

^1^H NMR (500 MHz, DMSO-*d*_6_): δ = 1.8–2.5 (m, 10H, B-H), 1.28 (dd, *J =* 12.7, 6.7 Hz, 6H, CH(CH_3_)_2_), 1.72 (m, THF), 2.80–2.62 (m, 2H, -CH_2_-), 3.02 (m, *J* = 7.0 Hz, 1H, CH(CH_3_)_2_), 3.32 (s, H_2_O), 3.57 (s,THF), 5.13 (dd, *J* = 10.1, 4.0 Hz, 1H, C-H), 7.47 (m, *J* = 7.5, 3.4 Hz, 2H, C_sp2_H), 7.53 (m, *J* = 7.8 Hz, 2H, C_sp2_H), 7.80 (d, *J* = 8.0 Hz, 1H, C_sp2_H), 7.88 (d, *J* = 8.1 Hz, 1H, C_sp2_H), 8.19 (d, *J* = 8.6 Hz, 1H, C_sp2_H).

^13^C NMR (126 MHz, DMSO-*d*_6_): δ = 24.66 (-CH_3_), 24.98 (-CH_3_), 30.27 (C-H), 47.95 (C-H), 67.56 (-CH_2_-), 90.02 (C_4_, carborane core), 91.53 (C_4_, carborane core), 124.45 (C_sp2_H), 125.80 (C_sp2_H), 125.95 (C_sp2_H), 126.30 (C_sp2_H), 127.43 (C_sp2_H), 128.26 (C_sp2_H), 129.11 (C_sp2_H), 132.21 (C_4_, naphthyl), 133.93 (C_4_, naphthyl), 138.97 (C_4_, naphthyl), 172.46 (C_4_, -COO^−^).

^11^B NMR (160 MHz, DMSO-*d*_6_): δ = −6.13 (m, 5B), −12.01 (m, 5B).

Elemental analysis: calculated: for C_18_H_27_B_10_O_2_Na (%): C 53.17, H 6.70; found (%): C 53.68, H 7.288.

### 3.8. Cytotoxic Studies

The following cell lines were used in this study: neonatal human dermal fibroblasts (HDFn) (C0045C, Thermo Fisher Scientific Inc., Waltham, MA, USA), and human breast adenocarcinoma cells (MCF-7) (HTB-22, ATCC, Manassas, VA, USA).

HDFn and MCF-7 cells were cultured in complete DMEM/F12 medium containing 4.5 g/L glucose, 10% fetal bovine serum (FBS), 100 U/mL penicillin, and 100 µg/mL streptomycin at 37 °C in a 5% CO_2_ atmosphere. Cells were passaged using recombinant trypsin TrypLE Express every 5–6 days. The culture medium was replaced every 2 days. Cell counting was performed using a BioRad TC20 (BioRad, Hercules, CA, USA) automated cell counter.

The cytotoxicity of the compound was evaluated using the MTT assay, following the manufacturer’s protocol (https://www.abcam.com/kits/mtt-assay-protocol (accessed on 2 June 2025)). The MTT assay is a colorimetric method used to assess cellular metabolic activity as an indicator of cell viability and compound cytotoxicity. It is based on the reduction in the yellow tetrazolium dye to purple formazan crystals by NAD(P)H-dependent cellular oxidoreductase enzymes.

MCF-7 and HDFn were seeded 18 h before compound exposure into 96-well plates at a density of 10^4^ cells per well in triplicate. The tested compound was prepared in serum- and antibiotic-free DMEM/F12 and α-MEM at a stock concentration of 100 mg/mL (10% solution), corresponding to 281.69 mM, and sterilized via filtration through a 0.22 µm polyethersulfone membrane filter.

Studied compound was added to the cell monolayers at a volume of 100 µL per well with fetal bovine serum added to achieve a final concentration of 10%. Untreated cells served as the control group, and blank wells without cells were used as blanks. After 24 h of incubation, the medium was removed and replaced with fresh medium containing 10% MTT reagent (5 mg/mL), followed by 4 h of incubation. After formazan crystal formation, the medium was replaced with DMSO to dissolve the crystals. Optical density was measured at 580 nm using a BioRad spectrophotometer (BioRad, Hercules, CA, USA). The data were analyzed using GraphPad Prism 8 (version 8.0.2).

## 4. Conclusions

As part of this study, a series of carborane-containing β-arylaliphatic acids were synthesized, including 3-(2-isopropyl-1,2-dicarba-closo-dodecaboran-1-yl)-3-phenylpropanoic acid, 3-(2-isopropyl-1,2-dicarba-closo-dodecaboran-1-yl)-3-(naphthalene-1-yl)propanoic acid, and 3-(2-isopropyl-1,2-dicarba-closo-dodecaboran-1-yl)-3-(4-fluorophenyl)propanoic acid. These compounds were subsequently reacted with various primary and secondary amines, as well as with alkali metals and their hydroxides, to form the corresponding salts. Comprehensive characterization of all synthesized compounds was performed using elemental analysis, FTIR spectroscopy, 1H and 13C NMR spectroscopy, and mass spectrometry (LC-MS).

The analytical data confirmed that reactions between the carborane-containing 3-arylpropanoic acid derivatives and primary or secondary amines yielded ammonium carboxylate salts, while reactions with alkali metals or their hydroxides led to the formation of corresponding metal carboxylate salts. Cytotoxicity studies were performed for the water-soluble compound potassium 2-isopropyl-ortho-carboranyl-3-phenylpropanoate (compound 37). This compound does not induce a significant toxic effect on the viability of MCF-7 and HDFn cells at concentrations of 0.06776 mM and 0.05427 mM, respectively. These findings demonstrate that simple alkali metal salts of boron-rich carboranyl acids can serve as promising candidates for further development of boron-based therapeutic systems.

## Figures and Tables

**Table 1:** Comparison of yields of products of the synthesis of carboranyl-containing 3-arylpropanoic acid with primary amines.

pK_a_ of Primary Amine	Primary Amine	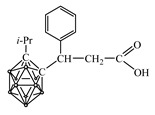 Yield, %	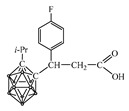 Yield, %	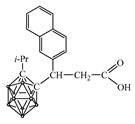 Yield, %
10.6	Methylamine	82	66	86
10.6	Butylamine	90	75	88
10.6	Cyclohexylamine	75	80	83
10.4	Tert-butylamine	66	88	68
10.1	Ethylenediamine	54	44	93
9.5	Allylamine	14	78	75

**Table 2:** Comparison of yields of products of the synthesis of carboranyl-containing 3-arylpropanoic acid with secondary amines.

pK_a_ of Secondary Amine	Secondary Amine	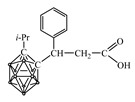 Yield, %	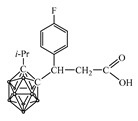 Yield, %	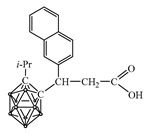 Yield, %
11.2	Piperidine	78	85	85
10.98	Diethylamine	54	62	68
8.36	Morpholine	90	85	63

## Data Availability

The original contributions presented in this study are included in the Supplementary Material. Further inquiries can be directed to the authors.

## Supplementary Materials

The following supporting information can be downloaded at: https://www.mdpi.com/article/10.3390/molecules30153250/s1, File S1: NMR, FTIR spectra, HPLC.
