# Supplementary material for: Synthesis of Carboranyl-Containing β-Arylaliphatic Acids for Potential Application in BNCT
Source: Molecules. 2025 Aug 2;30(15):3250. doi: 10.3390/molecules30153250 (PMC12348761; doi:10.3390/molecules30153250)
Supplement: Supplementary file 1 [file molecules-30-03250-s001.zip › molecules-3775769-supplementary.pdf]

Supplementary materials

Synthesis of carboranyl-containing derivatives of β-arylaliphatic acids for potential application in BNCT

Lana I. Lissovskaya[*1,2] and Ilya V. Korolkov[**1,2]

[1]The Institute of Nuclear Physics, Ibragimov Str. 1, 050032 Almaty, Kazakhstan

[2]L.N. Gumilyov Eurasian National University, Satbaev str. 5, 010008, Astana, Kazakhstan

*Correspondence: i.korolkov@inp.kz, l.lissovskaya@inp.kz

NMR spectra

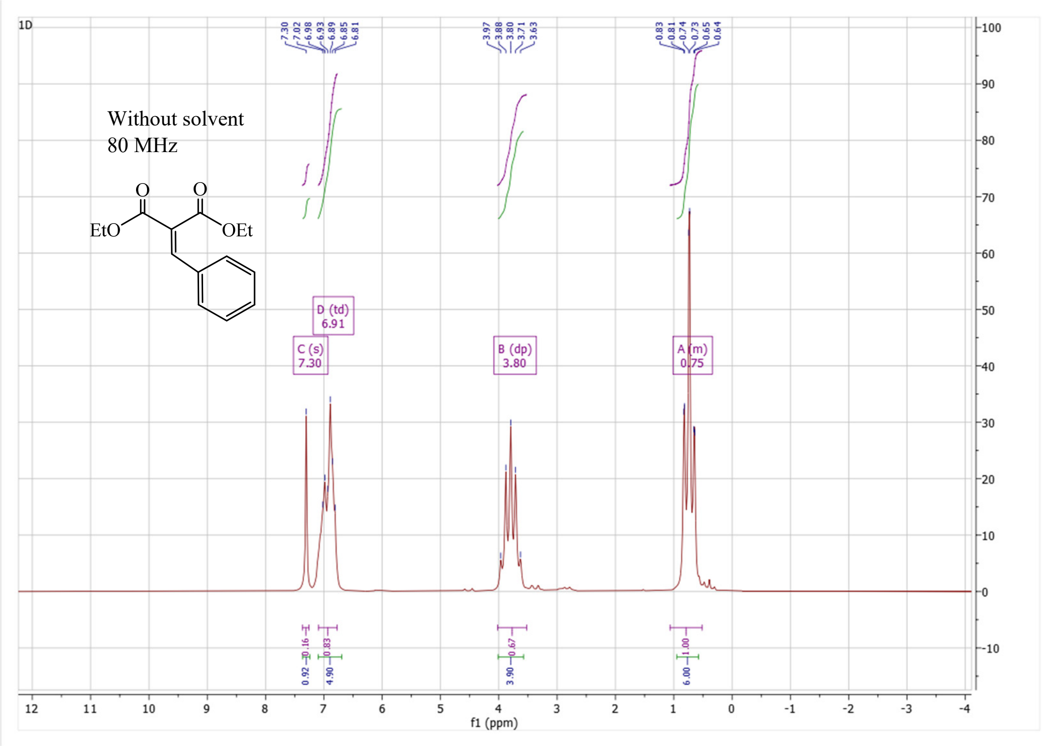

^{1}H NMR spectra of Diethyl 2-benzylidenemalonate (1)

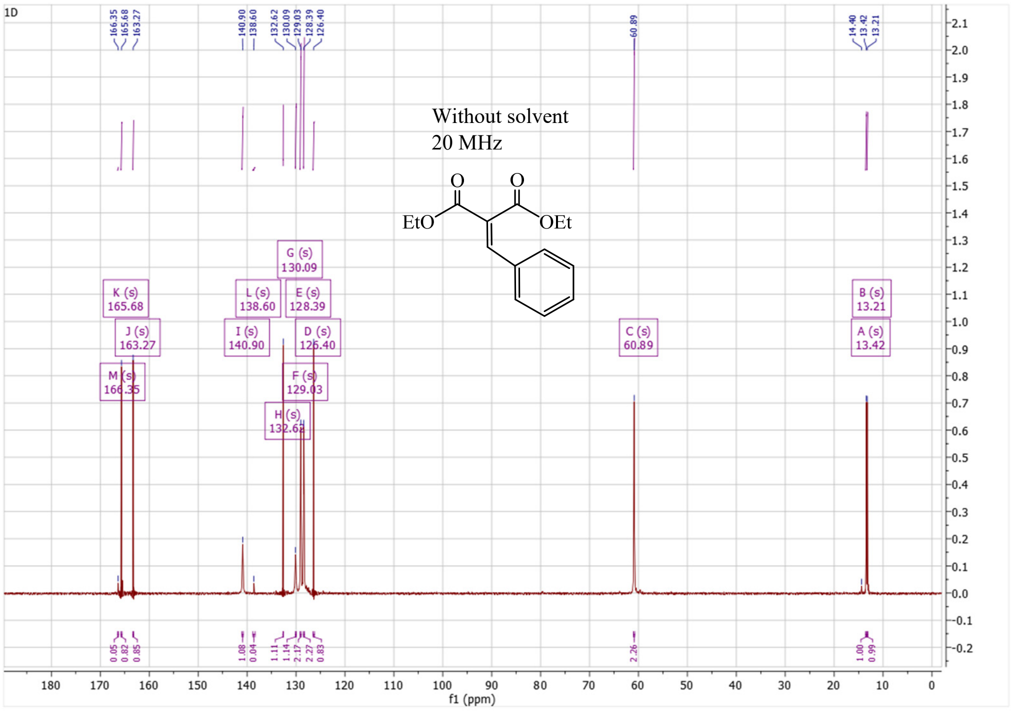

^{13}C NMR spectra of Diethyl 2-benzylidenemalonate (1)

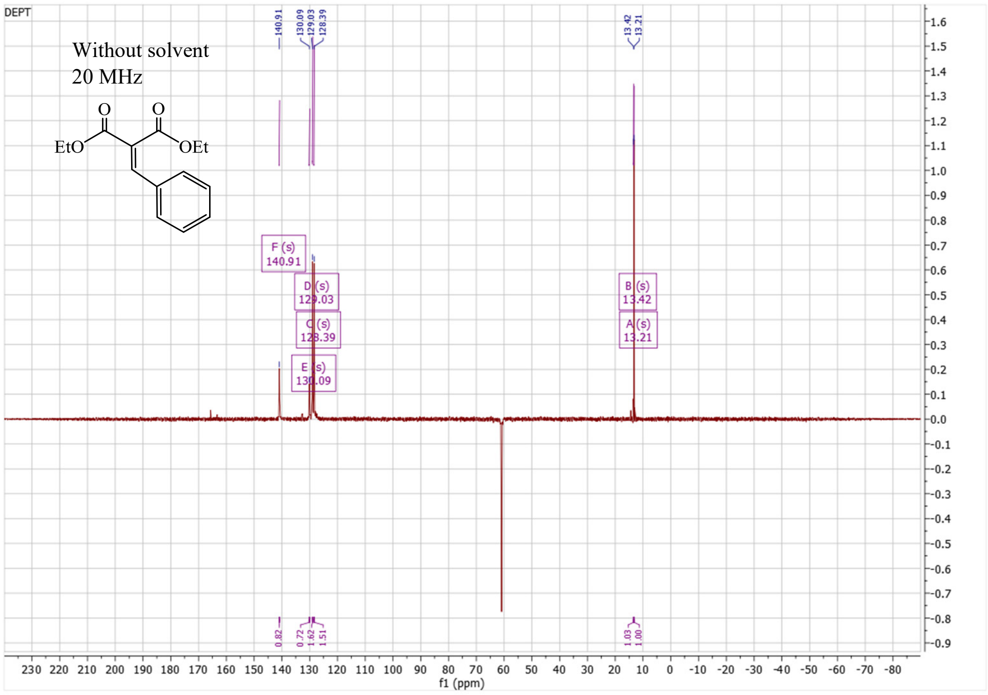

DEPT-135 NMR spectra of Diethyl 2-benzylidenemalonate (1)

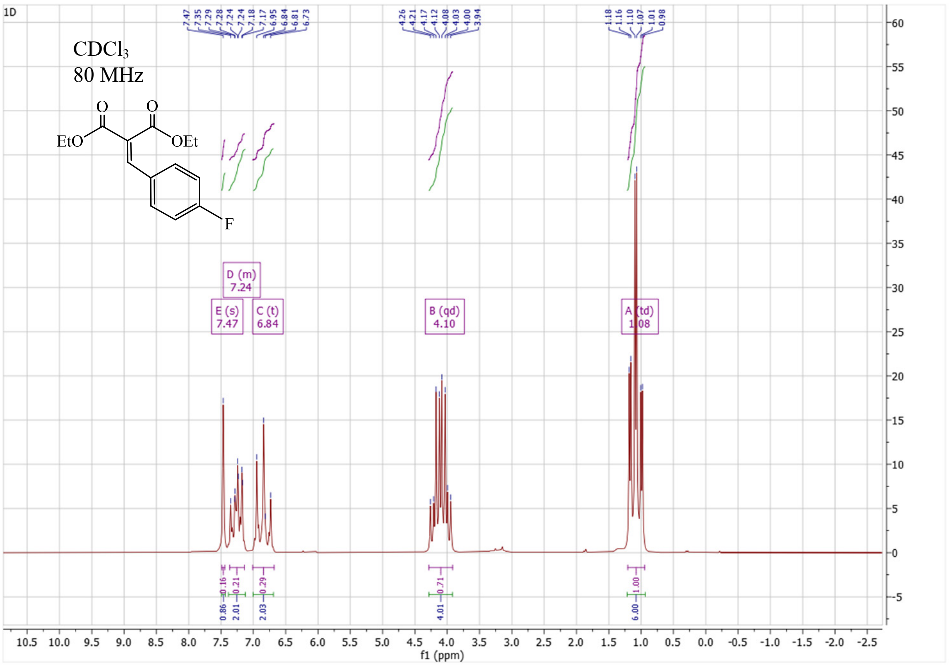

^{1}H NMR spectra of Diethyl 2-(4-fluorobenzylidene)malonate (2)

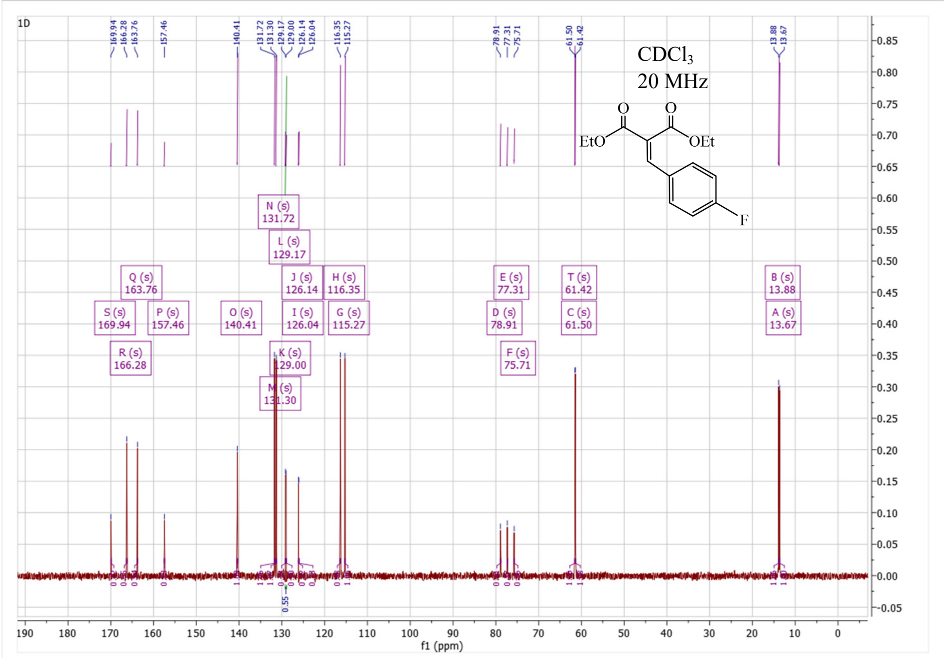

^{13}C NMR spectra of Diethyl 2-(4-fluorobenzylidene)malonate (2)

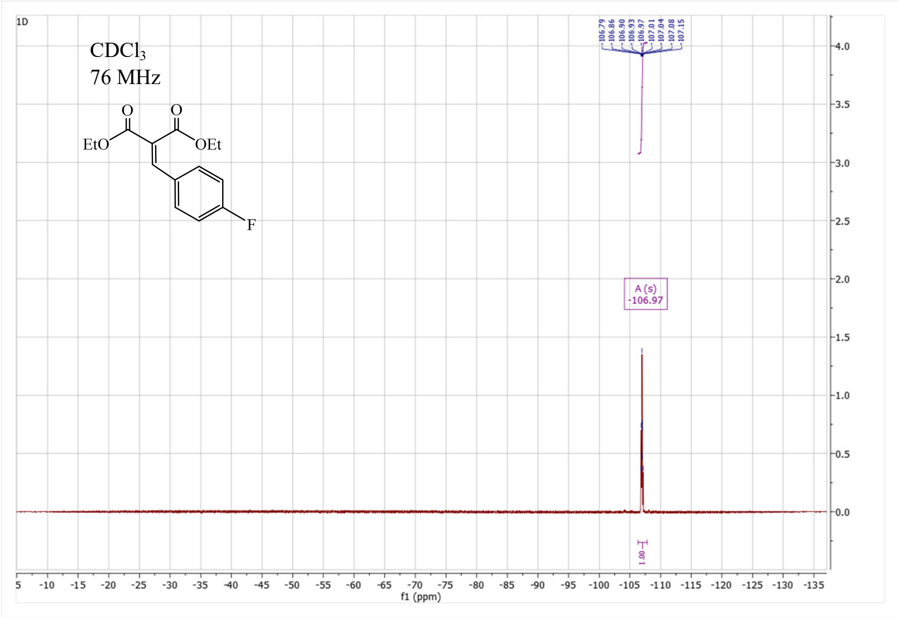

^{19}F NMR spectra of Diethyl 2-(4-fluorobenzylidene)malonate (2)

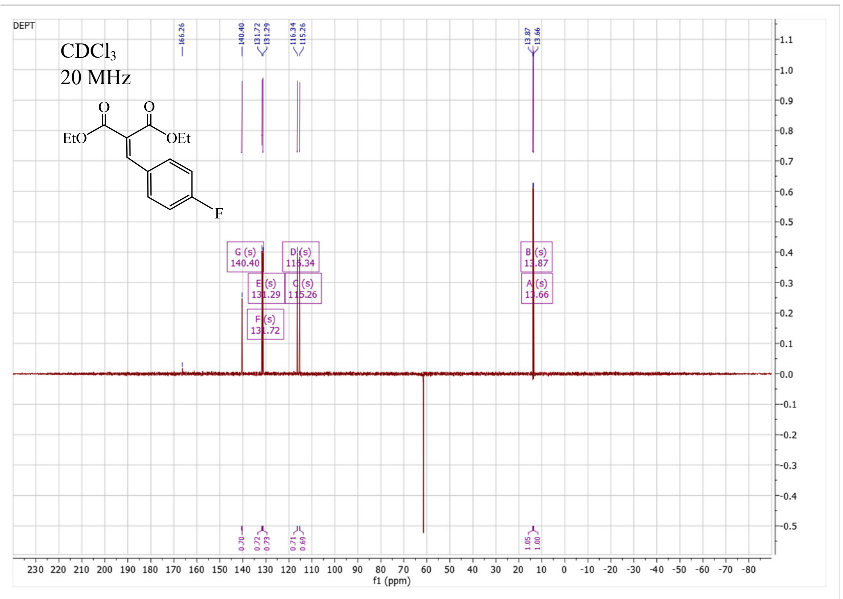

DEPT-135 NMR spectra of Diethyl 2-(4-fluorobenzylidene)malonate (2)

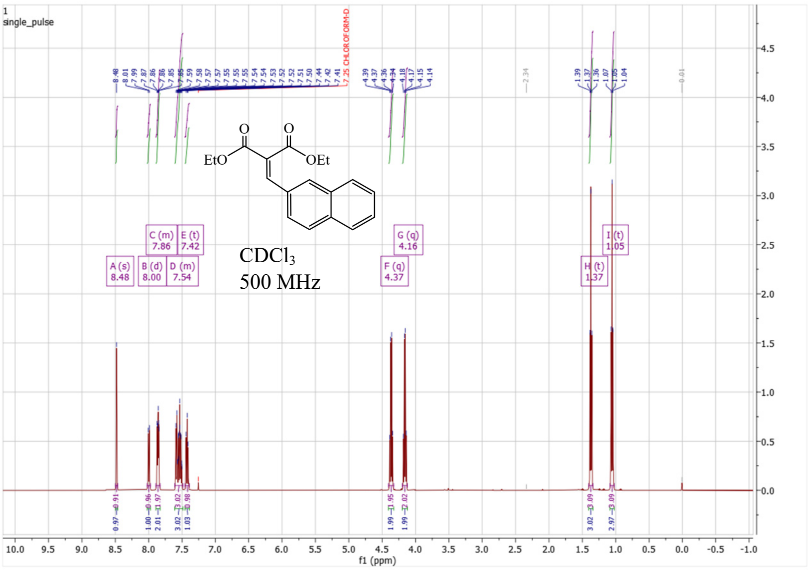

^{1}H NMR spectra of Diethyl 2-(naphthalen-1-yl)malonate (3)

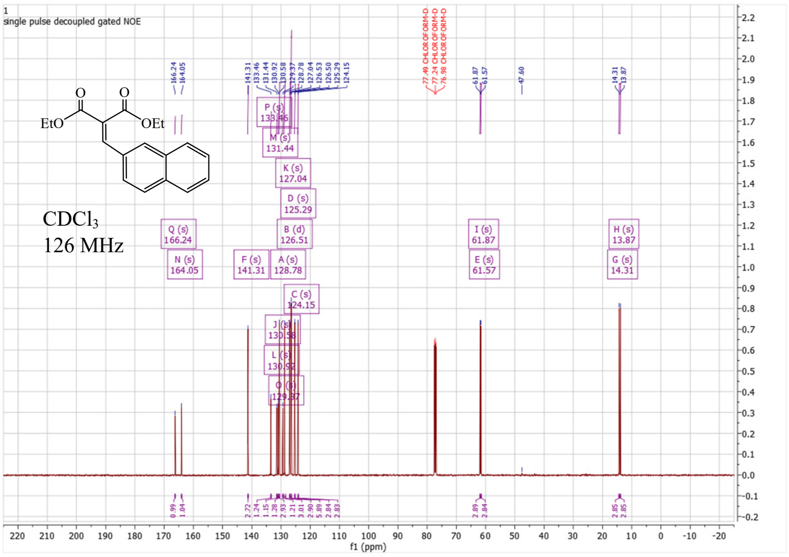

^{13}C NMR spectra of Diethyl 2-(naphthalen-1-yl)malonate (3)

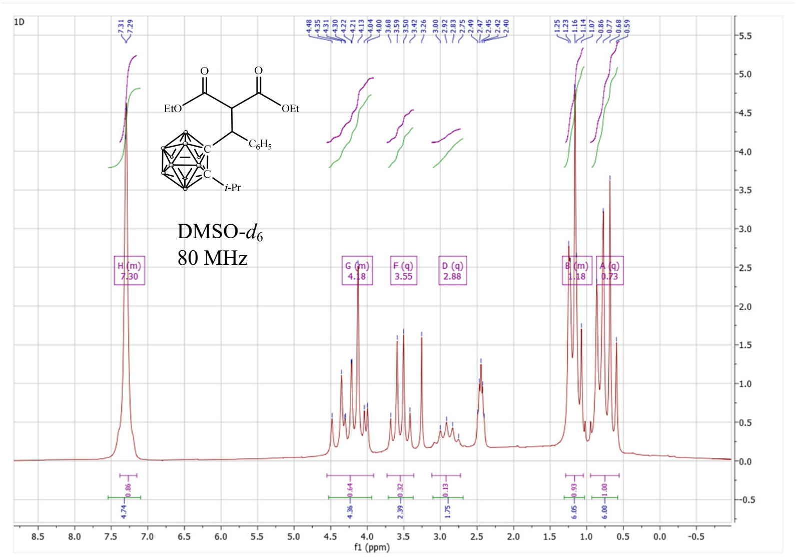

^{1}H NMR spectra of Diethyl 2-[4-(1-isopropyl-closo-1,2-dicarba-closo-dodecaboran-1-yl)benzyl]propanedioate (4)

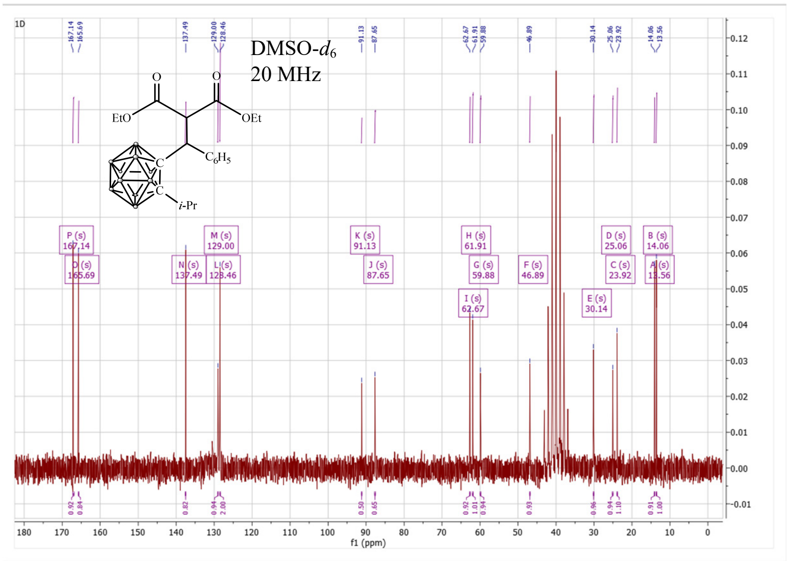

^{13}C NMR spectra of Diethyl 2-[4-(1-isopropyl-closo-1,2-dicarba-closo-dodecaboran-1-yl)benzyl]propanedioate (4)

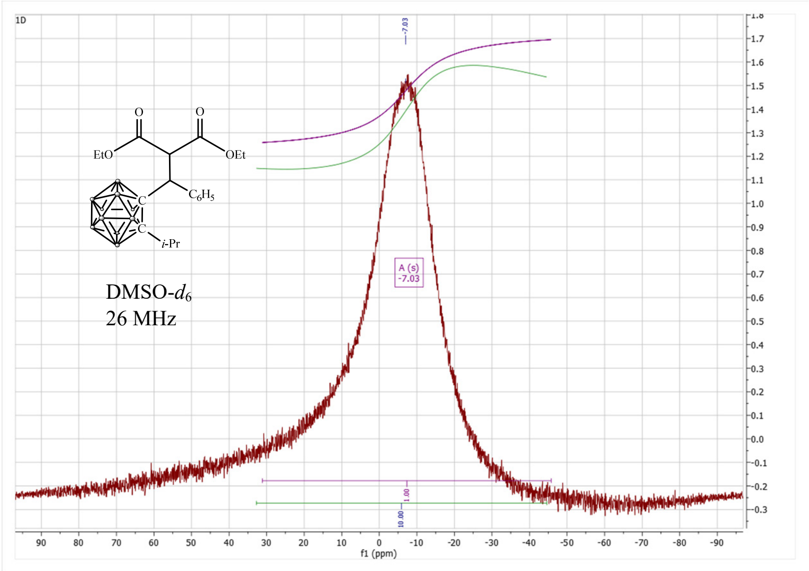

^{11}B NMR spectra of Diethyl 2-[4-(1-isopropyl-closo-1,2-dicarba-closo-dodecaboran-1-yl)benzyl]propanedioate (4)

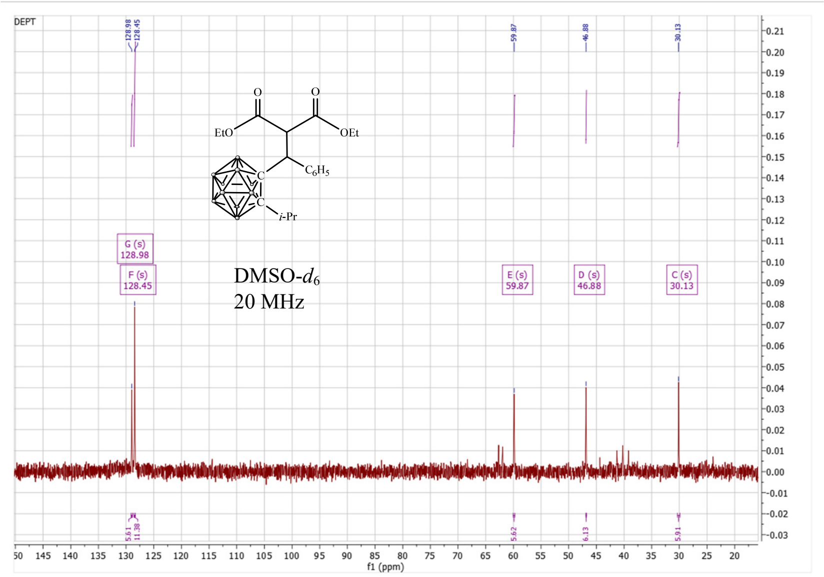

DEPT-90 NMR spectra of Diethyl 2-[4-(1-isopropyl-closo-1,2-dicarba-closo-dodecaboran-1-yl)benzyl]propanedioate (4)

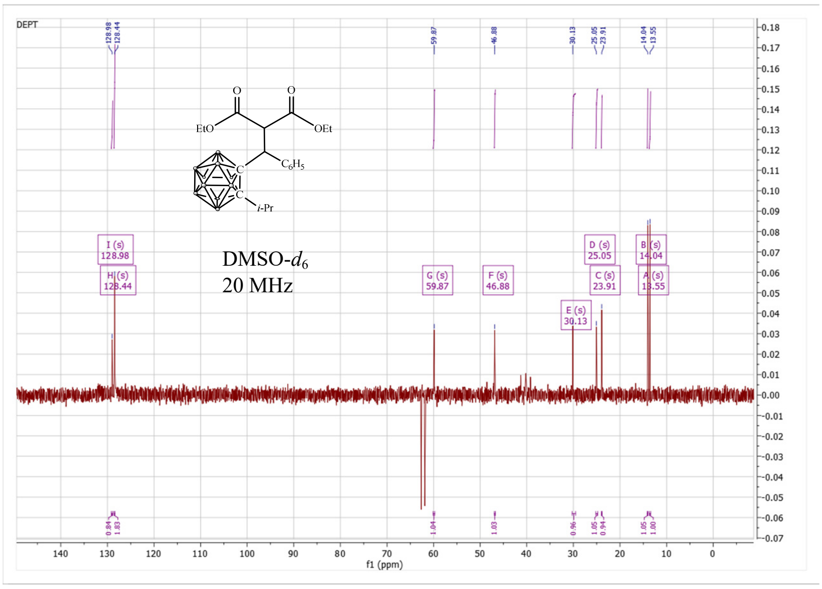

DEPT-135 NMR spectra of Diethyl 2-[4-(1-isopropyl-closo-1,2-dicarba-closo-dodecaboran-1-yl)benzyl]propanedioate (4)

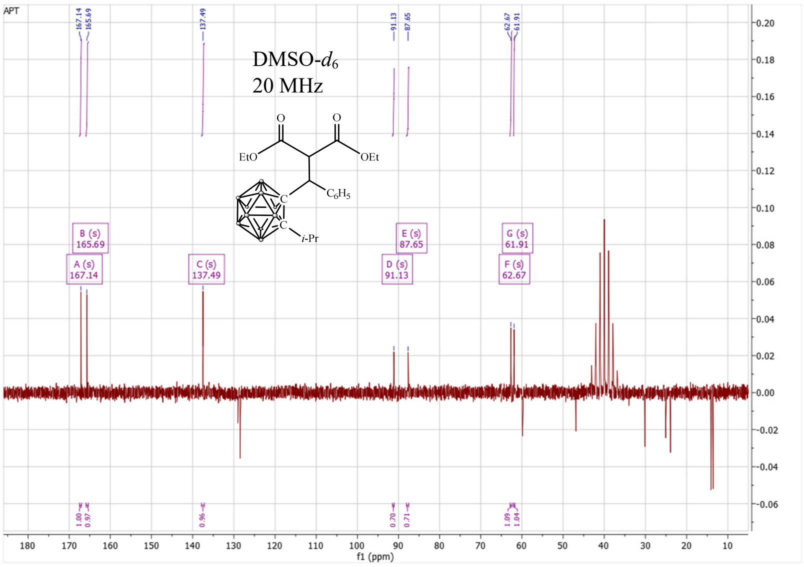

APT-WALTZ NMR spectra of Diethyl 2-[4-(1-isopropyl-closo-1,2-dicarba-closo-dodecaboran-1-yl)benzyl]propanedioate (4)

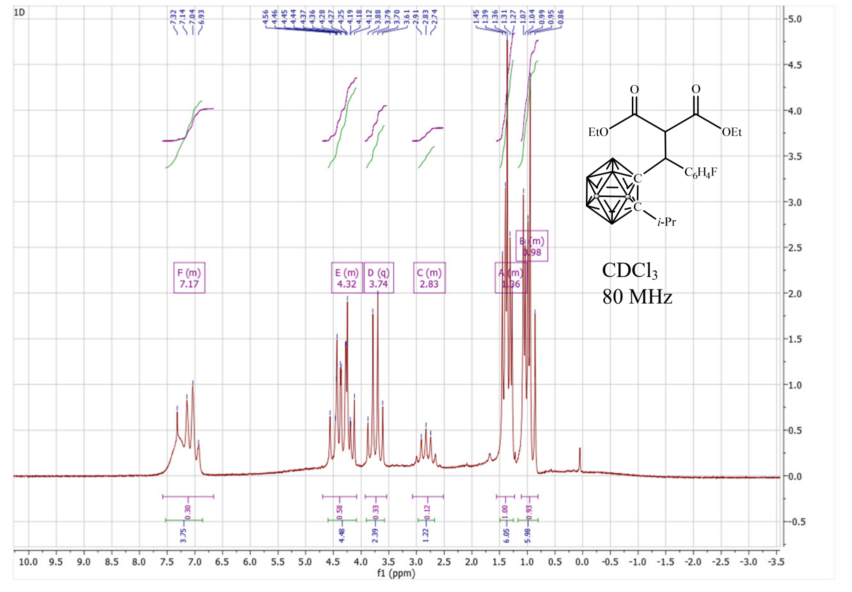

^{1}H NMR spectra of Diethyl 2-[4-fluoro-4-(1-isopropyl-closo-1,2-dicarba-closo-dodecaboran-1-yl)benzyl]propanedioate (5)

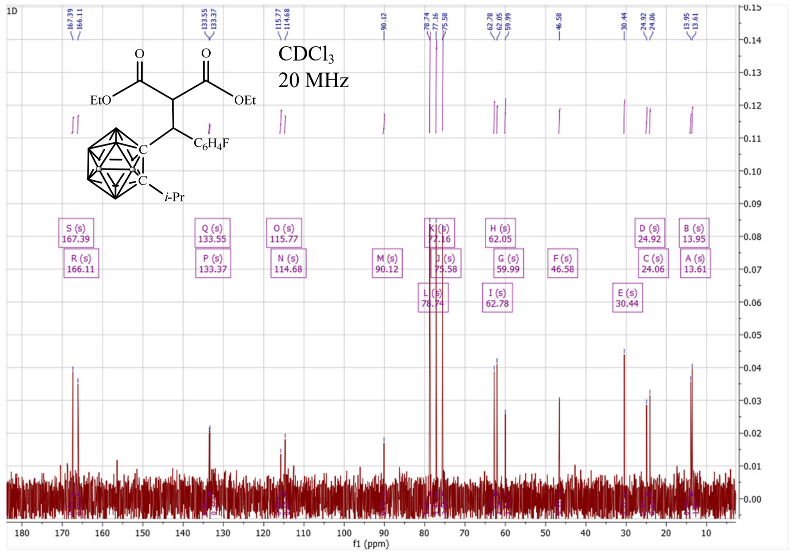

^{13}C NMR spectra of Diethyl 2-[4-fluoro-4-(1-isopropyl-closo-1,2-dicarba-closo-dodecaboran-1-yl)benzyl]propanedioate (5)

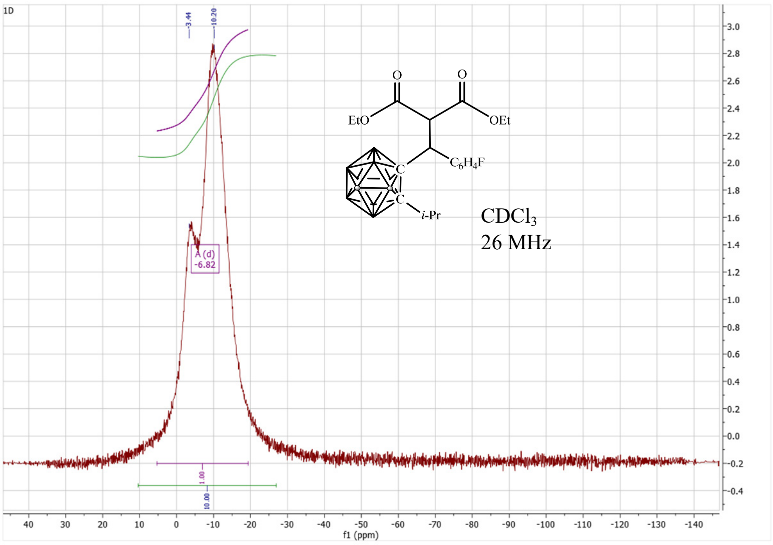

^{11}B NMR spectra of Diethyl 2-[4-fluoro-4-(1-isopropyl-closo-1,2-dicarba-closo-dodecaboran-1-yl)benzyl]propanedioate (5)

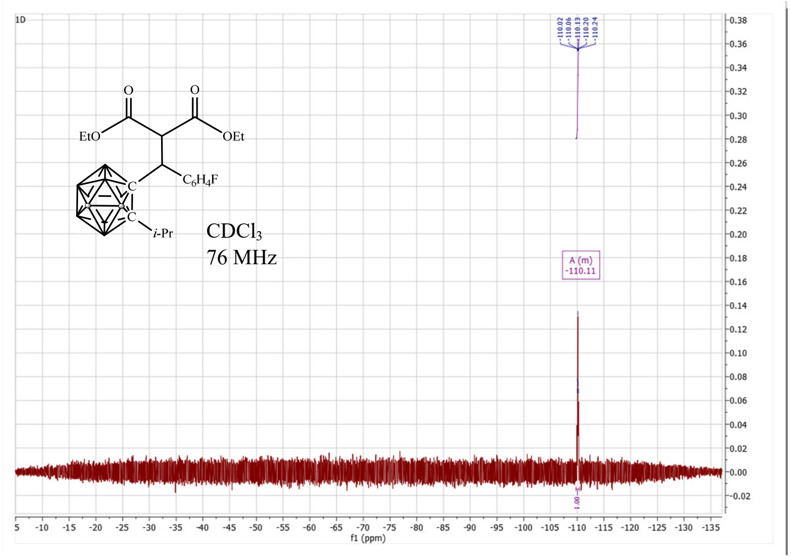

^{19}F NMR spectra of Diethyl 2-[4-fluoro-4-(1-isopropyl-closo-1,2-dicarba-closo-dodecaboran-1-yl)benzyl]propanedioate (5)

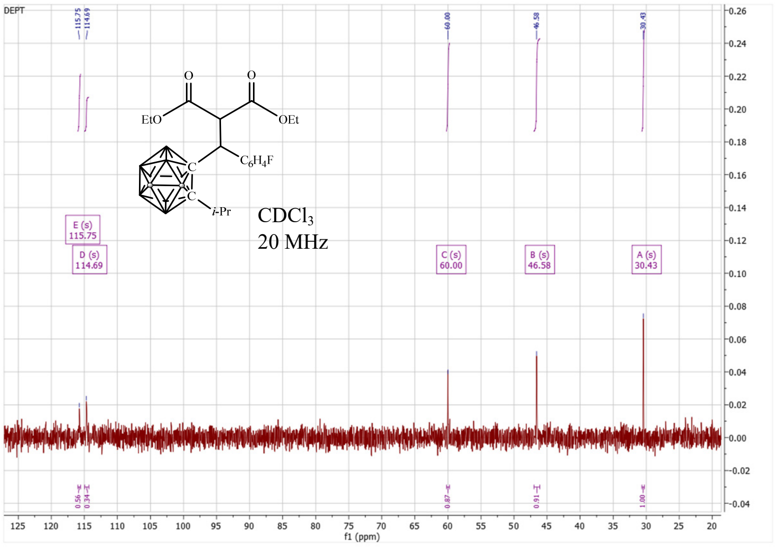

DEPT-90 NMR spectra of Diethyl 2-[4-fluoro-4-(1-isopropyl-closo-1,2-dicarba-closo-dodecaboran-1-yl)benzyl]propanedioate (5)

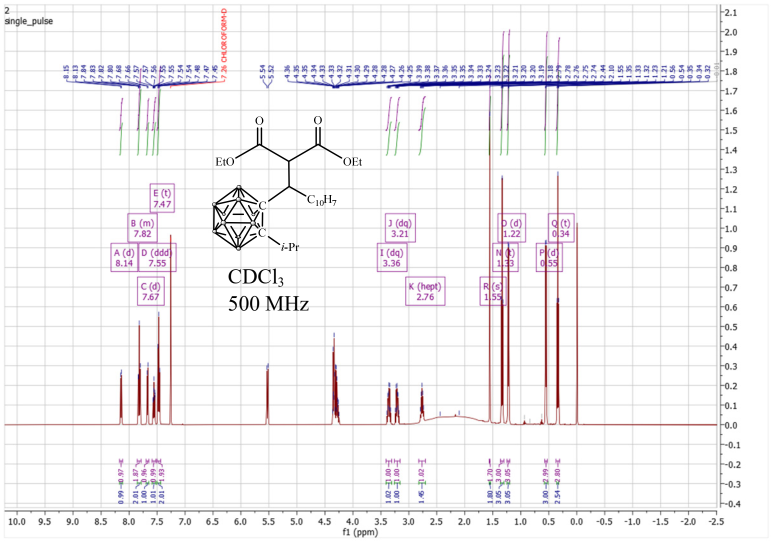

^1H NMR spectra of Diethyl 2-[(1-isopropyl-closo-1,2-dicarba-closo-dodecaboran-1-yl)naphthalen-1-yl]propanedioate (6)

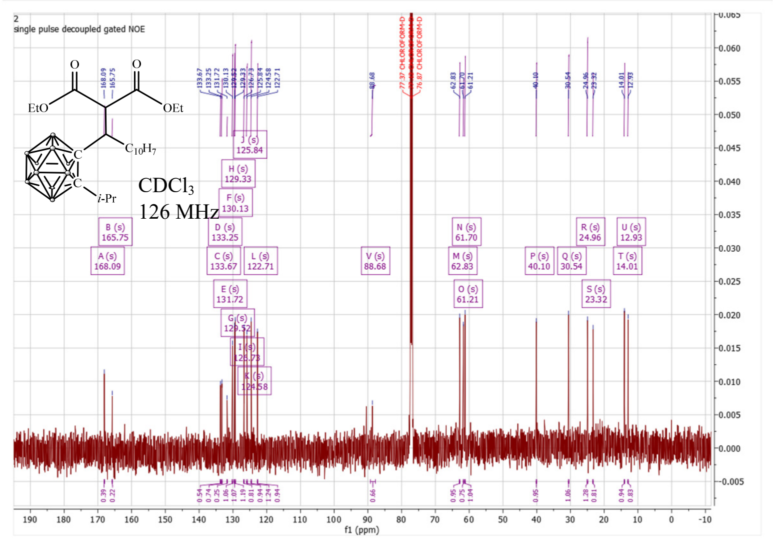

^{13}C NMR spectra of Diethyl 2-[(1-isopropyl-closo-1,2-dicarba-closo-dodecaboran-1-yl)naphthalen-1-yl]propanedioate (6)

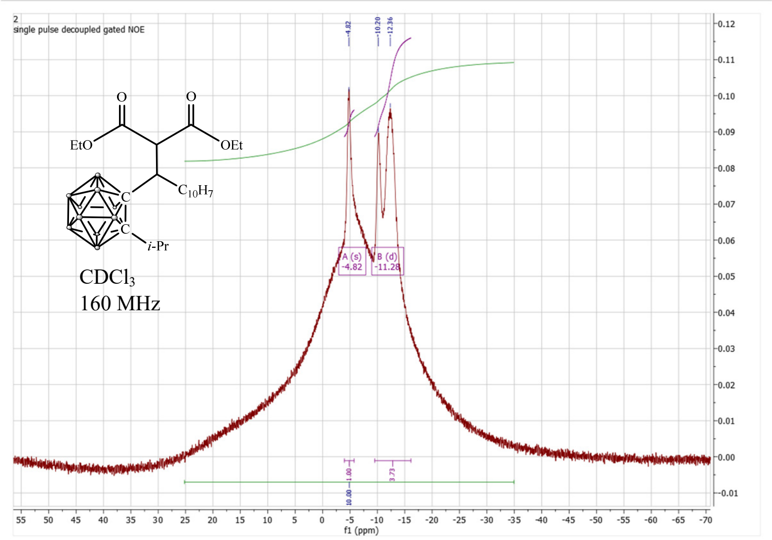

^{11}B NMR spectra of Diethyl 2-[(1-isopropyl-closo-1,2-dicarba-closo-dodecaboran-1-yl)naphthalen-1-yl]propanedioate (6)

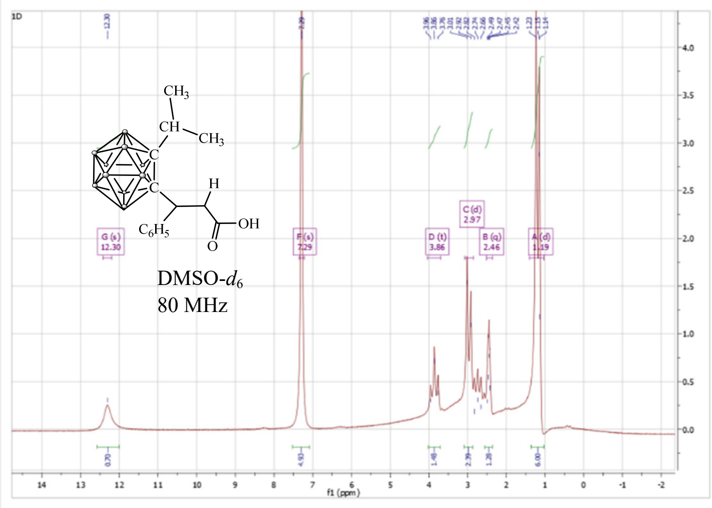

^{1}H NMR spectra of 3-(2-isopropyl-1,2-dicarba-closo-dodecaboran-1-yl)-3-phenylpropanoic acid (7)

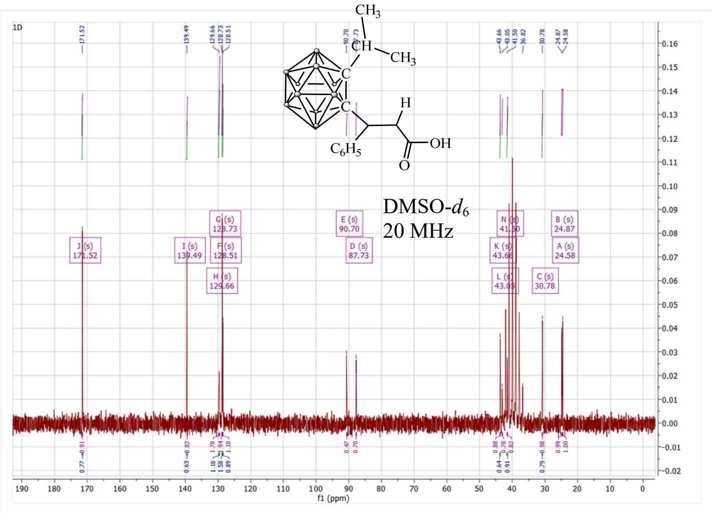

^{13}C NMR spectra of 3-(2-isopropyl-1,2-dicarba-closo-dodecaboran-1-yl)-3-phenylpropanoic acid (7)

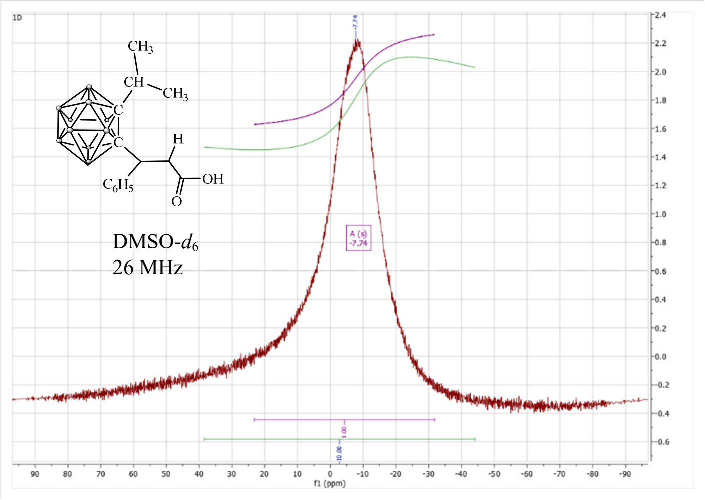

^{11}B NMR spectra of 3-(2-isopropyl-1,2-dicarba-closo-dodecaboran-1-yl)-3-phenylpropanoic acid (7)

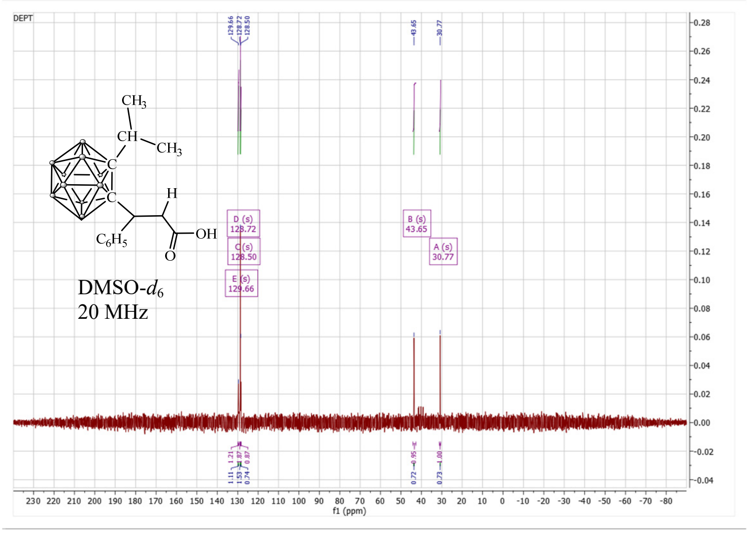

DEPT-90 NMR spectra of 3-(2-isopropyl-1,2-dicarba-closo-dodecaboran-1-yl)-3-phenylpropanoic acid (7)

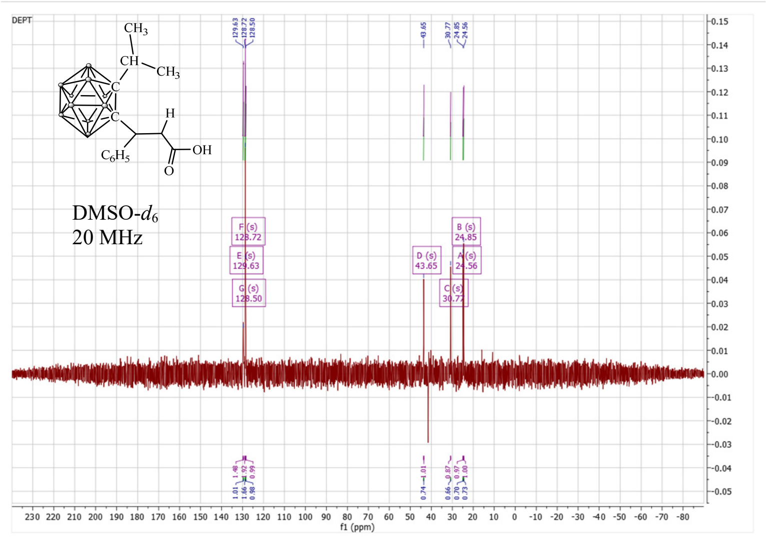

DEPT-135 NMR spectra of 3-(2-isopropyl-1,2-dicarba-closo-dodecaboran-1-yl)-3-phenylpropanoic acid (7)

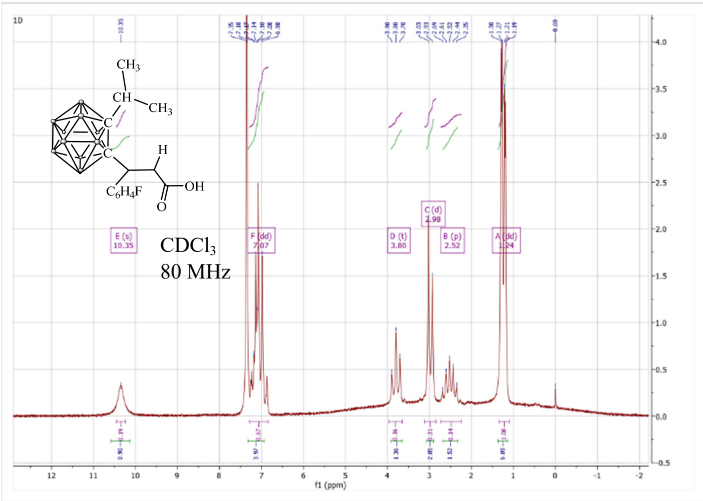

^{1}H NMR spectra of 3-(2-isopropyl-1,2-dicarba-closo-dodecaboran-1-yl)-3-(4-fluorophenyl)propanoic acid (8)

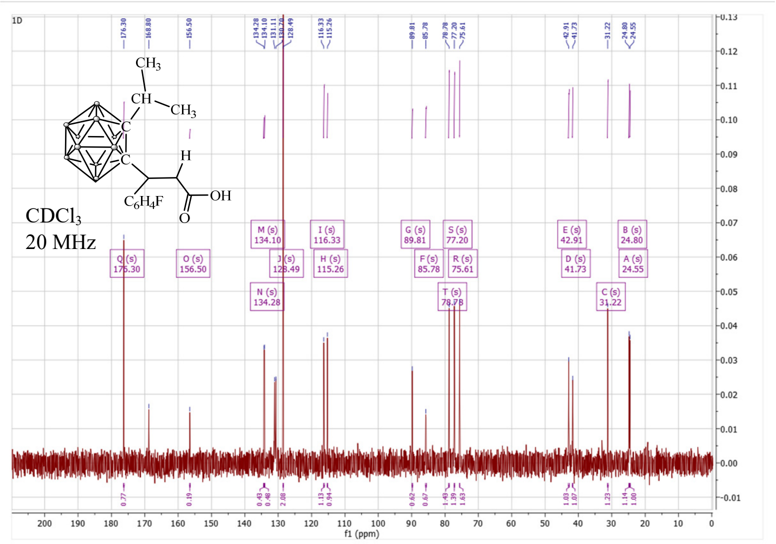

^{13}C NMR spectra of 3-(2-isopropyl-1,2-dicarba-closo-dodecaboran-1-yl)-3-(4-fluorophenyl)propanoic acid (8)

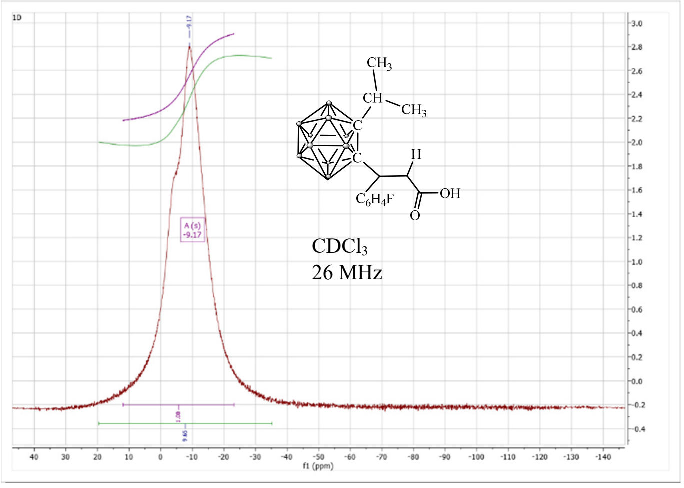

^{11}B NMR spectra of 3-(2-isopropyl-1,2-dicarba-closo-dodecaboran-1-yl)-3-(4-fluorophenyl)propanoic acid (8)

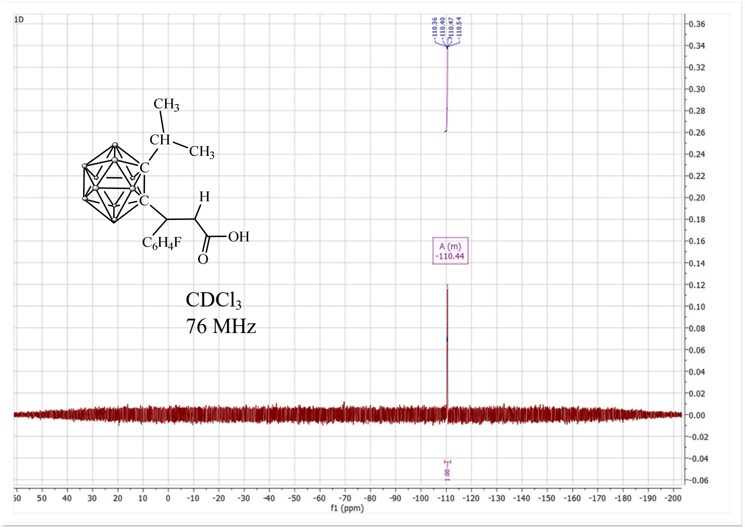

^{19}F NMR spectra of 3-(2-isopropyl-1,2-dicarba-closo-dodecaboran-1-yl)-3-(4-fluorophenyl)propanoic acid (8)

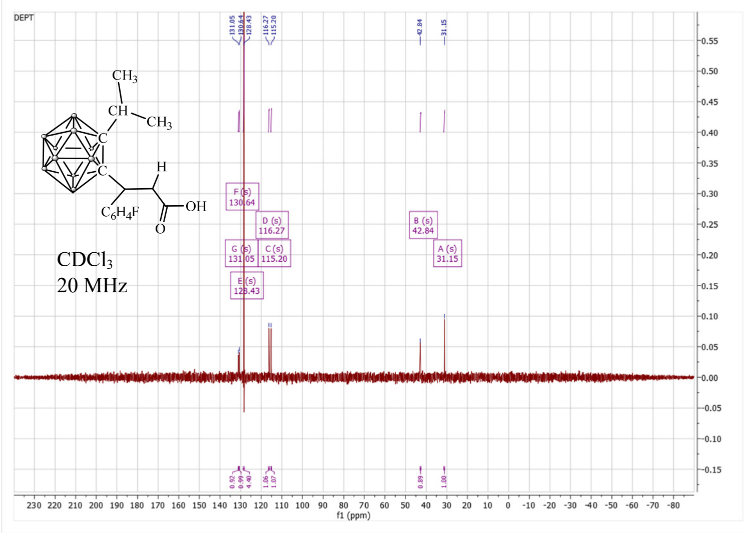

DEPT-90 NMR spectra of 3-(2-isopropyl-1,2-dicarba-closo-dodecaboran-1-yl)-3-(4-fluorophenyl)propanoic acid (8)

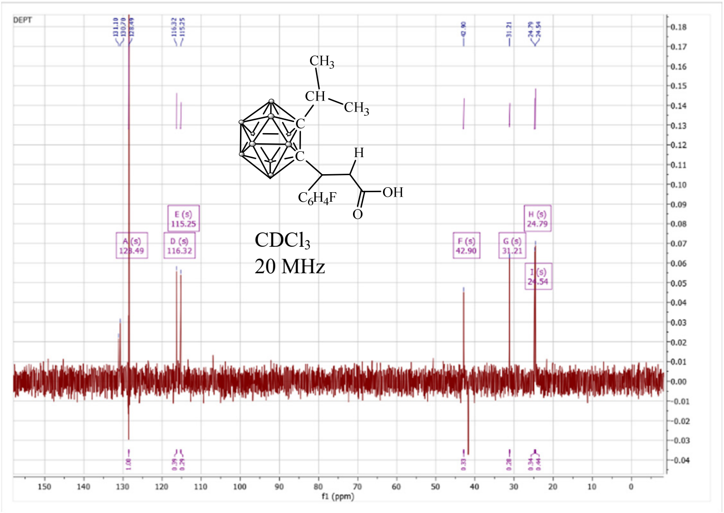

DEPT-135 NMR spectra of 3-(2-isopropyl-1,2-dicarba-closo-dodecaboran-1-yl)-3-(4-fluorophenyl)propanoic acid (8)

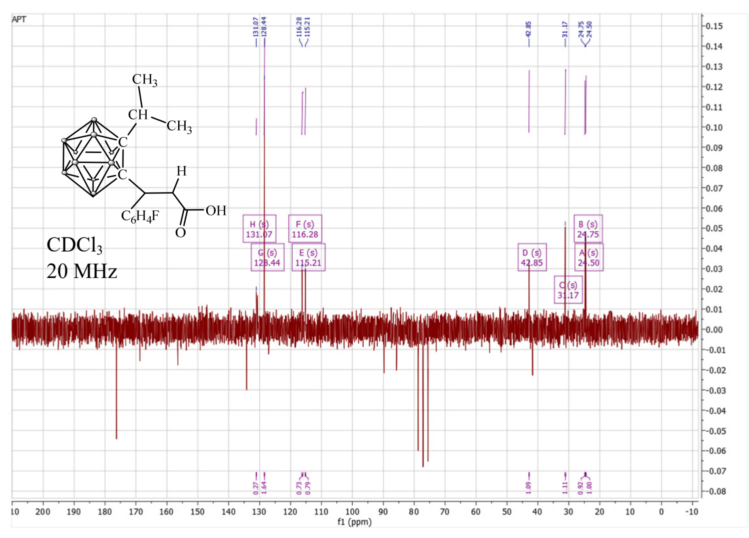

APT-WALTZ NMR spectra of 3-(2-isopropyl-1,2-dicarba-closo-dodecaboran-1-yl)-3-(4-fluorophenyl)propanoic acid (8)

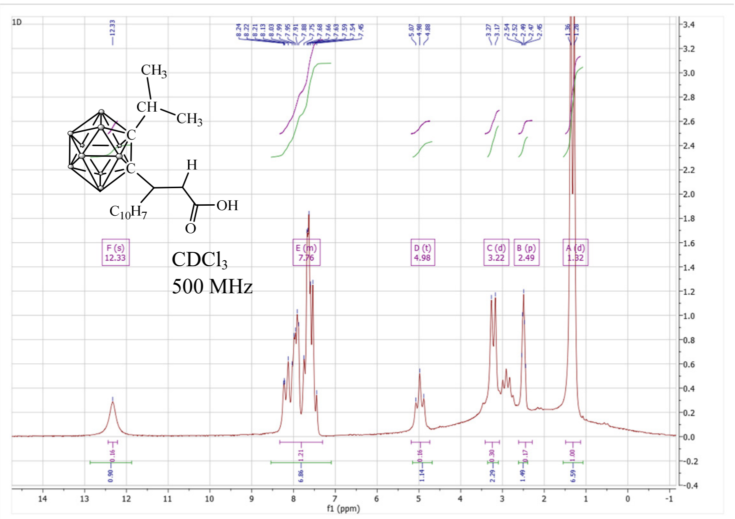

^1H NMR spectra of 3-(2-isopropyl-1,2-dicarba-closo-dodecaboran-1-yl)-3-(naphthalene-1-yl)propanoic acid (9)

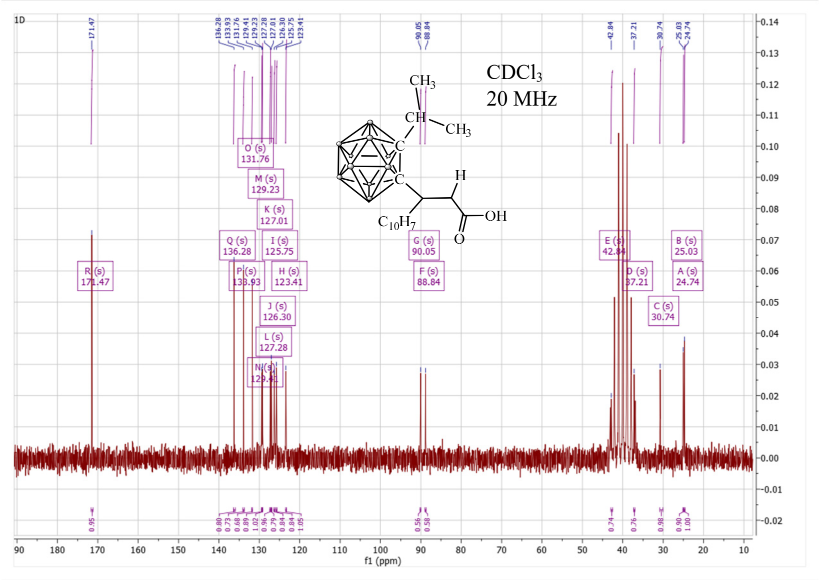

^{13}C NMR spectra of 3-(2-isopropyl-1,2-dicarba-closo-dodecaboran-1-yl)-3-(naphthalene-1-yl)propanoic acid (9)

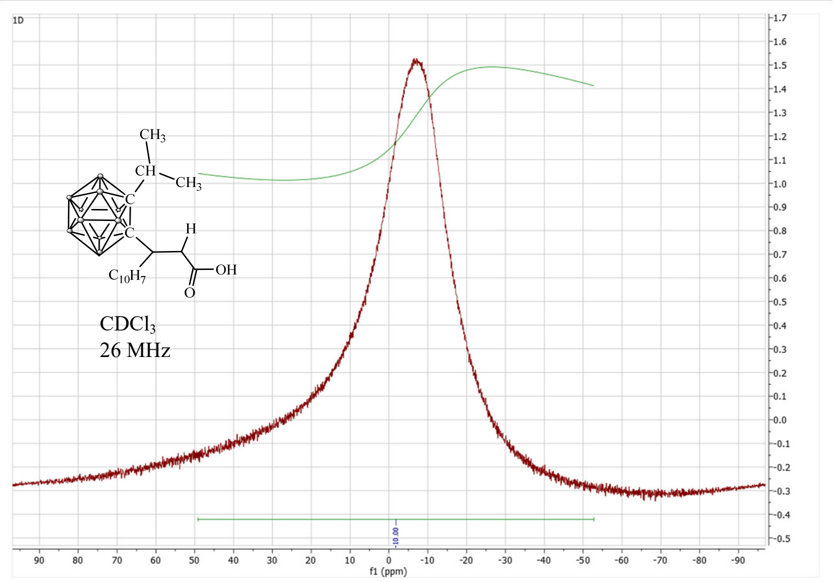

^{11}B NMR spectra of 3-(2-isopropyl-1,2-dicarba-closo-dodecaboran-1-yl)-3-(naphthalene-1-yl)propanoic acid (9)

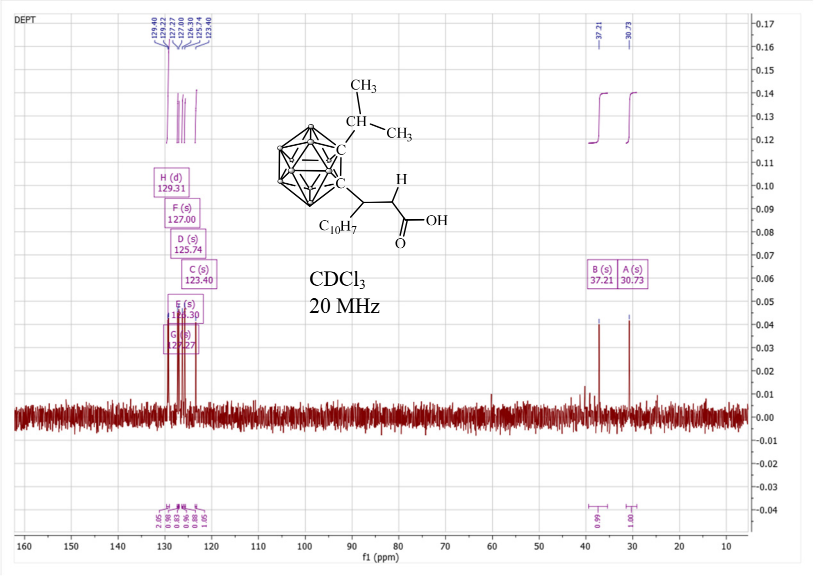

DEPT-90 NMR spectra of 3-(2-isopropyl-1,2-dicarba-closo-dodecaboran-1-yl)-3-(naphthalene-1-yl)propanoic acid (9)

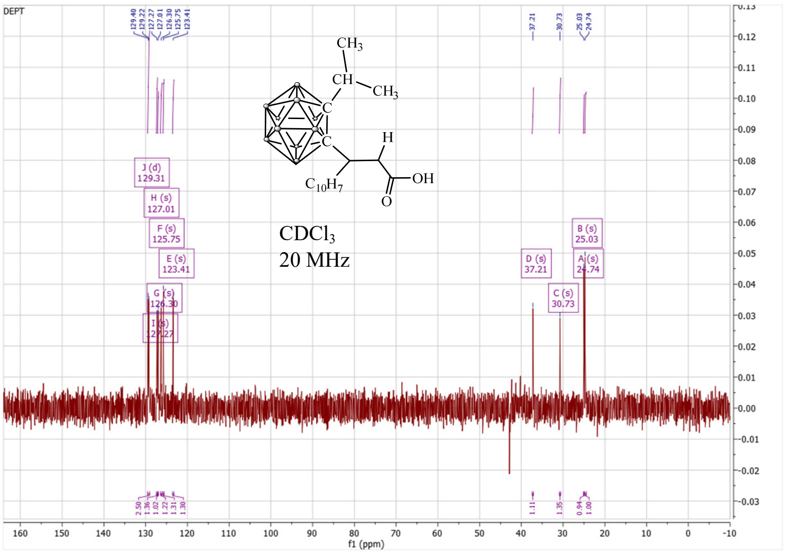

DEPT-135 NMR spectra of 3-(2-isopropyl-1,2-dicarba-closo-dodecaboran-1-yl)-3-(naphthalene-1-yl)propanoic acid (9)

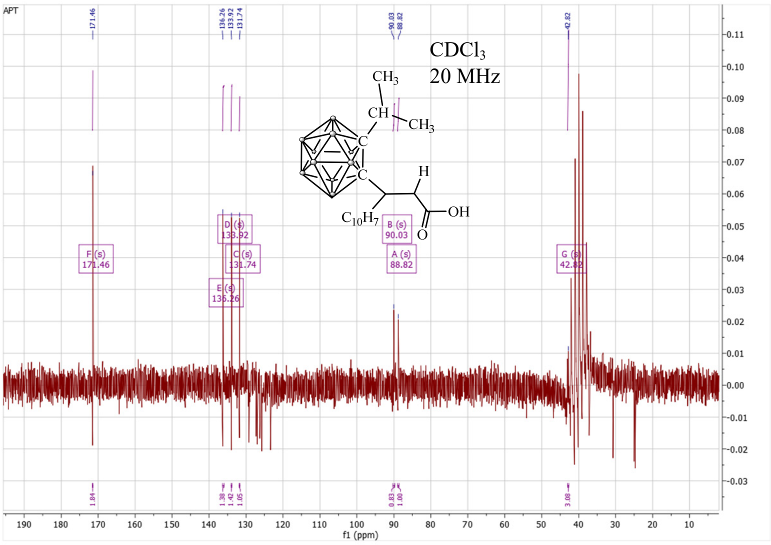

APT-WALTZ NMR spectra of 3-(2-isopropyl-1,2-dicarba-closo-dodecaboran-1-yl)-3-(naphthalene-1-yl)propanoic acid (9)

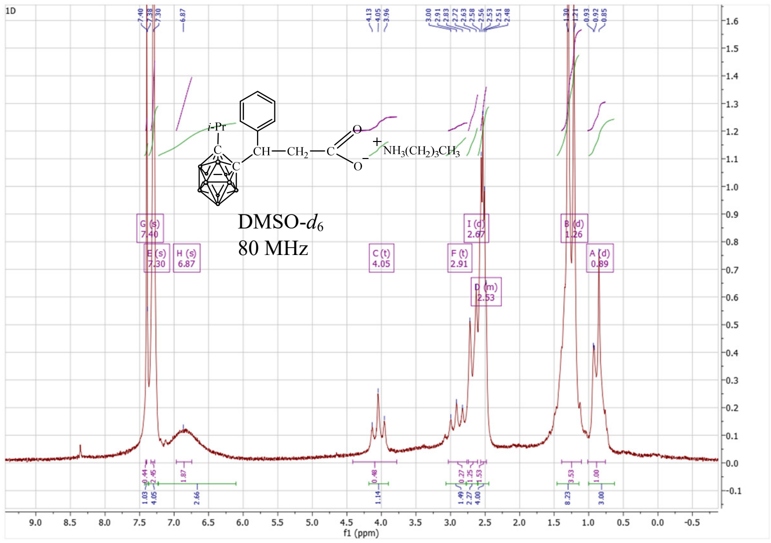

^{1}H NMR spectra of Butylammonium 3-(2-isopropyl-1,2-dicarba-closo-dodecaboran-1-yl)-3-phenylpropanoate (10)

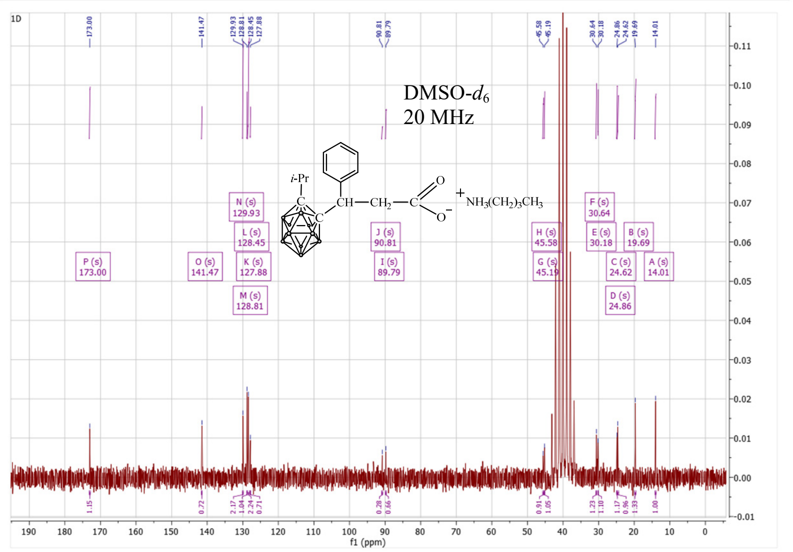

^{13}C NMR spectra of Butylammonium 3-(2-isopropyl-1,2-dicarba-closo-dodecaboran-1-yl)-3-phenylpropanoate (10)

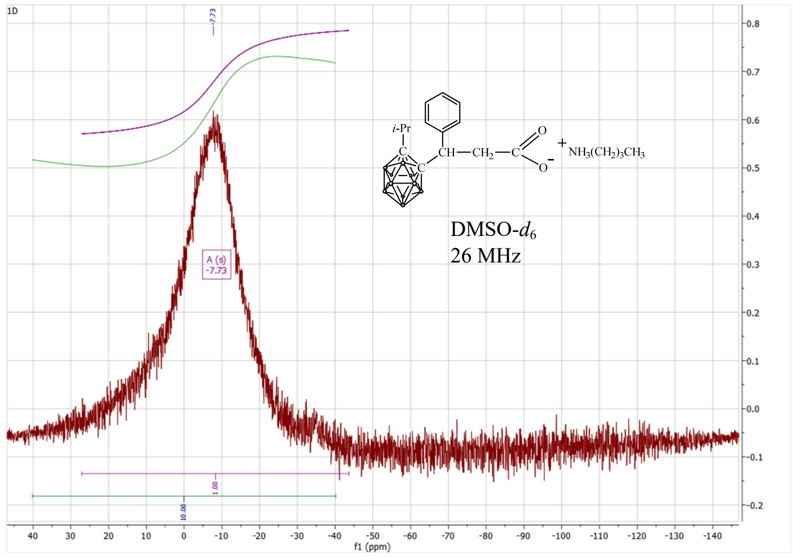

^{11}B NMR spectra of Butylammonium 3-(2-isopropyl-1,2-dicarba-closo-dodecaboran-1-yl)-3-phenylpropanoate (10)

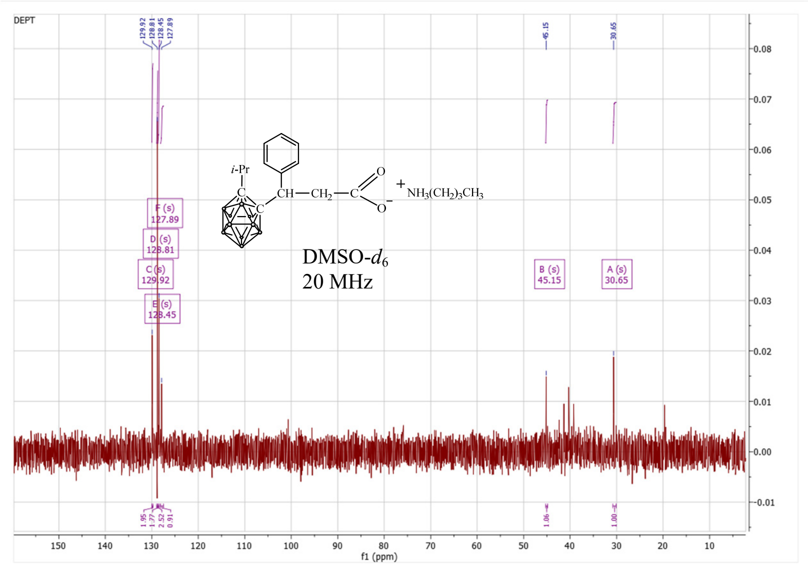

DEPT-90 NMR spectra of Butylammonium 3-(2-isopropyl-1,2-dicarba-closo-dodecaboran-1-yl)-3-phenylpropanoate (10)

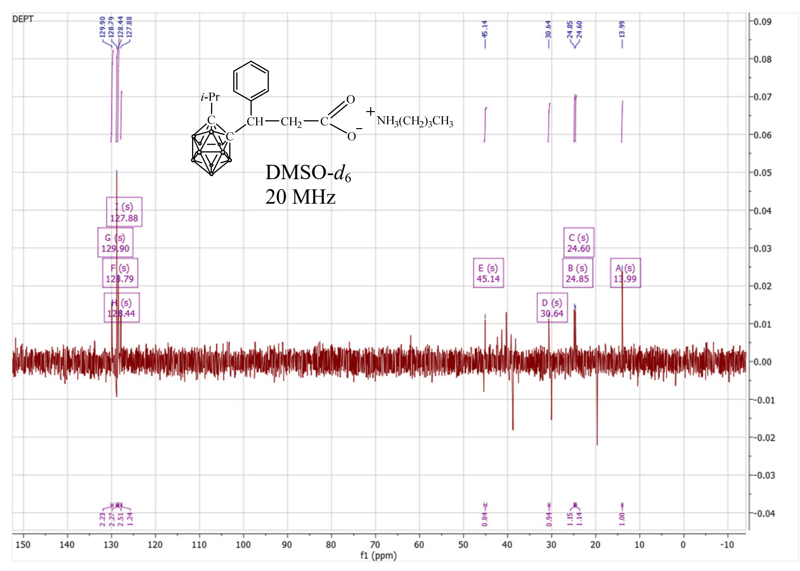

DEPT-135 NMR spectra of Butylammonium 3-(2-isopropyl-1,2-dicarba-closo-dodecaboran-1-yl)-3-phenylpropanoate (10)

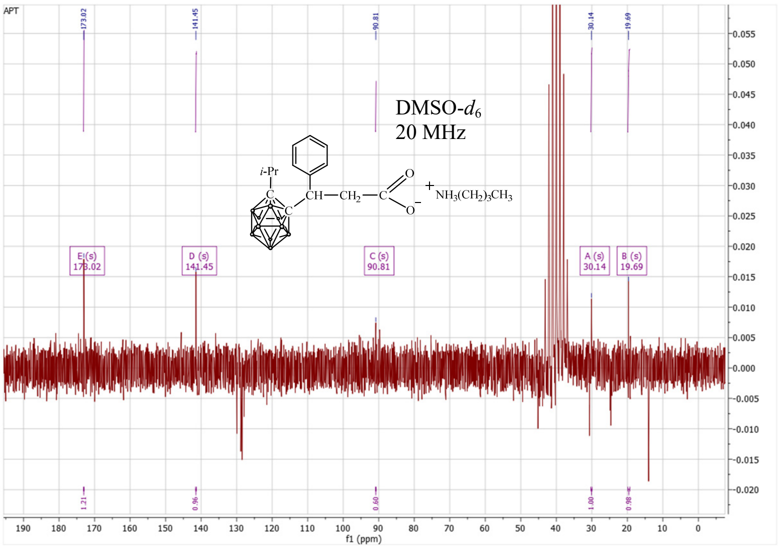

APT-WALTZ NMR spectra of Butylammonium 3-(2-isopropyl-1,2-dicarba-closo-dodecaboran-1-yl)-3-phenylpropanoate (10)

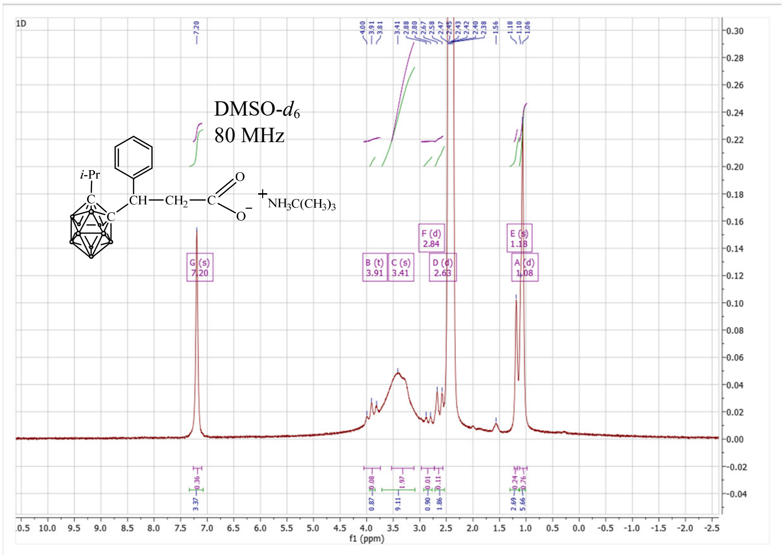

^{1}H NMR spectra of Tert-butylammonium 3-(2-isopropyl-1,2-dicarba-closo-dodecaboran-1-yl)-3-phenylpropanoate (11)

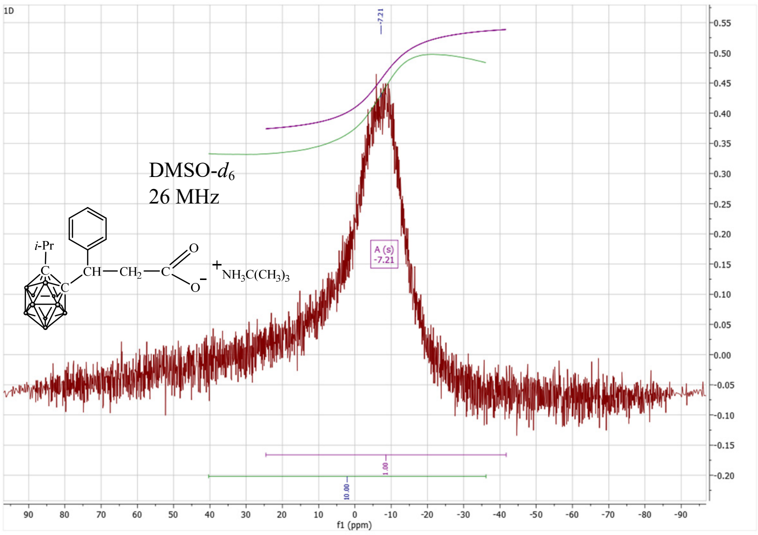

^{11}B NMR spectra of Tert-butylammonium 3-(2-isopropyl-1,2-dicarba-closo-dodecaboran-1-yl)-3-phenylpropanoate (11)

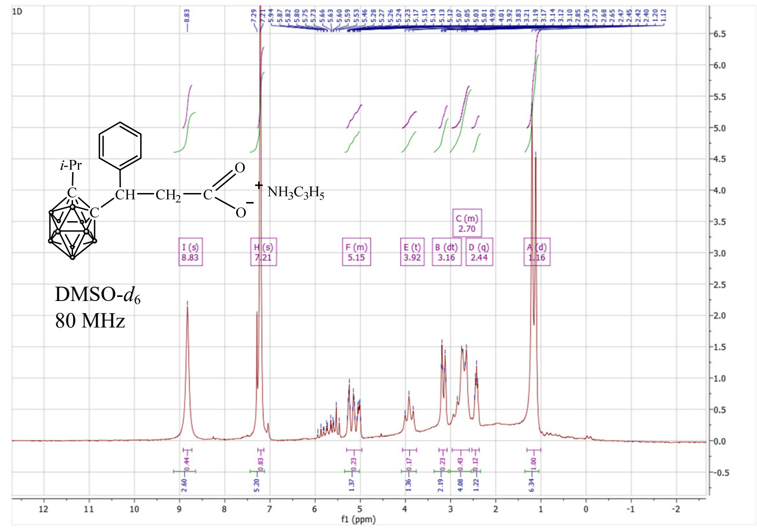

^{1}H NMR spectra of Allylammonium 3-(2-isopropyl-1,2-dicarba-closo-dodecaboran-1-yl)-3-phenylpropanoate (12)

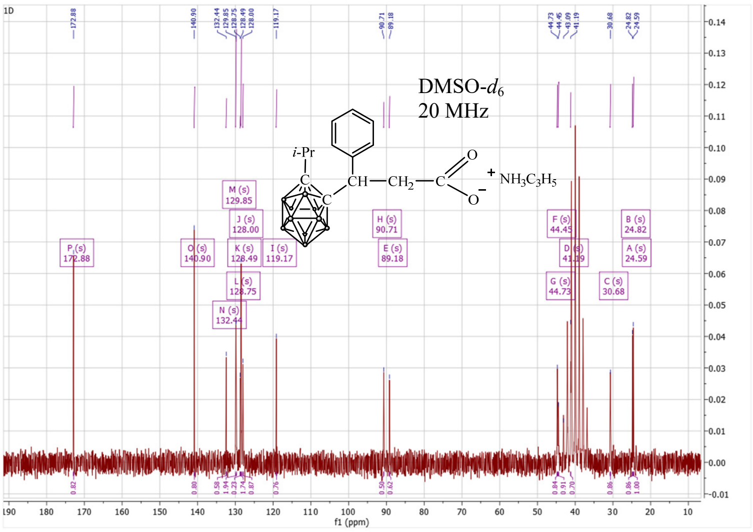

^{13}C NMR spectra of Allylammonium 3-(2-isopropyl-1,2-dicarba-closo-dodecaboran-1-yl)-3-phenylpropanoate (12)

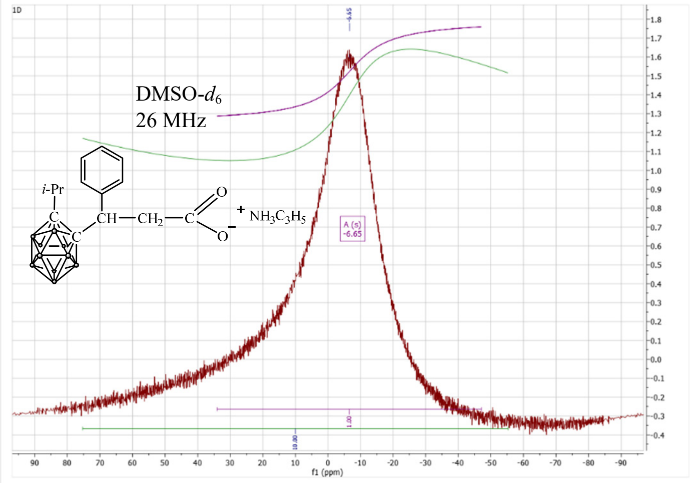

^{11}B NMR spectra of Allylammonium 3-(2-isopropyl-1,2-dicarba-closo-dodecaboran-1-yl)-3-phenylpropanoate (12)

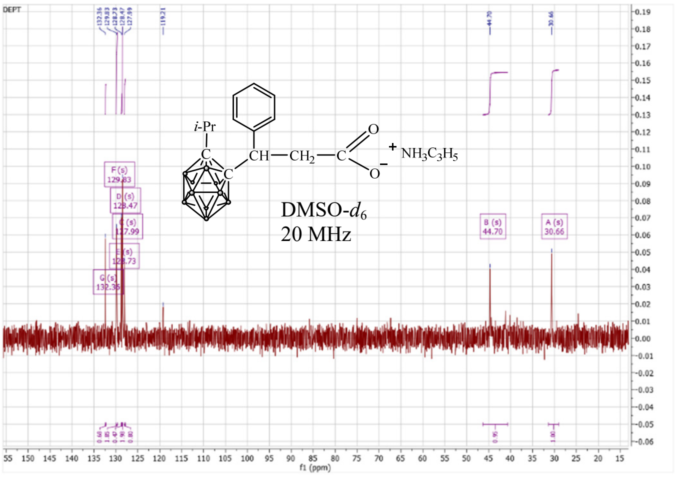

DEPT-90 NMR spectra of Allylammonium 3-(2-isopropyl-1,2-dicarba-closo-dodecaboran-1-yl)-3-phenylpropanoate (12)

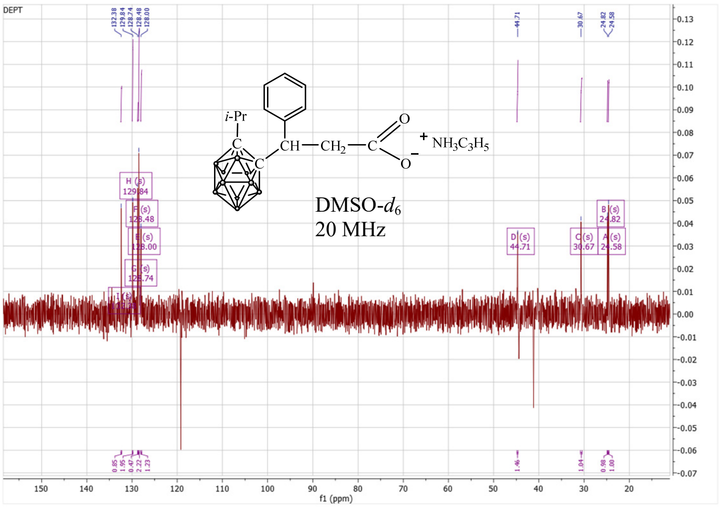

DEPT-135 NMR spectra of Allylammonium 3-(2-isopropyl-1,2-dicarba-closo-dodecaboran-1-yl)-3-phenylpropanoate (12)

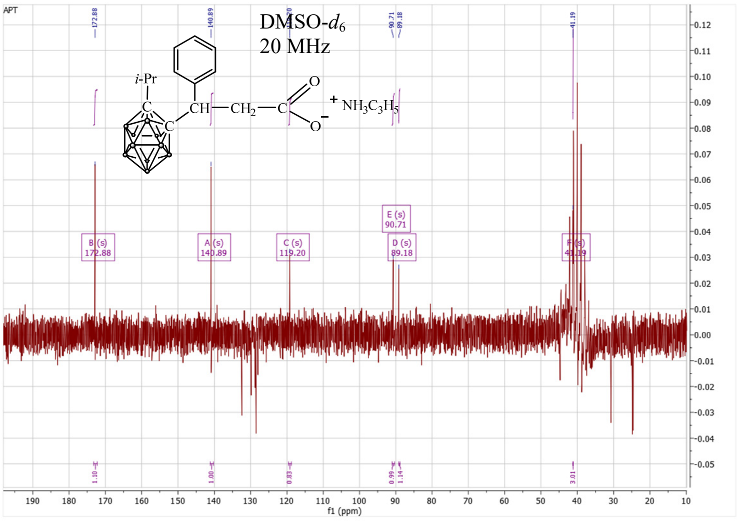

APT-WALTZ NMR spectra of Allylammonium 3-(2-isopropyl-1,2-dicarba-closo-dodecaboran-1-yl)-3-phenylpropanoate (12)

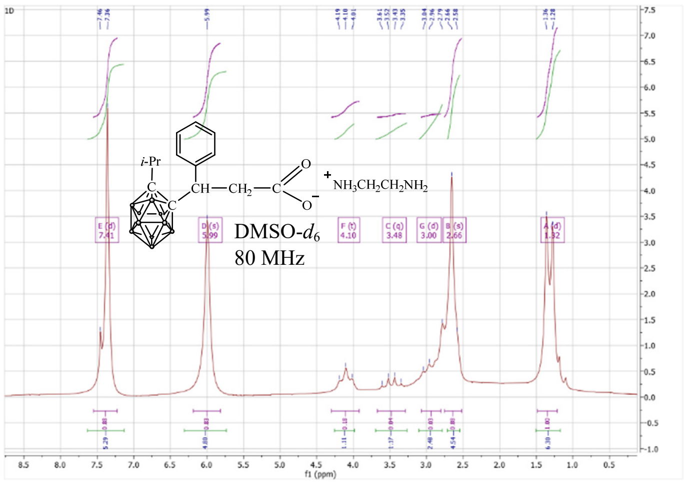

^{1}H NMR spectra of Ethan-1-amino-2-ammonium 3-(2-isopropyl-1,2-dicarba-closo-dodecaboran-1-yl)-3-phenylpropanoate (13)

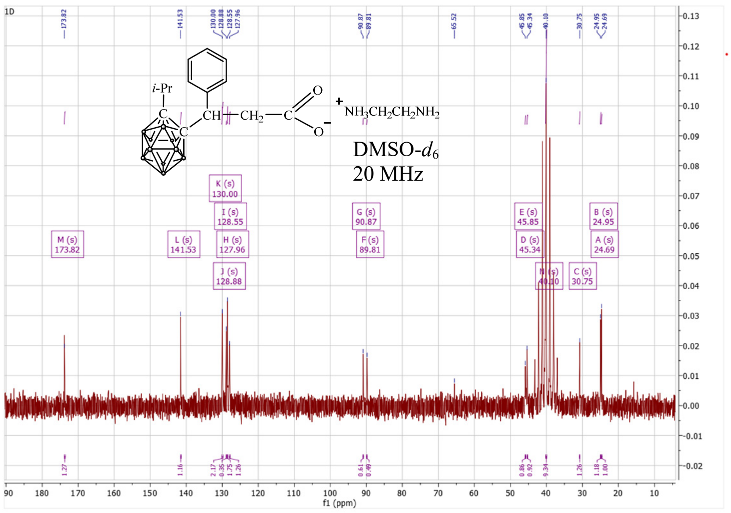

^{13}C NMR spectra of Ethan-1-amino-2-ammonium 3-(2-isopropyl-1,2-dicarba-closo-dodecaboran-1-yl)-3-phenylpropanoate (13)

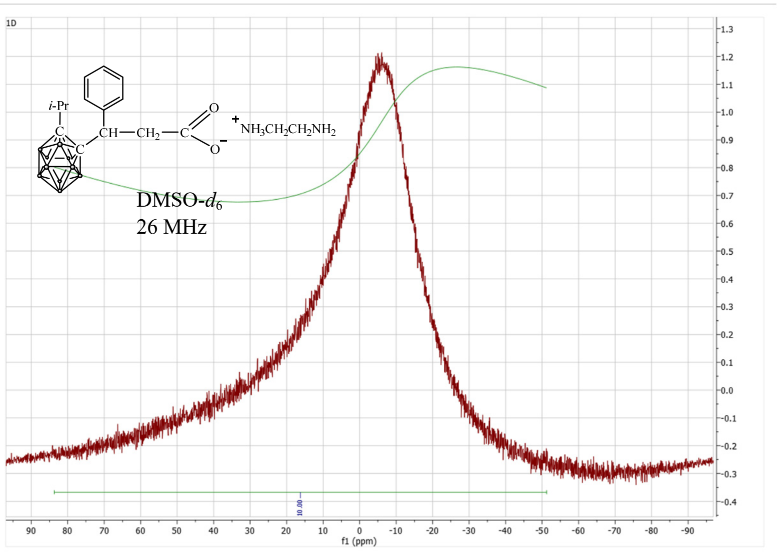

^{11}B NMR spectra of Ethan-1-amino-2-ammonium 3-(2-isopropyl-1,2-dicarba-closo-dodecaboran-1-yl)-3-phenylpropanoate (13)

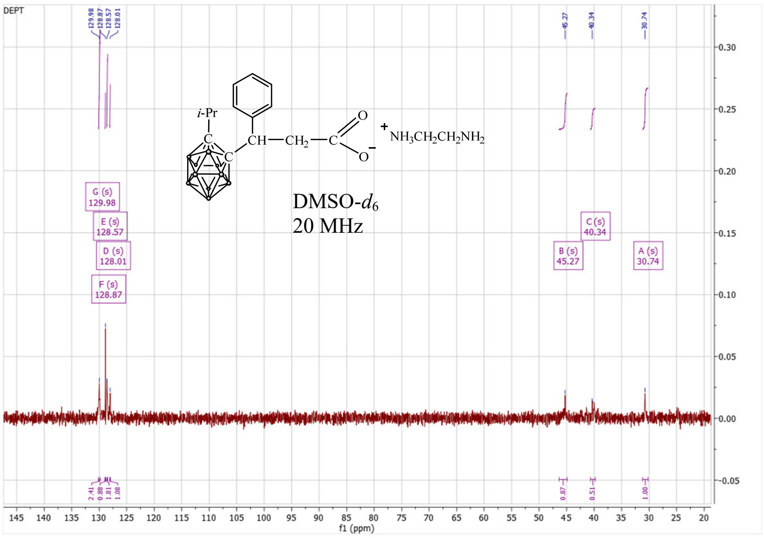

DEPT-90 NMR spectra of Ethan-1-amino-2-ammonium 3-(2-isopropyl-1,2-dicarba-closo-dodecaboran-1-yl)-3-phenylpropanoate (13)

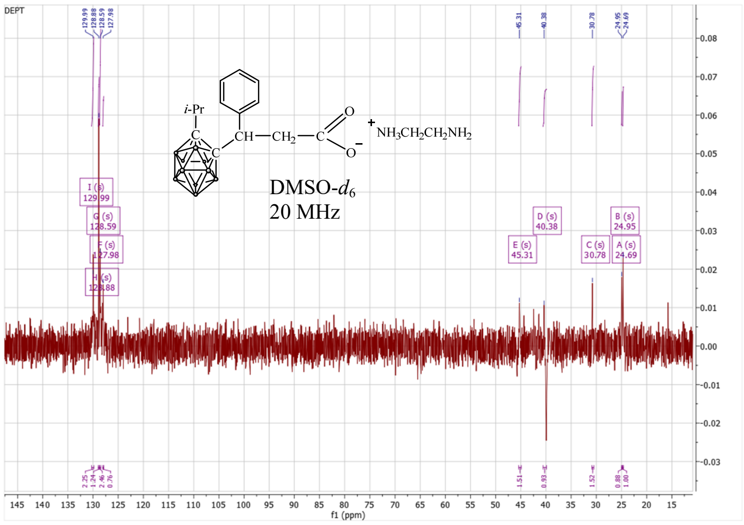

DEPT-135 NMR spectra of Ethan-1-amino-2-ammonium 3-(2-isopropyl-1,2-dicarba-closo-dodecaboran-1-yl)-3-phenylpropanoate (13)

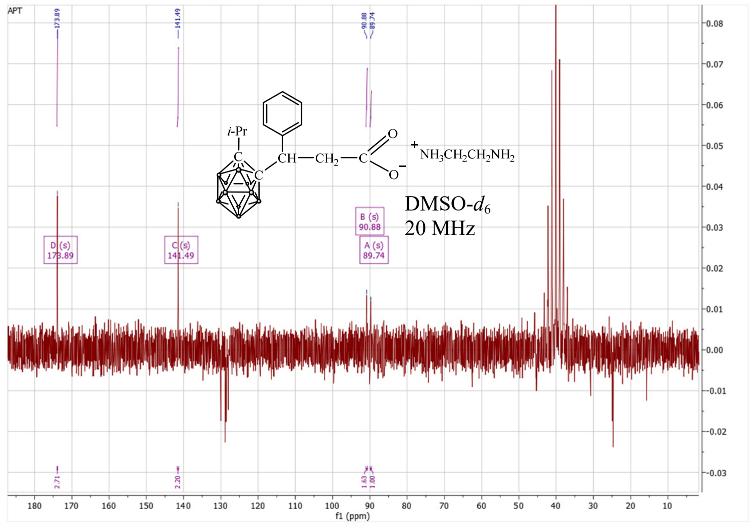

APT-WALTZ NMR spectra of Ethan-1-amino-2-ammonium 3-(2-isopropyl-1,2-dicarba-closo-dodecaboran-1-yl)-3-phenylpropanoate (13)

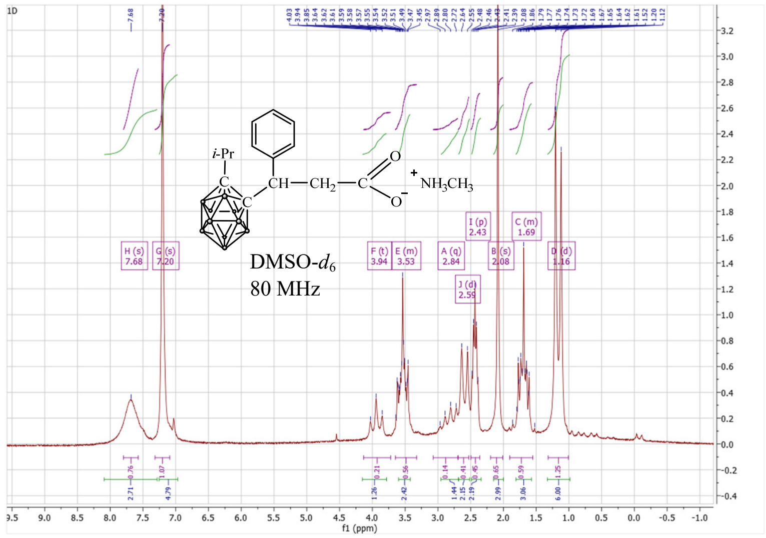

^{1}H NMR spectra of Methylammonium 3-(2-isopropyl-1,2-dicarba-closo-dodecaboran-1-yl)-3-phenylpropanoate (14)

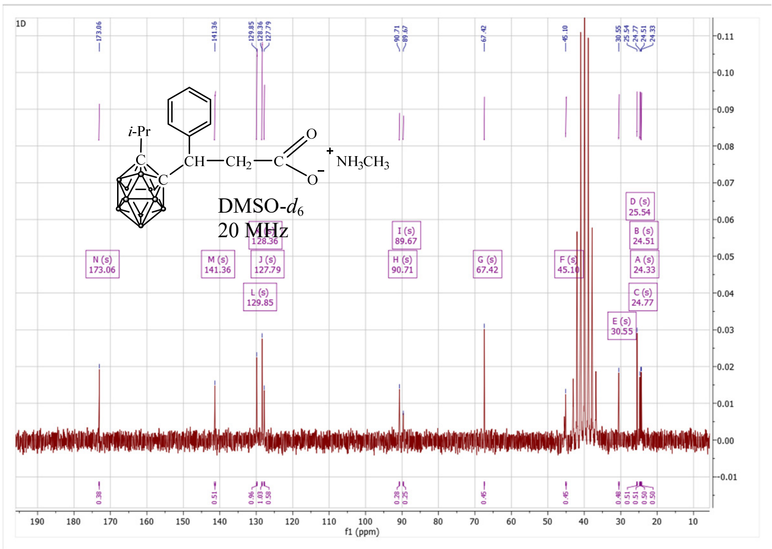

^{13}C NMR spectra of Methylammonium 3-(2-isopropyl-1,2-dicarba-closo-dodecaboran-1-yl)-3-phenylpropanoate (14)

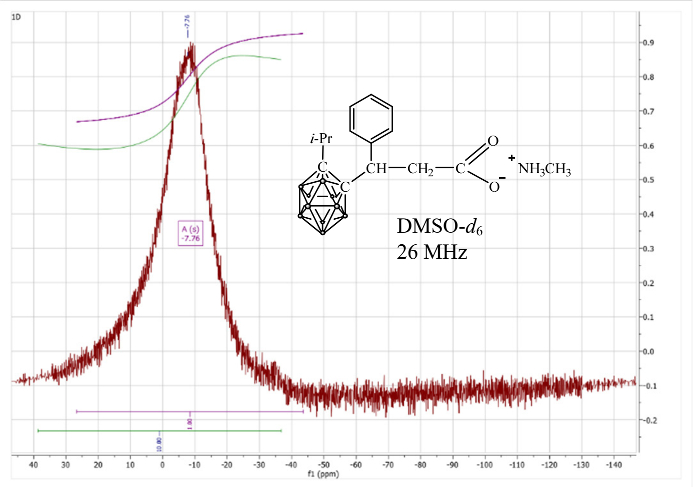

^{11}B NMR spectra of Methylammonium 3-(2-isopropyl-1,2-dicarba-closo-dodecaboran-1-yl)-3-phenylpropanoate (14)

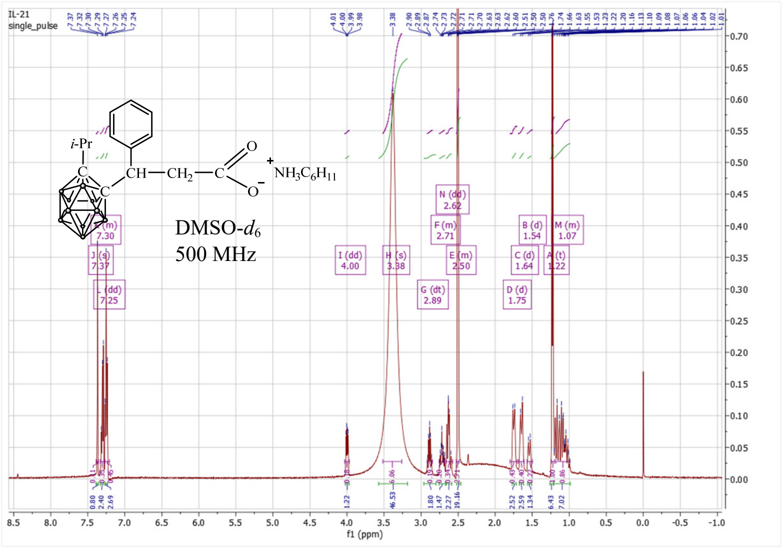

^{1}H NMR spectra of Cyclohexylammonium 3-(2-isopropyl-1,2-dicarba-closo-dodecaboran-1-yl)-3-phenylpropanoate (15)

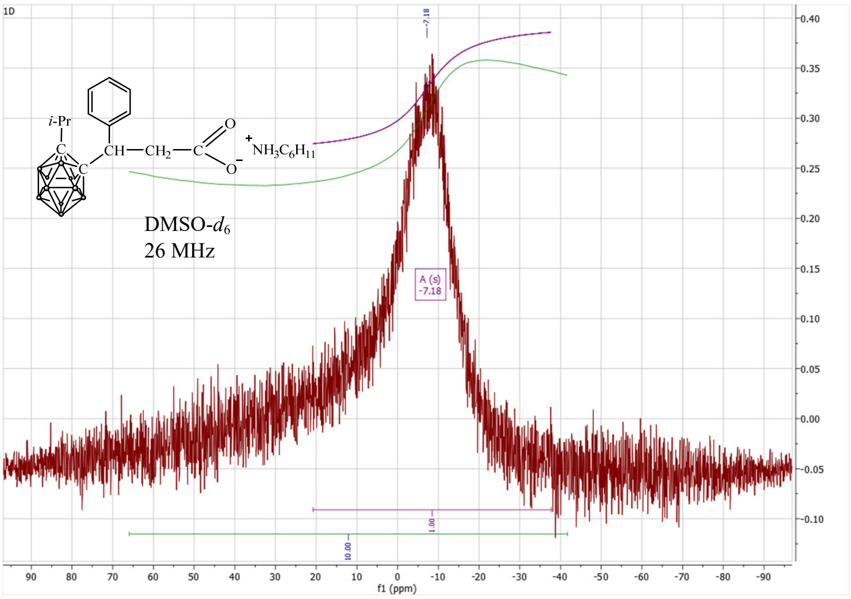

^{11}B NMR spectra of Cyclohexylammonium 3-(2-isopropyl-1,2-dicarba-closo-dodecaboran-1-yl)-3-phenylpropanoate (15)

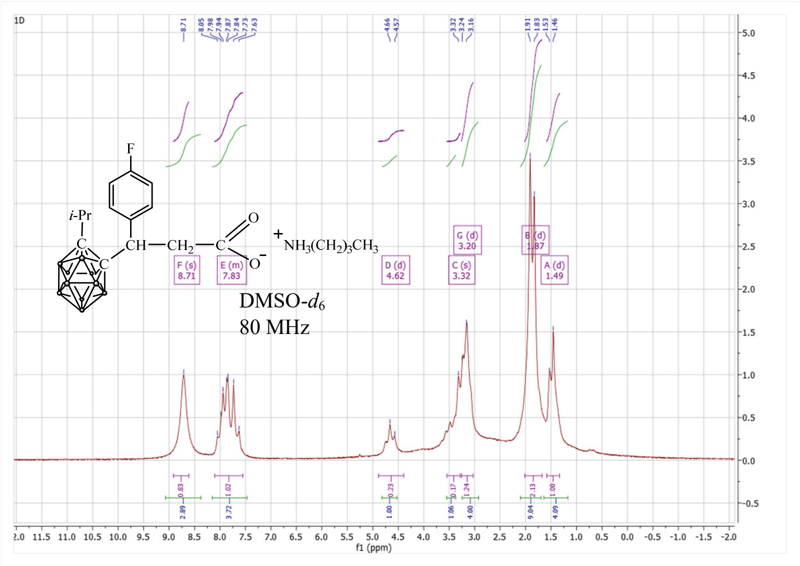

1H NMR spectra of Butylammonium 3-(2-isopropyl-1,2-dicarba-closo-dodecaboran-1-yl)-3-(4-fluorophenyl)propanoate (16)

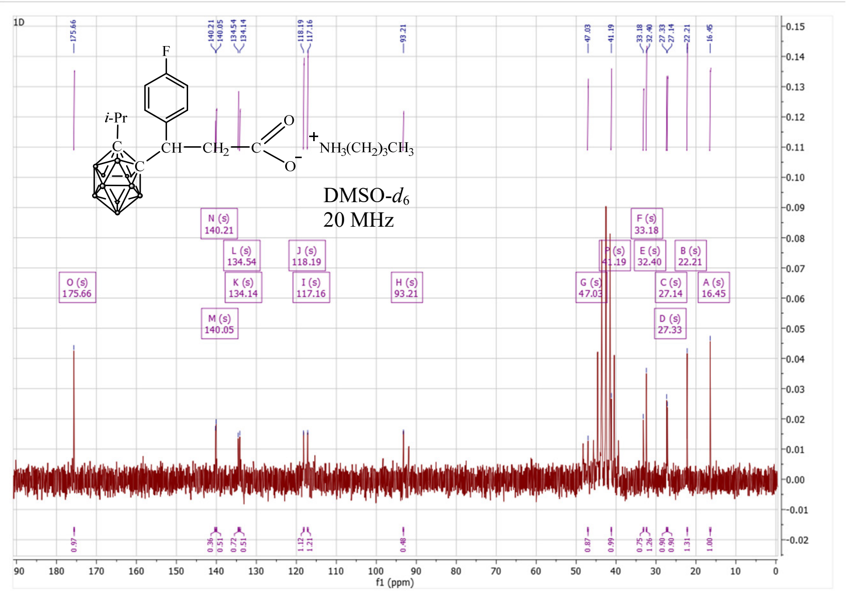

^{13}C NMR spectra of Butylammonium 3-(2-isopropyl-1,2-dicarba-closo-dodecaboran-1-yl)-3-(4-fluorophenyl)propanoate (16)

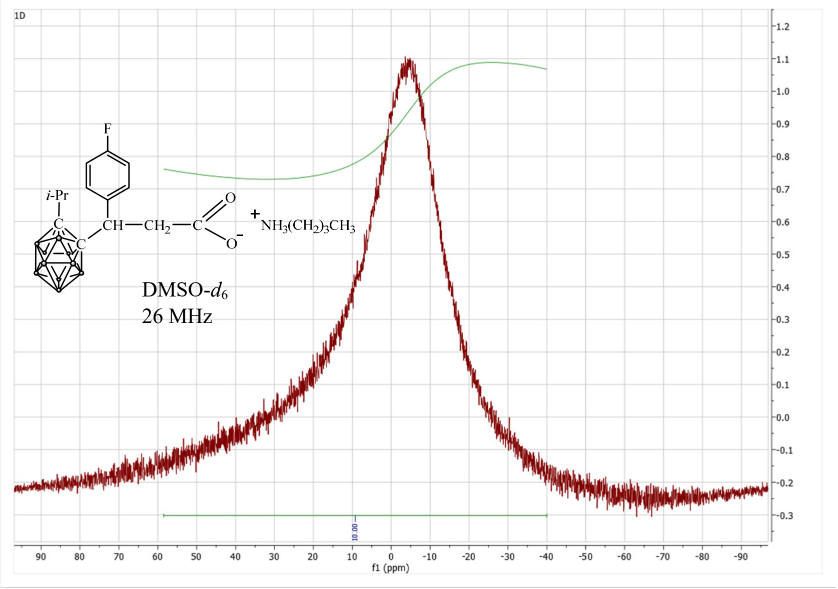

^{11}B NMR spectra of Butylammonium 3-(2-isopropyl-1,2-dicarba-closo-dodecaboran-1-yl)-3-(4-fluorophenyl)propanoate (16)

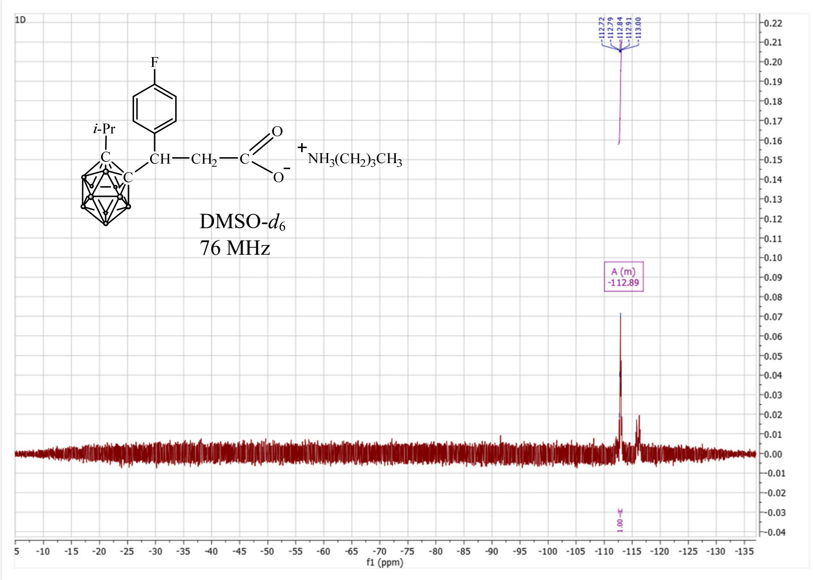

^{19}F NMR spectra of Butylammonium 3-(2-isopropyl-1,2-dicarba-closo-dodecaboran-1-yl)-3-(4-fluorophenyl)propanoate (16)

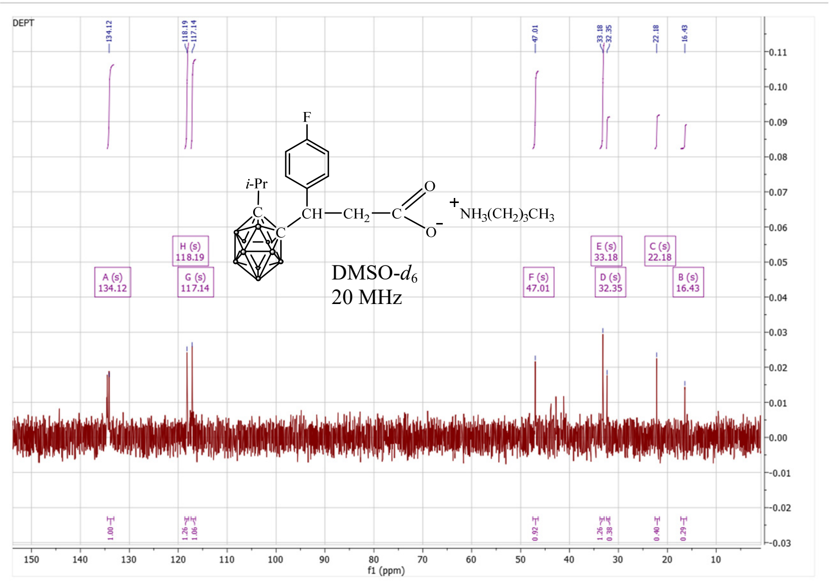

DEPT-90 NMR spectra of Butylammonium 3-(2-isopropyl-1,2-dicarba-closo-dodecaboran-1-yl)-3-(4-fluorophenyl)propanoate (16)

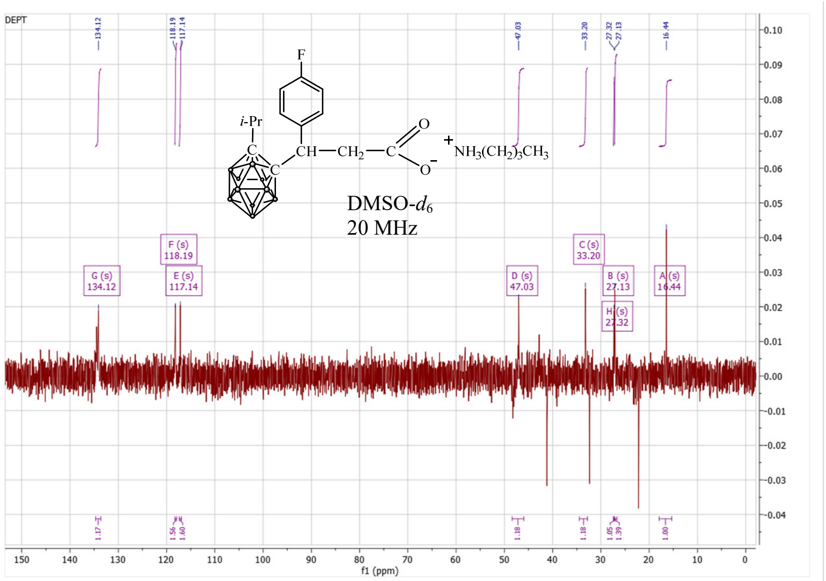

DEPT-135 NMR spectra of Butylammonium 3-(2-isopropyl-1,2-dicarba-closo-dodecaboran-1-yl)-3-(4-fluorophenyl)propanoate (16)

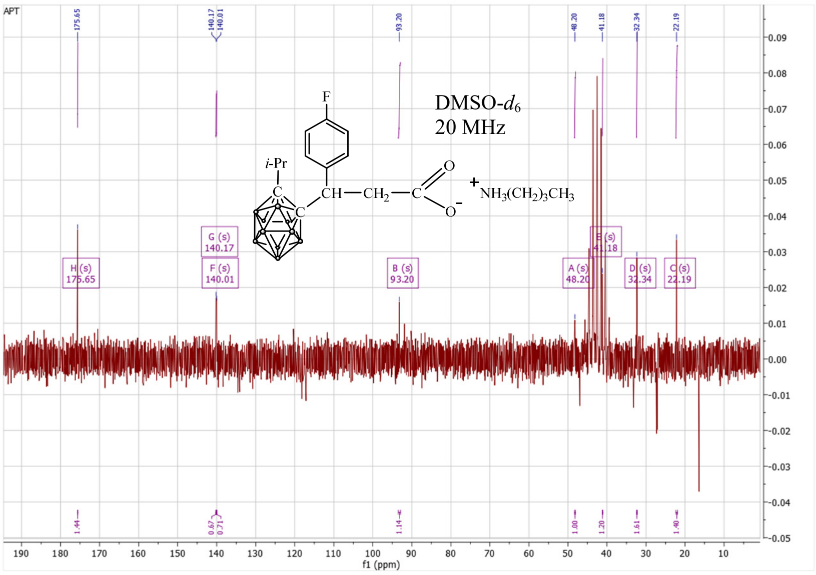

APT-WALTZ NMR spectra of Butylammonium 3-(2-isopropyl-1,2-dicarba-closo-dodecaboran-1-yl)-3-(4-fluorophenyl)propanoate (16)

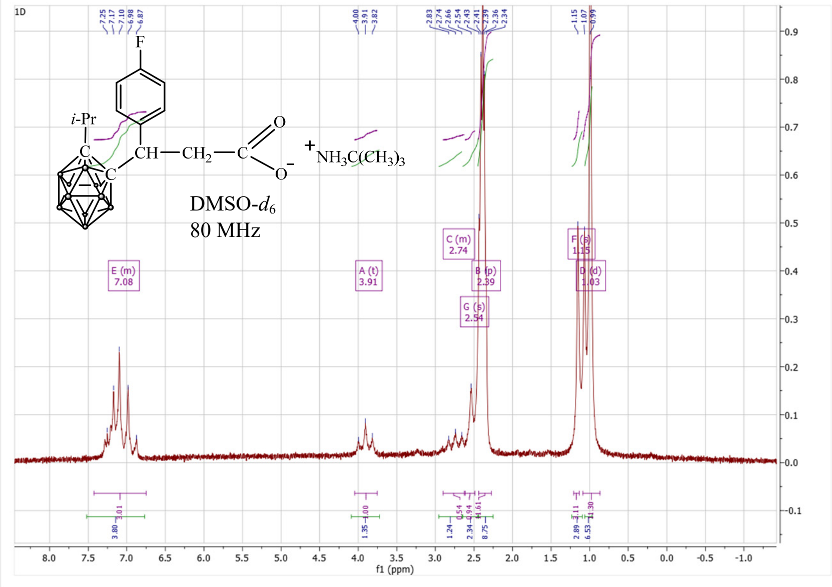

^{1}H NMR spectra of Tert-butylammonium 3-(2-isopropyl-1,2-dicarba-closo-dodecaboran-1-yl)-3-(4-fluorophenyl)propanoate (17)

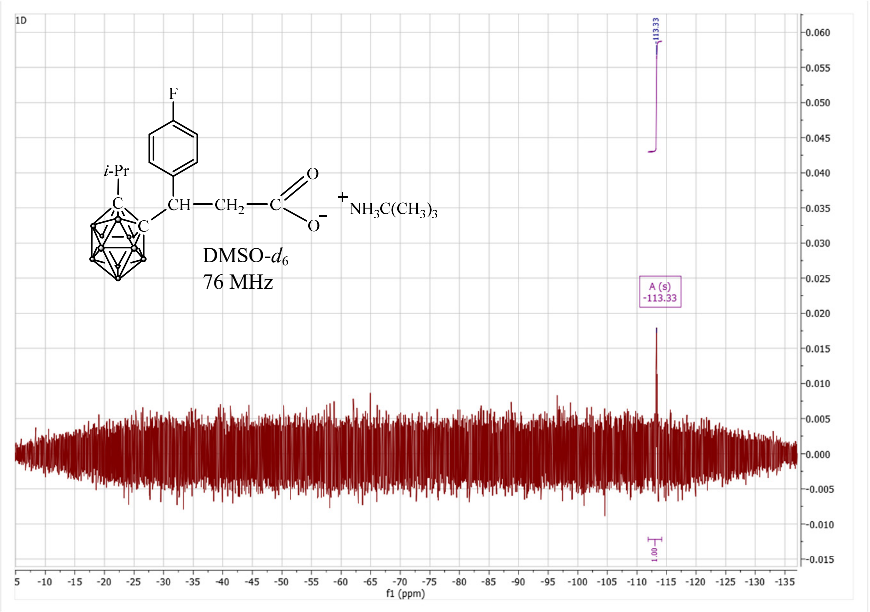

^{19}F NMR spectra of Tert-butylammonium 3-(2-isopropyl-1,2-dicarba-closo-dodecaboran-1-yl)-3-(4-fluorophenyl)propanoate (17)

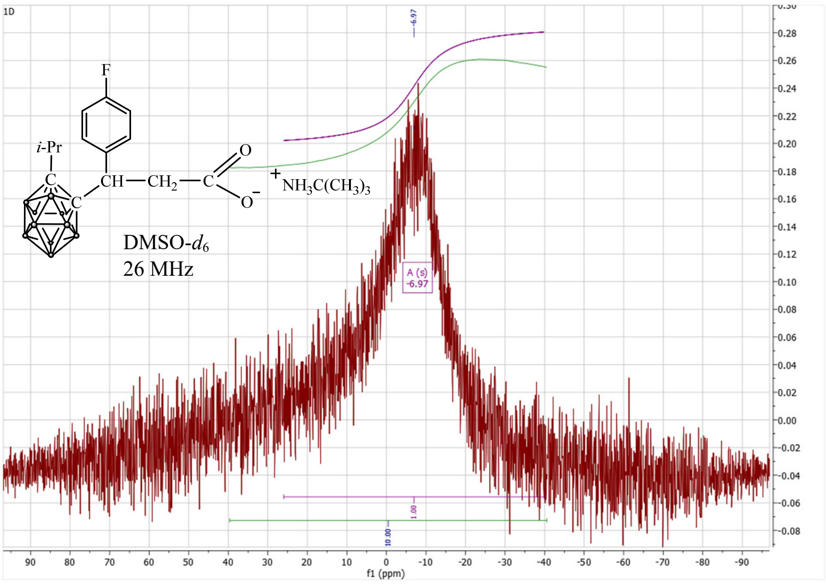

^{11}B NMR spectra of Tert-butylammonium 3-(2-isopropyl-1,2-dicarba-closo-dodecaboran-1-yl)-3-(4-fluorophenyl)propanoate (17)

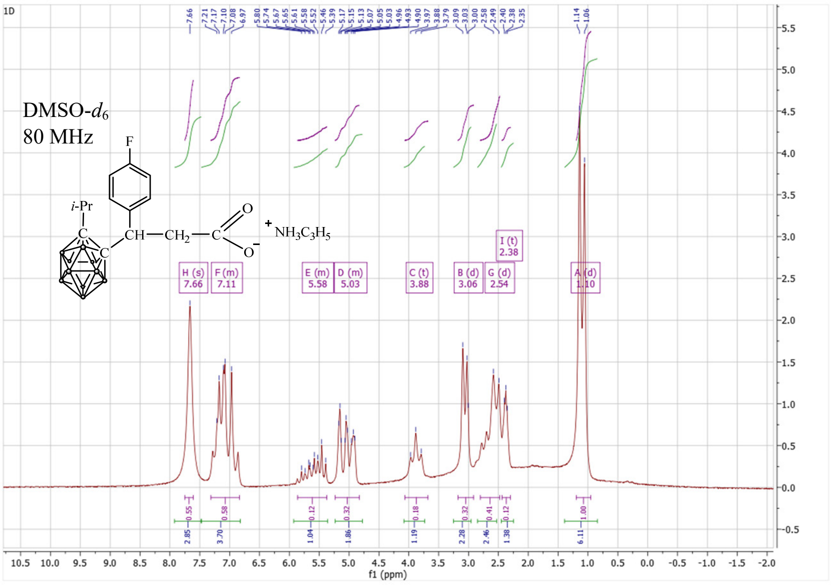

^{1}H NMR spectra of Allylammonium 3-(2-isopropyl-1,2-dicarba-closo-dodecaboran-1-yl)-3-(4-fluorophenyl)propanoate (18)

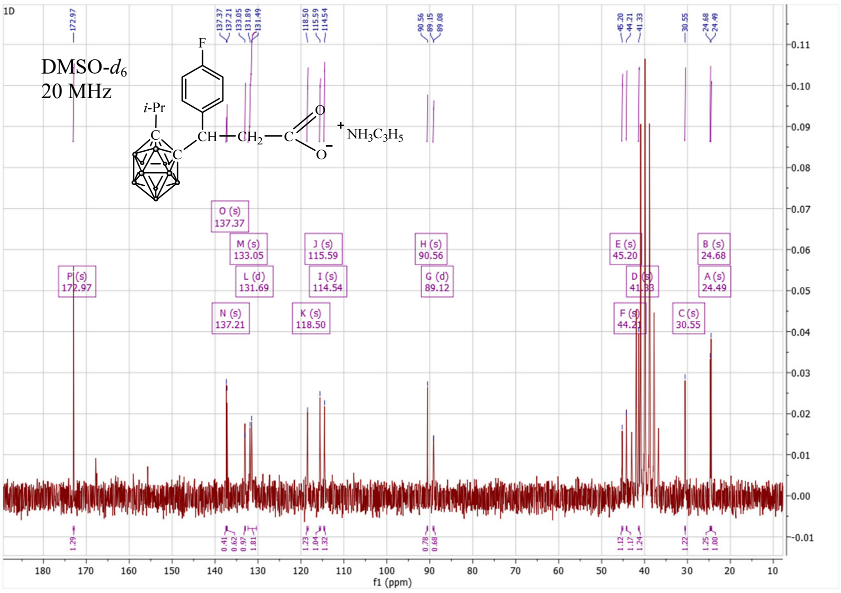

^{13}C NMR spectra of Allylammonium 3-(2-isopropyl-1,2-dicarba-closo-dodecaboran-1-yl)-3-(4-fluorophenyl)propanoate (18)

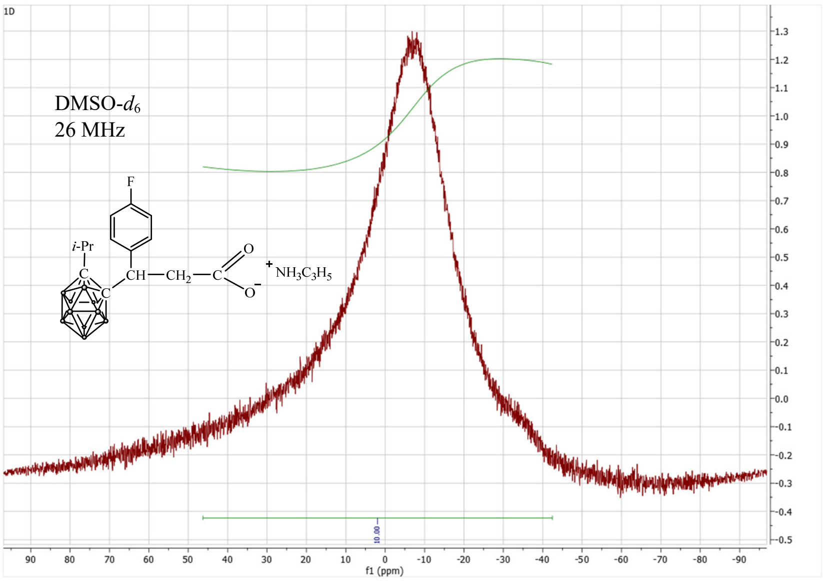

^{11}B NMR spectra of Allylammonium 3-(2-isopropyl-1,2-dicarba-closo-dodecaboran-1-yl)-3-(4-fluorophenyl)propanoate (18)

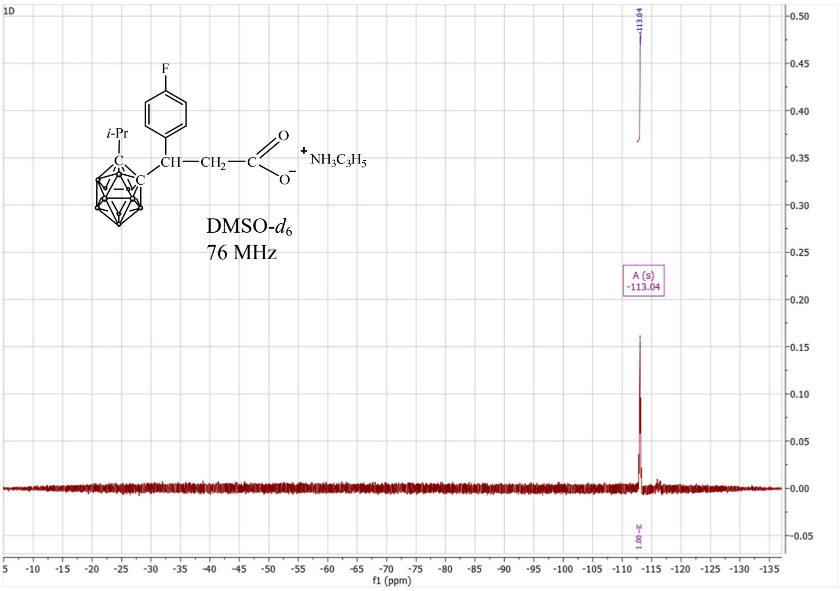

^{19}F NMR spectra of Allylammonium 3-(2-isopropyl-1,2-dicarba-closo-dodecaboran-1-yl)-3-(4-fluorophenyl)propanoate (18)

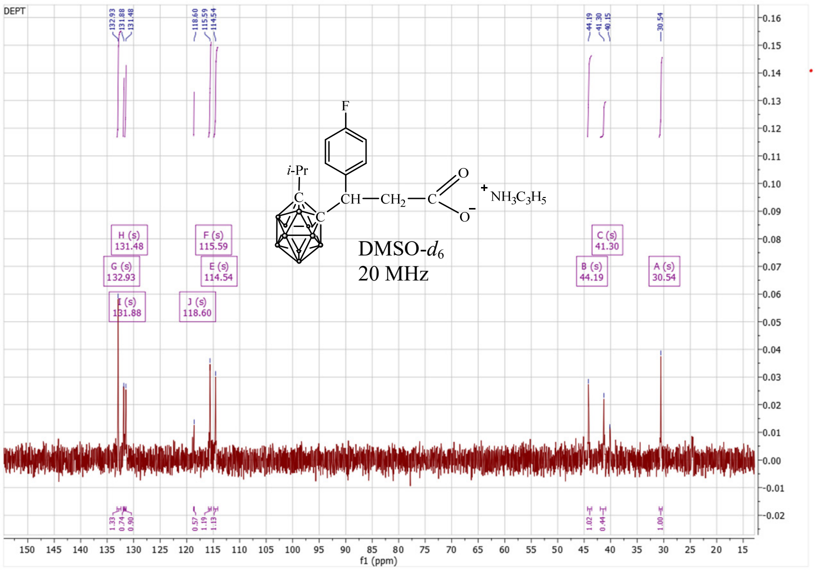

DEPT-90 NMR spectra of Allylammonium 3-(2-isopropyl-1,2-dicarba-closo-dodecaboran-1-yl)-3-(4-fluorophenyl)propanoate (18)

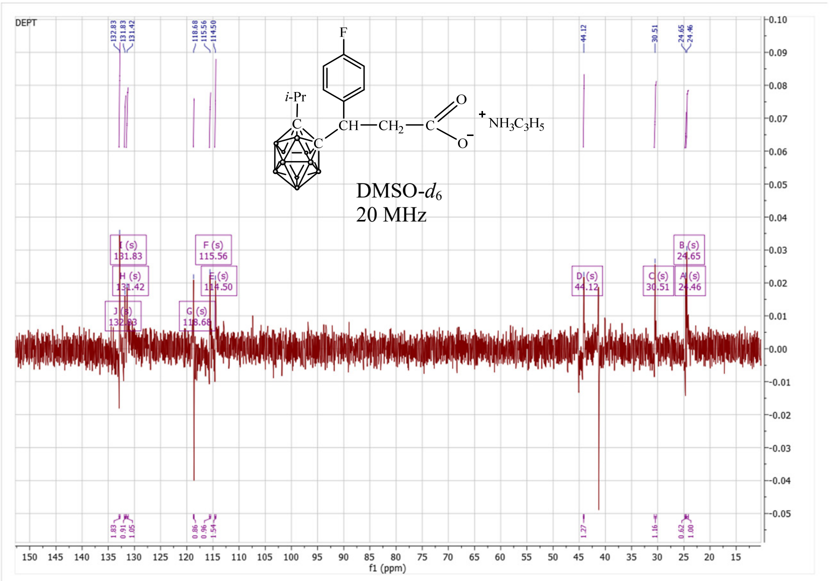

DEPT-135 NMR spectra of Allylammonium 3-(2-isopropyl-1,2-dicarba-closo-dodecaboran-1-yl)-3-(4-fluorophenyl)propanoate (18)

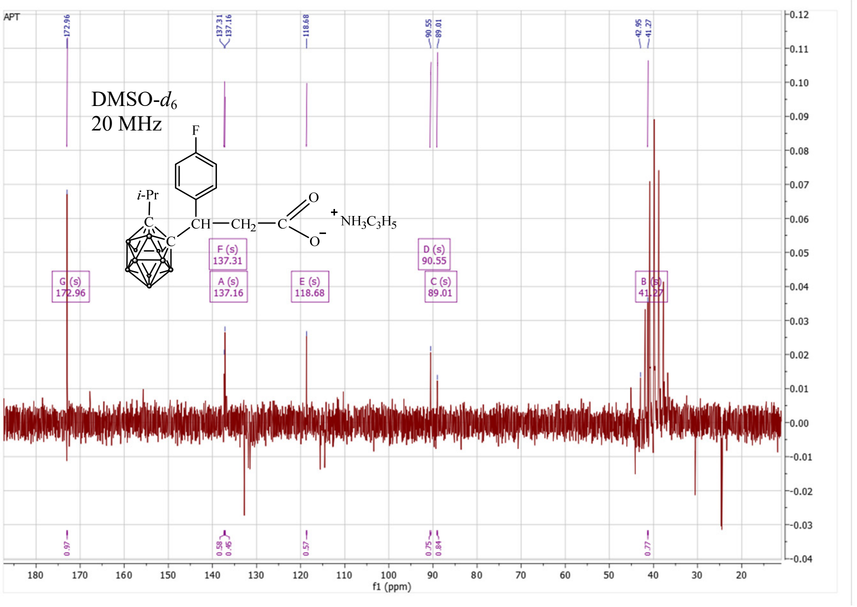

APT-WALTZ NMR spectra of Allylammonium 3-(2-isopropyl-1,2-dicarba-closo-dodecaboran-1-yl)-3-(4-fluorophenyl)propanoate (18)

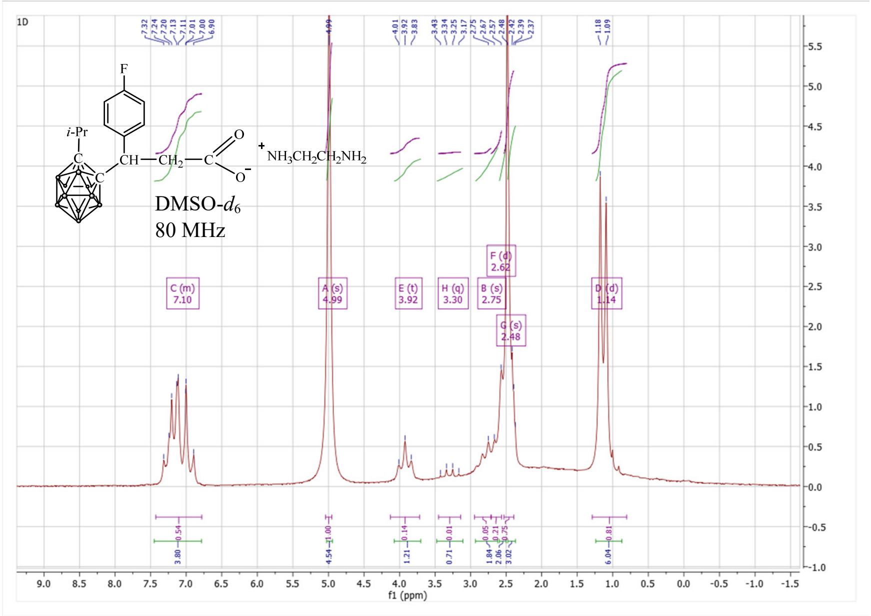

^{1}H NMR spectra of Ethan-1-amino-2-ammonium 3-(2-isopropyl-1,2-dicarba-closo-dodecaboran-1-yl)-3-(4-fluorophenyl)propanoate (19)

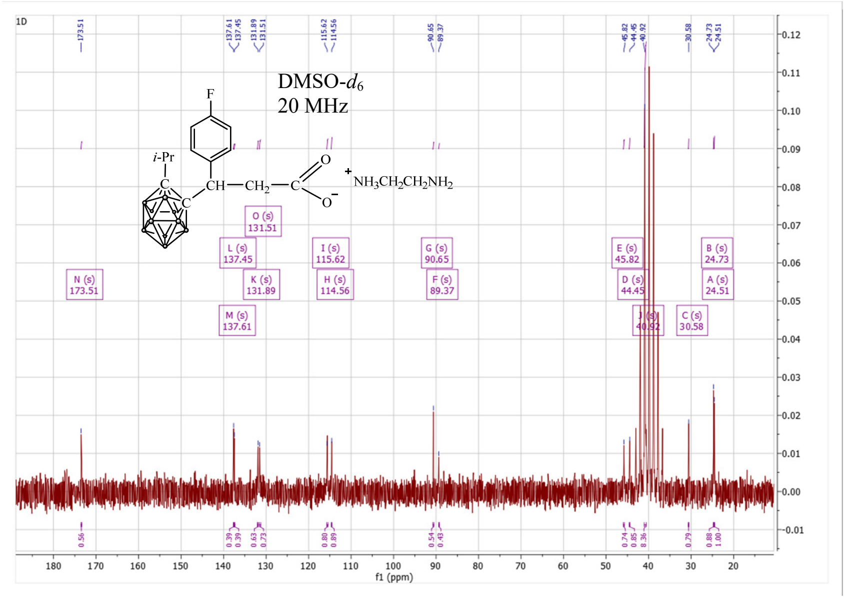

^{13}C NMR spectra of Ethan-1-amino-2-ammonium 3-(2-isopropyl-1,2-dicarba-closo-dodecaboran-1-yl)-3-(4-fluorophenyl)propanoate (19)

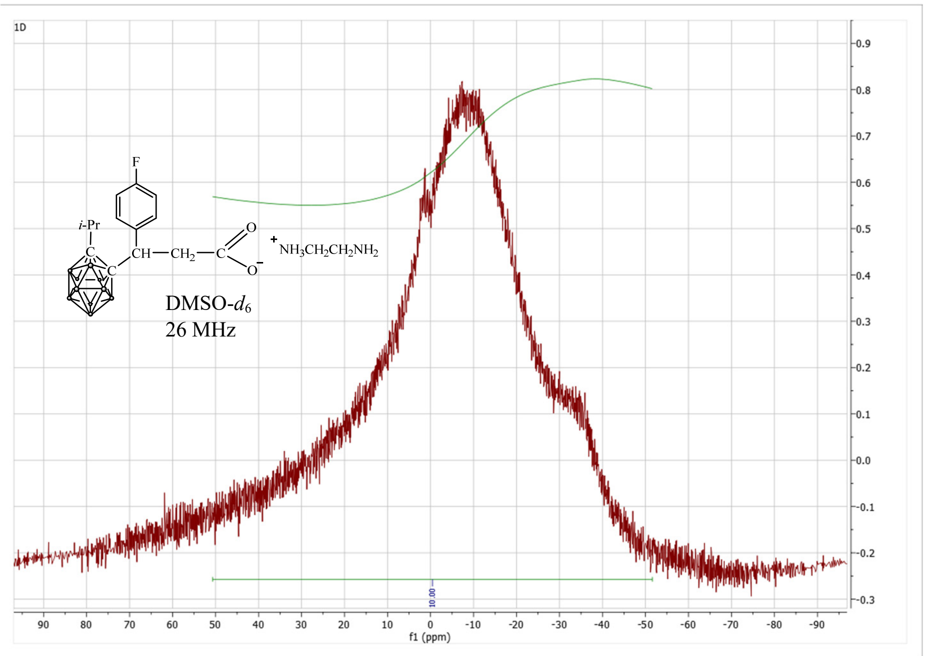

^{11}B NMR spectra of Ethan-1-amino-2-ammonium 3-(2-isopropyl-1,2-dicarba-closo-dodecaboran-1-yl)-3-(4-fluorophenyl)propanoate (19)

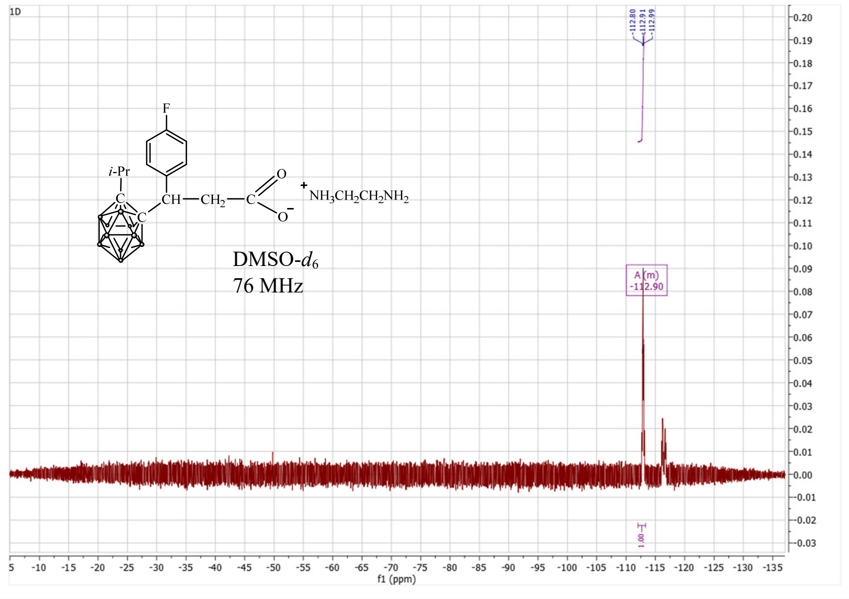

^{19}F NMR spectra of Ethan-1-amino-2-ammonium 3-(2-isopropyl-1,2-dicarba-closo-dodecaboran-1-yl)-3-(4-fluorophenyl)propanoate (19)

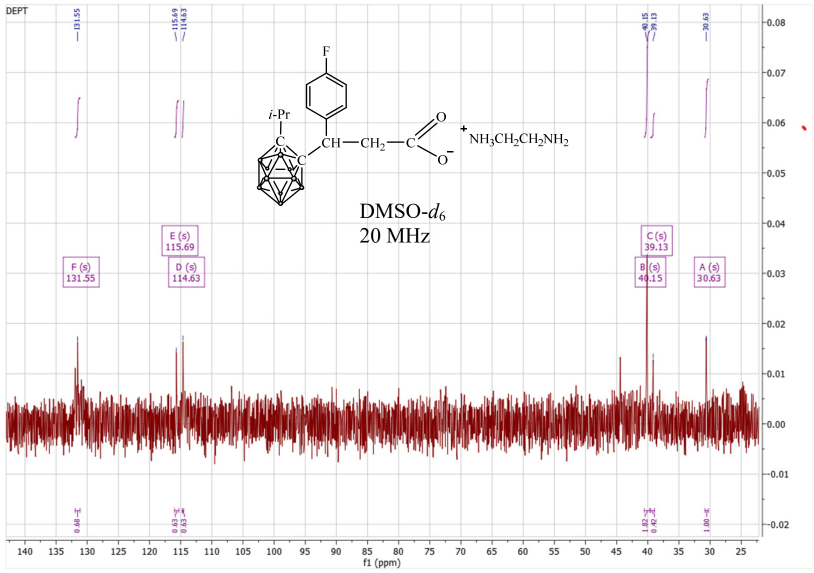

DEPT-90 NMR spectra of Ethan-1-amino-2-ammonium 3-(2-isopropyl-1,2-dicarba-closo-dodecaboran-1-yl)-3-(4-fluorophenyl)propanoate (19)

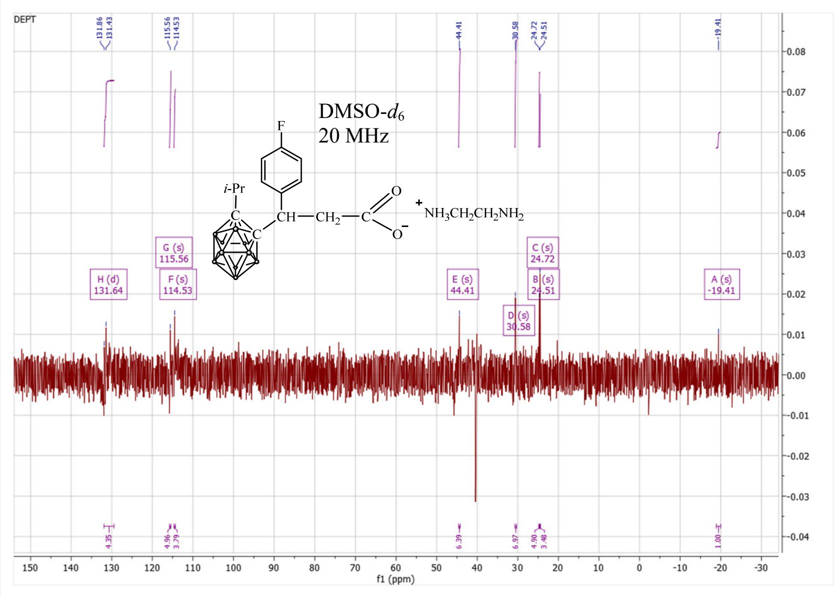

DEPT-135 NMR spectra of Ethan-1-amino-2-ammonium 3-(2-isopropyl-1,2-dicarba-closo-dodecaboran-1-yl)-3-(4-fluorophenyl)propanoate (19)

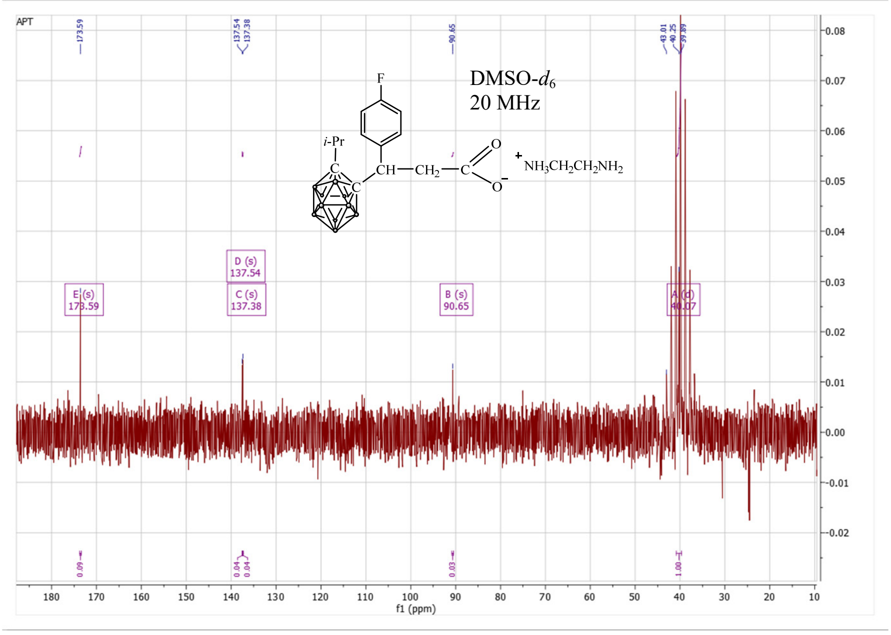

APT-WALTZ NMR spectra of Ethan-1-amino-2-ammonium 3-(2-isopropyl-1,2-dicarba-closo-dodecaboran-1-yl)-3-(4-fluorophenyl)propanoate (19)

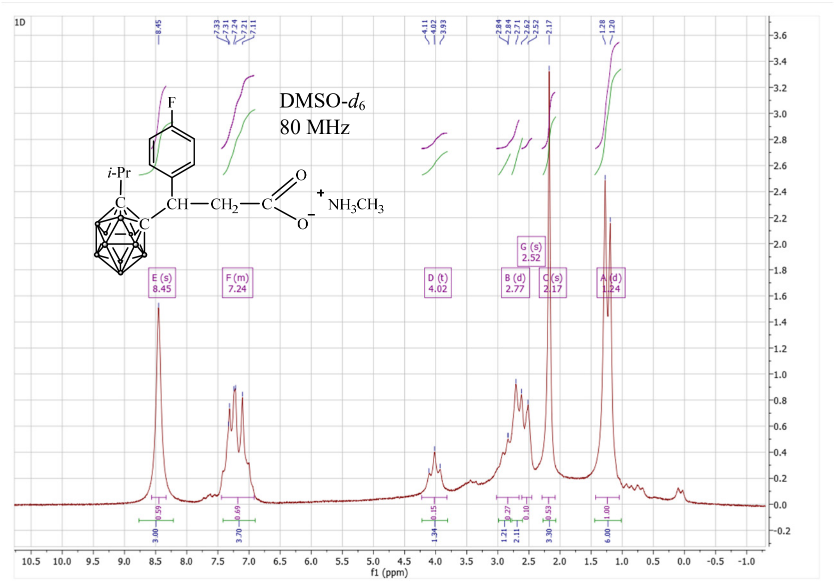

^{1}H NMR spectra of Methylammonium 3-(2-isopropyl-1,2-dicarba-closo-dodecaboran-1-yl)-3-(4-fluorophenyl)propanoate (20)

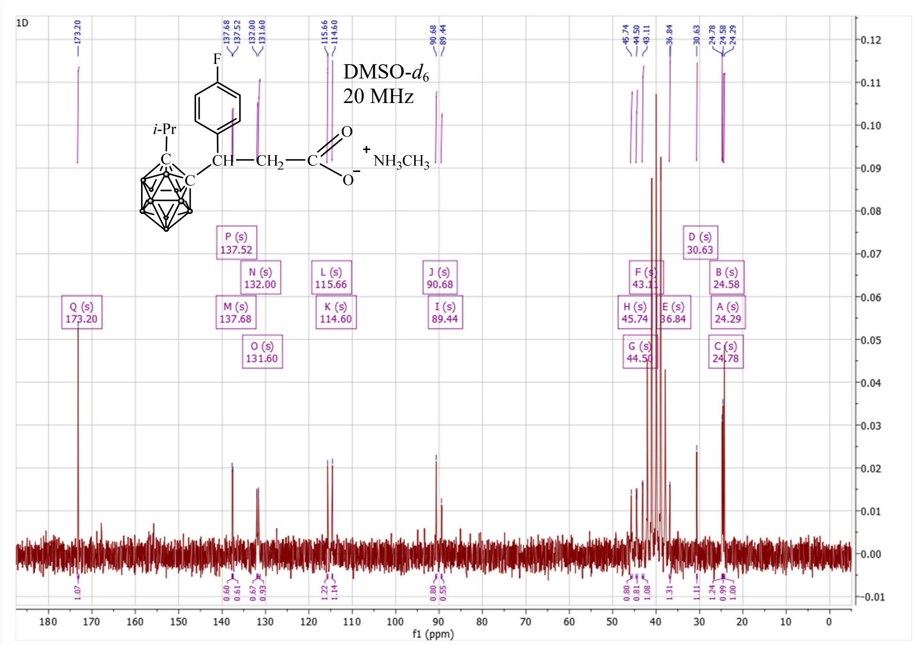

^{13}C NMR spectra of Methylammonium 3-(2-isopropyl-1,2-dicarba-closo-dodecaboran-1-yl)-3-(4-fluorophenyl)propanoate (20)

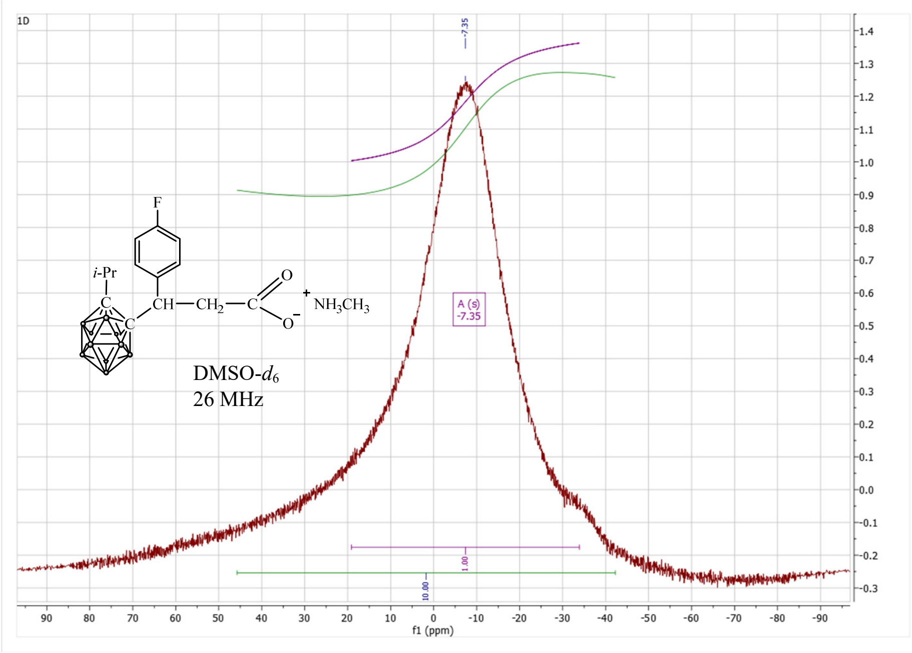

^{11}B NMR spectra of Methylammonium 3-(2-isopropyl-1,2-dicarba-closo-dodecaboran-1-yl)-3-(4-fluorophenyl)propanoate (20)

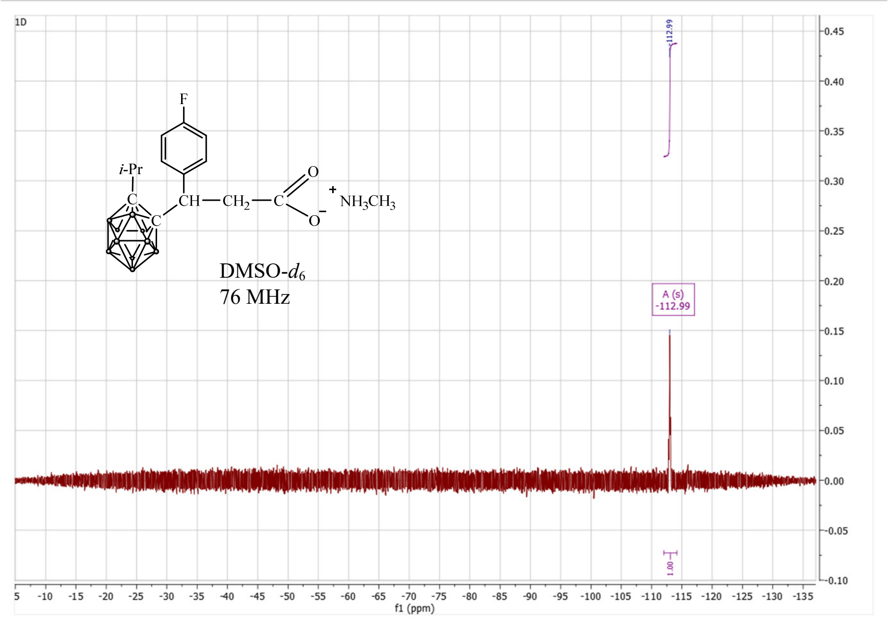

^{19}F NMR spectra of Methylammonium 3-(2-isopropyl-1,2-dicarba-closo-dodecaboran-1-yl)-3-(4-fluorophenyl)propanoate (20)

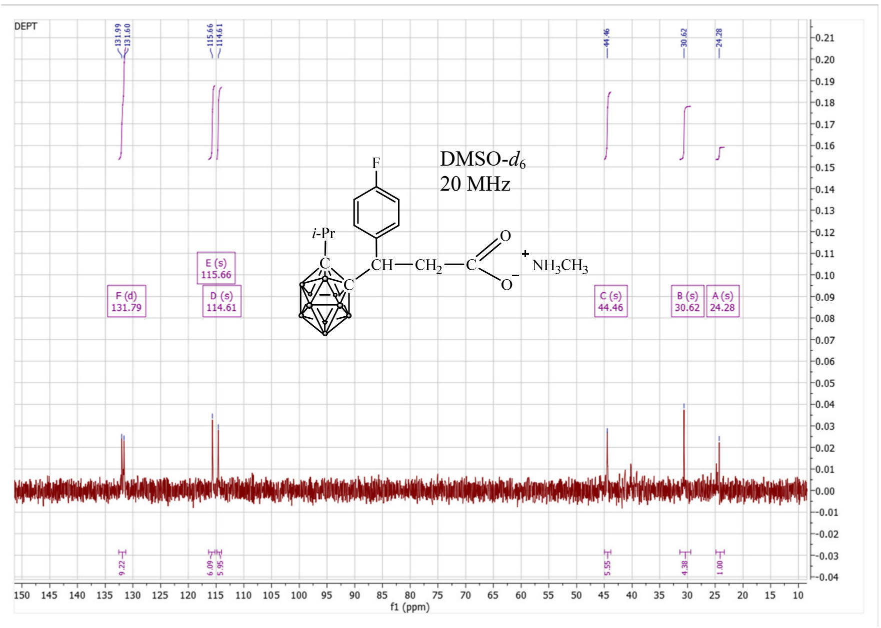

DEPT-90 NMR spectra of Methylammonium 3-(2-isopropyl-1,2-dicarba-closo-dodecaboran-1-yl)-3-(4-fluorophenyl)propanoate (20)

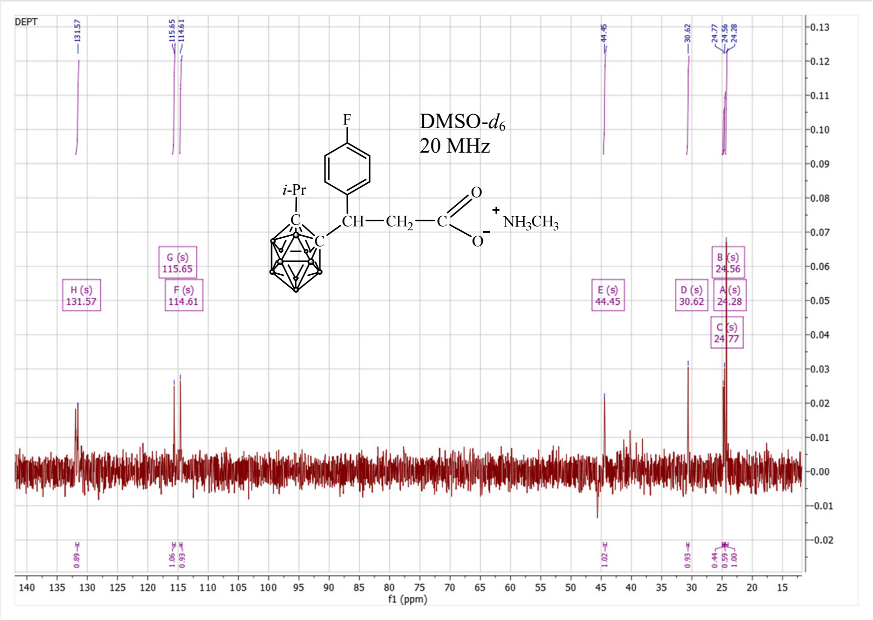

DEPT-135 NMR spectra of Methylammonium 3-(2-isopropyl-1,2-dicarba-closo-dodecaboran-1-yl)-3-(4-fluorophenyl)propanoate (20)

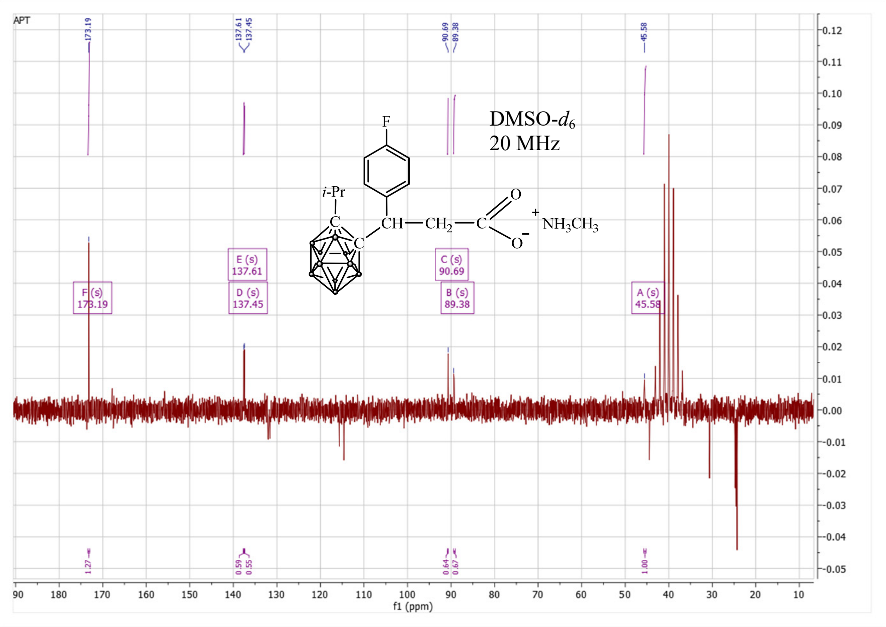

APT-WALTZ NMR spectra of Methylammonium 3-(2-isopropyl-1,2-dicarba-closo-dodecaboran-1-yl)-3-(4-fluorophenyl)propanoate (20)

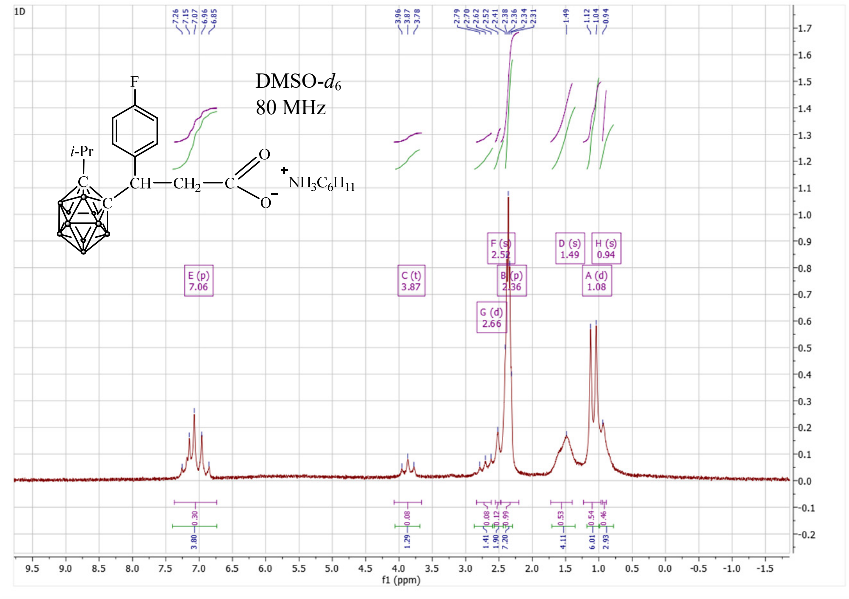

^{1}H NMR spectra of Cyclohexylammonium 3-(2-isopropyl-1,2-dicarba-closo-dodecaboran-1-yl)-3-(4-fluorophenyl)propanoate (21)

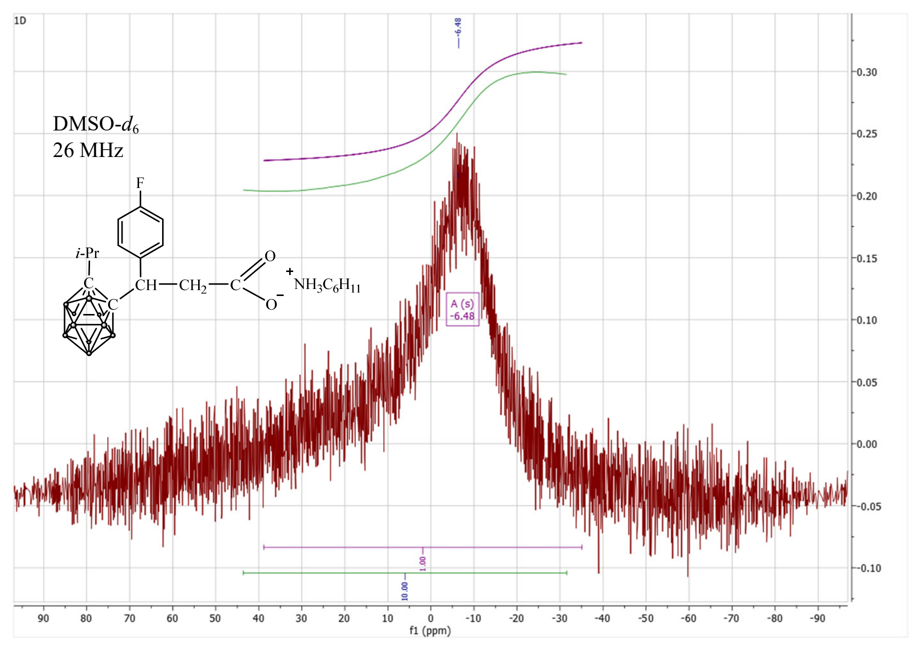

^{11}B NMR spectra of Cyclohexylammonium 3-(2-isopropyl-1,2-dicarba-closo-dodecaboran-1-yl)-3-(4-fluorophenyl)propanoate (21)

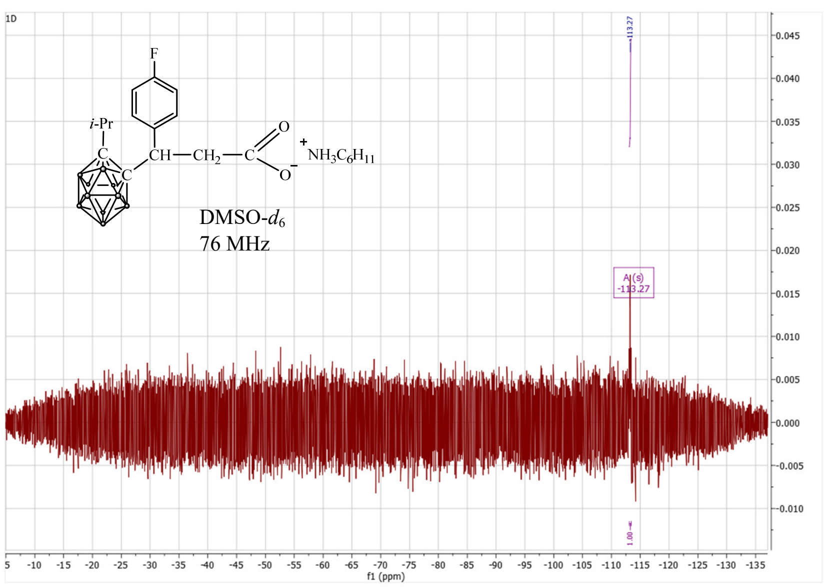

^{19}F NMR spectra of Cyclohexylammonium 3-(2-isopropyl-1,2-dicarba-closo-dodecaboran-1-yl)-3-(4-fluorophenyl)propanoate (21)

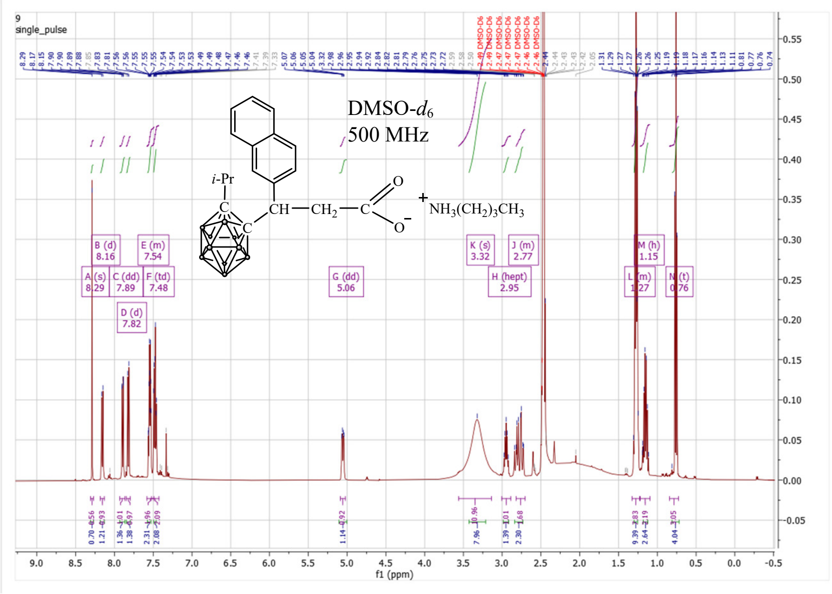

^{1}H NMR spectra of Butylammonium 3-(2-isopropyl-1,2-dicarba-closo-dodecaboran-1-yl)-3-(naphthalene-1-yl)propanoate (22)

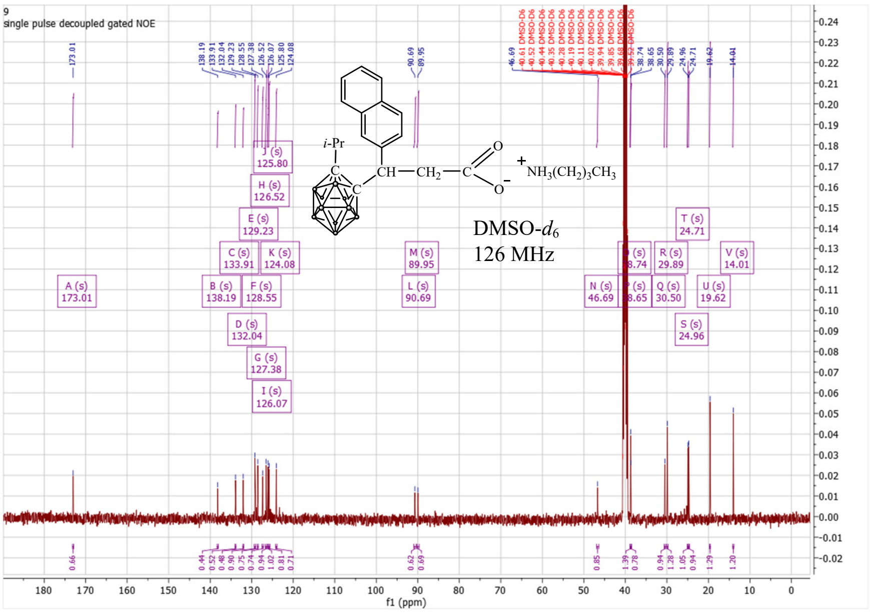

^{13}C NMR spectra of Butylammonium 3-(2-isopropyl-1,2-dicarba-closo-dodecaboran-1-yl)-3-(naphthalene-1-yl)propanoate (22)

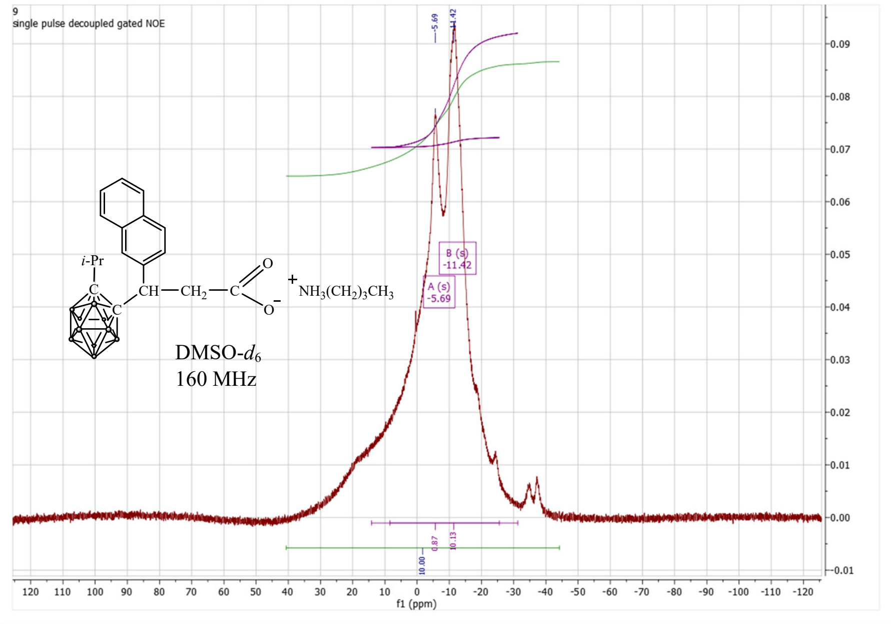

^{11}B NMR spectra of Butylammonium 3-(2-isopropyl-1,2-dicarba-closo-dodecaboran-1-yl)-3-(naphthalene-1-yl)propanoate (22)

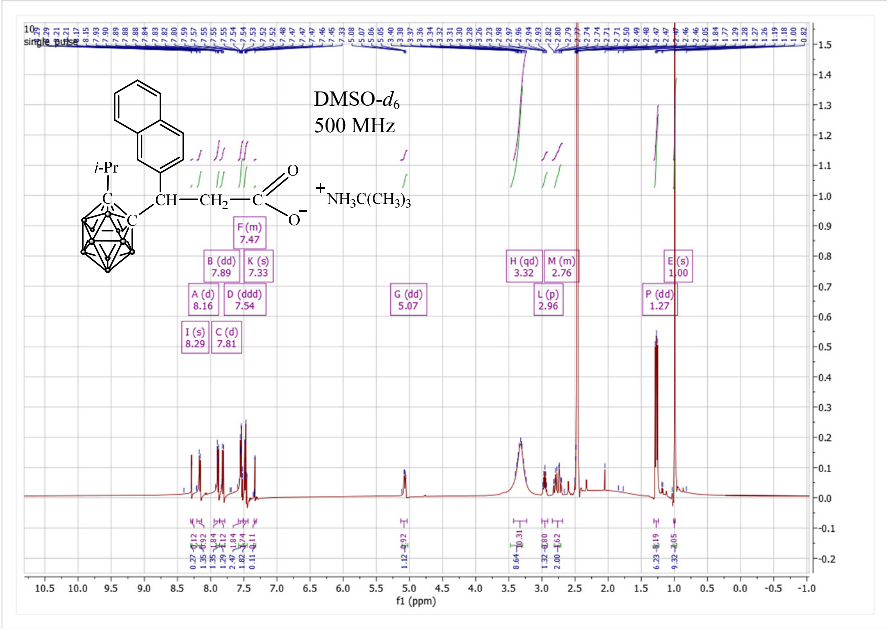

^{1}H NMR spectra of Tert-butylammonium 3-(2-isopropyl-1,2-dicarba-closo-dodecaboran-1-yl)-3-(naphthalene-1-yl)propanoate (23)

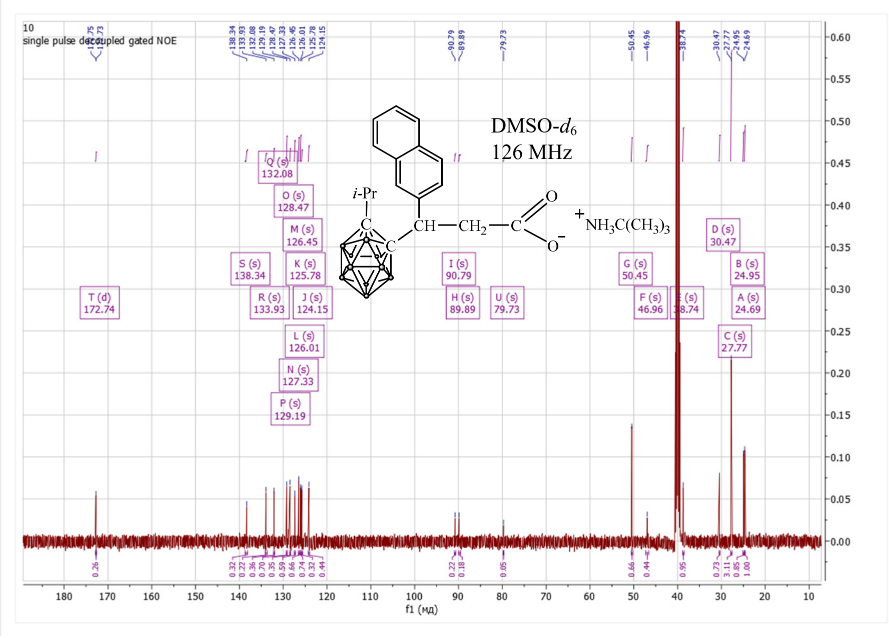

^{13}C NMR spectra of Tert-butylammonium 3-(2-isopropyl-1,2-dicarba-closo-dodecaboran-1-yl)-3-(naphthalene-1-yl)propanoate (23)

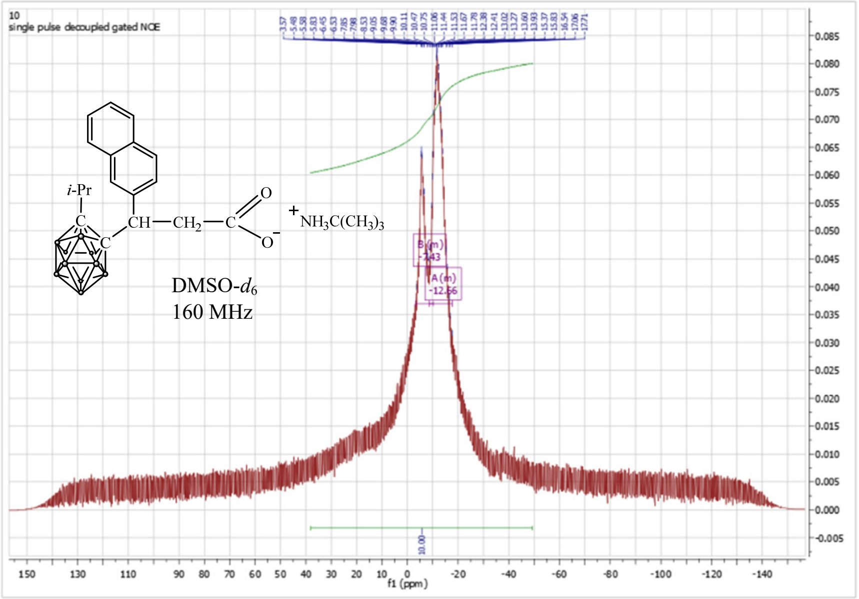

^{11}B NMR spectra of Tert-butylammonium 3-(2-isopropyl-1,2-dicarba-closo-dodecaboran-1-yl)-3-(naphthalene-1-yl)propanoate (23)

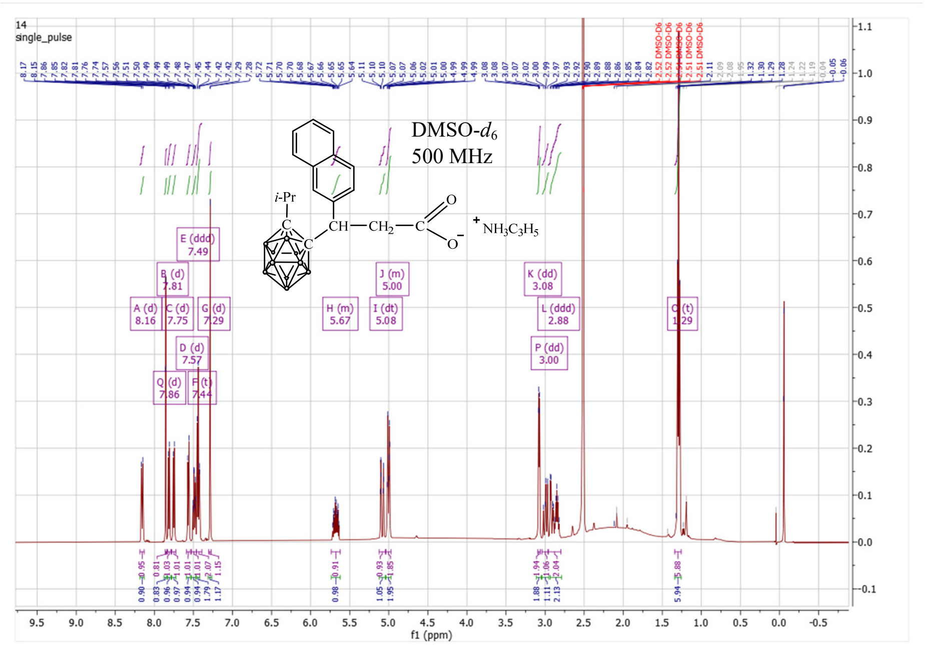

^{1}H NMR spectra of Allylammonium 3-(2-isopropyl-1,2-dicarba-closo-dodecaboran-1-yl)-3-(naphthalene-1-yl)propanoate (24)

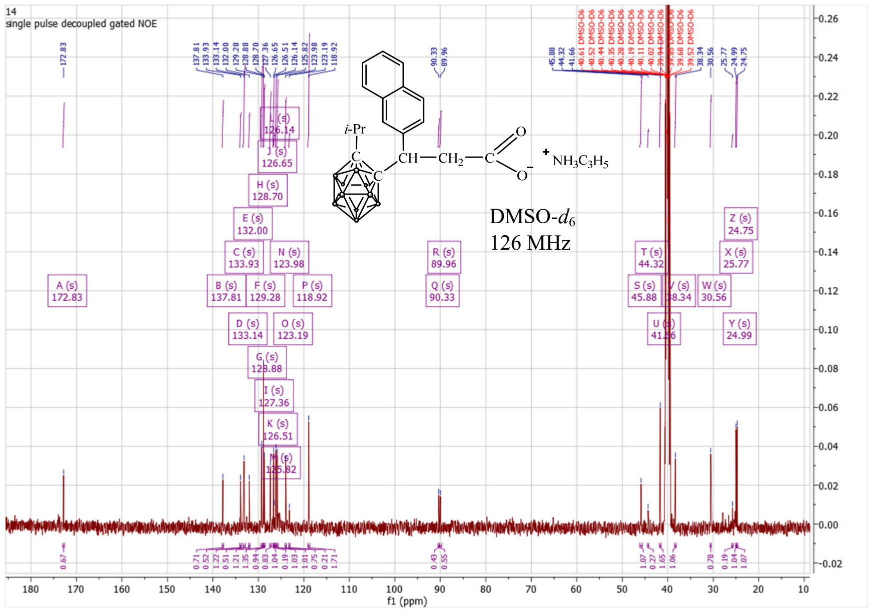

^{13}C NMR spectra of Allylammonium 3-(2-isopropyl-1,2-dicarba-closo-dodecaboran-1-yl)-3-(naphthalene-1-yl)propanoate (24)

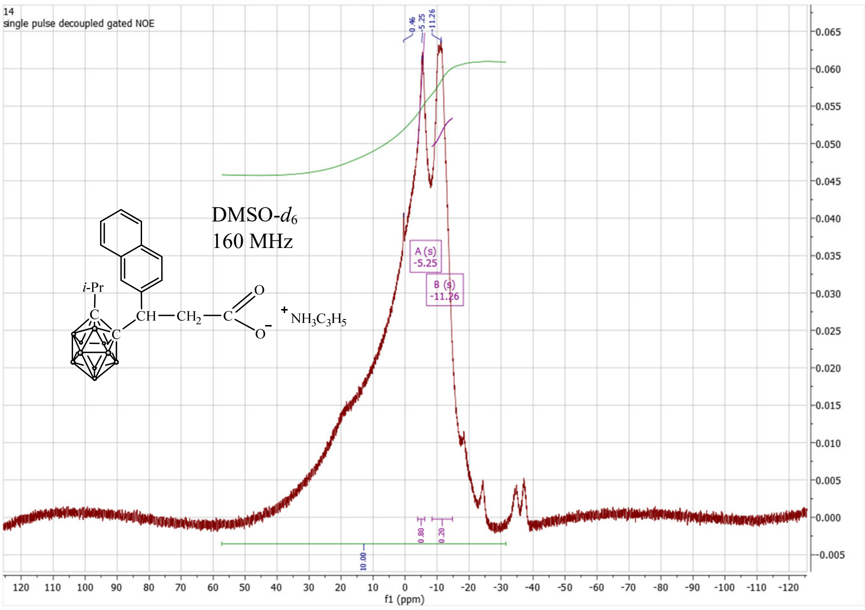

^{11}B NMR spectra of Allylammonium 3-(2-isopropyl-1,2-dicarba-closo-dodecaboran-1-yl)-3-(naphthalene-1-yl)propanoate (24)

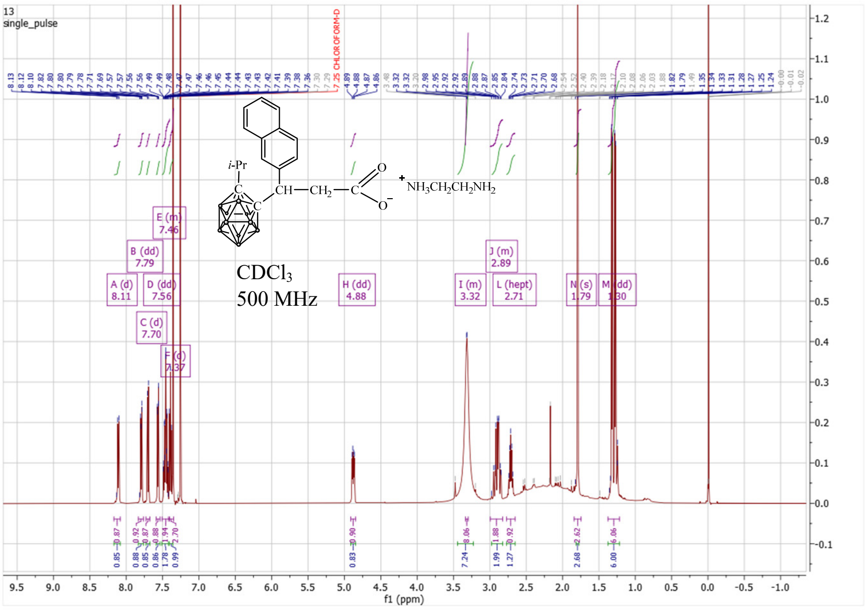

^{1}H NMR spectra of Ethan-1-amino-2-ammonium 3-(2-isopropyl-1,2-dicarba-closo-dodecaboran-1-yl)-3-(naphthalene-1-yl)propanoate (25)

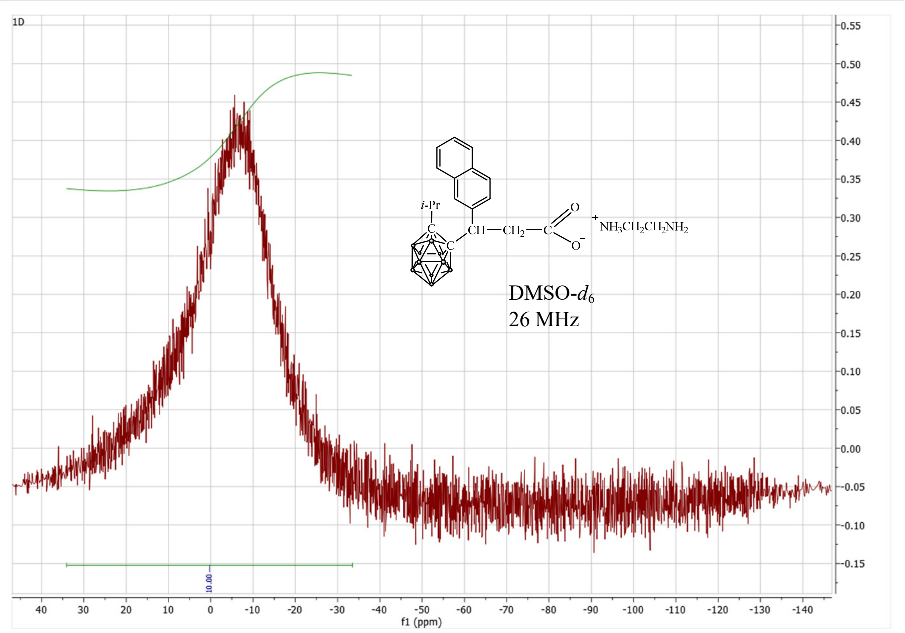

^{11}B NMR spectra of Ethan-1-amino-2-ammonium 3-(2-isopropyl-1,2-dicarba-closo-dodecaboran-1-yl)-3-(naphthalene-1-yl)propanoate (25)

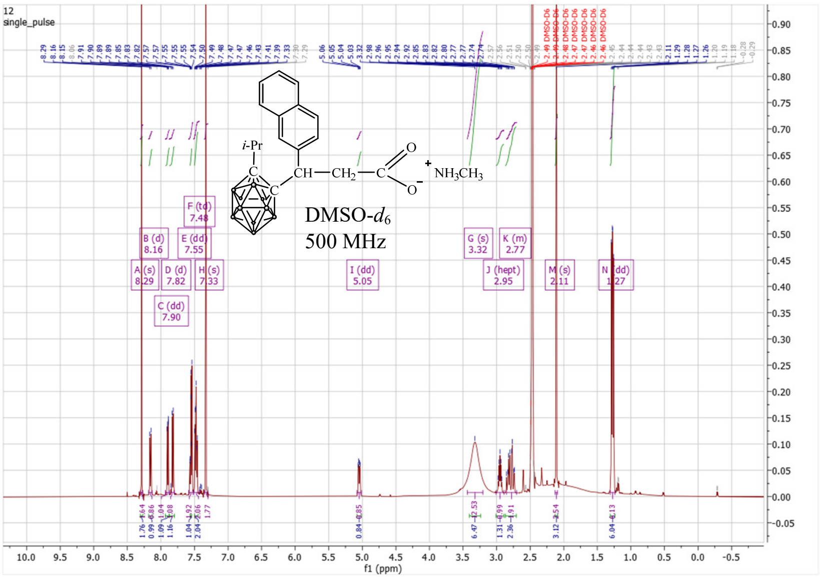

^{1}H NMR spectra of Methylammonium 3-(2-isopropyl-1,2-dicarba-closo-dodecaboran-1-yl)-3-(naphthalene-1-yl)propanoate (26)

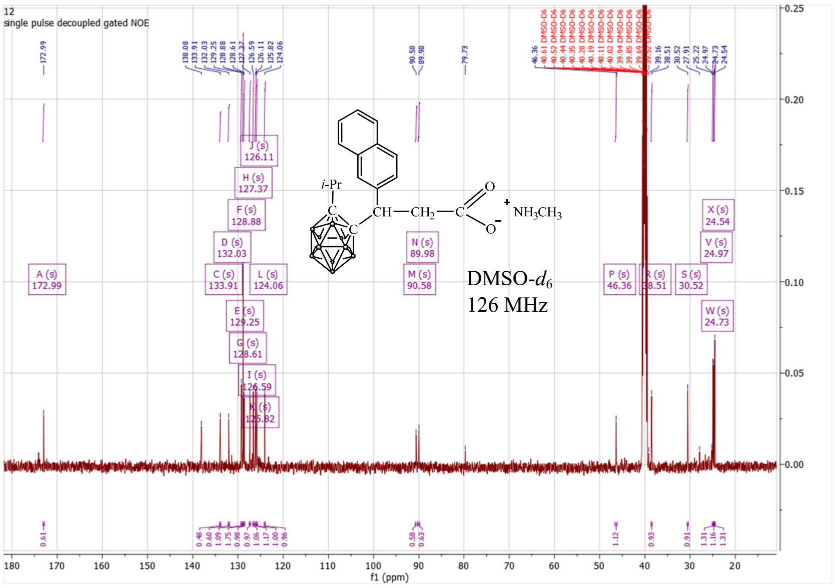

^{13}C NMR spectra of Methylammonium 3-(2-isopropyl-1,2-dicarba-closo-dodecaboran-1-yl)-3-(naphthalene-1-yl)propanoate (26)

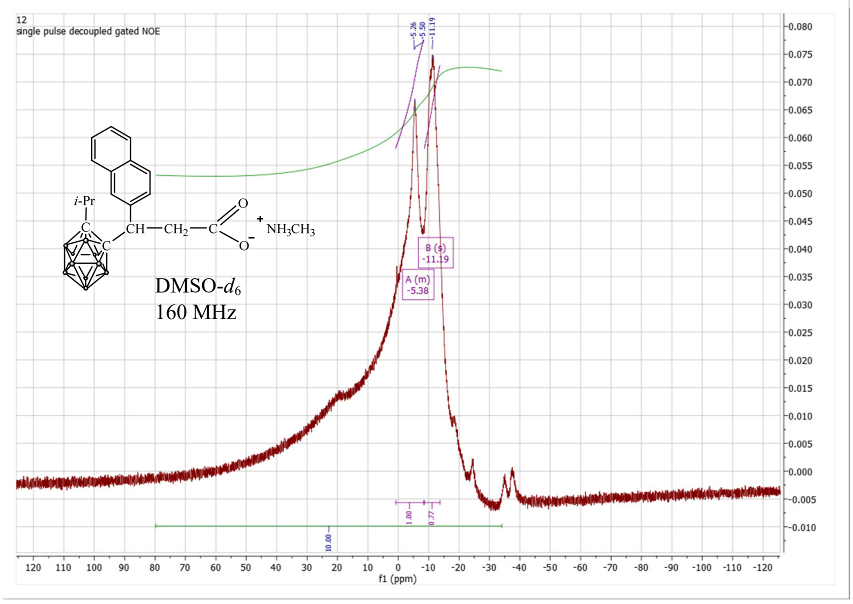

^{11}B NMR spectra of Methylammonium 3-(2-isopropyl-1,2-dicarba-closo-dodecaboran-1-yl)-3-(naphthalene-1-yl)propanoate (26)

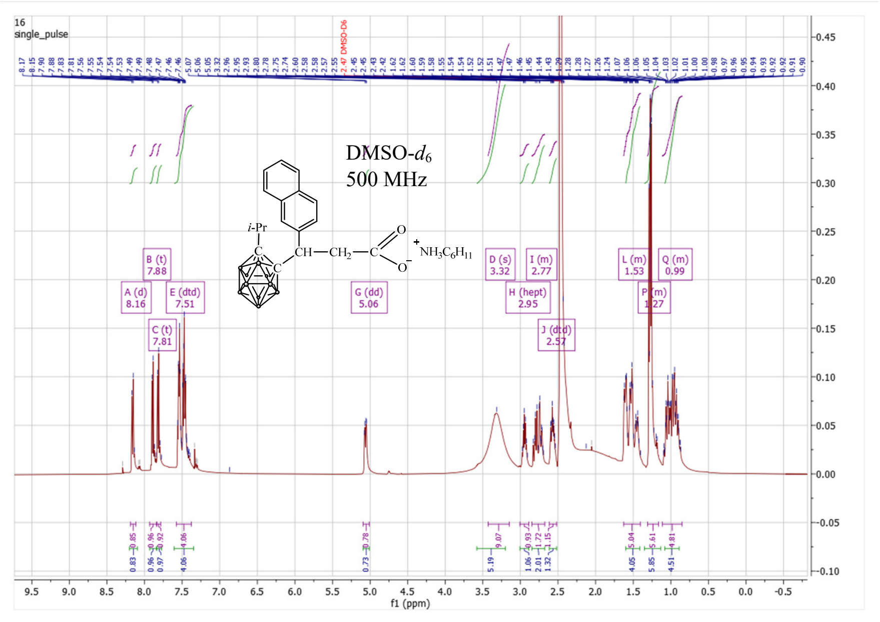

^{1}H NMR spectra of Cyclohexylammonium 3-(2-isopropyl-1,2-dicarba-closo-dodecaboran-1-yl)-3-(naphthalene-1-yl)propanoate (27)

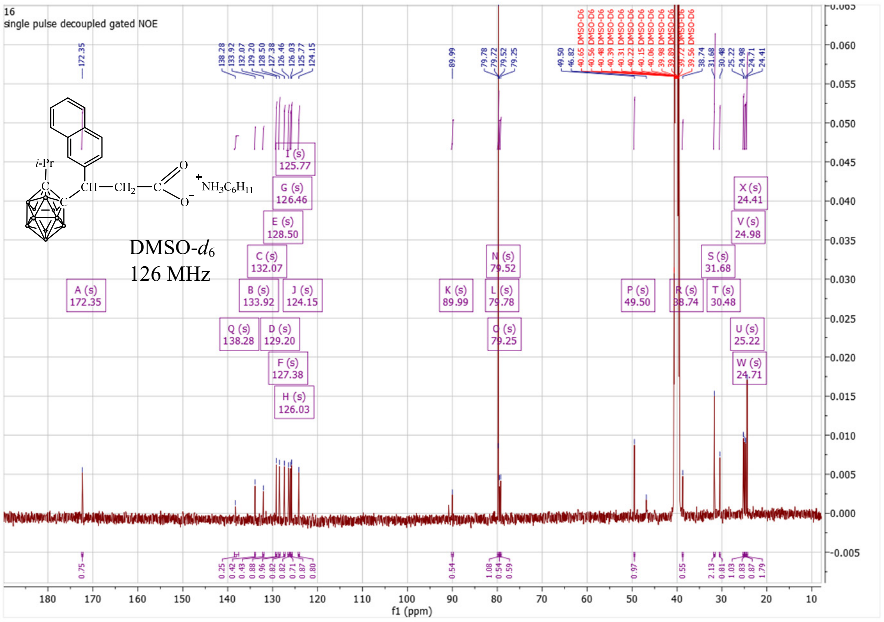

^{13}C NMR spectra of Cyclohexylammonium 3-(2-isopropyl-1,2-dicarba-closo-dodecaboran-1-yl)-3-(naphthalene-1-yl)propanoate (27)

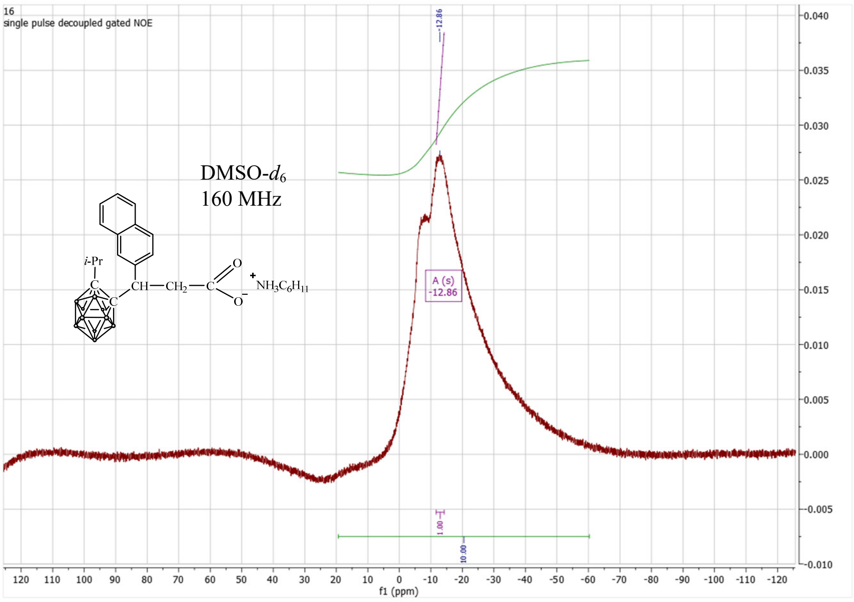

^{11}B NMR spectra of Cyclohexylammonium 3-(2-isopropyl-1,2-dicarba-closo-dodecaboran-1-yl)-3-(naphthalene-1-yl)propanoate (27)

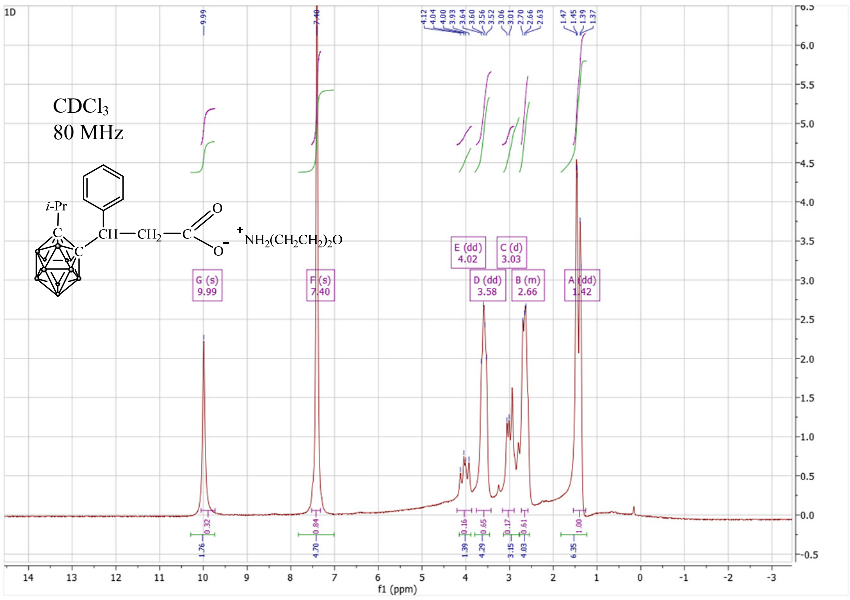

^{1}H NMR spectra of Morpholine 3-(2-isopropyl-1,2-dicarba-closo-dodecaboran-1-yl)-3-phenylpropanoate salt (28)

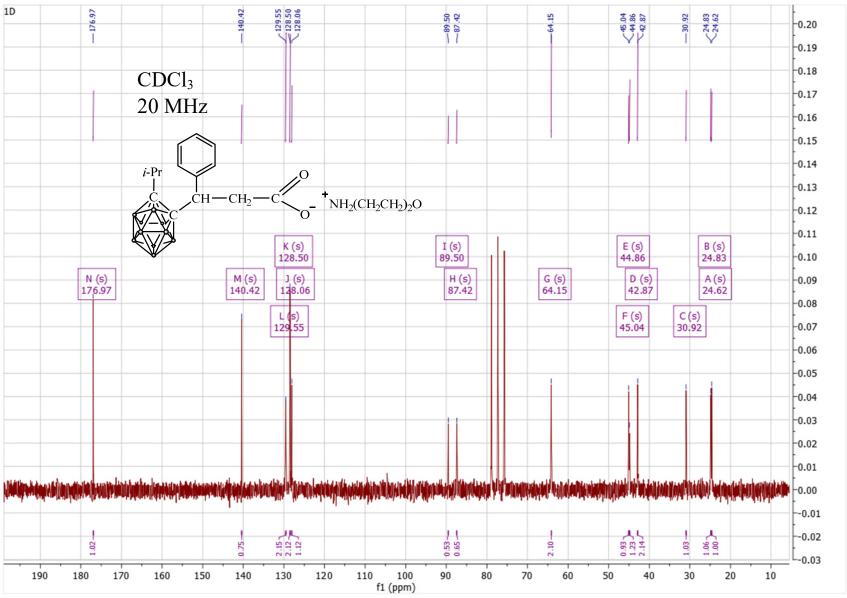

^{13}C NMR spectra of Morpholine 3-(2-isopropyl-1,2-dicarba-closo-dodecaboran-1-yl)-3-phenylpropanoate salt (28)

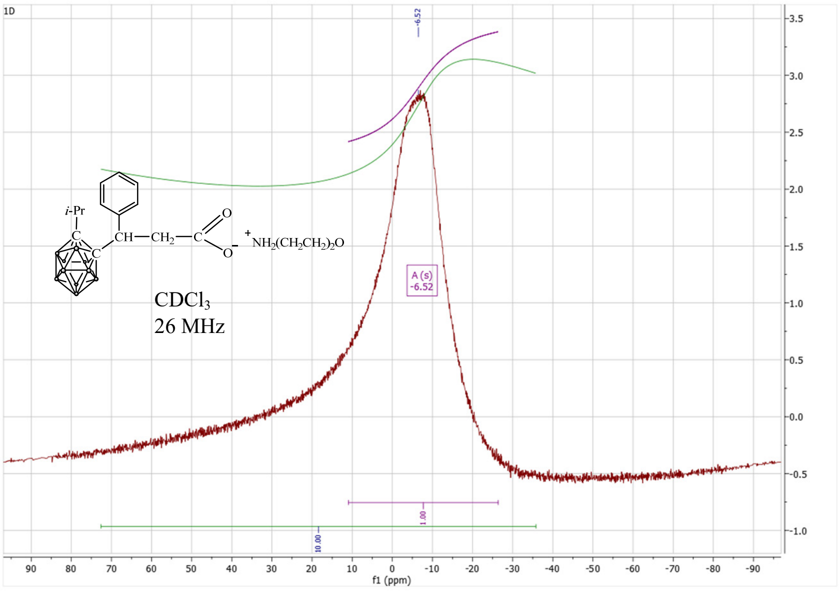

^{11}B NMR spectra of Morpholine 3-(2-isopropyl-1,2-dicarba-closo-dodecaboran-1-yl)-3-phenylpropanoate salt (28)

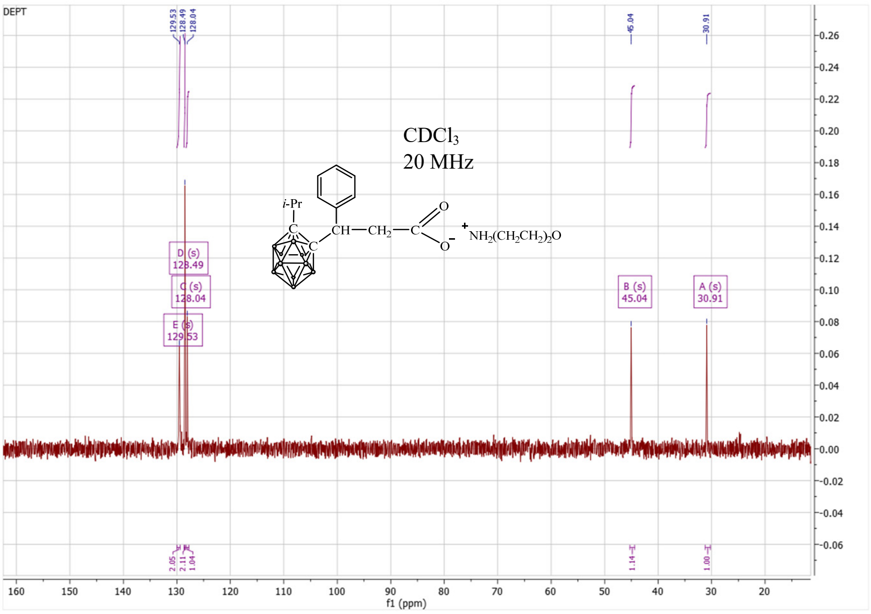

DEPT-90 NMR spectra of Morpholine 3-(2-isopropyl-1,2-dicarba-closo-dodecaboran-1-yl)-3-phenylpropanoate salt (28)

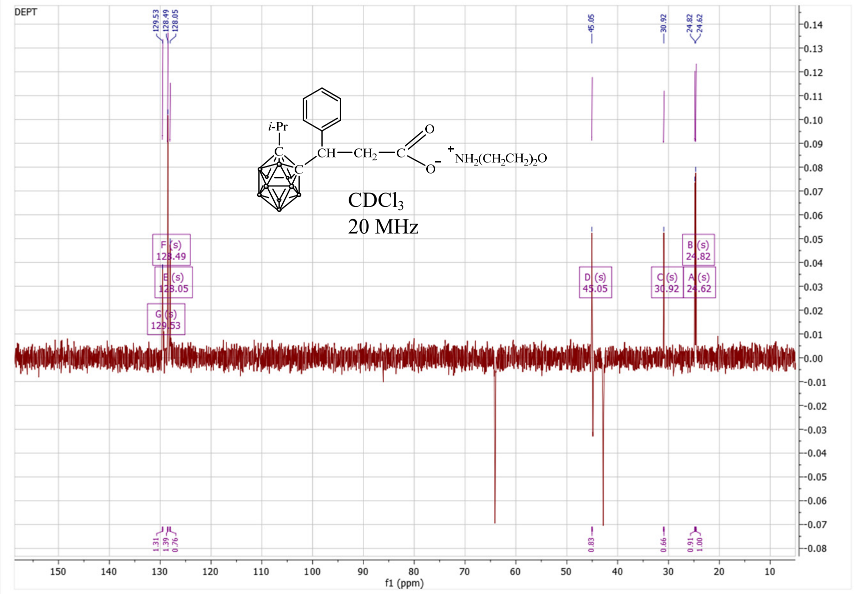

DEPT-135 NMR spectra of Morpholine 3-(2-isopropyl-1,2-dicarba-closo-dodecaboran-1-yl)-3-phenylpropanoate salt (28)

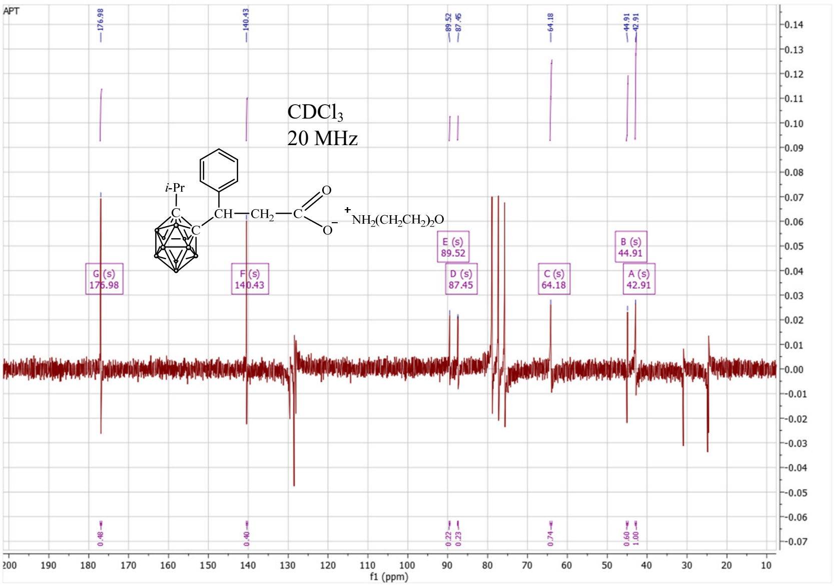

APT-WALTZ NMR spectra of Morpholine 3-(2-isopropyl-1,2-dicarba-closo-dodecaboran-1-yl)-3-phenylpropanoate salt (28)

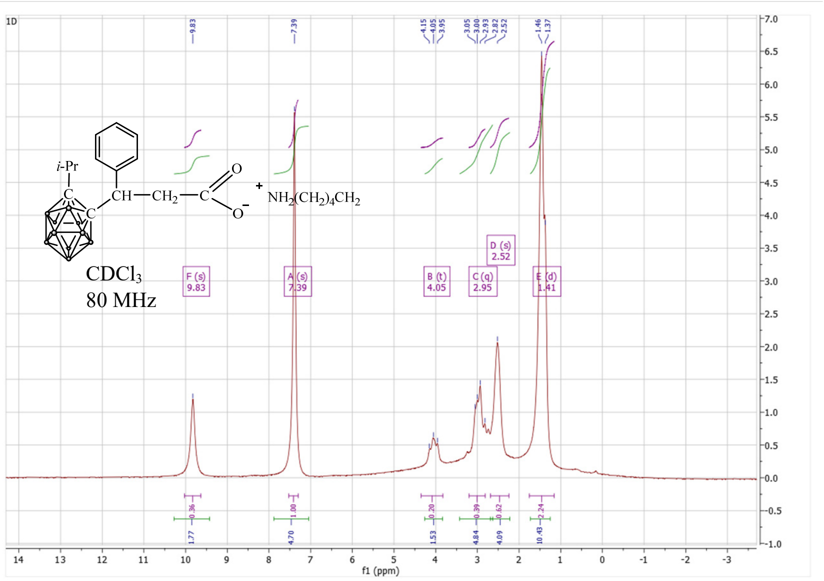

^{1}H NMR spectra of Piperidine 3-(2-isopropyl-1,2-dicarba-closo-dodecaboran-1-yl)-3-phenylpropanoate salt (29)

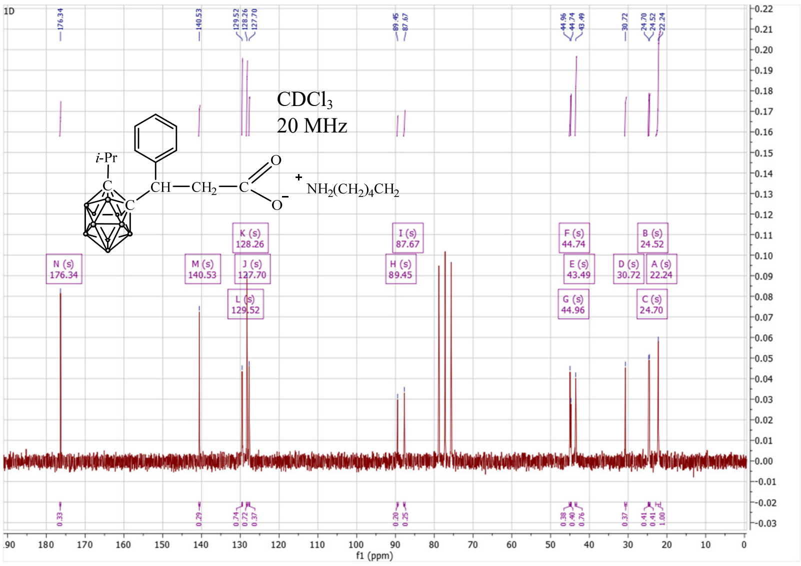

^{13}C NMR spectra of Piperidine 3-(2-isopropyl-1,2-dicarba-closo-dodecaboran-1-yl)-3-phenylpropanoate salt (29)

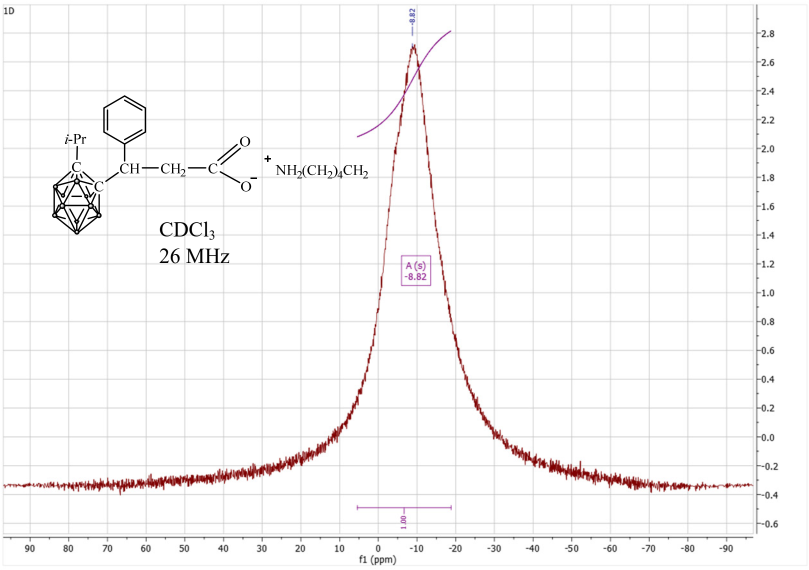

^{11}B NMR spectra of Piperidine 3-(2-isopropyl-1,2-dicarba-closo-dodecaboran-1-yl)-3-phenylpropanoate salt (29)

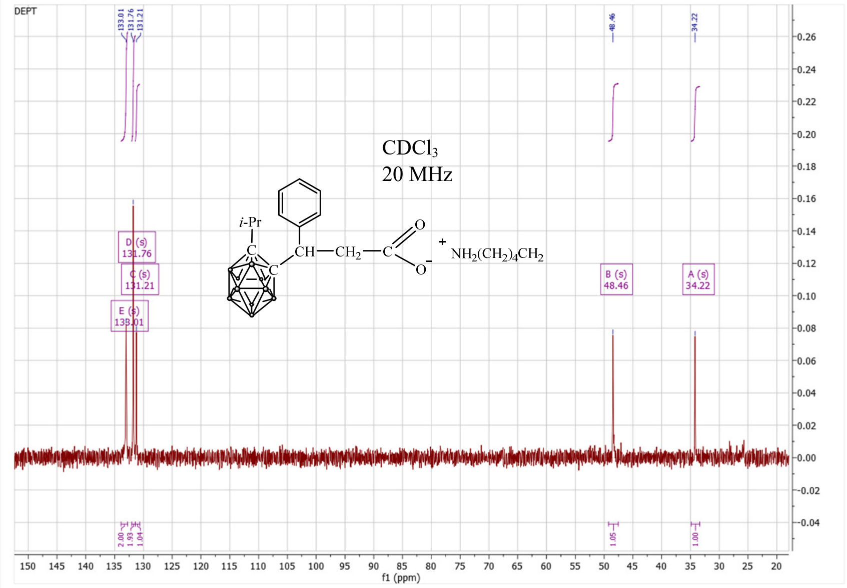

DEPT-90 NMR spectra of Piperidine 3-(2-isopropyl-1,2-dicarba-closo-dodecaboran-1-yl)-3-phenylpropanoate salt (29)

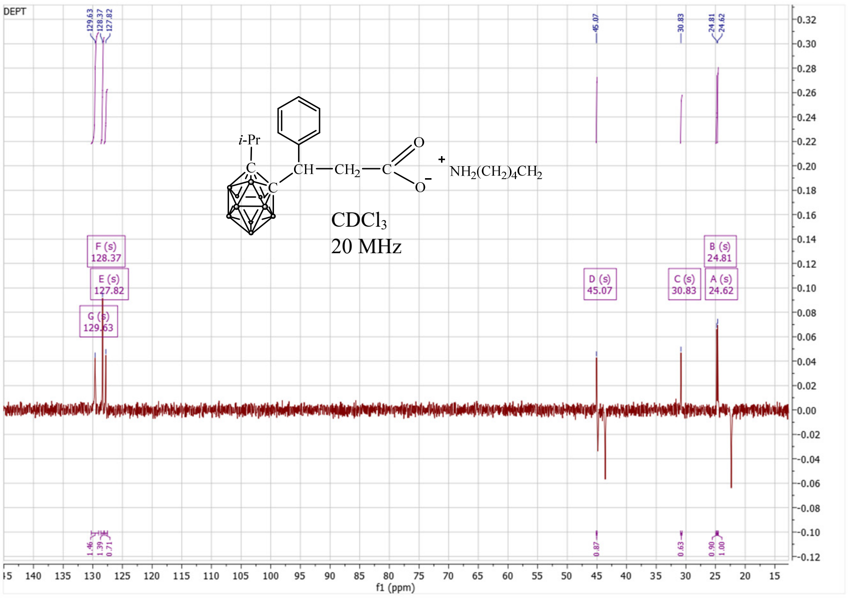

DEPT-135 NMR spectra of Piperidine 3-(2-isopropyl-1,2-dicarba-closo-dodecaboran-1-yl)-3-phenylpropanoate salt (29)

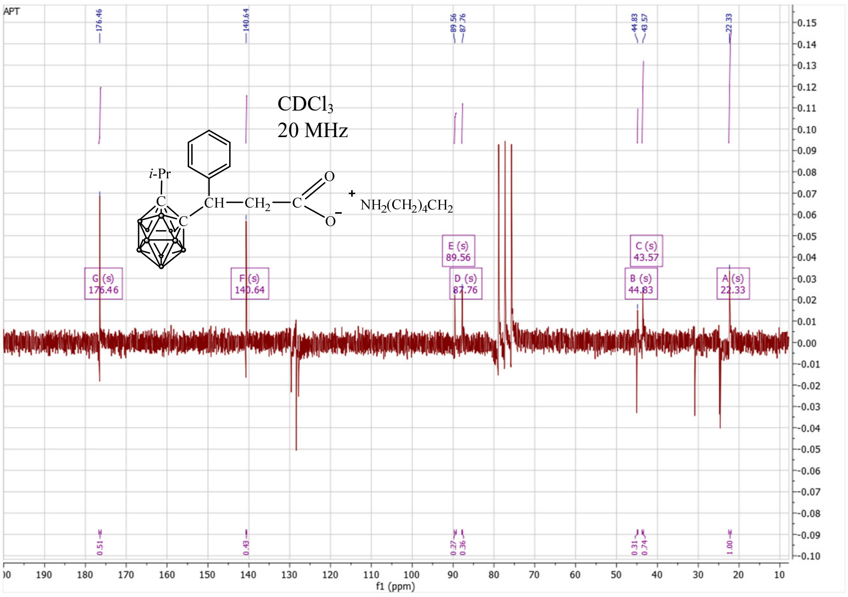

APT-WALTZ NMR spectra of Piperidine 3-(2-isopropyl-1,2-dicarba-closo-dodecaboran-1-yl)-3-phenylpropanoate salt (29)

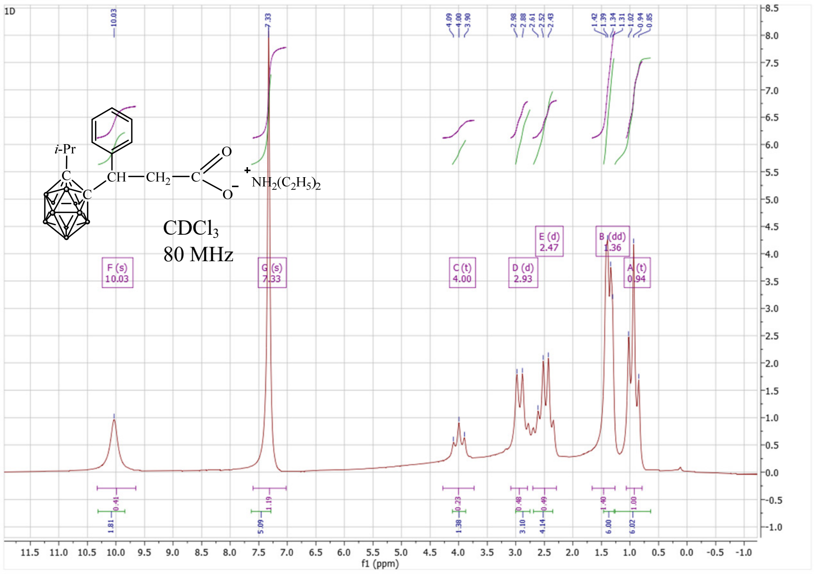

^{1}H NMR spectra of Diethylammonium 3-(2-isopropyl-1,2-dicarba-closo-dodecaboran-1-yl)-3-phenylpropanoate salt (30)

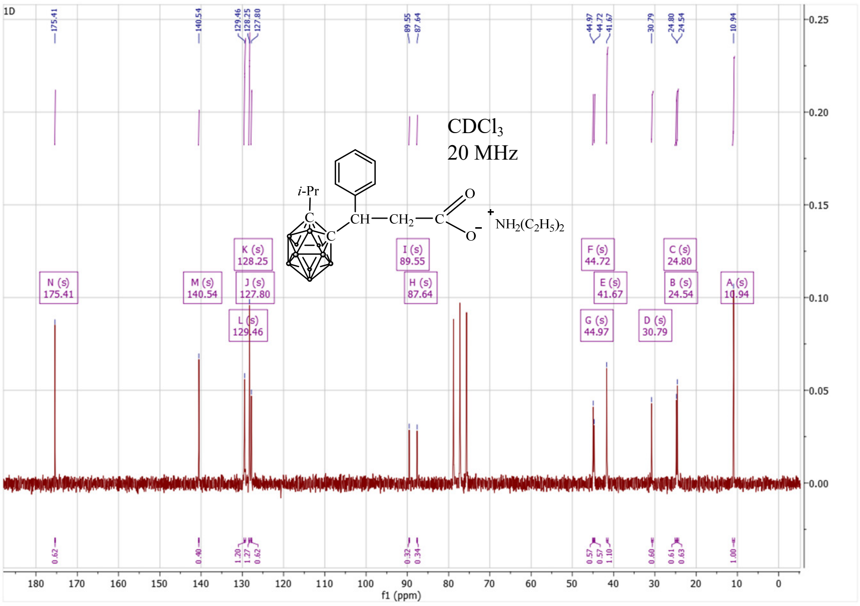

^{13}C NMR spectra of Diethylammonium 3-(2-isopropyl-1,2-dicarba-closo-dodecaboran-1-yl)-3-phenylpropanoate salt (30)

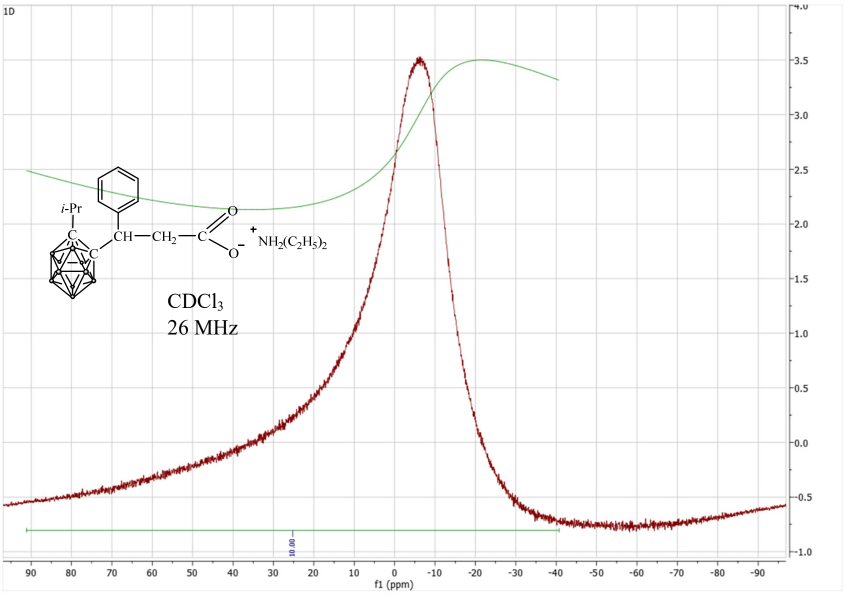

^{11}B NMR spectra of Diethylammonium 3-(2-isopropyl-1,2-dicarba-closo-dodecaboran-1-yl)-3-phenylpropanoate salt (30)

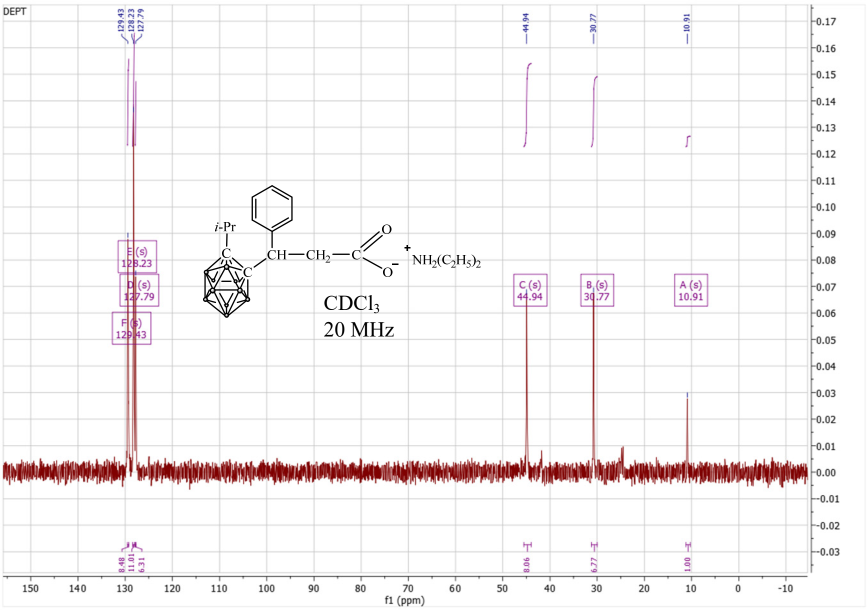

DEPT-90 NMR spectra of Diethylammonium 3-(2-isopropyl-1,2-dicarba-closo-dodecaboran-1-yl)-3-phenylpropanoate salt (30)

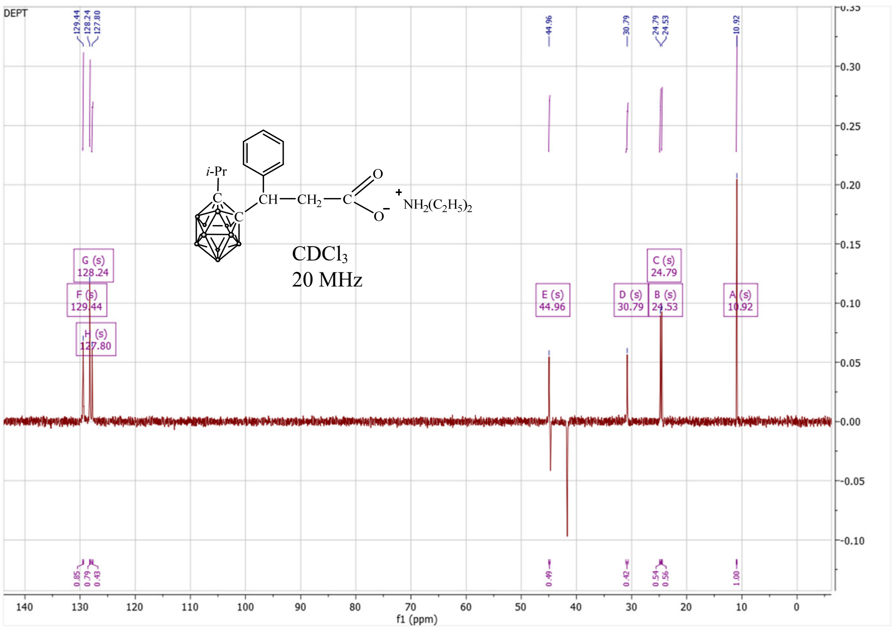

DEPT-135 NMR spectra of Diethylammonium 3-(2-isopropyl-1,2-dicarba-closo-dodecaboran-1-yl)-3-phenylpropanoate salt (30)

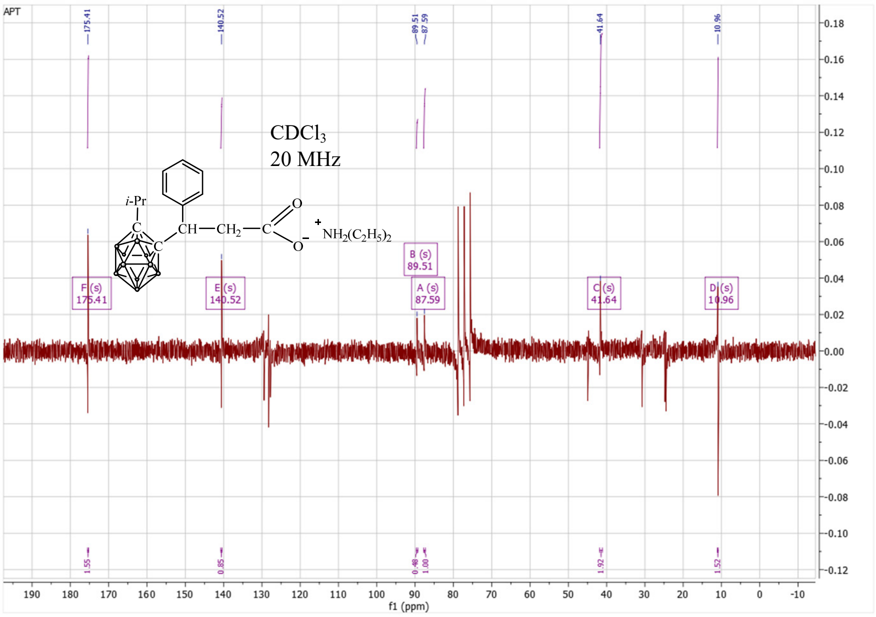

APT-WALTZ NMR spectra of Diethylammonium 3-(2-isopropyl-1,2-dicarba-closo-dodecaboran-1-yl)-3-phenylpropanoate salt (30)

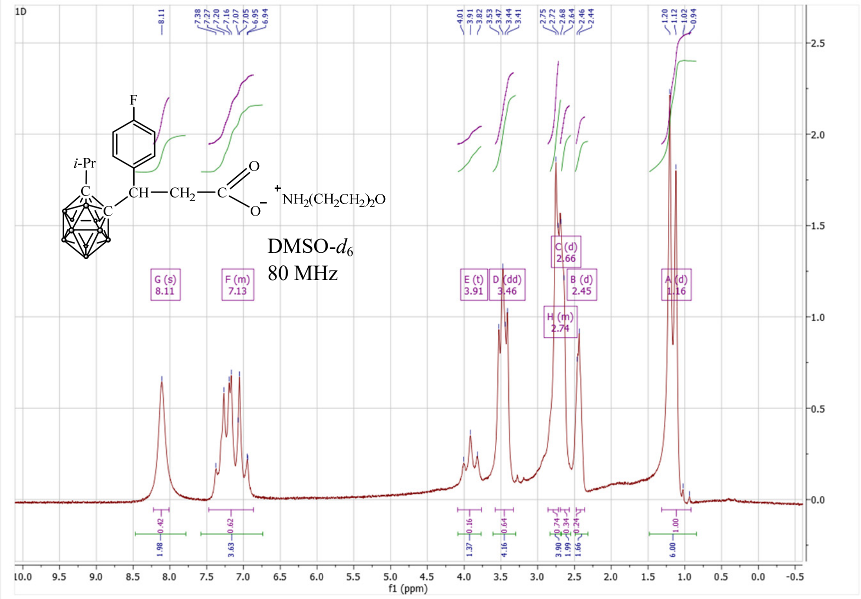

^{1}H NMR spectra of Morpholine 3-(2-isopropyl-1,2-dicarba-closo-dodecaboran-1-yl)-3-(4-fluorophenyl)propanoate salt (31)

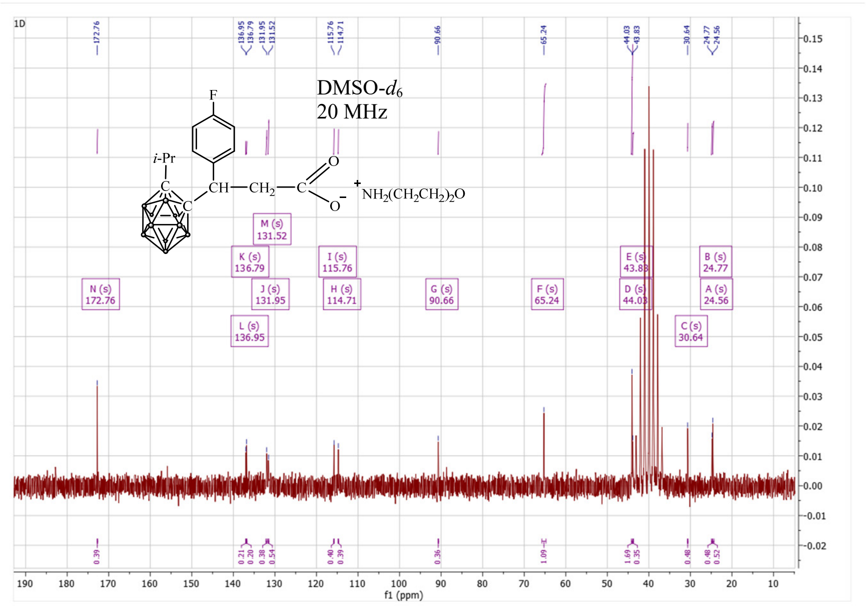

^{13}C NMR spectra of Morpholine 3-(2-isopropyl-1,2-dicarba-closo-dodecaboran-1-yl)-3-(4-fluorophenyl)propanoate salt (31)

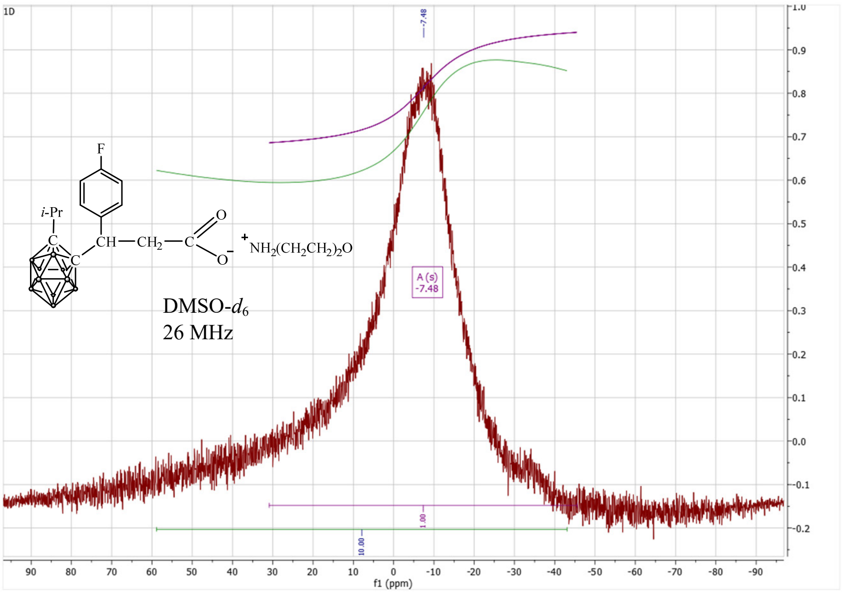

^{11}B NMR spectra of Morpholine 3-(2-isopropyl-1,2-dicarba-closo-dodecaboran-1-yl)-3-(4-fluorophenyl)propanoate salt (31)

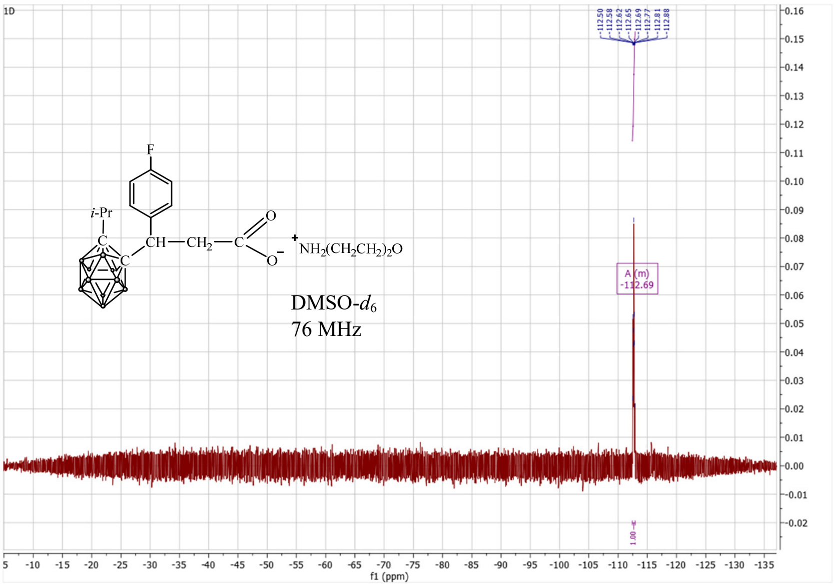

^{19}F NMR spectra of Morpholine 3-(2-isopropyl-1,2-dicarba-closo-dodecaboran-1-yl)-3-(4-fluorophenyl)propanoate salt (31)

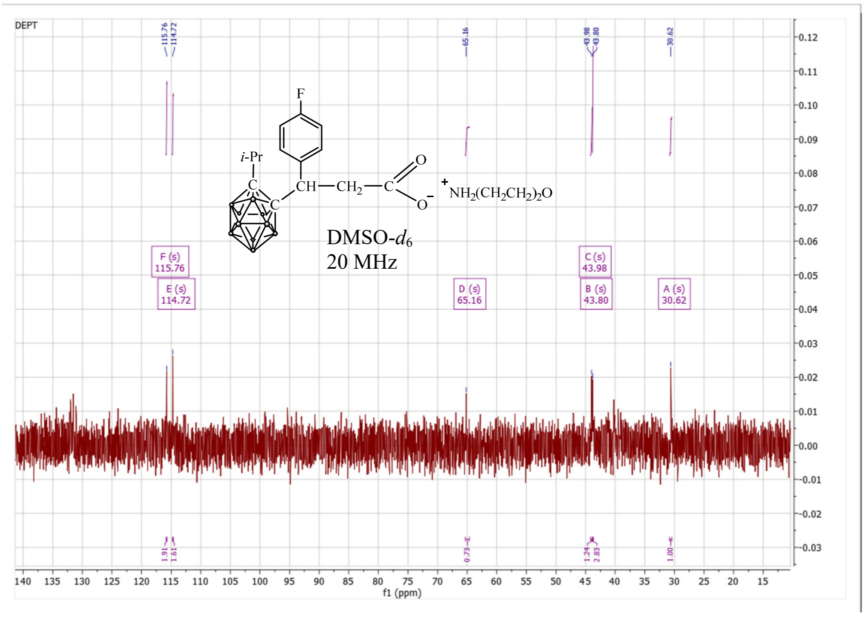

DEPT-90 NMR spectra of Morpholine 3-(2-isopropyl-1,2-dicarba-closo-dodecaboran-1-yl)-3-(4-fluorophenyl)propanoate salt (31)

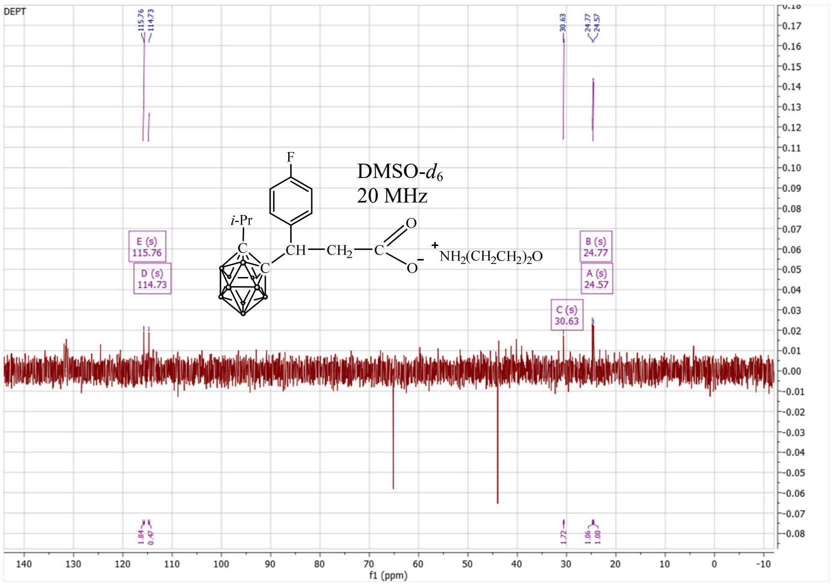

DEPT-135 NMR spectra of Morpholine 3-(2-isopropyl-1,2-dicarba-closo-dodecaboran-1-yl)-3-(4-fluorophenyl)propanoate salt (31)

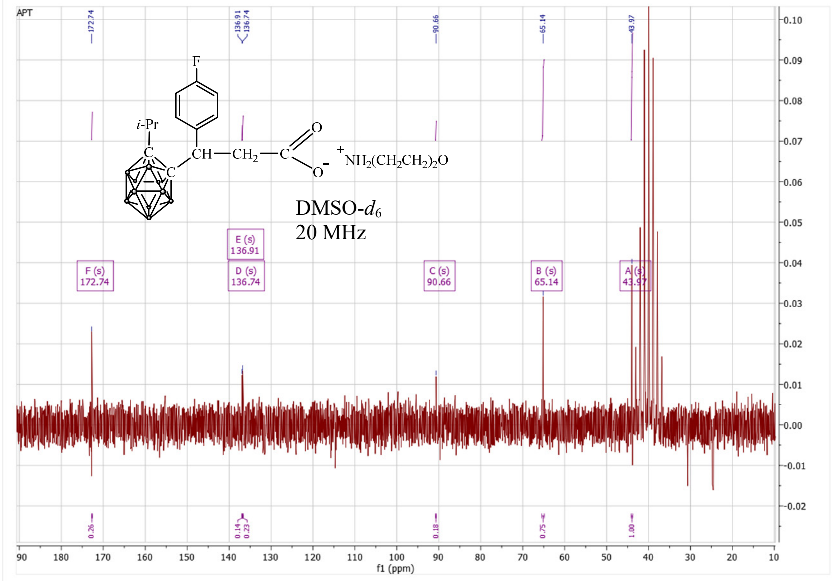

APT-WALTZ NMR spectra of Morpholine 3-(2-isopropyl-1,2-dicarba-closo-dodecaboran-1-yl)-3-(4-fluorophenyl)propanoate salt (31)

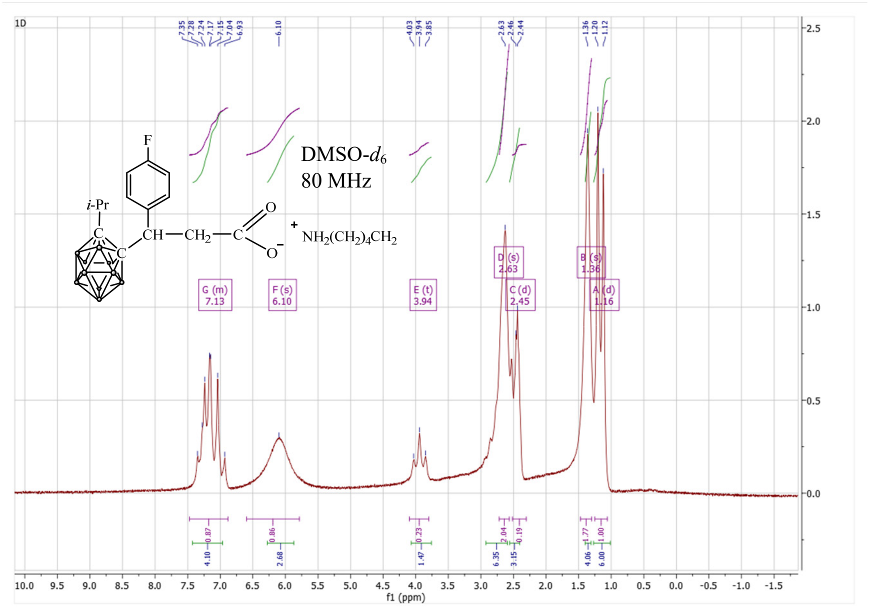

^{1}H NMR spectra of Piperidine 3-(2-isopropyl-1,2-dicarba-closo-dodecaboran-1-yl)-3-(4-fluorophenyl)propanoate salt (32)

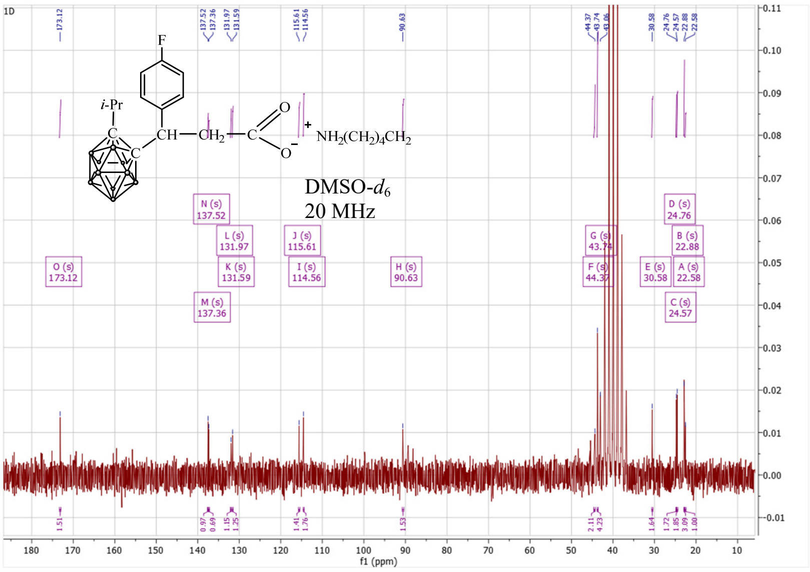

^{13}C NMR spectra of Piperidine 3-(2-isopropyl-1,2-dicarba-closo-dodecaboran-1-yl)-3-(4-fluorophenyl)propanoate salt (32)

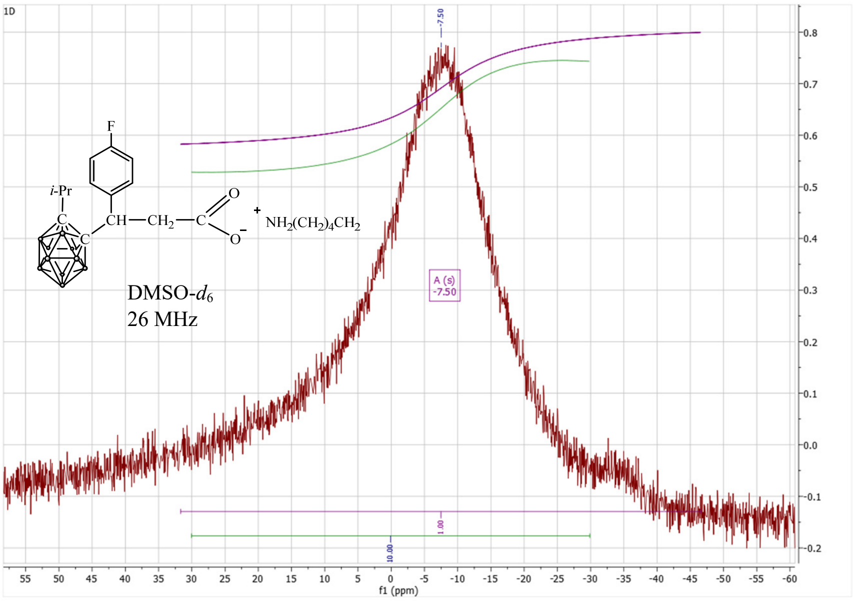

^{11}B NMR spectra of Piperidine 3-(2-isopropyl-1,2-dicarba-closo-dodecaboran-1-yl)-3-(4-fluorophenyl)propanoate salt (32)

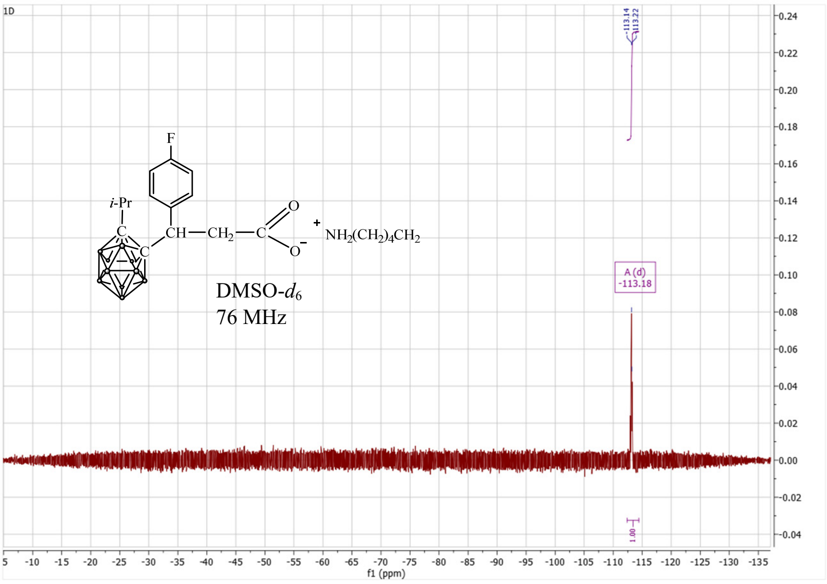

^{19}F NMR spectra of Piperidine 3-(2-isopropyl-1,2-dicarba-closo-dodecaboran-1-yl)-3-(4-fluorophenyl)propanoate salt (32)

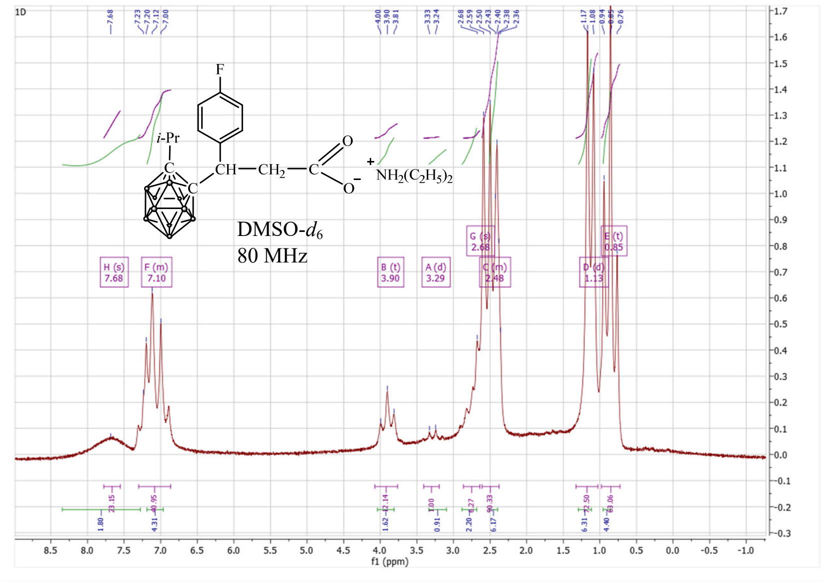

^{1}H NMR spectra of Diethylammonium 3-(2-isopropyl-1,2-dicarba-closo-dodecaboran-1-yl)-3-(4-fluorophenyl)propanoate salt (33)

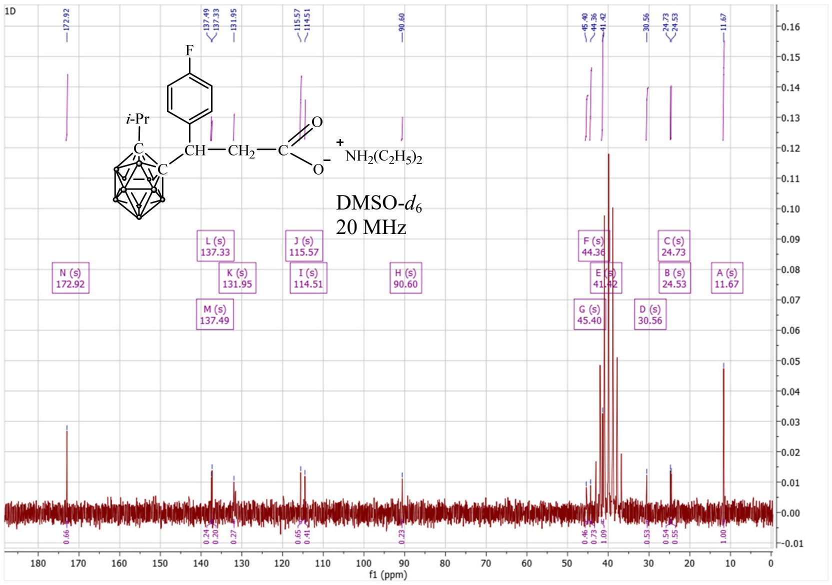

^{13}C NMR spectra of Diethylammonium 3-(2-isopropyl-1,2-dicarba-closo-dodecaboran-1-yl)-3-(4-fluorophenyl)propanoate salt (33)

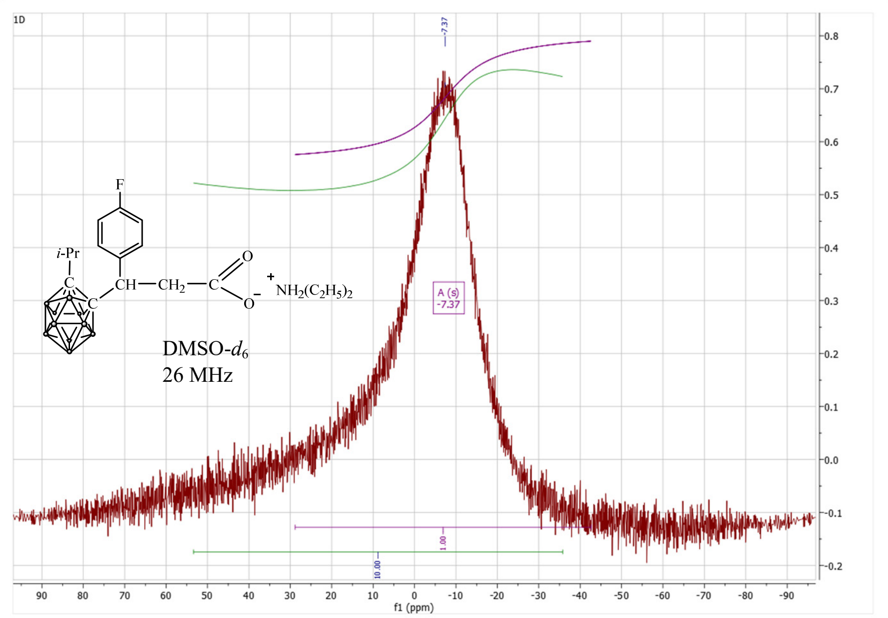

^{11}B NMR spectra of Diethylammonium 3-(2-isopropyl-1,2-dicarba-closo-dodecaboran-1-yl)-3-(4-fluorophenyl)propanoate salt (33)

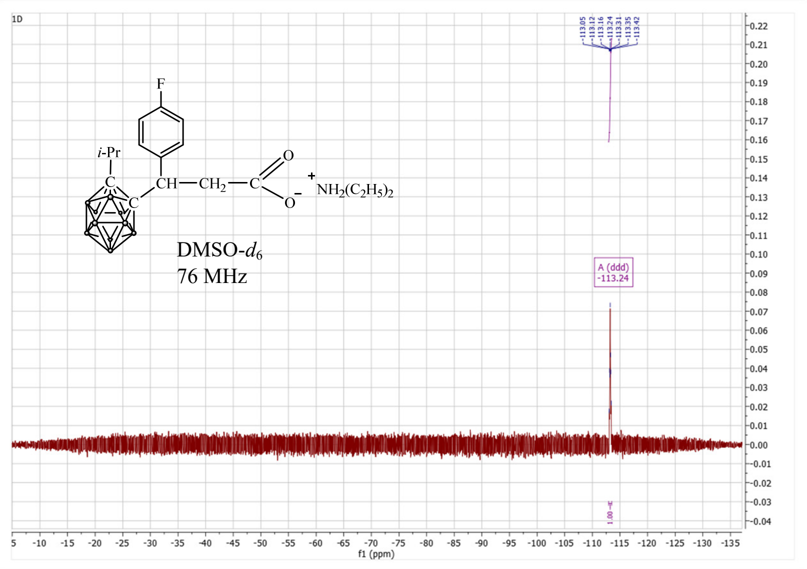

^{19}F NMR spectra of Diethylammonium 3-(2-isopropyl-1,2-dicarba-closo-dodecaboran-1-yl)-3-(4-fluorophenyl)propanoate salt (33)

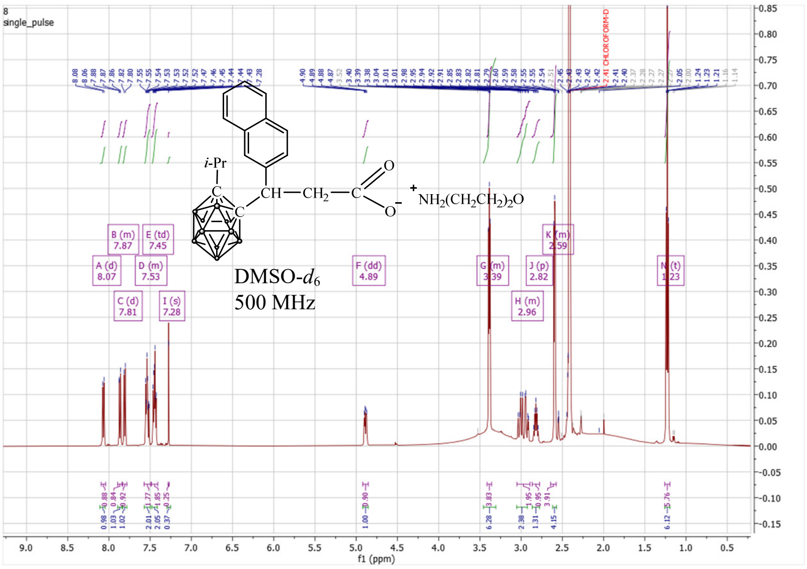

^{1}H NMR spectra of Morpholine 3-(2-isopropyl-1,2-dicarba-closo-dodecaboran-1-yl)-3-(naphthalene-1-yl)propanoate salt (34)

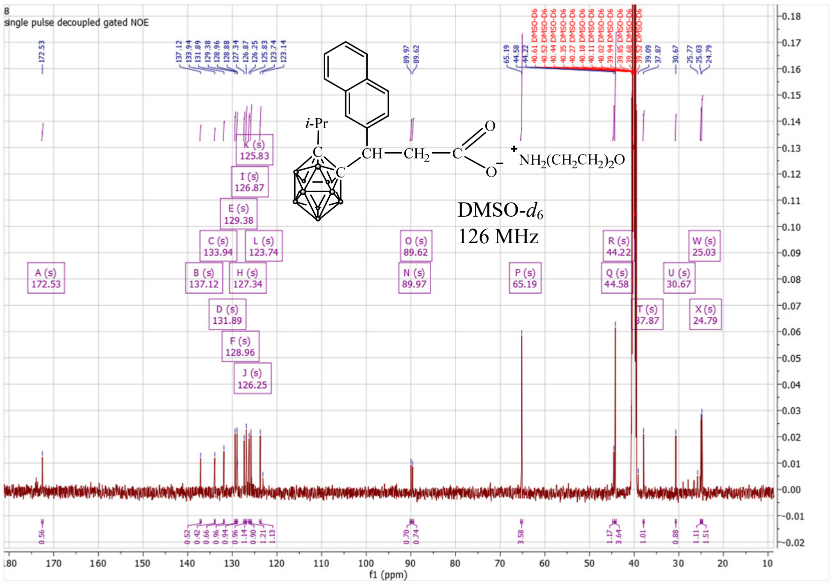

^{13}C NMR spectra of Morpholine 3-(2-isopropyl-1,2-dicarba-closo-dodecaboran-1-yl)-3-(naphthalene-1-yl)propanoate salt (34)

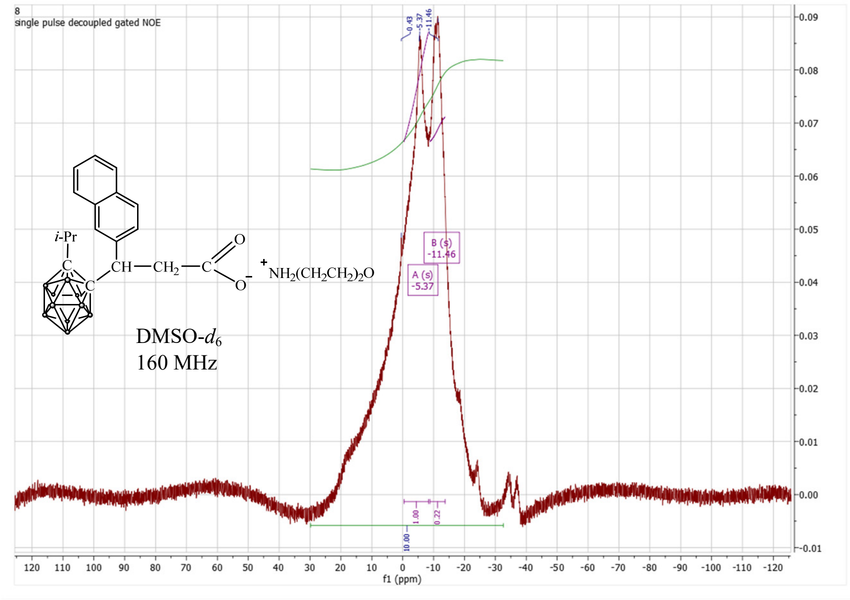

^{11}B NMR spectra of Morpholine 3-(2-isopropyl-1,2-dicarba-closo-dodecaboran-1-yl)-3-(naphthalene-1-yl)propanoate salt (34)

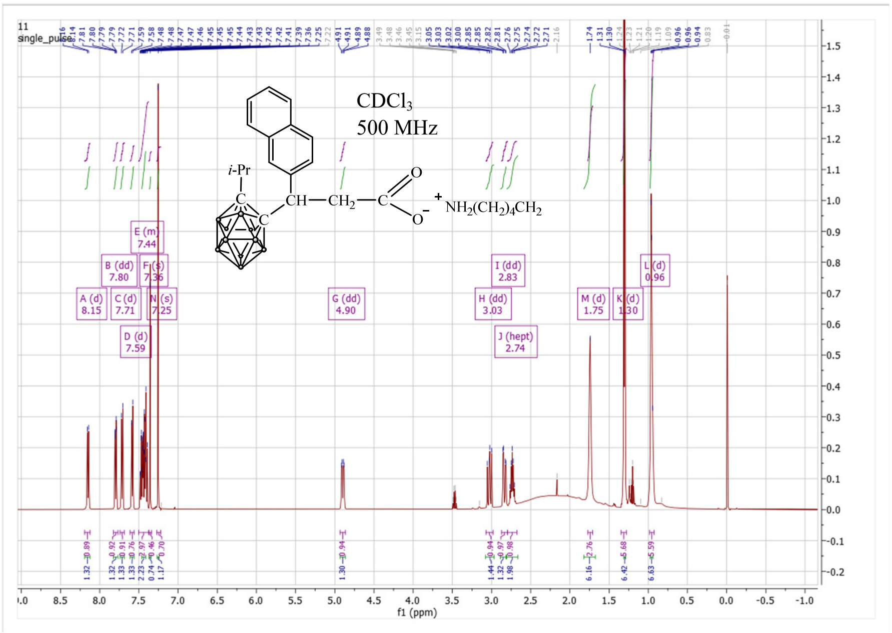

^{1}H NMR spectra of Piperidine 3-(2-isopropyl-1,2-dicarba-closo-dodecaboran-1-yl)-3-(naphthalene-1-yl)propanoate salt (35)

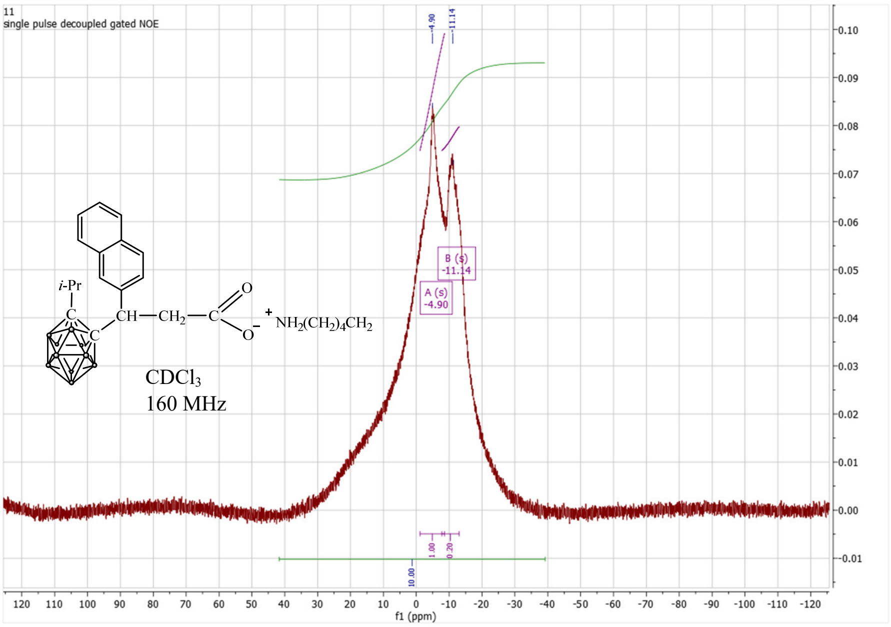

^{11}B NMR spectra of Piperidine 3-(2-isopropyl-1,2-dicarba-closo-dodecaboran-1-yl)-3-(naphthalene-1-yl)propanoate salt (35)

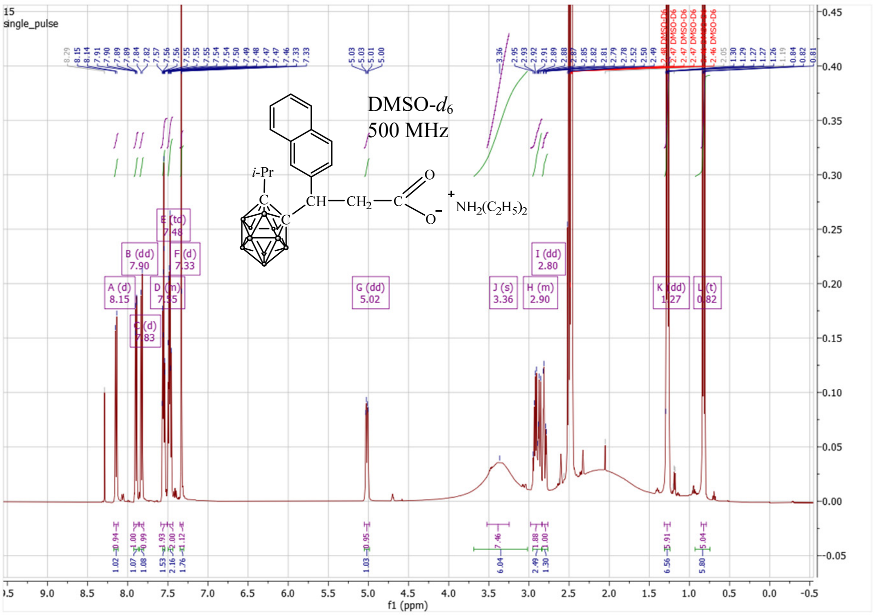

^{1}H NMR spectra of Diethylammonium 3-(2-isopropyl-1,2-dicarba-closo-dodecaboran-1-yl)-3-(naphthalene-1-yl)propanoate salt (36)

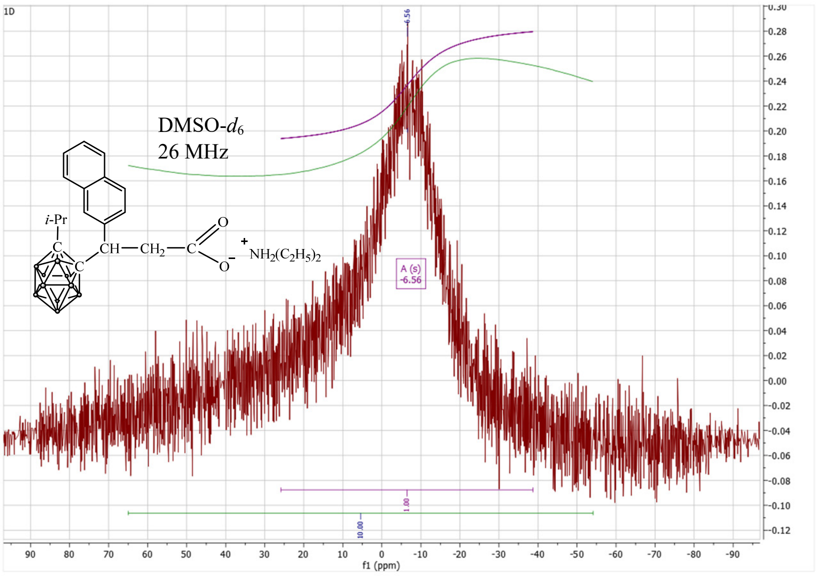

^{11}B NMR spectra of Diethylammonium 3-(2-isopropyl-1,2-dicarba-closo-dodecaboran-1-yl)-3-(naphthalene-1-yl)propanoate salt (36)

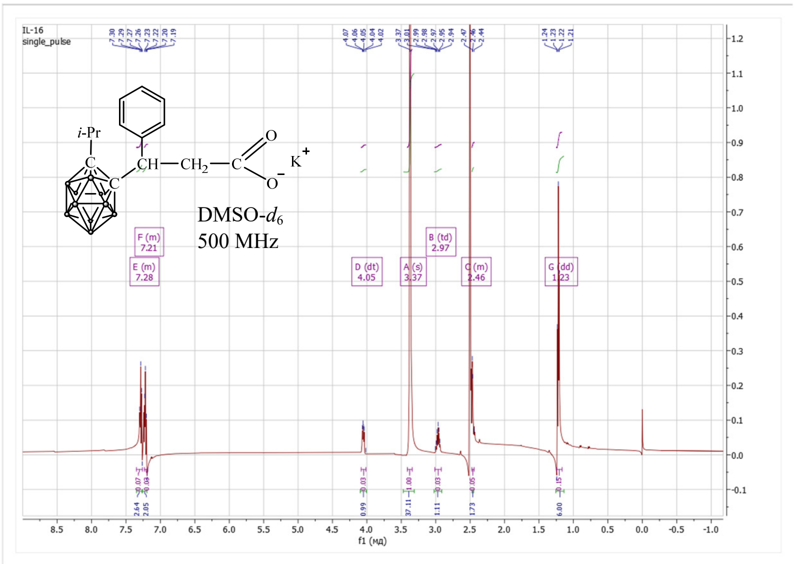

^{1}H NMR spectra of Potassium 3-(2-isopropyl-1,2-dicarba-closo-dodecaboran-1-yl)-3-phenylpropanoate (37)

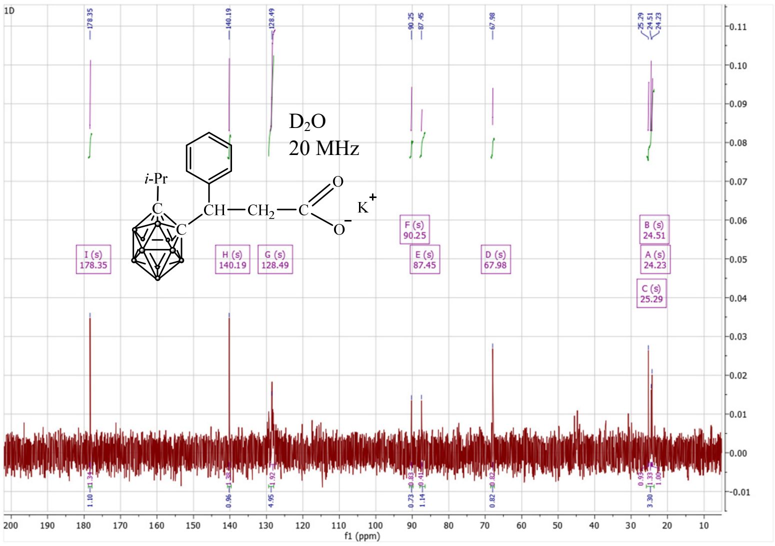

^{13}C NMR spectra of Potassium 3-(2-isopropyl-1,2-dicarba-closo-dodecaboran-1-yl)-3-phenylpropanoate (37)

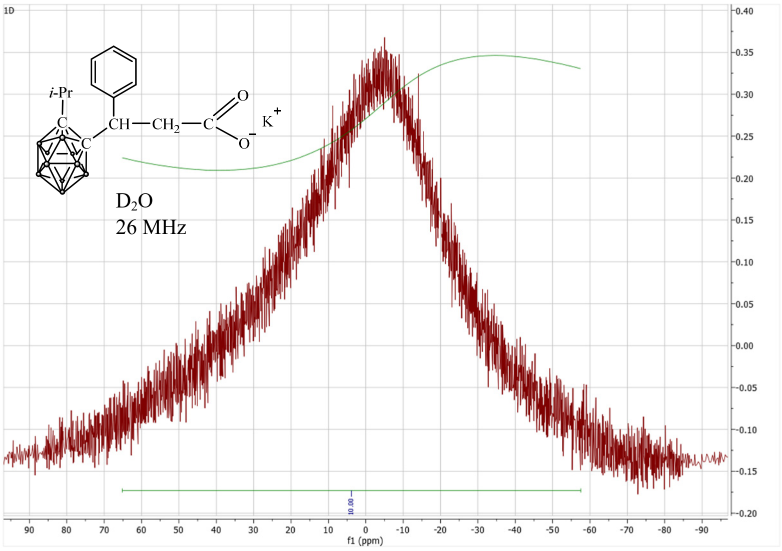

^{11}B NMR spectra of Potassium 3-(2-isopropyl-1,2-dicarba-closo-dodecaboran-1-yl)-3-phenylpropanoate (37)

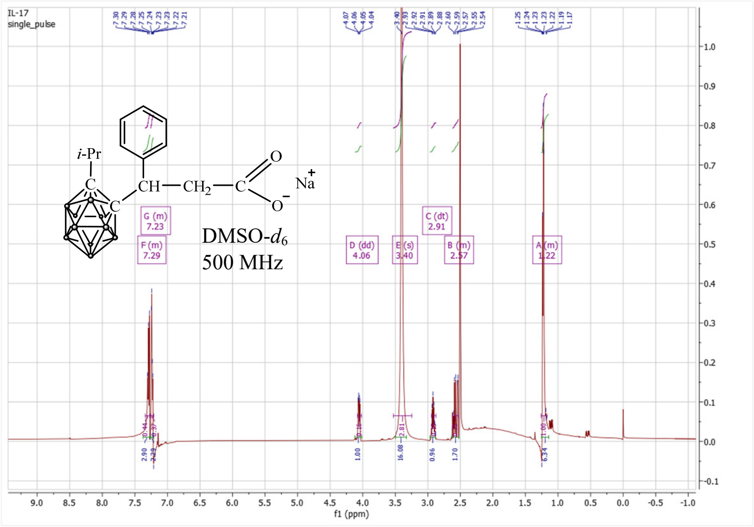

^{1}H NMR spectra of Sodium 3-(2-isopropyl-1,2-dicarba-closo-dodecaboran-1-yl)-3-phenylpropanoate (38)

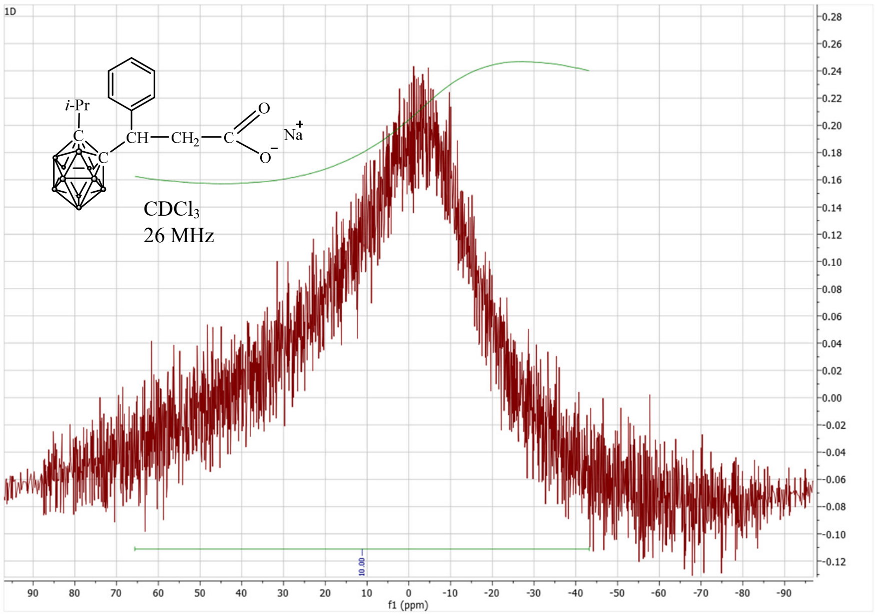

^{11}B NMR spectra of Sodium 3-(2-isopropyl-1,2-dicarba-closo-dodecaboran-1-yl)-3-phenylpropanoate (38)

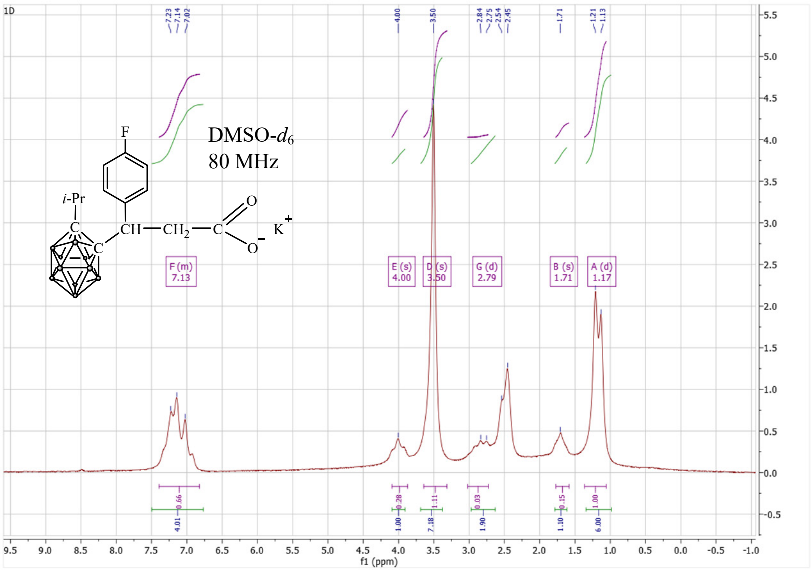

^{1}H NMR spectra of Potassium 3-(2-isopropyl-1,2-dicarba-closo-dodecaboran-1-yl)-3-(4-fluorophenyl)propanoate (39)

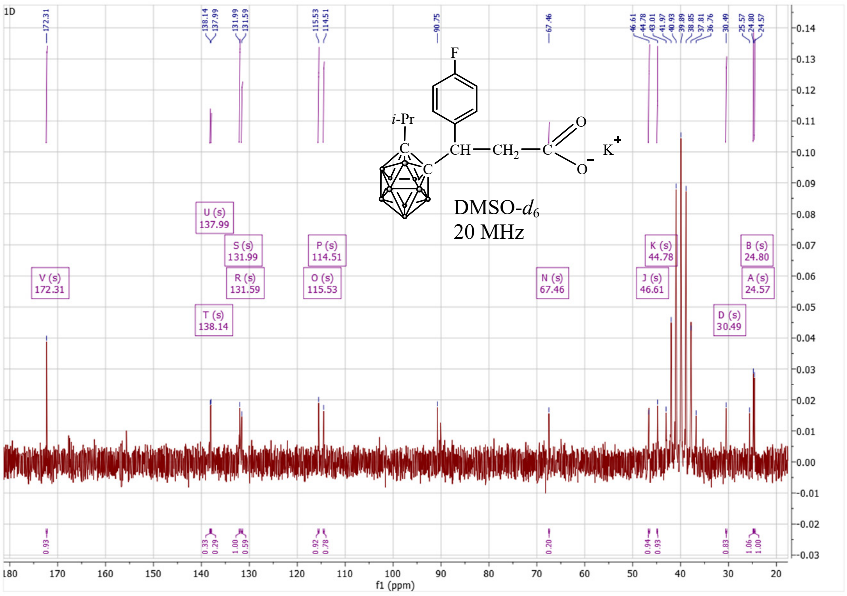

^{13}C NMR spectra of Potassium 3-(2-isopropyl-1,2-dicarba-closo-dodecaboran-1-yl)-3-(4-fluorophenyl)propanoate (39)

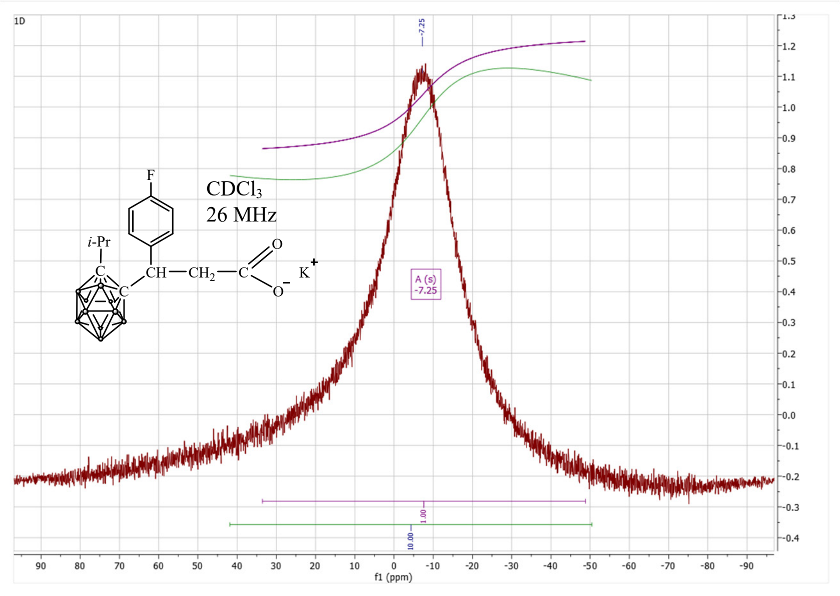

^{11}B NMR spectra of Potassium 3-(2-isopropyl-1,2-dicarba-closo-dodecaboran-1-yl)-3-(4-fluorophenyl)propanoate (39)

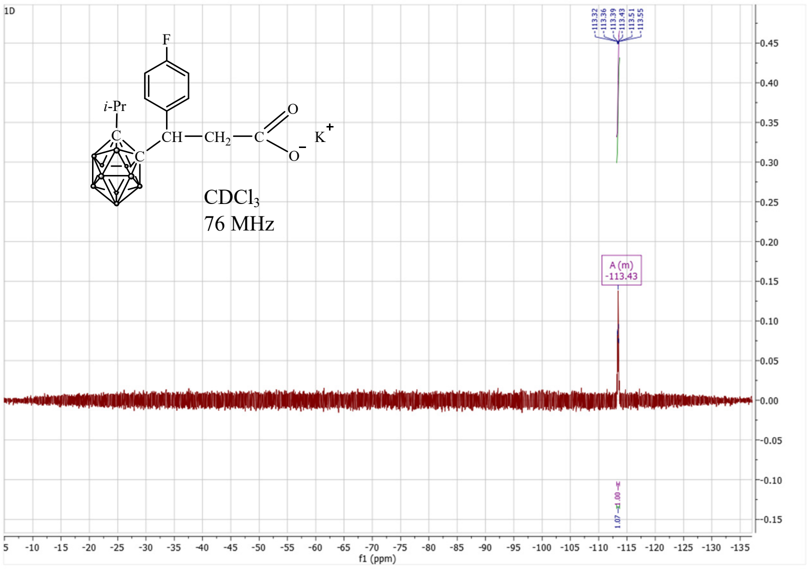

^{19}F NMR spectra of Potassium 3-(2-isopropyl-1,2-dicarba-closo-dodecaboran-1-yl)-3-(4-fluorophenyl)propanoate (39)

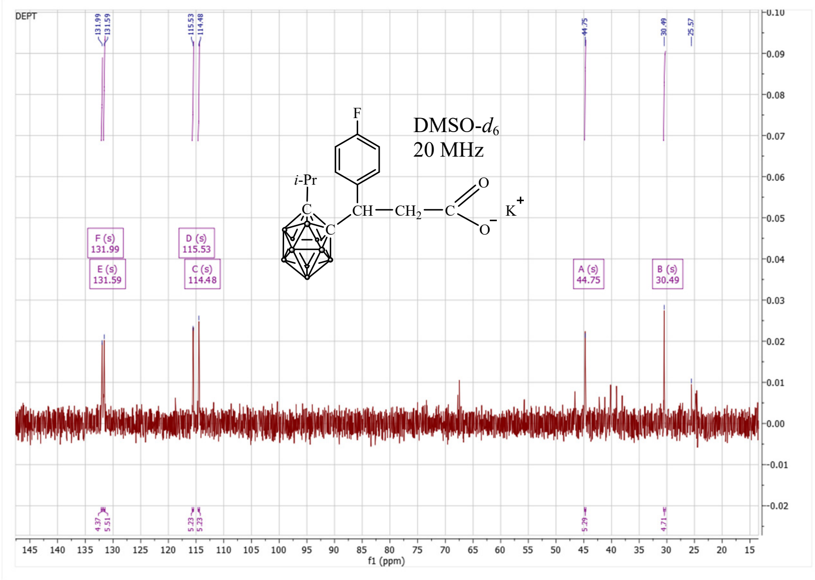

DEPT-90 NMR spectra of Potassium 3-(2-isopropyl-1,2-dicarba-closo-dodecaboran-1-yl)-3-(4-fluorophenyl)propanoate (39)

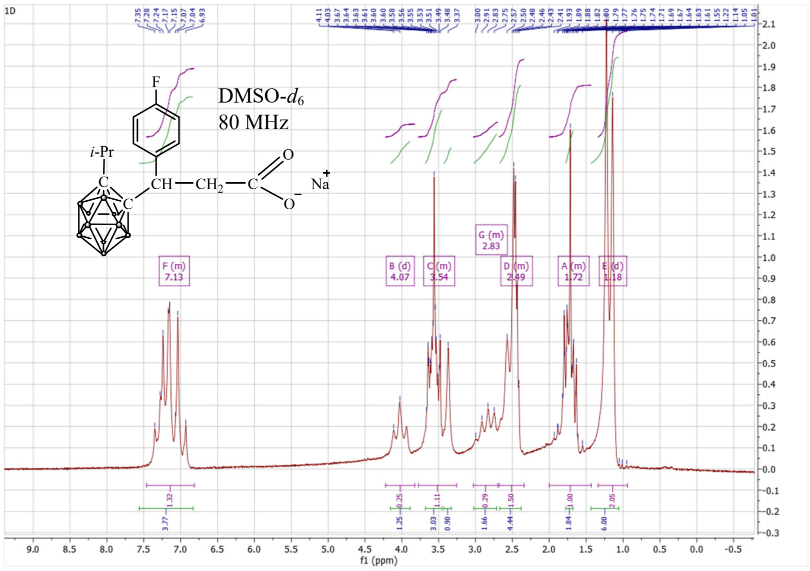

^{1}H NMR spectra of Sodium 3-(2-isopropyl-1,2-dicarba-closo-dodecaboran-1-yl)-3-(4-fluorophenyl)propanoate (40)

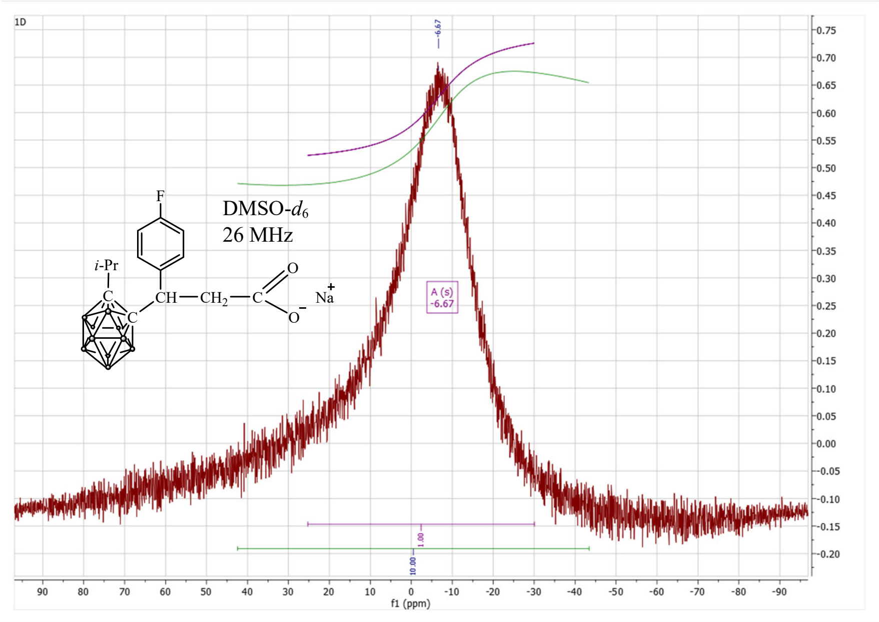

^{11}B NMR spectra of Sodium 3-(2-isopropyl-1,2-dicarba-closo-dodecaboran-1-yl)-3-(4-fluorophenyl)propanoate (40)

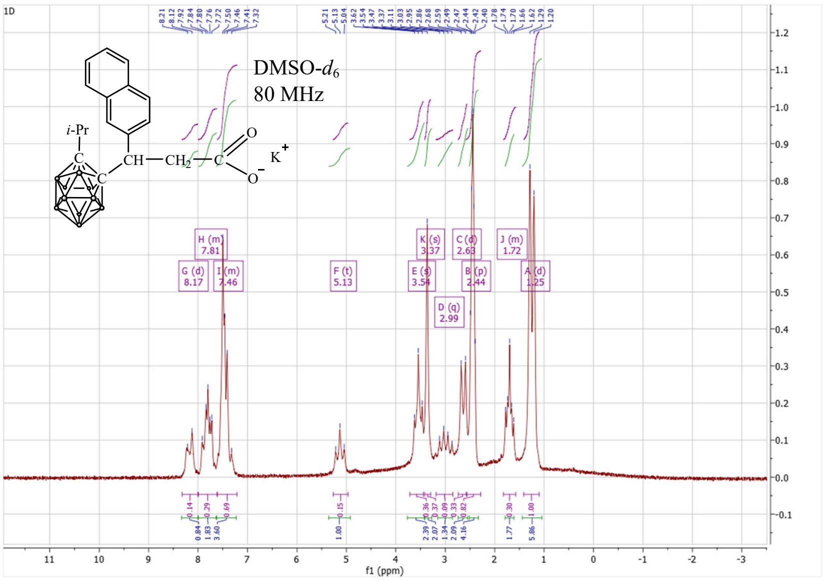

^{1}H NMR spectra of Potassium 3-(2-isopropyl-1,2-dicarba-closo-dodecaboran-1-yl)-3-(naphthalene-1-yl)propanoate (41)

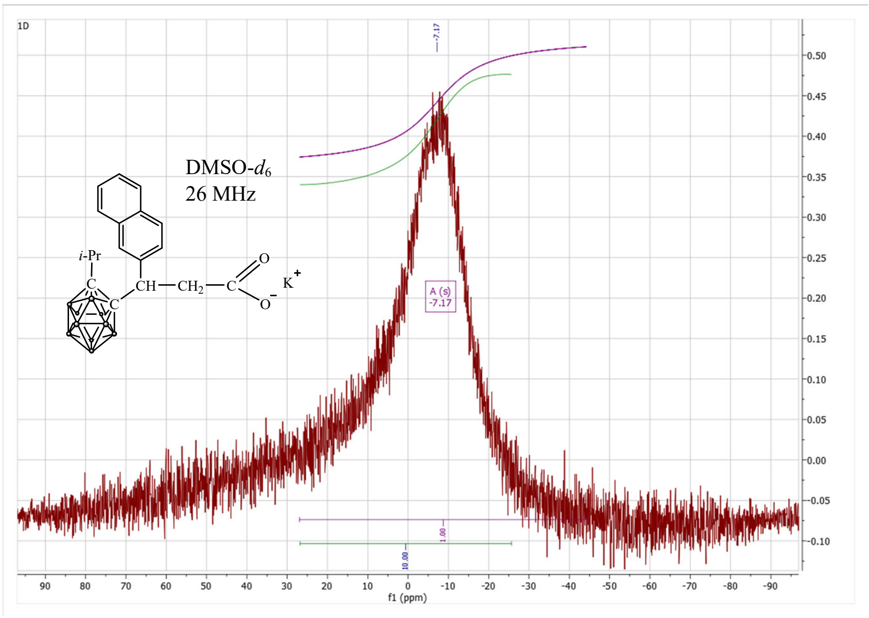

^{11}B NMR spectra of Potassium 3-(2-isopropyl-1,2-dicarba-closo-dodecaboran-1-yl)-3-(naphthalene-1-yl)propanoate (41)

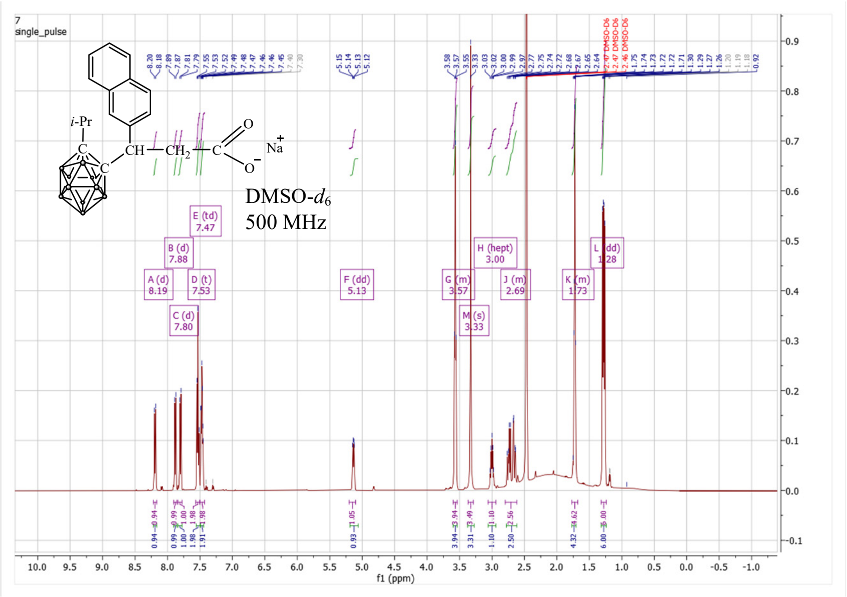

^{1}H NMR spectra of Sodium 3-(2-isopropyl-1,2-dicarba-closo-dodecaboran-1-yl)-3-(naphthalene-1-yl)propanoate (42)

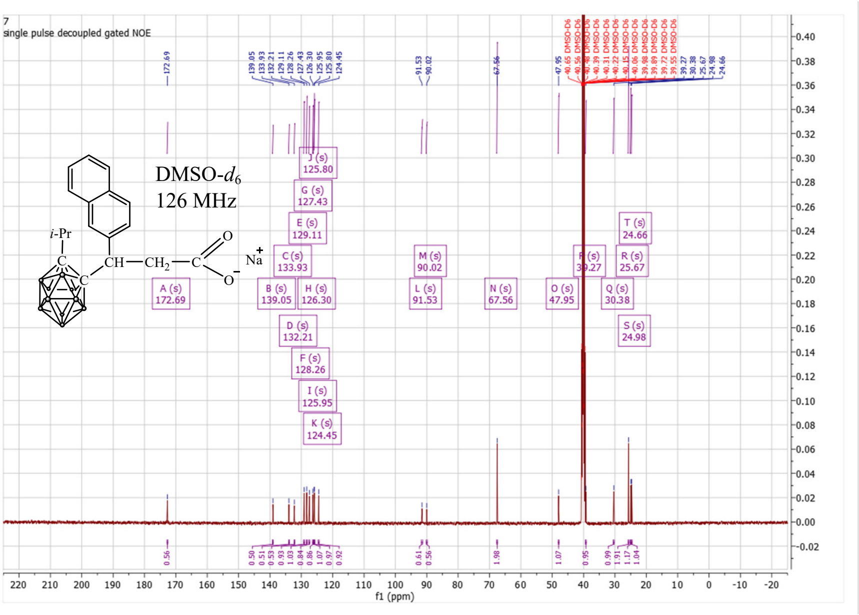

^{13}C NMR spectra of Sodium 3-(2-isopropyl-1,2-dicarba-closo-dodecaboran-1-yl)-3-(naphthalene-1-yl)propanoate (42)

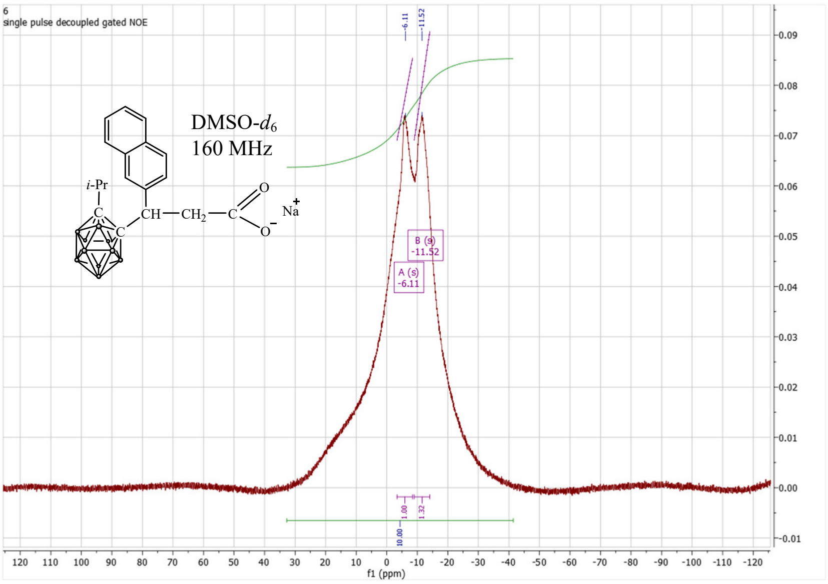

^{11}B NMR spectra of Sodium 3-(2-isopropyl-1,2-dicarba-closo-dodecaboran-1-yl)-3-(naphthalene-1-yl)propanoate (42)

FTIR spectra

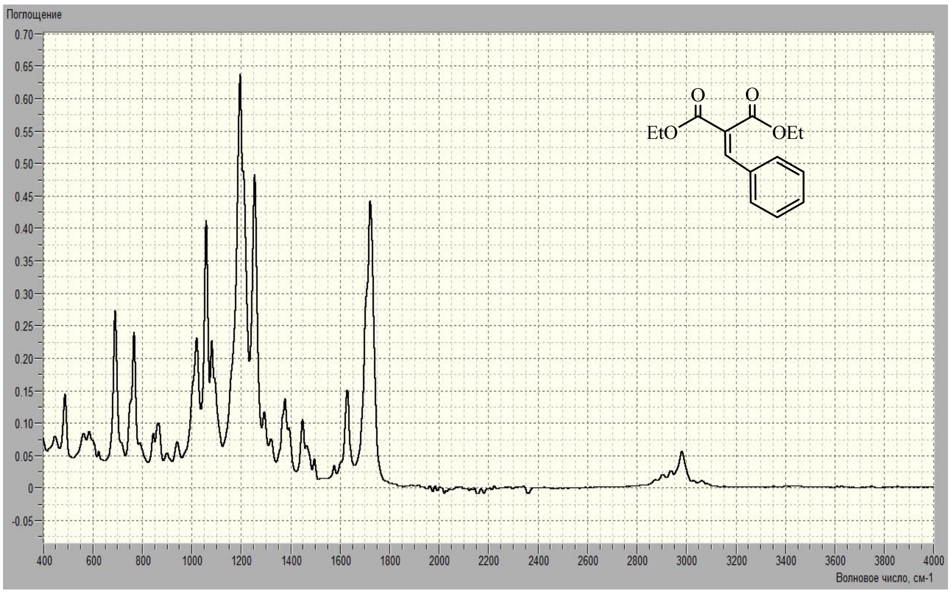

FTIR spectra of Diethyl 2-benzylidenemalonate (1)

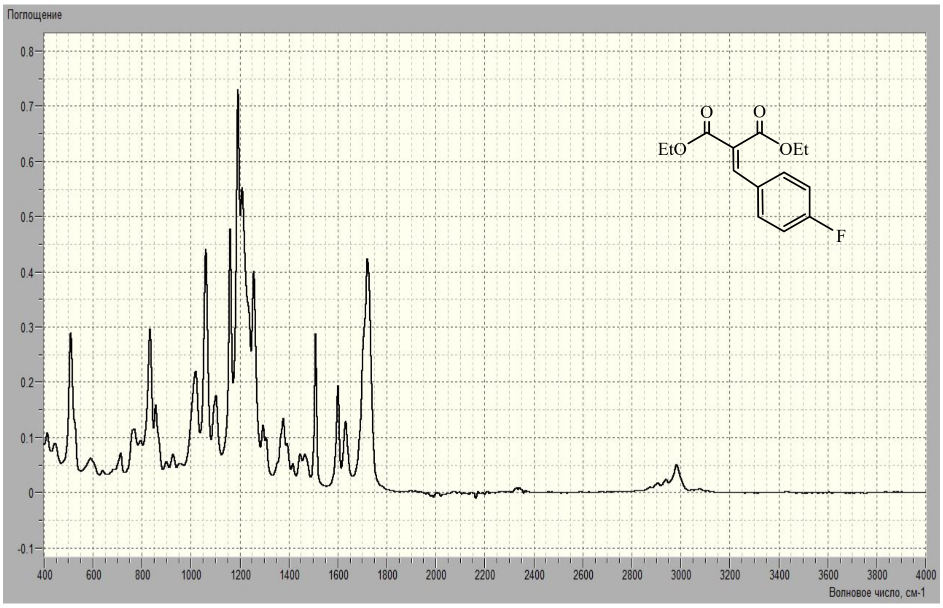

FTIR spectra of Diethyl 2-(4-fluorobenzylidene)malonate (2)

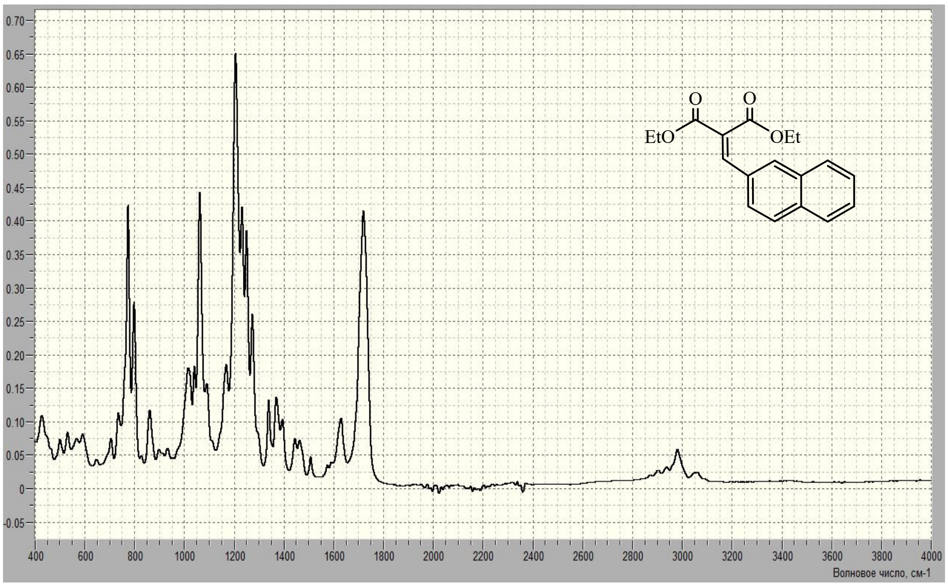

FTIR spectra of Diethyl 2-(naphthalen-1-yl)malonate (3)

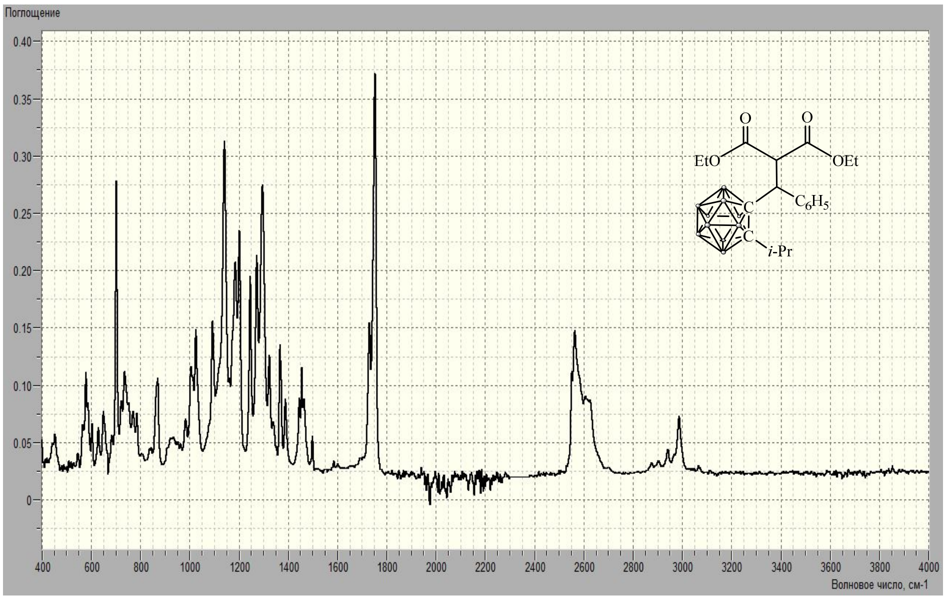

FTIR spectra of Diethyl 2-[4-(1-isopropyl-closo-1,2-dicarba-closo-dodecaboran-1-yl)benzyl]propanedioate (4)

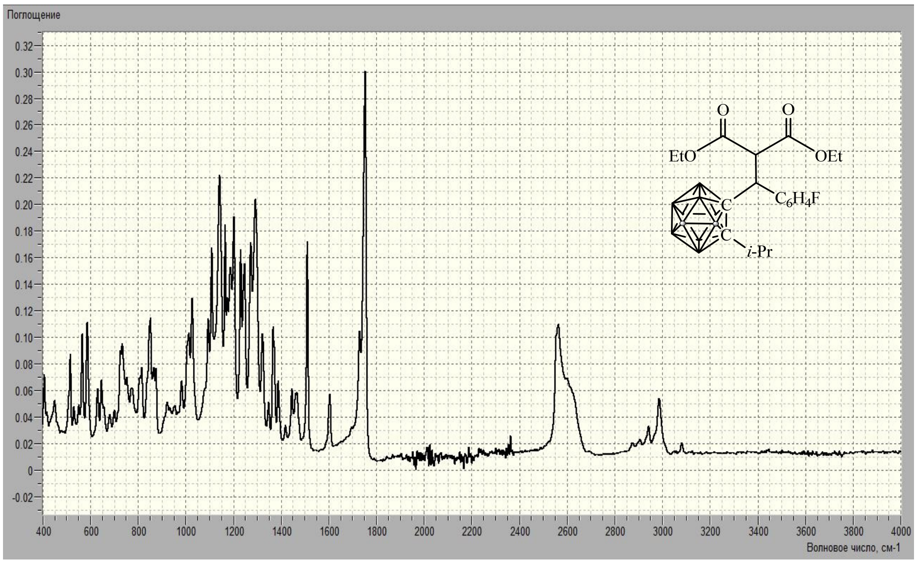

FTIR spectra of Diethyl 2-[4-fluoro-4-(1-isopropyl-closo-1,2-dicarba-closo-dodecaboran-1-yl)benzyl]propanedioate (5)

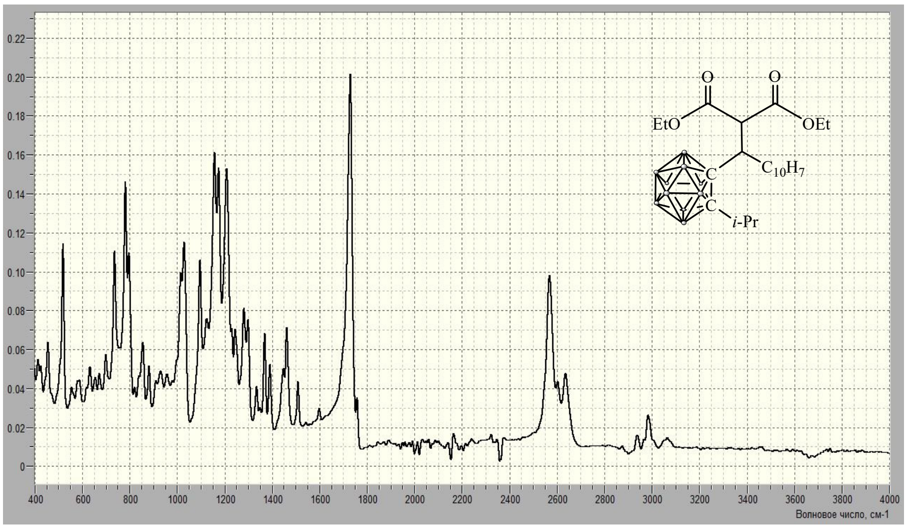

FTIR spectra of Diethyl 2-[(1-isopropyl-closo-1,2-dicarba-closo-dodecaboran-1-yl)naphthalen-1-yl]propanedioate (6)

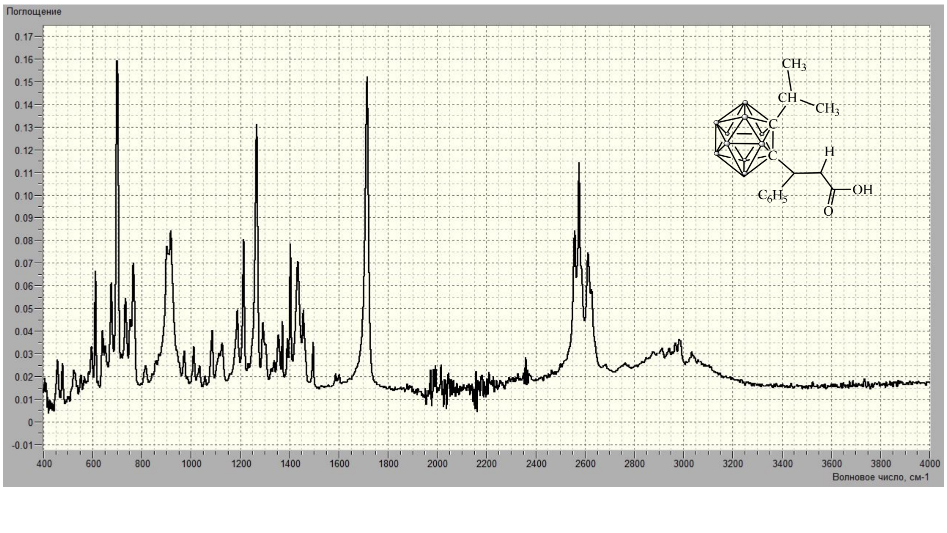

FTIR spectra of 3-(2-isopropyl-1,2-dicarba-closo-dodecaboran-1-yl)-3-phenylpropanoic acid (7)

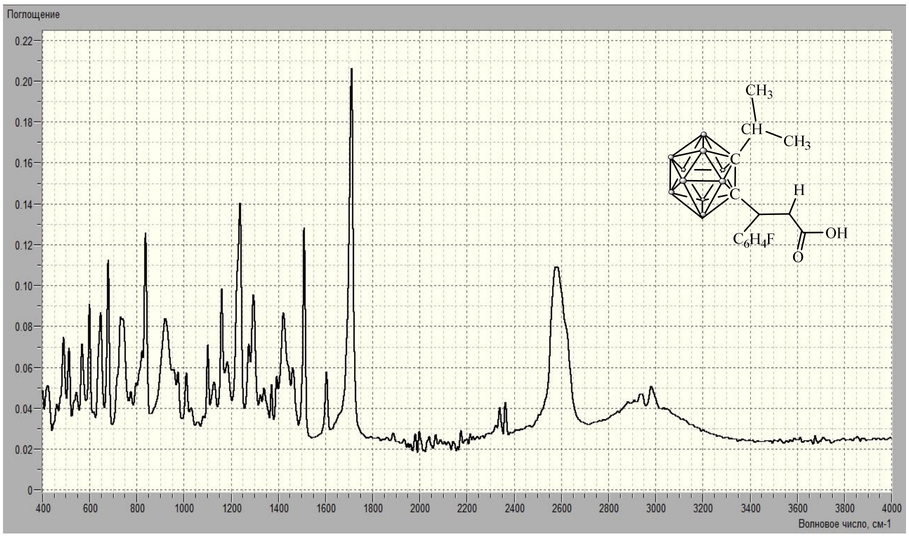

FTIR spectra of 3-(2-isopropyl-1,2-dicarba-closo-dodecaboran-1-yl)-3-(4-fluorophenyl)propanoic acid (8)

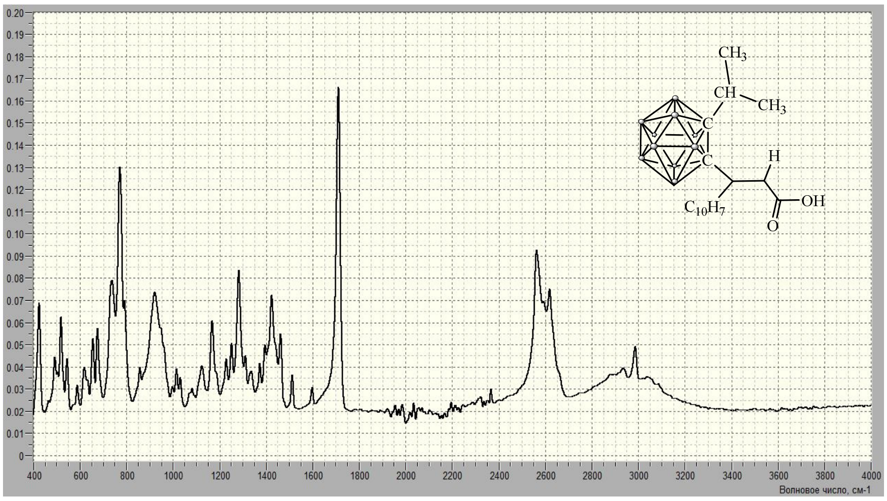

FTIR spectra of 3-(2-isopropyl-1,2-dicarba-closo-dodecaboran-1-yl)-3-(naphthalene-1-yl)propanoic acid (9)

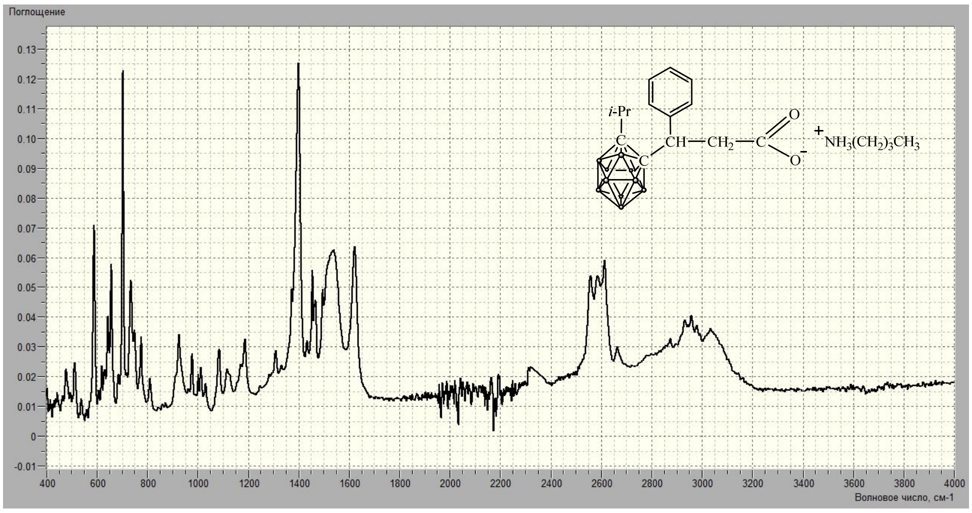

FTIR spectra of Butylammonium 3-(2-isopropyl-1,2-dicarba-closo-dodecaboran-1-yl)-3-phenylpropanoate (10)

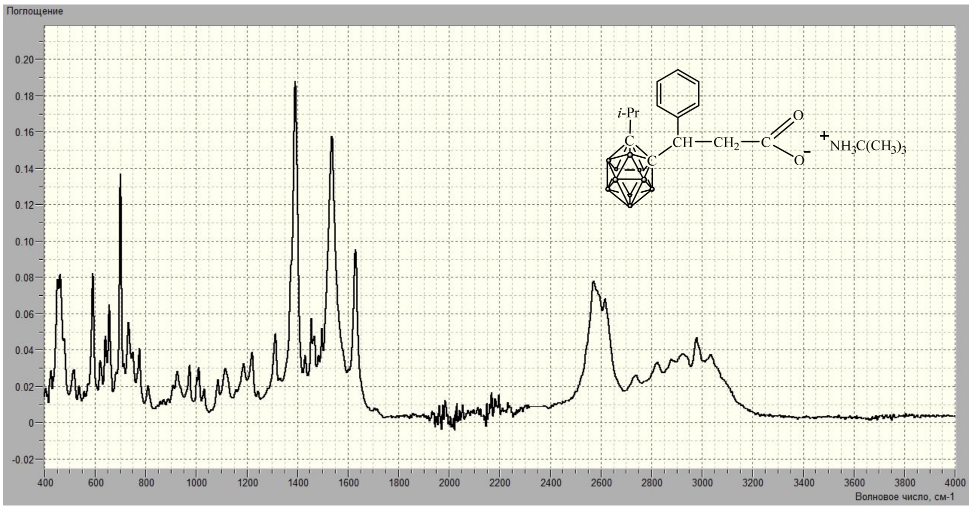

FTIR spectra of Tert-butylammonium 3-(2-isopropyl-1,2-dicarba-closo-dodecaboran-1-yl)-3-phenylpropanoate (11)

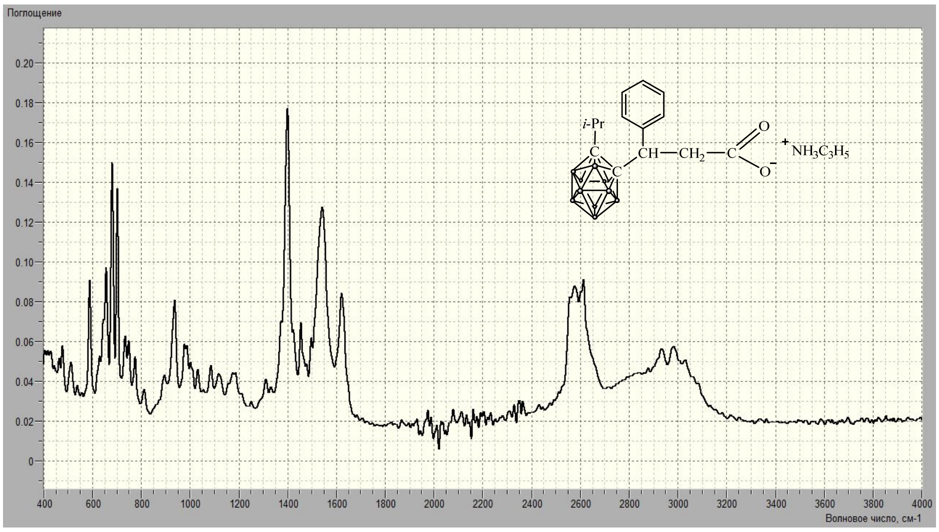

FTIR spectra of Allylammonium 3-(2-isopropyl-1,2-dicarba-closo-dodecaboran-1-yl)-3-phenylpropanoate (12)

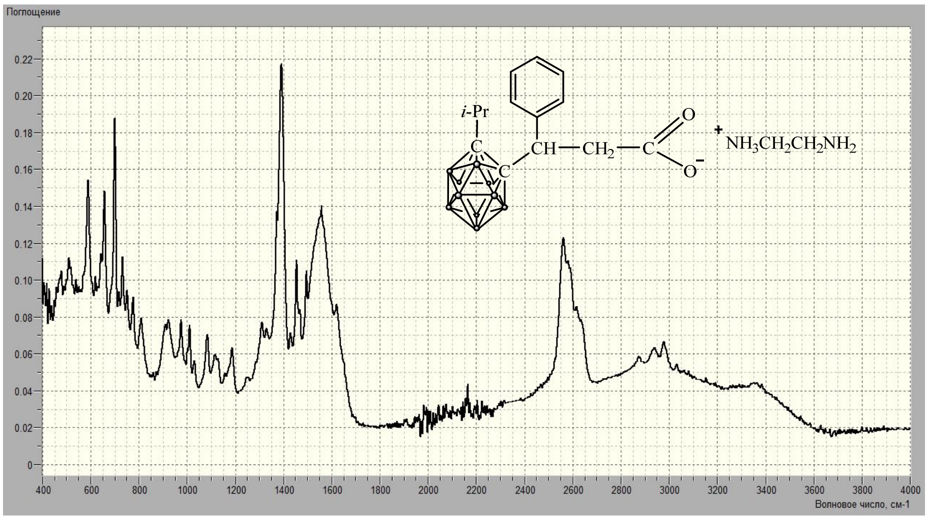

FTIR spectra of Ethan-1-amino-2-ammonium 3-(2-isopropyl-1,2-dicarba-closo-dodecaboran-1-yl)-3-phenylpropanoate (13)

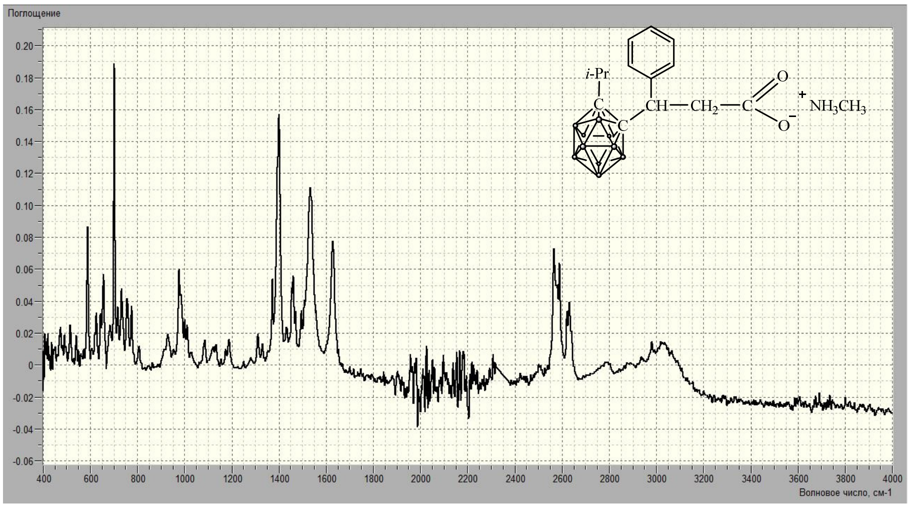

FTIR spectra of Methylammonium 3-(2-isopropyl-1,2-dicarba-closo-dodecaboran-1-yl)-3-phenylpropanoate (14)

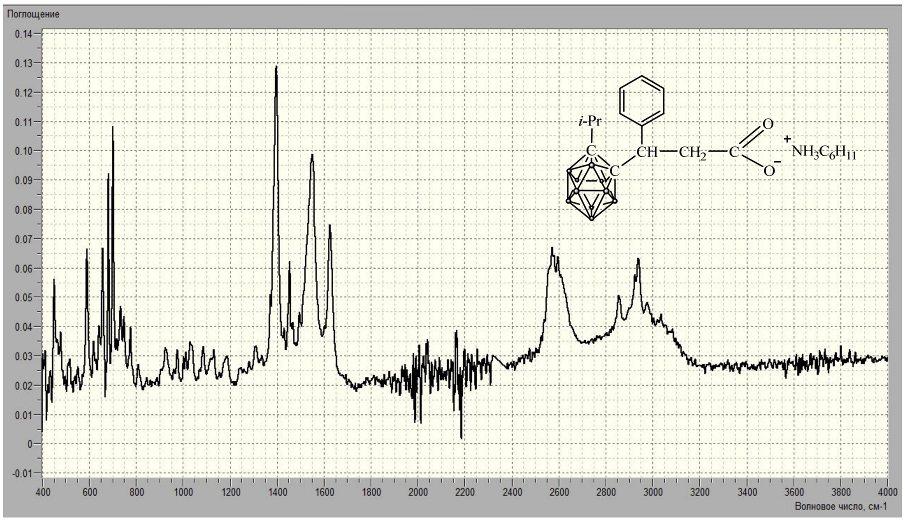

FTIR spectra of Cyclohexylammonium 3-(2-isopropyl-1,2-dicarba-closo-dodecaboran-1-yl)-3-phenylpropanoate (15)

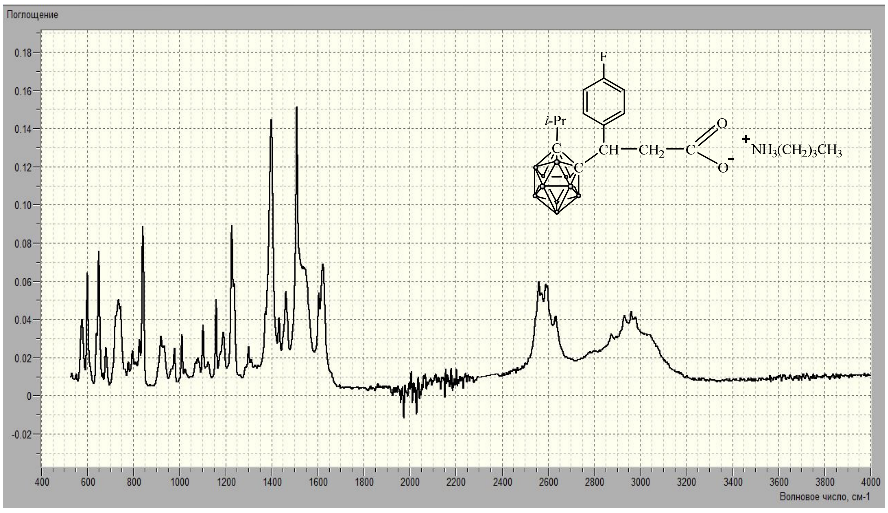

FTIR spectra of Butylammonium 3-(2-isopropyl-1,2-dicarba-closo-dodecaboran-1-yl)-3-(4-fluorophenyl)propanoate (16)

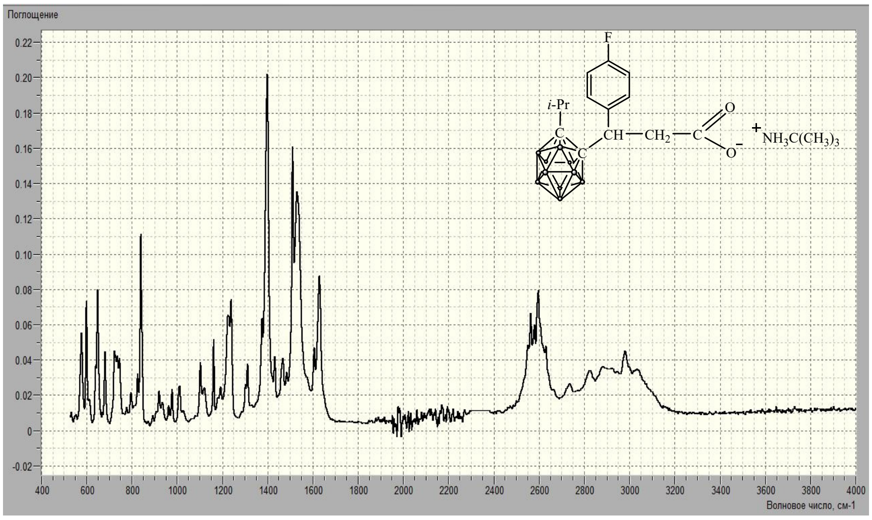

FTIR spectra of Tert-butylammonium 3-(2-isopropyl-1,2-dicarba-closo-dodecaboran-1-yl)-3-(4-fluorophenyl)propanoate (17)

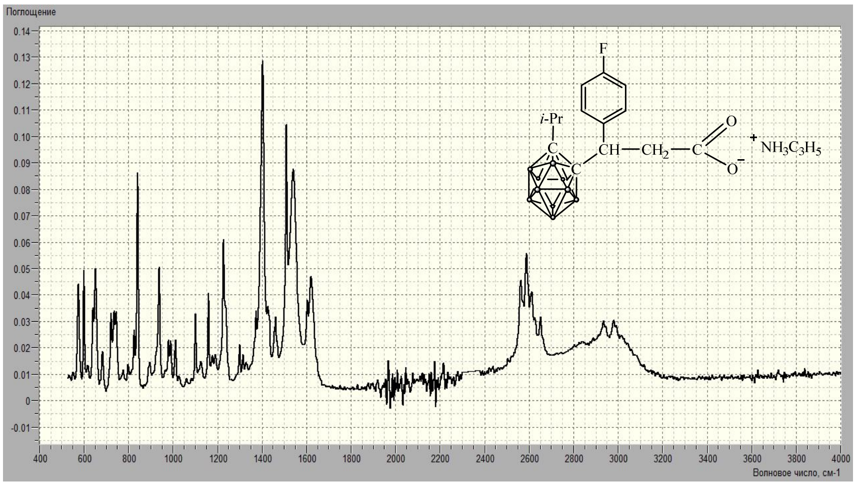

FTIR spectra of Allylammonium 3-(2-isopropyl-1,2-dicarba-closo-dodecaboran-1-yl)-3-(4-fluorophenyl)propanoate (18)

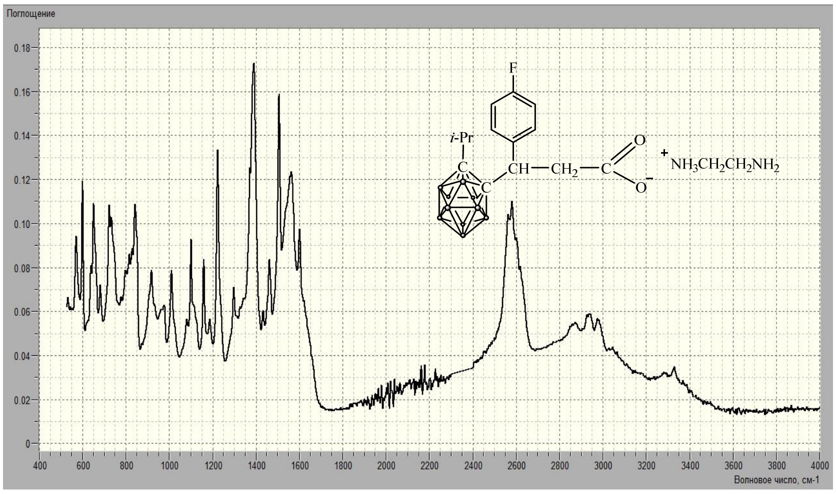

FTIR spectra of Ethan-1-amino-2-ammonium 3-(2-isopropyl-1,2-dicarba-closo-dodecaboran-1-yl)-3-(4-fluorophenyl)propanoate (19)

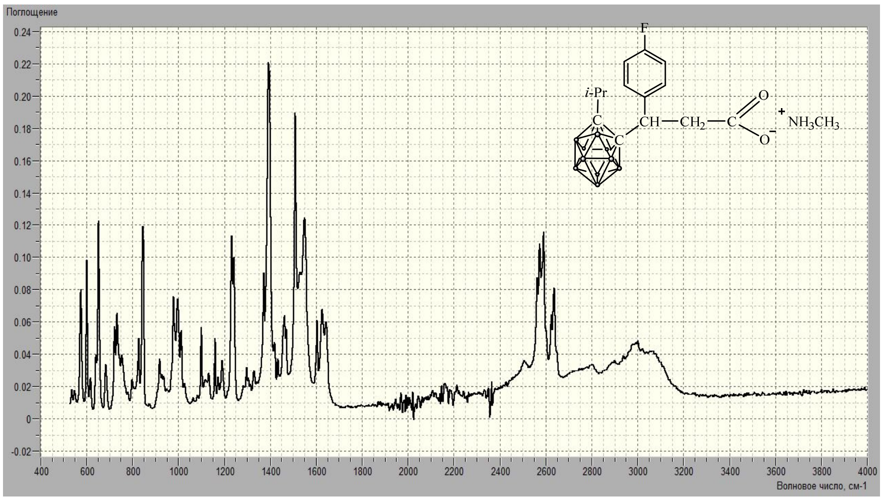

FTIR spectra of Methylammonium 3-(2-isopropyl-1,2-dicarba-closo-dodecaboran-1-yl)-3-(4-fluorophenyl)propanoate (20)

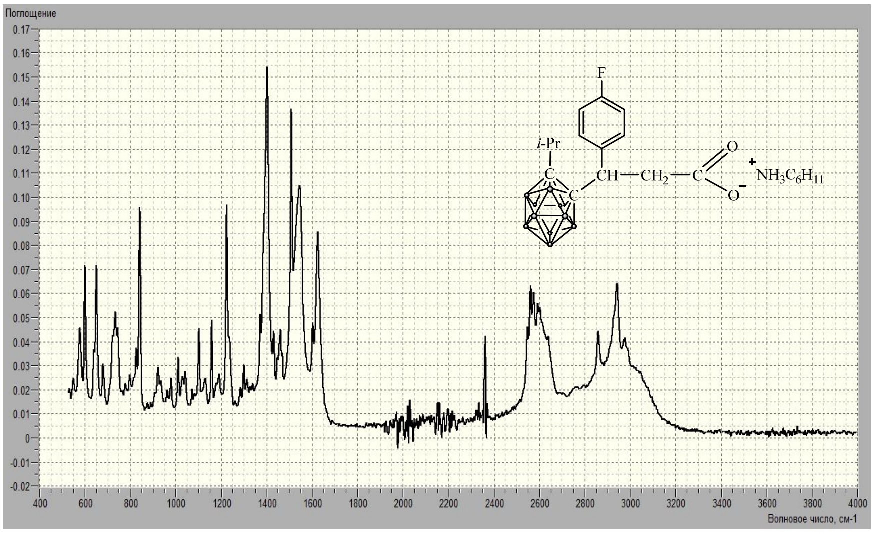

FTIR spectra of Cyclohexylammonium 3-(2-isopropyl-1,2-dicarba-closo-dodecaboran-1-yl)-3-(4-fluorophenyl)propanoate (21)

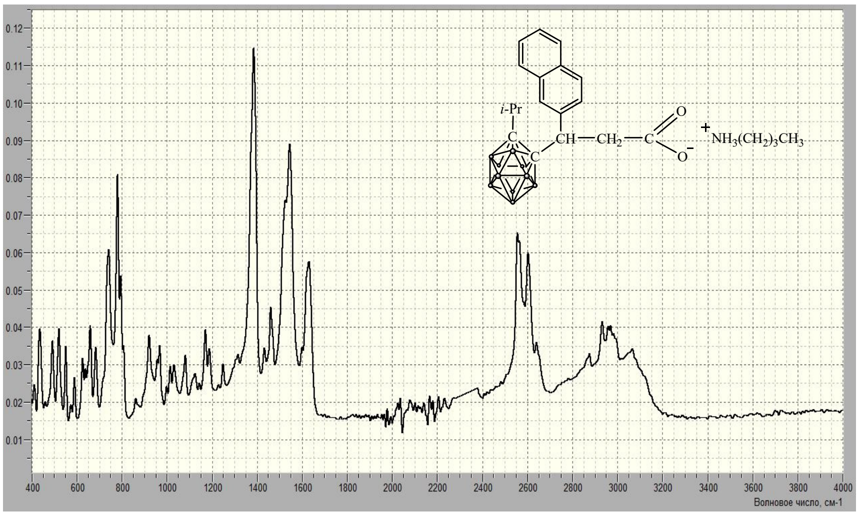

FTIR spectra of Butylammonium 3-(2-isopropyl-1,2-dicarba-closo-dodecaboran-1-yl)-3-(naphthalene-1-yl)propanoate (22)

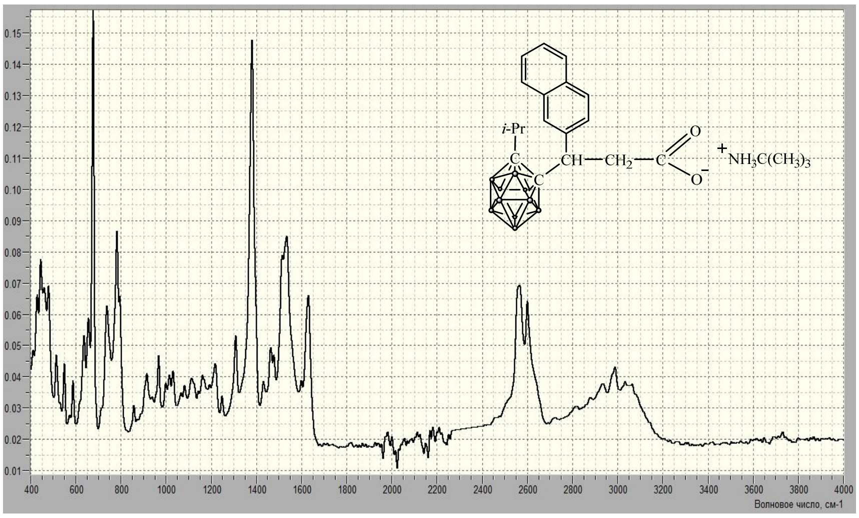

FTIR spectra of Tert-butylammonium 3-(2-isopropyl-1,2-dicarba-closo-dodecaboran-1-yl)-3-(naphthalene-1-yl)propanoate (23)

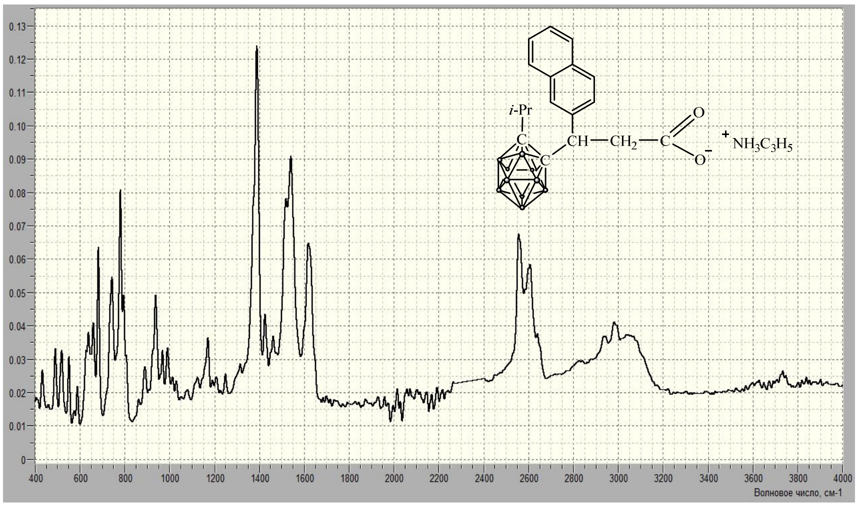

FTIR spectra of Allylammonium 3-(2-isopropyl-1,2-dicarba-closo-dodecaboran-1-yl)-3-(naphthalene-1-yl)propanoate (24)

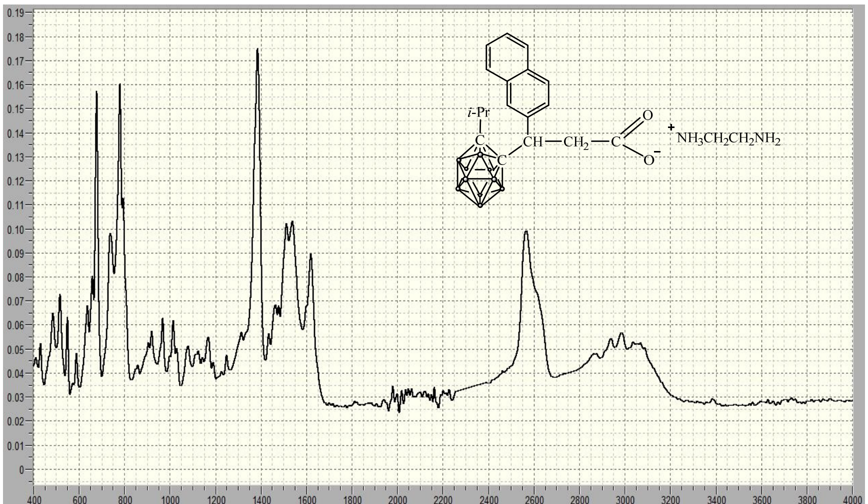

FTIR spectra of Ethan-1-amino-2-ammonium 3-(2-isopropyl-1,2-dicarba-closo-dodecaboran-1-yl)-3-(naphthalene-1-yl)propanoate (25)

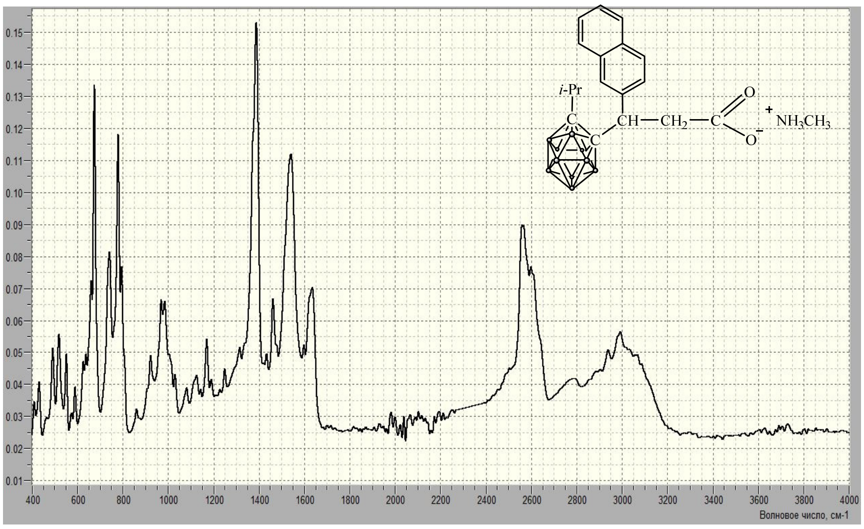

FTIR spectra of Methylammonium 3-(2-isopropyl-1,2-dicarba-closo-dodecaboran-1-yl)-3-(naphthalene-1-yl)propanoate (26)

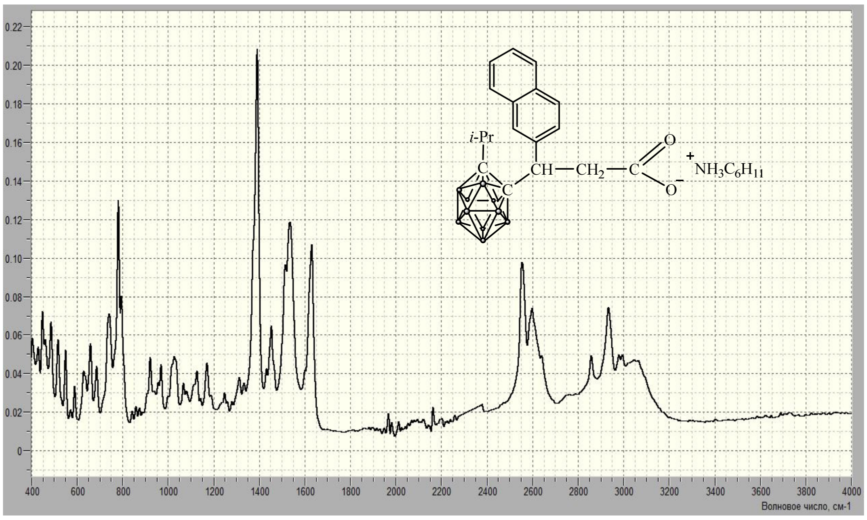

FTIR spectra of Cyclohexylammonium 3-(2-isopropyl-1,2-dicarba-closo-dodecaboran-1-yl)-3-(naphthalene-1-yl)propanoate (27)

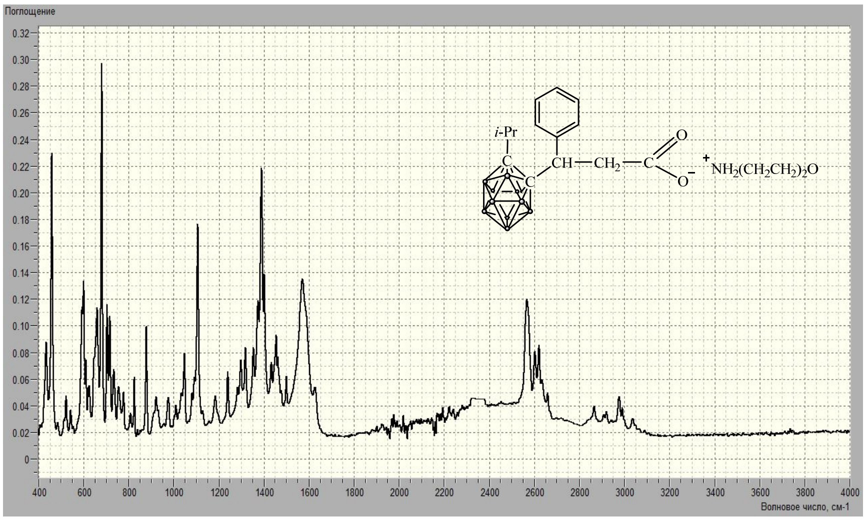

FTIR spectra of Morpholine 3-(2-isopropyl-1,2-dicarba-closo-dodecaboran-1-yl)-3-phenylpropanoate salt (28)

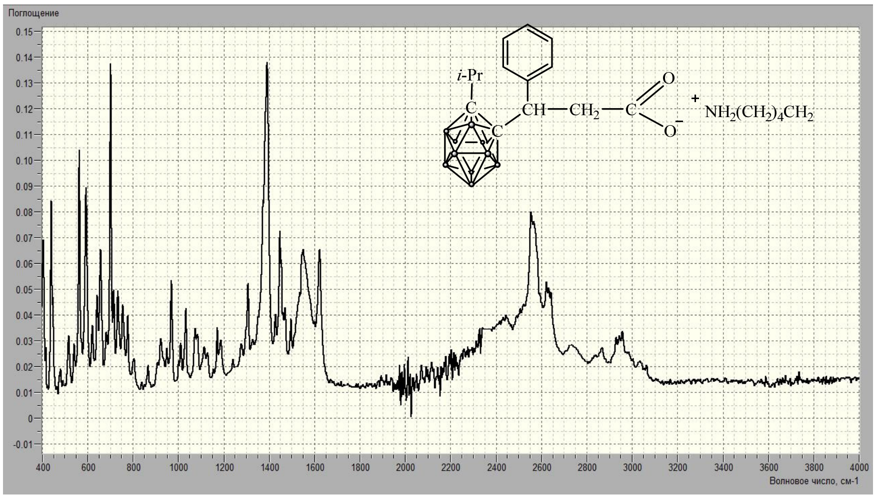

FTIR spectra of Piperidine 3-(2-isopropyl-1,2-dicarba-closo-dodecaboran-1-yl)-3-phenylpropanoate salt (29)

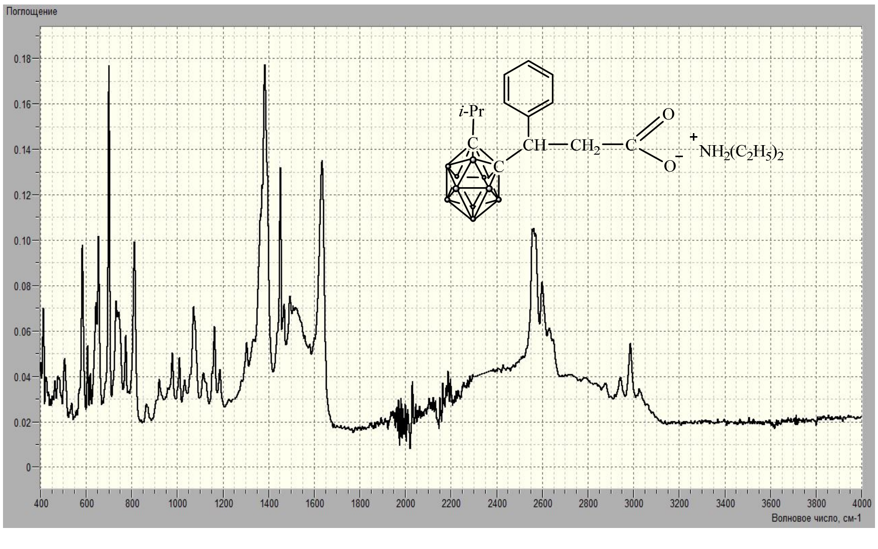

FTIR spectra of Diethylammonium 3-(2-isopropyl-1,2-dicarba-closo-dodecaboran-1-yl)-3-phenylpropanoate salt (30)

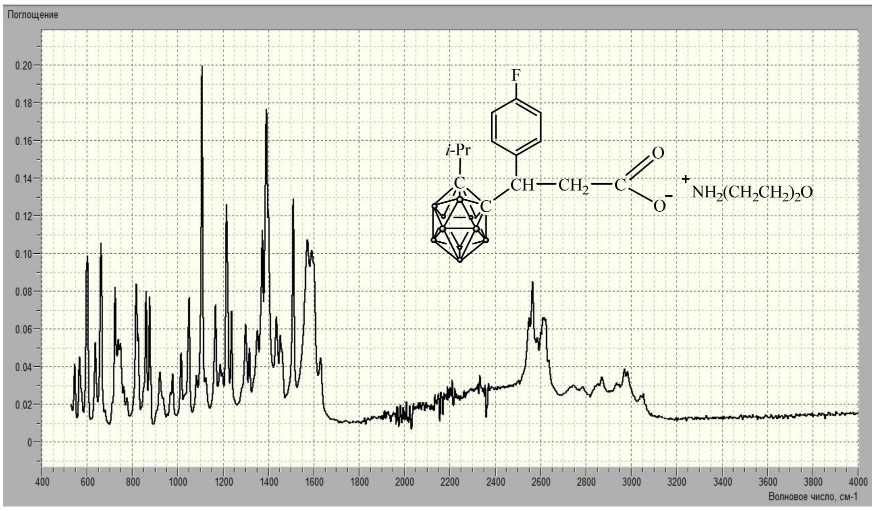

FTIR spectra of Morpholine 3-(2-isopropyl-1,2-dicarba-closo-dodecaboran-1-yl)-3-(4-fluorophenyl)propanoate salt (31)

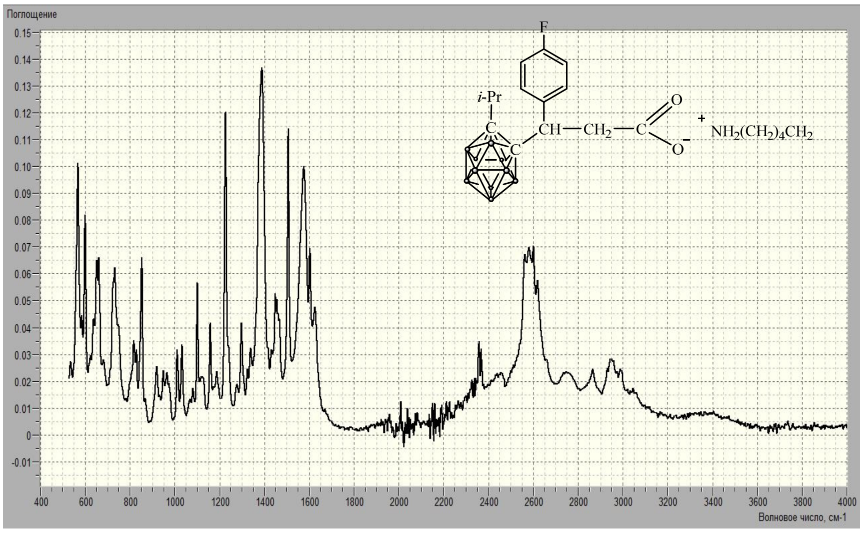

FTIR spectra of Piperidine 3-(2-isopropyl-1,2-dicarba-closo-dodecaboran-1-yl)-3-(4-fluorophenyl)propanoate salt (32)

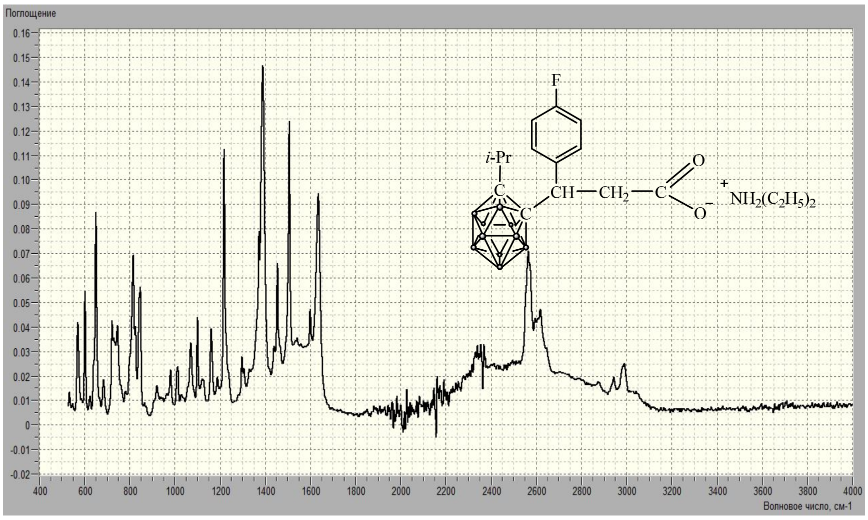

FTIR spectra of Diethylammonium 3-(2-isopropyl-1,2-dicarba-closo-dodecaboran-1-yl)-3-(4-fluorophenyl)propanoate salt (33)

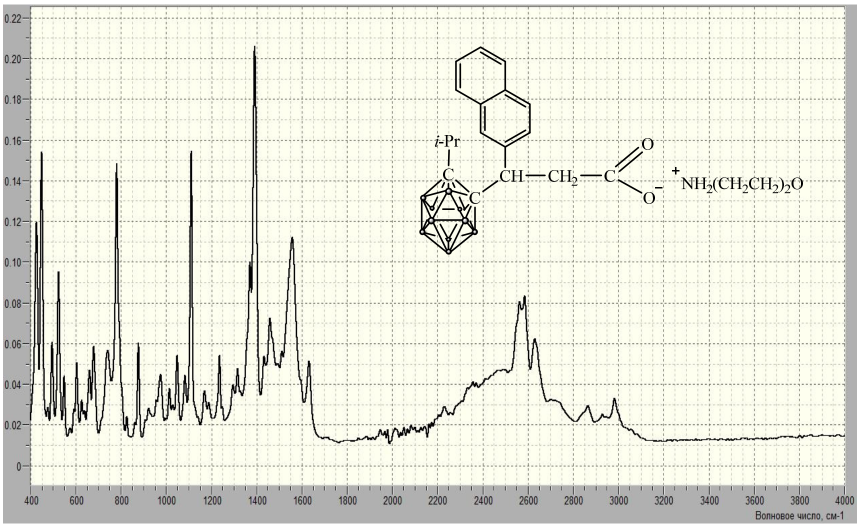

FTIR spectra of Morpholine 3-(2-isopropyl-1,2-dicarba-closo-dodecaboran-1-yl)-3-(naphthalene-1-yl)propanoate salt (34)

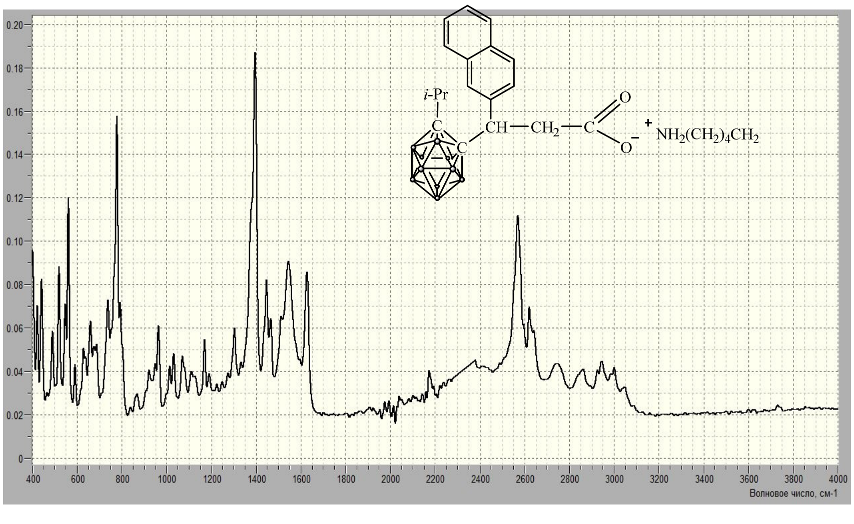

FTIR spectra of Piperidine 3-(2-isopropyl-1,2-dicarba-closo-dodecaboran-1-yl)-3-(naphthalene-1-yl)propanoate salt (35)

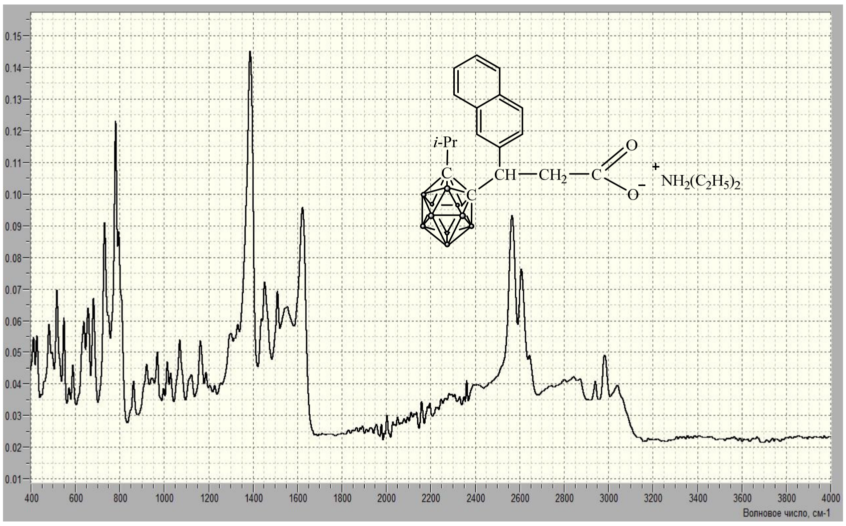

FTIR spectra of Diethylammonium 3-(2-isopropyl-1,2-dicarba-closo-dodecaboran-1-yl)-3-(naphthalene-1-yl)propanoate salt (36)

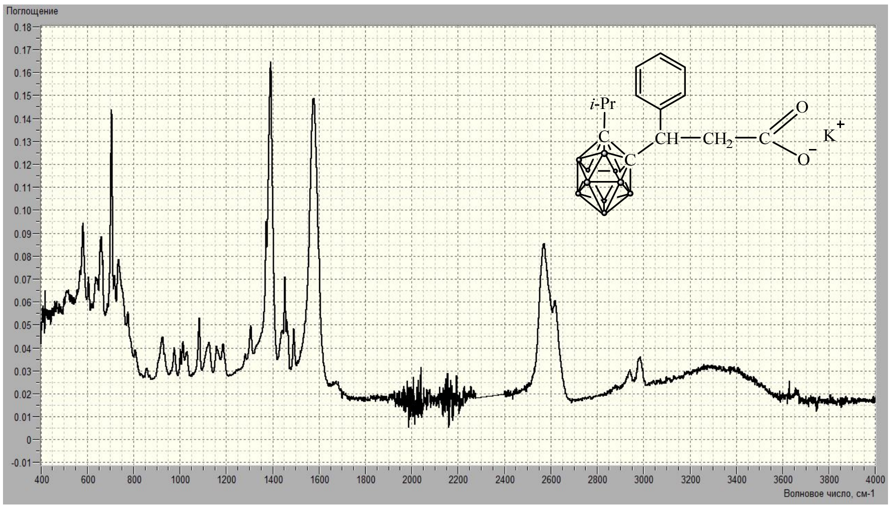

FTIR spectra of Potassium 3-(2-isopropyl-1,2-dicarba-closo-dodecaboran-1-yl)-3-phenylpropanoate (37)

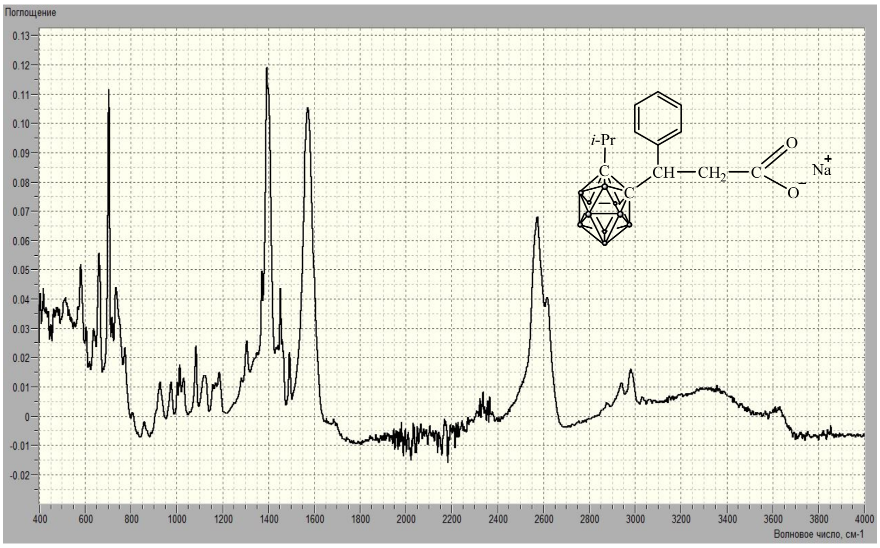

FTIR spectra of Sodium 3-(2-isopropyl-1,2-dicarba-closo-dodecaboran-1-yl)-3-phenylpropanoate (38)

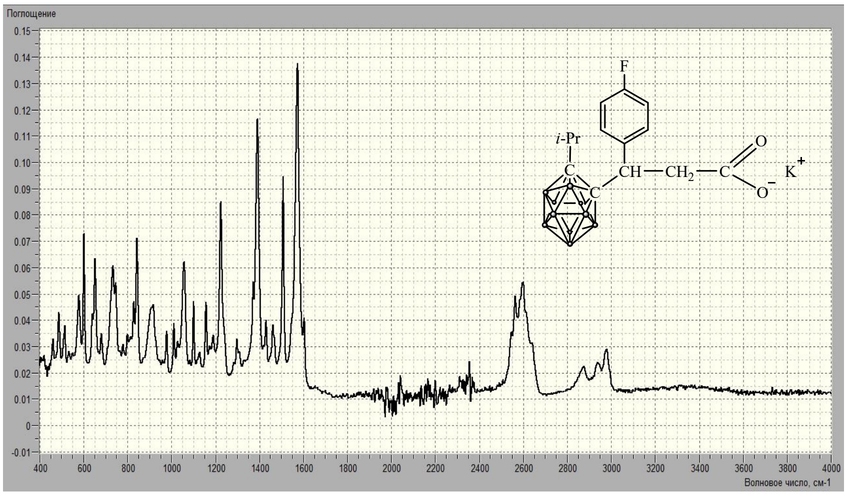

FTIR spectra of Potassium 3-(2-isopropyl-1,2-dicarba-closo-dodecaboran-1-yl)-3-(4-fluorophenyl)propanoate (39)

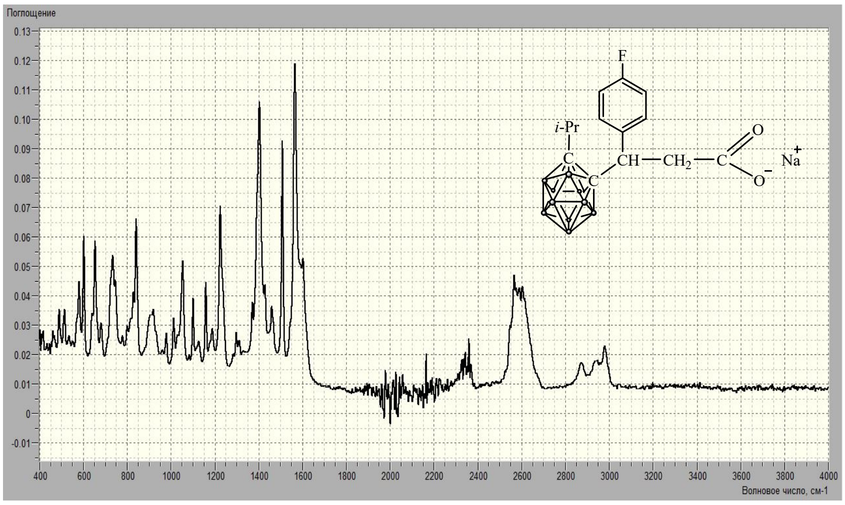

FTIR spectra of Sodium 3-(2-isopropyl-1,2-dicarba-closo-dodecaboran-1-yl)-3-(4-fluorophenyl)propanoate (40)

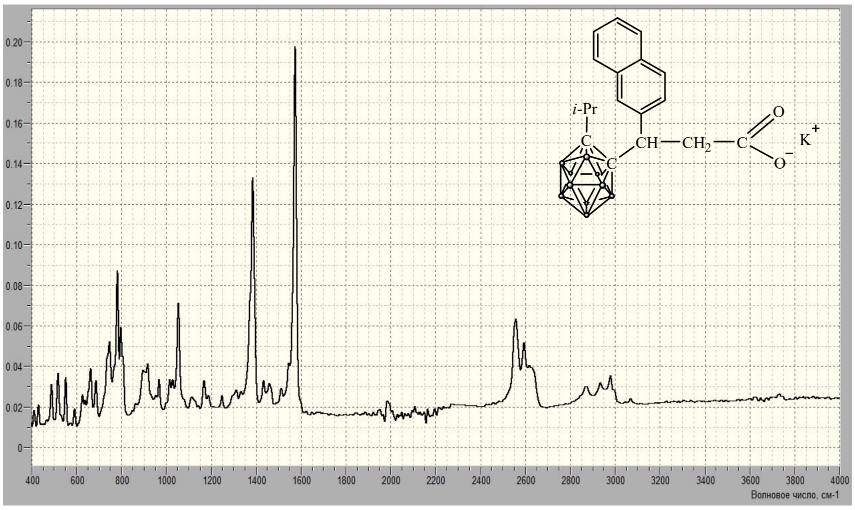

FTIR spectra of Potassium 3-(2-isopropyl-1,2-dicarba-closo-dodecaboran-1-yl)-3-(naphthalene-1-yl)propanoate (41)

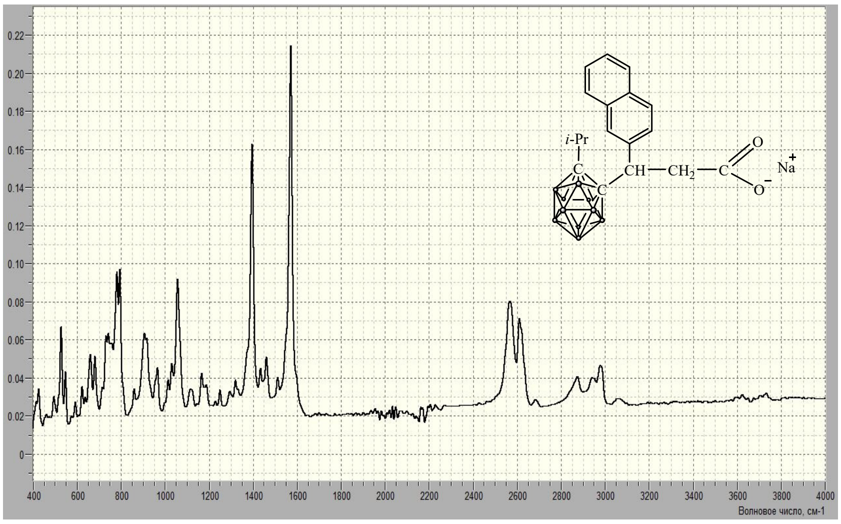

FTIR spectra of Sodium 3-(2-isopropyl-1,2-dicarba-closo-dodecaboran-1-yl)-3-(naphthalene-1-yl)propanoate (42)

HPLC

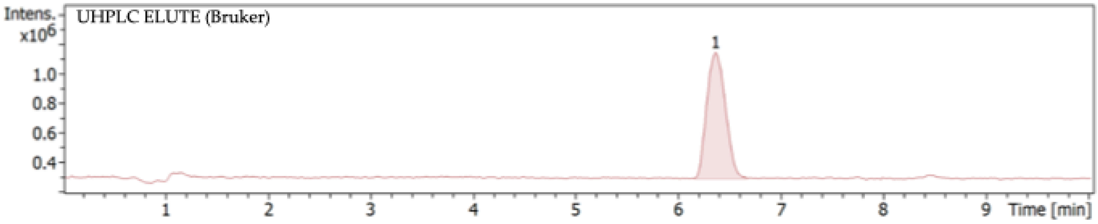

3-(2-isopropyl-1,2-dicarba-closo-dodecaboran-1-yl)-3-(4-fluorophenyl)propanoic acid (8)

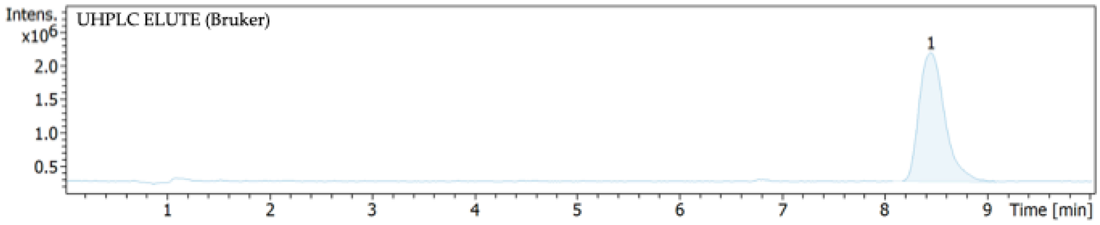

3-(2-isopropyl-1,2-dicarba-closo-dodecaboran-1-yl)-3-(naphthalene-1-yl)propanoic acid (9)

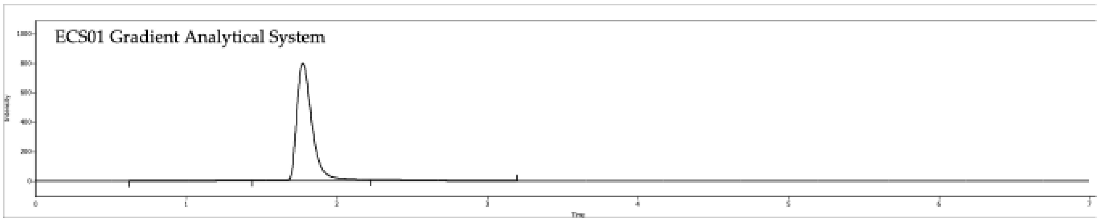

Potassium 3-(2-isopropyl-1,2-dicarba-closo-dodecaboran-1-yl)-3-phenylpropanoate (37)
